# Designing Heterolytic and Homolytic Bond Scission in Diarylmethane Mechanophores

**DOI:** 10.1021/jacsau.6c00989

**Published:** 2026-08-17

**Authors:** Gaia Egizzo, Eduardo García-Padilla, Federico Frateloreto, Florent Monie, Dnyaneshwar Ajinath Mache, Feliu Maseras, José Augusto Berrocal

**Affiliations:** † 202569 Institute of Chemical Research of Catalonia (ICIQ), The Barcelona Institute of Science and Technology (BIST), Av. Països Catalans 16, 43007 Tarragona, Spain; ‡ Department Química Analítica i Química Orgànica, Universitat Rovira i Virgili (URV), 43007 Tarragona, Spain; § Catalan Institution for Research and Advanced Studies (ICREA), Pg. Lluís Companys 23, 08010 Barcelona, Spain

**Keywords:** mechanochemical pathway control, mechanochromic polymers, radical vs ionic reactivity, force-coupled reactions, diarylmethane mechanophores

## Abstract

Mechanical force can often unlock chemical reactivity inaccessible under conventional conditions. However, understanding how molecular structure and environment govern mechanochemical reaction pathways in the solid state remains a significant challenge. In particular, establishing design principles to access heterolytic bond scission in nonpolar materials remains elusive. Here, we investigate a family of diarylmethane-based mechanophores embedded in polymer networks to elucidate how bond identity, substituent electronics, leaving-group stabilization, and matrix interactions collectively determine mechanochemical reactivity. By systematically varying the nature of the scissile bond (C–O vs C–C), the electronic properties of the diarylmethane core, and the presence or absence of hydrogen-bonding functionalities in the surrounding polymer matrix, we establish structure–reactivity relationships that govern the formation of radical and ionic species under tensile load. Mechanophores containing C–O bonds display environment-dependent behavior, with homolytic cleavage dominating in non-hydrogen-bonding matrices and heterolytic scission accessible only when both strongly donating substituents and hydrogen-bond donors are present. In contrast, mechanophores incorporating polarized C–C bonds with strongly stabilizing leaving groups undergo heterolytic cleavage to yield diarylcarbenium ions, even in matrices lacking hydrogen-bonding functionality. These results establish design principles linking molecular structure and polymer environment to mechanochemical reactivity and demonstrate that ionic pathways can be accessed and stabilized in the solid state. More broadly, this work provides guidelines for designing mechanoresponsive materials with distinct reaction pathways and mechanochromic responses.

## Graphical abstract

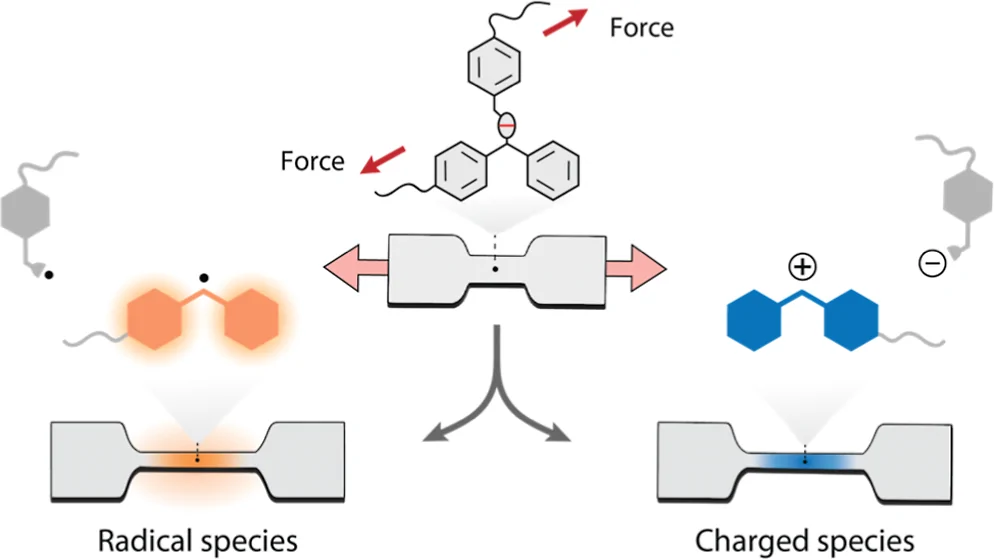

## Introduction

Mechanical force offers a unique means to promote chemical transformations by reshaping potential energy landscapes, enabling access to reaction pathways that are often inaccessible under thermal or photochemical activation.^1–5^ In polymer materials, tensile loading concentrates force along specific portions of the macromolecular backbone, where embedded mechanophores can undergo selective bond scission or structural rearrangement.^6–9^ These mechanically triggered reactions underpin a growing range of functions in polymer materials, with promising examples in sensing,^10,11^ self-repairing systems,^12^ additive manufacturing,^13^ recycling,^14^ drug delivery,^15^ and flexible devices,^16^ to name but a few.

A central challenge in polymer mechanochemistry is understanding how a mechanophore responds under load and what factors govern the resulting reaction pathway. Mechanophores are commonly categorized by their dominant cleavage mechanism, which can be homolytic or heterolytic.^17–22^ Homolytic cleavage produces radical species that are often transient but can, in some cases, be sufficiently stabilized to persist.^23,24^ Heterolytic scission generates charged fragments that either remain within a single species (i.e., zwitterions) or dissociate completely.^19^ Notable examples of force-induced homolysis have been extensively reported for dimers of stabilized radicals derived from highly conjugated frameworks.^22^ These include diarylbibenzofuranones,^25^ tetraarylsuccinonitriles,^26^ difluorenylsuccinonitriles,^27^ hexaarylbiimidazoles,^28^ and dithiomaleimides,^29^ all of which feature extensive π-delocalization and sterically protected radical centers that promote reversible homolytic bond cleavage under mechanical stress. Additionally, nonchromogenic mechanophores such as gem-difluorocyclopropanes also undergo force-triggered homolytic bond scission, without generating an optical signal.^30^ All these systems consistently produce observable radicals under force, reflecting both the stabilizing effects of delocalized unpaired electron density and the limited ability of solid polymer matrices to stabilize charge-separated species.

In contrast, heterolytic mechanochemical pathways in polymer networks remain considerably less common, reflecting the inherent difficulty of stabilizing charged species in low-polarity solid environments. Previous experimental observations and theoretical studies have shown that the balance between homolytic and heterolytic pathways can depend on both molecular structure and environment, highlighting the role of polarity and hydrogen-bonding interactions, as well as the intrinsic stability of radical and ionic intermediates.^31–35^ In particular, our recent work on triarylmethane-based mechanophores embedded in poly­(methyl acrylate-*co*-2-hydroxyethyl acrylate) (P­(MA-*co*-HEA)) networks demonstrated that hydrogen-bonding interactions in the polymer matrix can stabilize carbocationic intermediates and enable heterolytic C–O bond scission in the solid state.^34,36,37^ Despite these advances, establishing simple and transferable design principles that enable heterolytic bond scission in polymer solids remains an open challenge.

A mechanophore platform that systematically evaluates molecular structure and environment provides an effective approach to address this challenge. Diarylmethanes can serve as a compelling framework in this context. Their connectivity resembles that of well-established radical-forming motifs,^25–27^ while their corresponding diarylcarbenium ions have been extensively characterized in solution in terms of substituent-dependent stability and electrophilicity.^38–41^ However, mechanophores based on classical diarylmethane architectures—i.e., CH-centered diphenylmethane scaffolds capable of forming either radicals or carbocations—have not been explored under mechanical activation, and the interplay between radical and ionic pathways in such systems remains largely unexamined in the solid state.^42^

Here, we report the first family of diarylmethane mechanophores that display either homolytic or heterolytic bond scission in solid-state polymers under tensile load (Figure 1). By systematically varying aromatic substituents, leaving-group identity, and the hydrogen-bonding characteristics of the surrounding polymer matrix, we identify how these factors collectively govern the preference for homolytic or heterolytic bond scission. Our results show that substituent electronics define the intrinsic propensity toward radical or ionic pathways, while matrix interactions determine whether heterolytic processes can be expressed in the solid state. In addition, we demonstrate that appropriate leaving-group design enables heterolytic C–C bond scission independently of matrix stabilization, providing an alternative route to ionic reactivity under force. Together, these findings define structure–reactivity relationships for diarylmethane mechanophores and establish design principles linking molecular structure and polymer environment to mechanochemical outcomes. More broadly, this work shows that concepts from solution-phase physical organic chemistry, such as carbocation and radical stabilization, can be translated to the solid state to guide the design of mechanophores with distinct and well-defined reactivity (Figure 1).

**Figure 1:**
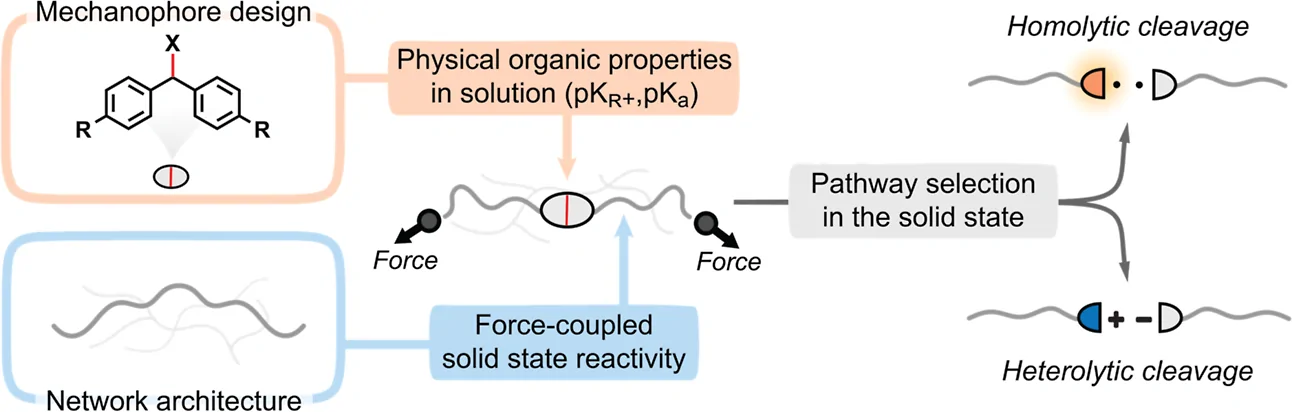
Conceptual illustration of force-induced pathway selection in diarylmethane mechanophores embedded in solid polymer networks, highlighting how solution-phase physical organic properties (e.g., p*K*_R+_, p*K*_a_) can be translated into well-defined solid-state reactivity.

## Result and Discussion

### Design of Diarylmethane Derivatives and Polymer Network Preparation

To disentangle how intrinsic electronic structure and polymer matrix environment govern force-induced reactivity, we prepared a family of four diarylmethane derivatives (**Dr-1–4**, Figure 2a). Their design varies both the substituent electronics on the diaryl core and the identity of the counterfragment at the scissile bond.

**Figure 2:**
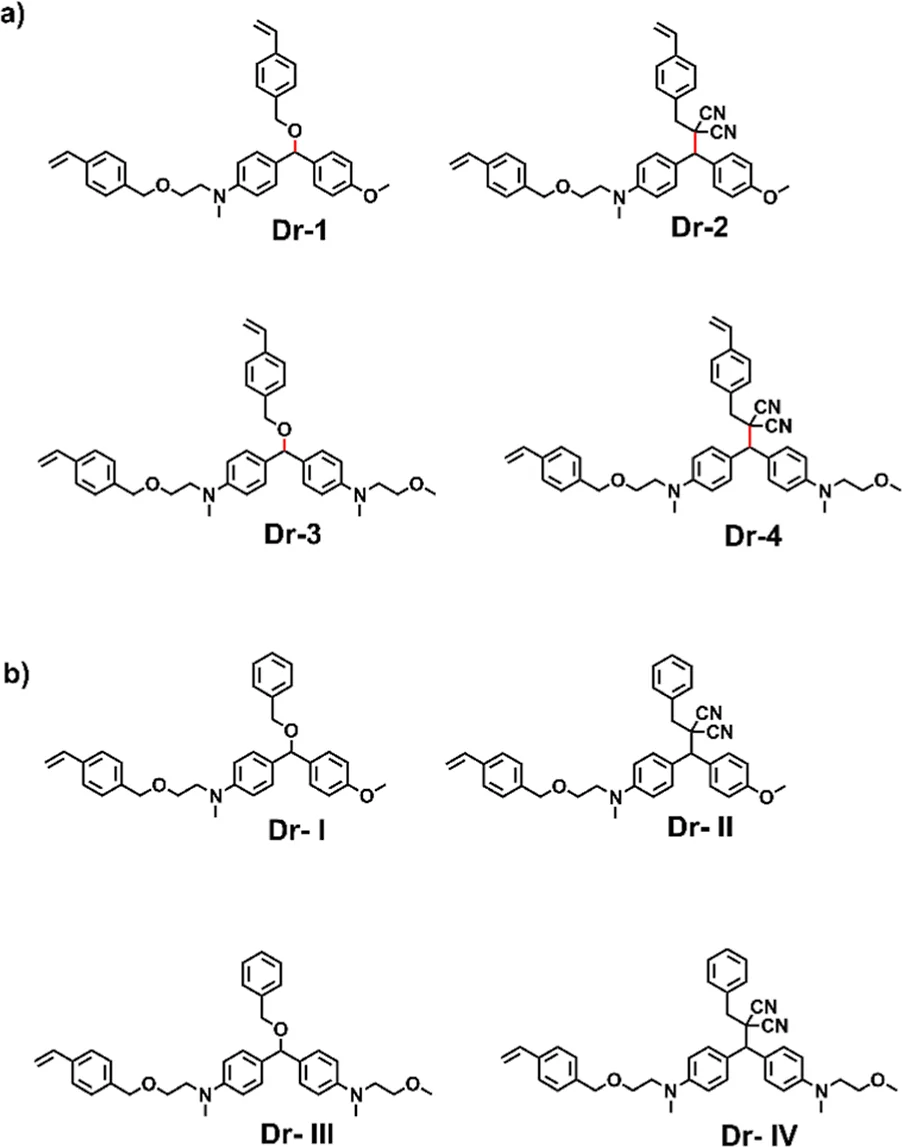
(a) Diarylmethane structures experimentally investigated **Dr-1–4**. (b) Control diarylmethane structures **Dr-I–IV**.

**Dr-1** and **Dr-3** retain the diarylmethane–benzyl ether connectivity previously used by our group in triarylmethane architectures.^36,37^ **Dr-2** and **Dr-4** incorporate a benzyl malononitrile leaving-group, instead. This structural variation introduces a π-extended, more electron-withdrawing motif compared to the benzyl ether of **Dr-1** and **Dr-3**. Importantly, this modification also changes the connectivity from a C–O to a C–C weak bond. Together, these four structures form two matched electronic pairs (i.e., **Dr-1** and **Dr-2**, and **Dr-3** and **Dr-4**) that should enable us to evaluate how substituent effects and leaving-group stabilization bias mechanochemical pathways.

**Dr-1–4** were designed with two styrenyl groups to enable covalent incorporation as cross-linkers and thus allow these diarylmethane skeletons to experience well-defined tensile loading within polymer networks. Corresponding monofunctional analogs **Dr-I–IV** were also synthesized as controls (Figure 2b). The detailed synthesis of **Dr-1–4** and **Dr-I–IV** is discussed in the electronic Supporting Information (ESI) (Pages S6–S8).

**Dr-1–4** were incorporated as cross-linkers at 1 mol % concentration into poly­(methyl acrylate) (PMA) or (P­(MA-*co*-HEA)) networks (containing 10 wt % of HEA monomer in the formulation) via UV-induced photopolymerization, affording **PMA-Dr-1–4** and **P­(MA-** *co* **-HEA)-Dr-1–4** (Figure 3a,b) (Pages S67–S69).

**Figure 3:**
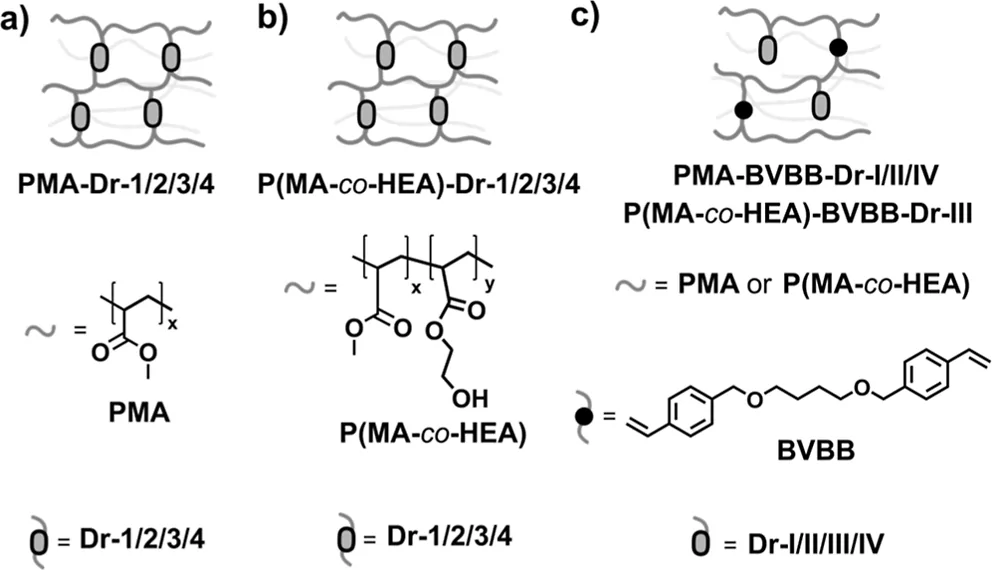
(a) Schematic illustration of the PMA network series. (b) Schematic illustration of the P­(MA-*co*-HEA) network series. (c) Schematic illustration of the control polymer series with the nonmechanoresponsive cross-linker **BVBB**.

Using 1 mol % of 1,4-*bis*((4-vinylbenzyl)­oxy)­butane (**BVBB**) as a cross-linker and 1 mol % of **Dr-I–IV** as pendent groups, we also prepared control polymer networks **PMA-BVBB-Dr-I**/**II**/**IV** and **P­(MA-** *co* **-HEA)-BVBB-Dr-III** (Figure 3c; Pages S67–S69). We anticipate that the choice of polymer matrices for control polymers **PMA-BVBB-Dr-I**/**II**/**IV** and **P­(MA-** *co* **-HEA)-BVBB-Dr-III** mirrors the conditions in which their bifunctionalized counterparts (i.e., **Dr-1/2/4** and **Dr-3**) displayed mechanochemical activation (vide infra).

The glass transition temperature (*T*_g_) of all PMA and P­(MA-*co*-HEA) networks was determined by differential scanning calorimetry (DSC) and was found to be below room temperature (≤23 °C) (Supporting Information, Pages S87–S103, Tables S4–S6). The *T*_g_ values found are consistent with reported data for cross-linked PMA and with our previous studies on triarylmethane systems.^36,37^ Finally, gel content measurements confirmed that cross-link densities were consistent across the entire scope of investigated polymers (Pages S70 and S71, Table S1).

### Force-Responsive Behavior of Polymer Networks Comprising Benzylether-Based Derivatives **Dr-1** and **Dr-3**

To evaluate how aromatic substituents and matrix polarity influence mechanochemical pathway selection, we first investigated the benzyl-ether diarylmethanes **Dr-1** and **Dr-3**. These two structures differ primarily in the electron-donating strength of the substituents and therefore probe how carbocation stability and radical delocalization compete under force. The polymer films were subjected to uniaxial tensile deformation at a strain rate of 2.2%·s^–1^ (375 μm·s^–1^) at 25 °C.

The first system we studied was **PMA-Dr-1**, whose stress–strain curve revealed a profile characteristic of amorphous, cross-linked elastomers, with strain hardening above 150%, and stress and strain at break values of ca. 20 MPa and above 300%, respectively (Figures 4a and S82).

**Figure 4:**
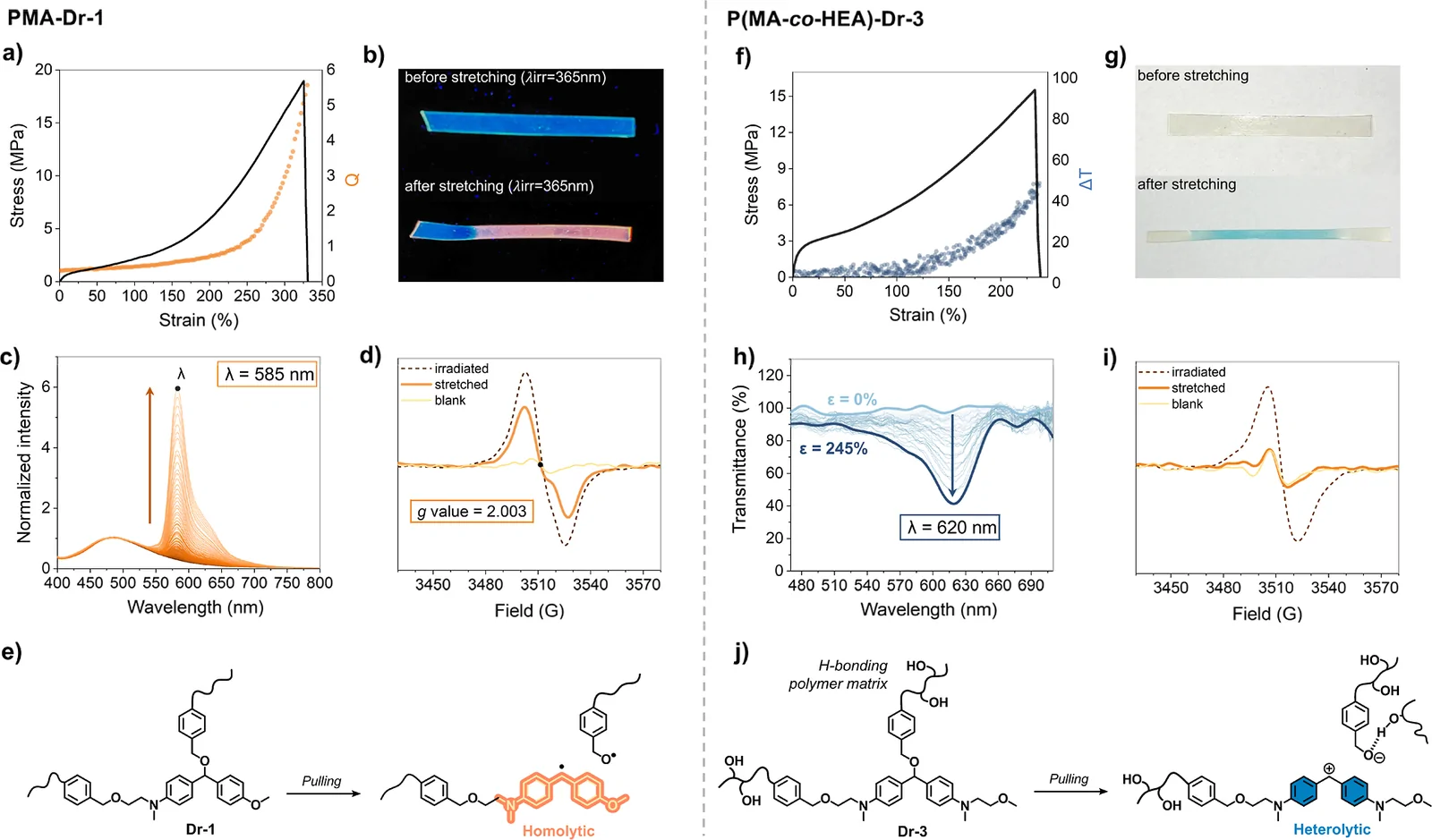
(a) Stress–strain curve and evolution of *Q*, defined as the ratio between emission at 585 nm and the reference at 490 nm, in **PMA-Dr-1** films (strain rate of 2.2%·s^–1^). (b) Photographs of **PMA-Dr-1** films before and after being manually pulled. (c) Emission spectrum of **PMA-Dr-1** under 365 nm irradiation measured during the uniaxial elongation experiments (strain rate of 2.2%·s^–1^). (d) EPR spectrum of **PMA-Dr-1** films measured before and after being manually pulled, and after irradiation with a Hg lamp. (e) Schematic representation of the force-induced C–O homolytic bond scission of **Dr-1**. (f) Stress–strain curve and evolution of Δ*T* of **P­(MA-** *co* **-HEA)-Dr-3** (strain rate of 2.2%·s^–1^). Δ*T* is the difference between transmittance at the target wavelength (620 nm) and a reference (720 nm). (g) Photographs of **P­(MA-** *co* **-HEA)-Dr-3** films before and after being manually pulled. (h) Transmittance spectrum of **P­(MA-** *co* **-HEA)-Dr-3** measured during the uniaxial elongation experiments (strain rate of 2.2%·s^–1^). (i) EPR spectrum of **P­(MA-** *co* **-HEA)-Dr-3** measured before and after being manually pulled, and after irradiation with a Hg lamp. (j) Schematic representation of the force-induced C–O heterolytic bond scission of **Dr-3** in a polymer matrix doped with hydrogen-bond donors.

Pulling on **PMA-Dr-1** films revealed mechanochromism, as the films turned from colorless to highly emissive (fluorescence) upon illumination with 365 nm light (Figure 4b; Supporting Information Movie S1). Thus, the uniaxial elongation experiments were repeated under constant excitation at 365 nm, with the films’ emission captured by an optical probe connected to a spectrophotometer. The films exhibited a pre-existing band centered at 490 nm in the resting state, i.e., without pulling, which is ascribed to the intrinsic fluorescence of **Dr-1** (Figure S33). However, a new band centered at 585 nm, accompanied by a shoulder at 640 nm, gradually emerged during deformation (Figure 4c). Since the relative intensities of the two emission bands at 490 and 585 nm changed systematically with strain and time, we probed the evolution of the fluorescence through their ratio *Q* (*Q* = *I*_490_/*I*_585_) (Figure 4a). The plot of *Q* as a function of elongation reveals that the onset of emissive behavior for **Dr-1** occurs above ≈150% strain. Above this elongation value, the emission increases sharply (Figure 4a).

Based on previously reported examples of diarylbibenzofuranones, tetraarylsuccinonitriles, and difluorenylsuccinonitriles mechanophores, whose related radicals displayed emission bands in the region of 560–610 nm (orange-red) under mechanical stress,^25–27^ we hypothesized that the mechanofluorochromism observed with deformed **PMA-Dr-1** films could be ascribed to diarylmethane radicals produced via homolysis of the C–O bond. Thus, we performed electron paramagnetic resonance (EPR) measurements on **PMA-Dr-1** films before and after stretching to detect radical species. The EPR spectrum of unstretched **PMA-Dr-1** showed no significant signal attributable to a radical species, while, after stretching, it displayed a signal with *g* = 2.003, which is characteristic of carbon-centered radicals (Figures 4d and S104).^43^ The same signal was also observed upon powerful photoirradiation of the film (mercury lamp, λ = 200–2000 nm, power intensity of 100 W, irradiation time of 1 h)—i.e., without mechanical load—which suggests that the **Dr-1** moieties in **PMA-Dr-1** can undergo homolytic cleavage in the solid state also photochemically. Notably, the EPR signal of the photogenerated species is more intense. This difference can be explained by the distinct populations of moieties effectively addressed by the two stimuli: during a stretching event, only a fraction of embedded mechanophores is expected to experience forces above the activation threshold, whereas photoexcitation of a thin polymer film should activate a larger population of chromophores. Taken together, these observations provide convincing evidence that stretching of **PMA-Dr-1** triggers homolytic cleavage of the C–O bond of **Dr-1**, giving rise to emissive diarylmethane radicals (Figure 4e). The mechanically generated fluorescence decays markedly more slowly under an argon atmosphere than under air (Figure S77), supporting oxygen-mediated quenching of the diarylmethane radical species. The observed behavior is consistent with oxidative pathways leading to ketone products.^44^ Radical–radical recombination cannot be excluded, although previous studies suggest ketone formation as the more plausible process.^27^ These results point to the fact that weakly donating substituents enforce a purely homolytic response in environments lacking hydrogen-bond-donors, suggesting that intrinsic electronic bias dominates when matrix stabilization is minimal.

Control experiment on **PMA-BVBB-Dr-I**, in which **Dr-I** serves as a pendent, revealed that luminescence can only be achieved upon irradiation with UV light in these films (Figure S76). Further, mechanical deformation of **PMA-BVBB-Dr-I** did not produce any change in the optical properties, thus confirming the mechanochemical nature of the activation of **Dr-1** in **PMA-Dr-1**.

We then carried out similar experiments on **P­(MA-** *co* **-HEA)-Dr-1**. Previous studies in P­(MA-*co*-HEA) matrices with triarylmethane mechanophores similarly built to **Dr-1** demonstrated that “doping” the polymer matrix with hydrogen-bond donors (i.e., HEA) facilitates the heterolysis of the C–O bond.^36,37^ However, **P­(MA-** *co* **-HEA)-Dr-1** films remained colorless upon uniaxial elongation, showing a lack of mechanical activation. This behavior contrasts starkly with that of acetonitrile solutions of the carbinol precursor of **Dr-1**, which yielded a red carbocation upon treatment with boron trifluoride diethyl etherate (Scheme S4 and Figure S75). Additional support for the assignment of the colored species as diarylcarbenium ions is provided by ^1^H NMR characterization of independently generated carbocations in solution, which display the characteristic downfield shift of the benzylic resonance adjacent to the cationic center (Figures S73 and S74). Thus, we hypothesized that the lack of heterolytic cleavage in **P­(MA-** *co* **-HEA)-Dr-1** reflects insufficient stabilization of the fragments generated upon bond cleavage. Indeed, methoxy-substituted diarylcarbenium ions (e.g., derived from **Dr-1**) are known to possess a lower p*K*_R_+ (ca. −5.65) compared to similar carbocations featuring two dimethylamino substituents (e.g., derived from **Dr-3**) (p*K*_R_+ of ca. + 5.61).^38–40^ Hence, it appears that **Dr-1** is locked into a homolytic pathway in **PMA-Dr-1**. We speculate that the absence of detectable activation in **P­(MA-** *co* **-HEA)-Dr-1** does not imply that heterolysis is impossible, but rather that the polymer environment is insufficient to shift the free-energy landscape into a regime where either pathway generates detectable concentrations of products.^36^

Following this hypothesis of the typical solution-phase stabilization parameters (p*K*_R_+), we proceeded to study **PMA-Dr-3** and **P­(MA-** *co* **-HEA)-Dr-3**. Interestingly, the deformation of **PMA-Dr-3** did not produce any detectable effect (Figure S80), whereas the uniaxial elongation of **P­(MA-** *co* **-HEA)-Dr-3** resulted in a clear response. The stress–strain curve of **P­(MA-** *co* **-HEA)-Dr-3** revealed a similar profile to that of **PMA-Dr-1**, but with stress and strain at break values of approximately 16 MPa and above 250%, respectively (Figure 4f). Upon uniaxial elongation, the gauge section of the **P­(MA-** *co* **-HEA)-Dr-3** films changed from colorless to deep blue (Figures 4g and S74, Supporting Information Movie S2). The visual observation was confirmed by real-time transmission spectra recorded during tensile testing, which showed an absorption band centered at 620 nm at approximately 120% strain (Figure 4h). Upon unloading, the blue coloration gradually faded over approximately 1 h (under air at room temperature) (Figure S79). To correlate the optical signal with the stress–strain curve, we plotted Δtransmittance (Δ*T*)^36^ as a function of strain %, where Δ*T* is the difference between the transmittance measured at 620 nm and a reference wavelength of 720 nm (Figure 4f). Δ*T* allows us to mitigate baseline shifts during the measurements, compensating for sample thinning while pulling.^36^ The Δ*T* vs strain % profile of **P­(MA-** *co* **-HEA)-Dr-3** revealed a linear correlation with the applied strain above ca. 120%.

EPR measurements performed on **P­(MA-** *co* **-HEA)-Dr-3** films before and after pulling showed no significant difference between the two traces (Figures 4i and S108). These results are in line with the lack of emissive species—i.e., diaryl radicals—detected upon irradiation with UV light (365 nm) during/after pulling (Figure S80). In contrast, exhaustive photoirradiation (λ = 200–700 nm, power intensity of 100 W, irradiation time of 1 h) of the polymer produced an intense signal at 3515 G, with a *g* value of 2.003, highlighting that homolysis is the preferred photochemical pathway. Control experiments carried out on **P­(MA-** *co* **-HEA)-BVBB**-**Dr-III** further supported the mechanochemical nature of the activation of **Dr-3** in **P­(MA-** *co* **-HEA)-Dr-3**: no mechanochromism and silent EPR spectra were observed upon uniaxial elongation of these films (Figures S80,S81 and S112). Altogether, these experiments strongly point to C–O heterolysis in **P­(MA-** *co* **-HEA)-Dr-3**, with the consequent formation of carbocations (Figure 4j).

As a first partial conclusion, the results obtained with **Dr-1** and **Dr-3** suggest that the aromatic substituents tune the intrinsic balance between homolytic and heterolytic pathways, but the matrix design determines whether heterolysis can ultimately occur in the solid state. In other words, **Dr-1** and **Dr-3** form an instructive pair in which substituent electronics set the intrinsic thermodynamic preference, while the presence or absence of hydrogen-bond donors in the matrix dictates whether this preference can be expressed in the solid state. This interplay between substituent effects and matrix-enabled stabilization is a central component of the structure-dependent pathway-selection developed in this work.

### Force-Responsive Behavior of Polymer Networks Comprising Malononitrile-Based Derivatives **Dr-2** and **Dr-4**

The stress–strain curves of **PMA-Dr-2** and **P­(MA-** *co* **-HEA)-Dr-2** were qualitatively similar to those shown in Figure 4a,f (Figures 5a and S78–S81, Supporting Information Movie S3). Stress and strain at break values were approximately 20 MPa and above 410% in this case (using a strain rate of 30%·s^–1^, vide infra) (Figure 5a). Manual stretching of **PMA-Dr-2** and **P­(MA-** *co* **-HEA)-Dr-2** films led to the rapid appearance of red coloration (Figures 5b and S74), which faded promptly upon load release. In contrast, no perceptible color change was detected in the gauge section when the films were pulled with our microtensile tester at a strain rate of 2.2%·s^–1^ (375 μm·s^–1^). Increasing the strain rate to 30%·s^–1^ (5000 μm·s^–1^) yielded the same red coloration observed upon manual stretching. This rate-dependence is consistent with the transient nature of the chromophore: at low strain rate, the lifetime of the colored species is short relative to the time scale of deformation and therefore does not accumulate to levels detectable in transmission, whereas at higher strain rate the rate of generation and the rate of deformation are more closely matched. These observations suggest that, for **Dr-2**, effective optical readout requires deformation rates that outpace the relaxation of the colored state. Also in this case, although the coloration fades quickly (ca. 5 s) upon unloading (under air at room temperature; Figure S79), repeated loading–unloading cycles can produce the chromogenic response multiple times (i.e., 2–3×) prior to sample failure. This suggests that only a fraction of the mechanophores is activated during each deformation event.

**Figure 5:**
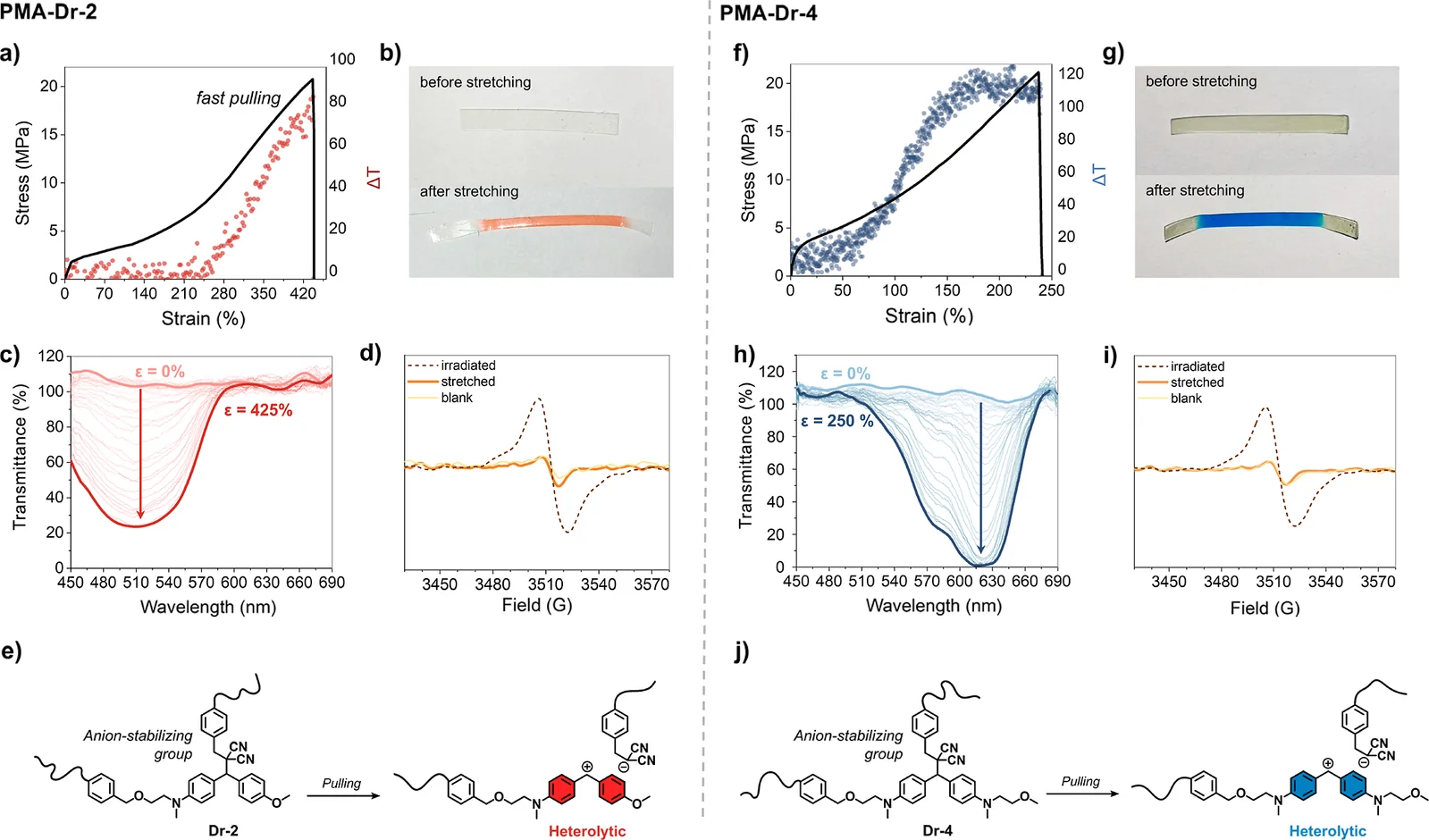
(a) Stress–strain curve and evolution of Δ*T* of **PMA-Dr-2** (strain rate of 30%·s^–1^). Δ*T* is the difference between transmittance at the target wavelength (525 nm) and a reference (700 nm). (b) Photographs of **PMA-Dr-2** films before and after being manually pulled. (c) Transmittance spectrum of **PMA-Dr-2** measured during the uniaxial elongation experiments (strain rate of 30%·s^–1^). (d) EPR spectrum of **PMA-Dr-2** films measured before and after being manually pulled, and after irradiation with a Hg lamp. (e) Schematic representation of the force-induced C–C bond cleavage of **Dr-2**. (f) Stress–strain curve and evolution of Δ*T* of **PMA-Dr-4** (strain rate of 2.2%·s^–1^). Δ*T* is the difference between transmittance at the target wavelength (620 nm) and a reference (720 nm). (g) Photographs of **PMA-Dr-4** films before and after being manually pulled. (h) Transmittance spectrum of **PMA-Dr-4** measured during the uniaxial elongation experiments (strain rate of 2.2%·s^–1^). (i) EPR spectrum of **PMA-Dr-4** films measured before and after being manually pulled, and after irradiation with a Hg lamp. (j) Schematic representation of the force-induced C–C bond cleavage of **Dr-4**.

Real-time monitoring of the transmittance spectrum of **PMA-Dr-2** films using our home-built setup revealed a band at 525 nm, whose intensity increased during deformation (Figure 5c). Using the Δ*T* mitigation (defined, in this case, as the difference between the transmittance at 525 and 700 nm), we observed a linear correlation between Δ*T* and the applied strain from ca. 200% strain (Figure 5a).

Similarly to what previously discussed for **P­(MA-** *co* **-HEA)-Dr-3**, EPR measurements on **PMA-Dr-2** showed a silent spectrum before and after stretching, while an intense signal was detected when an unstretched sample was irradiated with the powerful UV lamp of our EPR apparatus (Figures 5d and S102). Similar results were obtained with **P­(MA-** *co* **-HEA)-Dr-2** (Figure S79), whereas control experiments on **PMA-BVBB-Dr-II** showed no mechanochromism, supporting the mechanochemical nature of the activation of **Dr-2** (Figure S81). These observations indicate that polymer networks incorporating **Dr-2** cross-links consistently afford heterolytic C–C bond scission, producing a (short-lived) diarylmethane carbenium ion and a malononitrile carbanion, regardless of the design of the polymer matrix (Figure 5e). These results strongly suggest that the malononitrile fragment stabilizes the charge-separated state sufficiently even in the absence of hydrogen-bond donors. Thus, **Dr-2** highlights a distinct new design rule in which stabilization of the negative charge by the leaving-group can significantly reduce the influence of matrix effects, providing a complementary mechanism for enforcing heterolysis in the solid state.

Finally, we studied **PMA-Dr-4** and **P­(MA-** *co* **-HEA)-Dr-4** films under uniaxial tensile deformation at a strain rate of 2.2%·s^–1^ (375 μm·s^–1^). Both materials exhibited stress and strain at break values of approximately 20 MPa and 250%, respectively (Figures 5f, S80 and S82) and produced an intense blue coloration upon pulling (Figures 5g and S74, Supporting Information Movie S4). The latter was confirmed by the presence of an absorption band centered at 620 nm in the transmittance spectra (Figure 5h). The intensity of such a band was significantly more pronounced than in the case of **PMA-Dr-2** and **P­(MA-** *co* **-HEA)-Dr-2**, causing the detector to reach almost complete saturation (i.e., nearly complete loss of transmittance at high tensile strains) (Figure 5h). This behavior can be attributed to the fact that the extinction coefficient of the blue-colored species, namely the **Dr-3/4**-derived carbocations, in acetonitrile solution is almost 3× higher than that of the red **Dr-1/2**-derived carbocations (Page S66). In addition, we speculate that the C–C bond in **Dr-4** is more prone to cleavage than in **Dr-2**, as the final product benefits from a different stabilization due to the different p*K*_R_+ of the carbocations. A plot of the Δ*T* signal (analogously to **P­(MA-** *co* **-HEA)-Dr-3**, in this case, Δ*T* is defined as the difference between the transmittance at 620 nm and at the reference of 720 nm) as a function of applied strain showed, once again, a linear correlation, albeit starting at strain values as low as approximately 80% this time (Figure 5f). Finally, EPR spectra showed no detectable radical signal upon mechanical deformation (Figure 5i), whereas no mechanochemical activation was detected with control **PMA-BVBB-Dr-IV** (Figure S81). Thus, the evidence strongly suggests that **Dr-4** functions as a robust, matrix-independent heterolytic mechanophore (Figure 5j), producing a high-contrast, persistent signal. Similarly to what previously discussed for **PMA-Dr-2**, also the coloration of the pulled **PMA-Dr-4** films fades upon unloading after 12 h, under air, at room temperature (Figure S79). Compared with **PMA-Dr-2**, the longer lifetime of the generated species reflects the higher intrinsic stability of the **Dr-4**-derived carbocation. Repeated loading–unloading cycles (i.e., 2–3×) can still generate a detectable chromogenic response until mechanical failure of the sample.

All in all, the outcome of the mechanochemical activation experiments performed with polymer networks comprising malononitrile-based **Dr-2** and **Dr-4** cross-links strongly points to the fact that when the departing fragment efficiently stabilizes the negative charge, heterolytic cleavage is the strongly preferred mechanochemical pathway, regardless of matrix effects. Thus, **Dr-2** and **Dr-4** demonstrate that leaving-group design enables a second, matrix-independent route to ionic reactivity under force, depicting a two-dimensional design landscape (substituent electronics and leaving-group stabilization) for structure- and environment-dependent pathway selection.

### Computational Studies

To unify the mechanistic insights emerging across **Dr-1** through **Dr-4** and to quantify the interplay between electronic bias and environmental stabilization, we performed density functional theory (DFT) analyses on model compounds **Dr-A**–**D** (Figure 6). These compounds mirror the electronic structures of the experimental mechanophores, albeit with polymer chains replaced by methyl groups. This would allow the determination of the nature of the bond cleavage and the relative stability of the cleaved mechanophore fragments. In particular, the relative thermodynamic stability of the products was assessed to understand the influence of the *para* substituents as well as that of the leaving-group.

**Figure 6:**
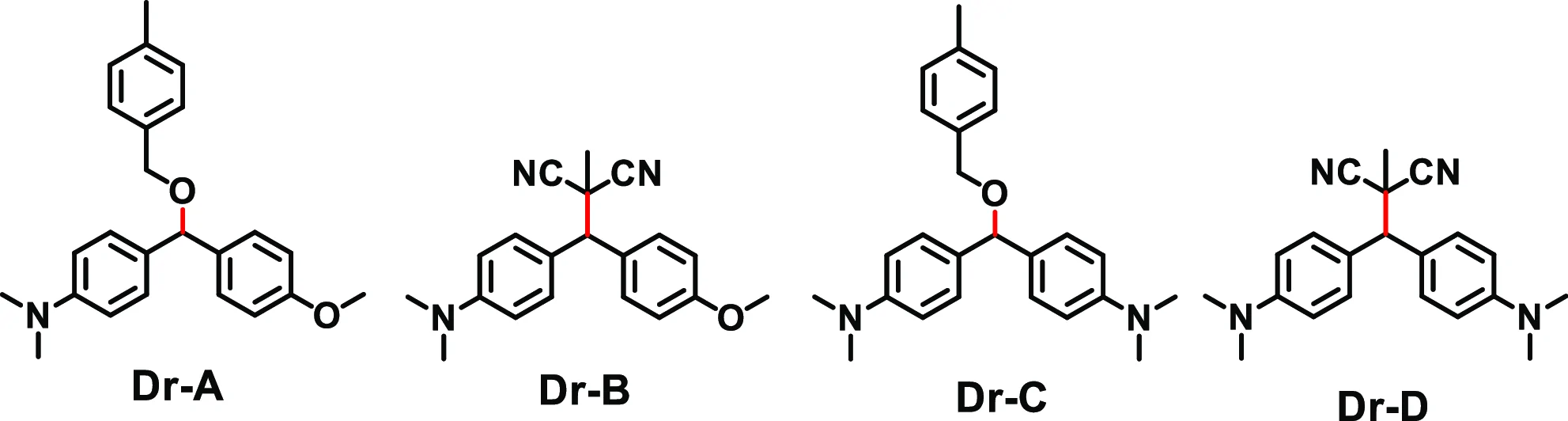
Model compounds used for DFT calculation. The cleaved bond is highlighted in red.

We used the polarizable continuum model (PCM) to model the environmental influence on the bond cleavage mechanism, as it is known to be important for the selectivity of mechanochemical processes.^45–47^ Thus, the PCM model allowed us to explore the effects of the polymer matrices on the relative preference for homolytic or heterolytic cleavage. Geometry optimizations were conducted at the B3LYP-D3/6-31G­(d) level of theory with single-point energy calculations at B3LYP-D3/6-311+G­(d,p). The free energy of the C–O and C–C bond homolysis or heterolysis using PCM (Δ*G*_hom_ and Δ*G*_het_, respectively), their difference to unravel thermodynamic bias ΔΔ*G*_hom–het_ and the free energy of the C–O and C–C bond heterolysis in the presence of explicit MeOH molecules (Δ*G*_het_·MeOH) were calculated at 298 K and 1 M.

Three different models were used, spanning from a purely polar medium (ethanol, ε = 24.9) to a low-polarity system modeled with poly­(methyl methacrylate) (PMMA) (ε = 3.9).^48^ This enables the determination of both main influences, i.e., the mechanophore and the environment, that determine the selectivity of the cleavage.

Since PCM is an implicit solvation method, we performed additional calculations including explicit methanol (MeOH) molecules as model hydrogen-bond donors with mechanophores **Dr-A** and **Dr-C**.^40^ These calculations model the effects of the HEA-comonomer, containing free alcohol groups in the polymer matrix. The energy difference between the two radical products and their ionic analogs then allowed the determination of the cleavage selectivity (Table 1). The absolute values are quite large, but it must be taken into account that the cleavage takes place under mechanical stress and the energies are referenced to the unstrained molecular fragments.

**Table 1:** Calculated Free Energy (kcal/mol) of C–O or C–C Bond Cleavage in **Dr-A**–**D**

solvent	Δ*G*_hom_	Δ*G*_het_	Δ*G*_het·MeOH_	ΔΔ*G*_hom–het_	ΔG_hom_	ΔG_het_	ΔΔG_hom–het_
	Dr-A				Dr-B		
EtOH	42.7	32.8	26.7	+9.8	28.2	14.8	+13.4
EtCH_2_CO_2_Me	43.4	47.3	(39.7)^a^	–3.8	28.7	26.6	+2.1
PMMA	43.9	54.9	(46.6)^a^	–11.0	28.9	32.9	–4.0
	**Dr-C**				**Dr-D**		
EtOH	42.9	27.8	21.7	+15.1	28.0	9.3	+18.7
EtCH_2_CO_2_Me	43.5	41.2	(33.6)^a^	+2.4	28.5	20.2	+8.3
PMMA	43.6	48.4	(40.0)^a^	–4.7	28.6	26.3	+2.3

a At low polarity, no hydrogen-bond moieties are expected to be available in the polymer.

Our calculations revealed that **Dr-A**, the model compound of **Dr-1**, exhibits the strongest bias toward homolytic C–O cleavage in apolar media (PMMA), with Δ*G*_hom_ 11.0 kcal/mol below Δ*G*_het_ (Table 1). Even under hydrogen-bond conditions—which should mimic the presence of the HEA comonomer in the polymer matrix—heterolysis remains thermodynamically disfavored (Table 1). This profile is consistent with the experimentally observed exclusive homolytic dissociation of **Dr-1**.

Model **Dr-C**, mimicking **Dr-3**, displayed conditional heterolysis instead. The calculated free energies for the homolytic and heterolytic pathways are closer in the apolar environment, with a slight preference for homolysis (Table 1). This preference is inverted by the explicit presence of MeOH, which significantly lowers the free energy of the process and renders carbocation formation thermodynamically favorable (Table 1). This trend matches the experimental observations, with **Dr-3** activating only in P­(MA-*co*-HEA) in the form of deep-blue carbocations that lack an EPR signal (cf. Figure 4f–i). Thus, these results confirm that stronger electron-donating substituents render heterolysis accessible, provided that a polar matrix or hydrogen-bond donors are accessible in the solid-state medium.

Finally, the computational investigation on **Dr-B** and **Dr-D** (model compounds for **Dr-2** and **Dr-4**, respectively) highlighted that introducing a malononitrile fragment dramatically alters the thermodynamic landscape (Table 1). **Dr-B** displays heterolytic C–C cleavage as the preferred pathway in polar media and near-isoenergetic competition in apolar environments. This reduced penalty for charge separation accounts for the matrix-independent heterolysis observed experimentally for **Dr-2**, including the rate-dependent, transient red mechanochromism that reflects moderate carbocation stabilization and rapid relaxation (cf. Figure 5a–e). **Dr-D** exacerbates the preference for heterolysis even further: it is thermodynamically favored across all environments, including apolar matrices, due to the simultaneous stabilization of the diarylmethane carbocation and the malononitrile carbanion (Table 1). Such a large driving force explains the earlier onset of activation (∼80% strain), the intense blue mechanochromism with near-complete loss of transmission at high strain, and the absence of radicals in EPR (cf. Figure 5f–j). The higher molar absorptivity of the **Dr-4**-derived carbocation relative to that derived from **Dr-2** further amplifies the optical contrast between mechanically activated polymer films incorporating these two mechanophores.

Overall, the DFT calculations provide a thermodynamic map that mirrors the experimental landscape and rationalizes how substituent electronics, matrix polarity, and leaving-group stabilization collectively govern pathway selection under force. Our computational study confirms the conclusions that (i) the electronic effect of the substituents tunes the stability of the carbocations, (ii) the bias between the homolytic and heterolytic pathways is gated by matrix polarity and presence of hydrogen-bond donors, and (iii) leaving-groups that can effectively stabilize a negative charge significantly reduce the influence of matrix effects. However, we note that the experimental techniques employed in this work cannot completely exclude the transient formation of minor populations of alternative reactive intermediates below the detection limit.

While thermodynamic preferences provide useful insight into product stability, mechanochemical processes are often kinetically controlled.^35,49–51^ To further assess the effect of force on bond dissociation, additional “external force explicitly included” (EFEI)^52^ calculations were performed on mechanophores **Dr-A**–**Dr-D** in ORCA 6.1 ^53^ under constant external applied force (Page S100). These calculations show that force substantially lowers the dissociation barriers and support the accessibility of the experimentally observed bond-cleavage events. No alternative competing pathways were identified on the force-modified potential energy surface. Thus, the calculated results support the experimentally observed reactivity trends and remain consistent with the structure–reactivity relationships discussed above. In other words, the computed thermodynamics provide chemical insight into the observed mechanochemical behavior of the system. Future work will incorporate force-dependent activation barriers to further refine these insights.^52–54^

## Conclusions

In this work, we have established how molecular structure and polymer environment collectively determine whether mechanical force leads to radical or ionic bond scission in polymer solids. By systematically varying aromatic substituent electronics, leaving-group stabilization, and the hydrogen-bonding capability of the polymer matrix, we identify the key factors governing the mechanochemical reactivity of diarylmethane mechanophores in the solid state.

To the best of our knowledge, the diarylmethane platform introduced here represents the first family of mechanophores based on classical CH-centered diphenylmethane architectures that can undergo both homolytic and heterolytic bond scission under tensile load. Weakly electron-donating substituents embedded in hydrogen-bond-poor matrices favor radical formation, whereas strongly electron-donating substituents combined with hydrogen-bond-donating environments promote heterolysis through stabilization of diarylcarbenium ions. In contrast, malononitrile-based leaving groups enable matrix-independent heterolytic activation by stabilizing the charge-separated state.

This work highlights how concepts from solution-phase physical organic chemistry (e.g., p*K*_a_, p*K*_R_+, radical stability) can be translated to solid-state polymer systems under force, providing a framework for the rational design of mechanophores with distinct and well-defined reactivity. Although the specific structure–reactivity relationships identified here were derived from diarylmethane mechanophores, the broader concepts of carbocation stabilization, leaving-group effects, and environmental stabilization may prove useful in the design of other force-responsive molecular systems. Establishing whether similar relationships emerge across mechanophore families with distinct electronic structures and force-coupling motifs represents an important direction for future research.

## Supplementary Material











## Data Availability

The computational data underlying this study have been made openly accessible on ioChem-BD at 10.19061/iochem-bd-1-342.^54^

## Notes

Experimental procedures for the synthesis of **Dr-1**–**4** and **Dr-I**-**IV**, characterization data for all newly synthesized compounds, including ^1^H and ^13^C NMR, FT-IR spectra, HR-MS, and UV–Vis spectra. Additional experimental details regarding the preparation and characterization of polymer networks, including swelling index measurements, mechanical and thermal analyses (tensile testing, TGA, and DSC), and electron paramagnetic resonance (EPR) spectroscopy are also provided. Computational details, calculated structures, and atomic coordinates of the calculated structures are included. (PDF)
Mechanochemical activation upon manual deformation of **PMA-Dr-1** Supplementary Movie 1 (mp4) (MP4)
Mechanochemical activation upon manual deformation of **P­(MA-** *co* **-HEA)-Dr-3** Supplementary Movie 2 (mp4) (MP4)
Mechanochemical activation upon manual deformation of **PMA-Dr-2** Supplementary Movie 3 (mp4) (MOV)
Mechanochemical activation upon manual deformation of **PMA-Dr-4** Supplementary Movie 4 (mp4) (MOV)

## References

[1] Davis D. A., Hamilton A., Yang J., Cremar L. D., Van Gough D., Potisek S. L., Ong M. T., Braun P. V., Martínez T. J., White S. R., Moore J. S., Sottos N. R. (2009). Force-Induced Activation
of Covalent Bonds in Mechanoresponsive Polymeric Materials. Nature.

[2] May P. A., Moore J. S. (2013). Polymer Mechanochemistry: Techniques to Generate Molecular
Force via Elongational Flows. Chem. Soc. Rev..

[3] Staudinger H., Bondy H. F. (1930). Über Isopren
Und Kautschuk, 19. Mitteil.: Über
Die Molekülgröße Des Kautschuks Und Der Balata. Ber. Dtsch. Chem. Ges..

[4] Staudinger H., Heuer W. (1934). Über Hochpolymere Verbindungen,
93. Mitteil.: Über
Das Zerreißen Der Faden-Moleküle Des Poly-Styrols. Ber. Dtsch. Chem. Ges..

[5] Kauzman W., Eyring H. (1940). The Viscous Flow of Large Molecules. J. Am. Chem. Soc..

[6] De
Bo G. (2020). Polymer Mechanochemistry and the Emergence of the Mechanophore Concept. Macromolecules.

[7] Deneke N., Rencheck M. L., Davis C. S. (2020). An Engineer’s
Introduction
to Mechanophores. Soft Matter.

[8] Kida J., Imato K., Goseki R., Aoki D., Morimoto M., Otsuka H. (2018). The Photoregulation
of a Mechanochemical Polymer Scission. Nat.
Commun..

[9] Li J., Nagamani C., Moore J. S. (2015). Polymer
Mechanochemistry: From Destructive
to Productive. Acc. Chem. Res..

[10] Lin Y., Kouznetsova T. B., Craig S. L. (2020). A Latent Mechanoacid for Time-Stamped
Mechanochromism and Chemical Signaling in Polymeric Materials. J. Am. Chem. Soc..

[11] Xu D., Cheng X., Liu W., Sun Y., Niu Y., Wang M., Qian H. (2026). Mechanochromic Mechanophores. Chem. Rev..

[12] Diesendruck C. E., Sottos N. R., Moore J. S., White S. R. (2015). Biomimetic
Self-Healing. Angew. Chem., Int. Ed..

[13] Ghanem M. A., Basu A., Behrou R., Boechler N., Boydston A. J., Craig S. L., Lin Y., Lynde B. E., Nelson A., Shen H., Storti D. W. (2021). The Role of Polymer
Mechanochemistry
in Responsive Materials and Additive Manufacturing. Nat. Rev. Mater..

[14] Zhang M., Choi W., Kim M., Choi J., Zang X., Ren Y., Chen H., Tsukruk V., Peng J., Liu Y., Kim D. H., Lin Z. (2024). Recent Advances
in Environmentally
Friendly Dual-Crosslinking Polymer Networks. Angew. Chem., Int. Ed..

[15] Shen H., Larsen M. B., Roessler A. G., Zimmerman P. M., Boydston A. J. (2021). Mechanochemical Release of *N*-Heterocyclic
Carbenes from Flex-Activated Mechanophores. Angew. Chem., Int. Ed..

[16] Anwer A. H., Khan N., Ansari M. Z., Baek S.-S., Yi H., Kim S., Noh S. M., Jeong C. (2022). Recent Advances in Touch Sensors
for Flexible Wearable Devices. Sensors.

[17] Costentin C., Robert M., Savéant J. M. (2003). Activation
Barriers in the Homolytic
Cleavage of Radicals and Ion Radicals. J. Am.
Chem. Soc..

[18] Morcillo S. P. (2019). Radical-Promoted
C–C Bond Cleavage: A Deconstructive Approach for Selective
Functionalization. Angew. Chem..

[19] Chen Y., Mellot G., Van Luijk D., Creton C., Sijbesma R. P. (2021). Mechanochemical
Tools for Polymer Materials. Chem. Soc. Rev..

[20] Huang Y., Huang S., Li Q. (2023). Mechanochromic
Polymers Based on
Mechanophores. ChemPlusChem.

[21] Streidl N., Denegri B., Kronja O., Mayr H. (2010). A Practical
Guide for
Estimating Rates of Heterolysis Reactions. Acc.
Chem. Res..

[22] Wang C., Sun C. L., Boulatov R. (2024). Productive
Chemistry Induced by Mechanochemically
Generated Macroradicals. Chem. Commun..

[23] Leifert D., Studer A. (2020). The Persistent Radical
Effect in Organic Synthesis. Angew. Chem., Int.
Ed..

[24] Zhang Z., Zhang J., Zhi Sun J., Zhang H., Zhang X., Zhong Tang B. (2025). Luminescent
Radical Polymers. Chem. Eur. J..

[25] Imato K., Irie A., Kosuge T., Ohishi T., Nishihara M., Takahara A., Otsuka H. (2015). Mechanophores with
a Reversible Radical
System and Freezing-Induced Mechanochemistry in Polymer Solutions
and Gels. Angew. Chem..

[26] Sumi T., Goseki R., Otsuka H. (2017). Tetraarylsuccinonitriles
as Mechanochromophores
to Generate Highly Stable Luminescent Carbon-Centered Radicals. Chem. Commun..

[27] Sakai H., Sumi T., Aoki D., Goseki R., Otsuka H. (2018). Thermally
Stable Radical-Type Mechanochromic Polymers Based on Difluorenylsuccinonitrile. ACS Macro Lett..

[28] Verstraeten F., Göstl R., Sijbesma R. P. (2016). Stress-Induced Colouration
and Crosslinking
of Polymeric Materials by Mechanochemical Formation of Triphenylimidazolyl
Radicals. Chem. Commun..

[29] Karman M., Verde-Sesto E., Weder C., Simon Y. C. (2018). Mechanochemical
Fluorescence Switching in Polymers Containing Dithiomaleimide Moieties. ACS Macro Lett..

[30] Lenhardt J. M., Ong M. T., Choe R., Evenhuis C. R., Martinez T. J., Craig S. L. (2010). Trapping a Diradical Transition State
by Mechanochemical
Polymer Extension. Science.

[31] Nixon R., De Bo G. (2021). Isotope Effect in the Activation
of a Mechanophore. J. Am. Chem. Soc..

[32] Lin Y., Kouznetsova T. B., Foret A. G., Craig S. L. (2024). Solvent Polarity
Effects on the Mechanochemistry of Spiropyran Ring Opening. J. Am. Chem. Soc..

[33] Aktah D., Frank I. (2002). Breaking Bonds by Mechanical Stress: When Do Electrons Decide for
the Other Side?. J. Am. Chem. Soc..

[34] Mache D. A., García-Padilla E., Frateloreto F., Egizzo G., Hemmer J. R., Valverde-Bastidas J. G., De la Flor S., Maseras F., Berrocal J. A. (2026). Controlling Solid-State
Mechanochemical Pathways in Triarylmethane Mechanophores via Molecular
and Matrix Design. Macromolecules.

[35] Nixon R., De Bo G. (2020). Three Concomitant C–C
Dissociation Pathways during the Mechanical
Activation of an *N*-Heterocyclic Carbene Precursor. Nat. Chem..

[36] Hemmer J. R., Bauernfeind V., Rader C., Petroselli M., Weder C., Berrocal J. A. (2023). Triarylmethane Mechanophores Enable
Full-Visible Spectrum Mechanochromism. Macromolecules.

[37] Hemmer J. R., Rader C., Wilts B. D., Weder C., Berrocal J. A. (2021). Heterolytic
Bond Cleavage in a Scissile Triarylmethane Mechanophore. J. Am. Chem. Soc..

[38] Mayr H., Bug T., Gotta M. F., Hering N., Irrgang B., Janker B., Kempf B., Loos R., Ofial A. R., Remennikov G., Schimmel H. (2001). Reference Scales for the Characterization of Cationic
Electrophiles and Neutral Nucleophiles. J. Am.
Chem. Soc..

[39] Mayr H. (2015). Reactivity
Scales for Quantifying Polar Organic Reactivity: The Benzhydrylium
Methodology. Tetrahedron.

[40] Mayr H., Ammer J., Baidya M., Maji B., Nigst T. A., Ofial A. R., Singer T. (2015). Scales of
Lewis Basicities toward
C-Centered Lewis Acids (Carbocations). J. Am.
Chem. Soc..

[41] Mayer R. J., Hampel N., Mayer P., Ofial A. R., Mayr H. (2019). Synthesis,
Structure, and Properties of Amino-Substituted Benzhydrylium Ions
– A Link between Ordinary Carbocations and Neutral Electrophiles. Eur. J. Org. Chem..

[42] Wesseler J. P., Hemmer J. R., Weder C., Berrocal J. A. (2025). Solid-State Mechanochemical
Activation of Anthracene-Maleimide Adducts: The Influence of the Polymer
Matrix. RSC Mechanochem..

[43] Yamamoto T., Kato S., Aoki D., Otsuka H. (2021). Diarylacetonitrile
as a Molecular Probe for the Detection of Polymeric Mechanoradicals
in the Bulk State through a Radical Chain-Transfer Mechanism. Angew. Chem., Int. Ed..

[44] Xu D., Huang R., He Y., Wang M., Wang Y. (2026). Mechanogated
PINO Radical Organocatalysis: Catalytic Amplification of Mechanical
Force. ACS Macro Lett..

[45] Pladevall B. S., de Aguirre A., Maseras F. (2021). Understanding Ball Milling Mechanochemical
Processes with DFT Calculations and Microkinetic Modeling. ChemSusChem.

[46] Galeas D. M., Tolbatov I., Colacino E., Maseras F. (2024). Computational Study
on the Mechanism for the Synthesis of Active Pharmaceutical Ingredients
Nitrofurantoin and Dantrolene in Both Solution and Mechanochemical
Conditions. Phys. Chem. Chem. Phys..

[47] Garcia-Padilla E., Maseras F. (2025). Understanding the Divergence
in Outcomes between Mechanochemical
and Solution Phase Reactivity of Fullerenes. Cell Rep. Phys. Sci..

[48] Nikolov I. D., Ivanov C. D. (2000). Optical Plastic
Refractive Measurements in the Visible
and the Near-Infrared Regions. Appl. Opt..

[49] Beedle A. E. M., Mora M., Davis C. T., Snijders A. P., Stirnemann G., Garcia-Manyes S. (2018). Forcing the
Reversibility of a Mechanochemical Reaction. Nat. Commun..

[50] Wang J., Kouznetsova T. B., Boulatov R., Craig S. L. (2016). Mechanical Gating
of a Mechanochemical Reaction Cascade. Nat.
Commun..

[51] Sun Y., Kevlishvili I., Kouznetsova T. B., Burke Z. P., Craig S. L., Kulik H. J., Moore J. S. (2024). The Tension-Activated Carbon–Carbon
Bond. Chem.

[52] Ribas-Arino J., Shiga M., Marx D. (2009). Understanding Covalent
Mechanochemistry. Angew. Chem., Int. Ed..

[53] Neese F. (2025). Software Update:
The ORCA Program System—Version 6.0. WIREs Comput. Mol. Sci..

[54] Álvarez-Moreno M., De Graaf C., López N., Maseras F., Poblet J. M., Bo C. (2015). Managing the
Computational Chemistry Big Data Problem: The IoChem-BD
Platform. J. Chem. Inf. Model..

