# Supplementary material for: Designing Heterolytic and Homolytic Bond Scission in Diarylmethane Mechanophores
Source: JACS Au. 2026 Aug 17;6(9):5307–16. doi: 10.1021/jacsau.6c00989 (PMC13625560; doi:10.1021/jacsau.6c00989)
Supplement: Supplementary file 1 [file au6c00989_si_001.pdf]

Supporting Information for

Designing Heterolytic and Homolytic Bond Scission in Diarylmethane Mechanophores

Gaia Egizzo,[a,b] Eduardo García-Padilla,[a] Federico Frateloreto,[a] Florent Monie,[a] Dnyaneshwar Ajinath Mache,[a,b] Feliu Maseras,[a] and José Augusto Berrocal[a,c]*

[a] Institute of Chemical Research of Catalonia (ICIQ), The Barcelona Institute of Science and Technology (BIST), Av. Països Catalans 16, 43007 Tarragona (Spain)

[b] Dept. Química Analítica i Química Orgànica, Universitat Rovira i Virgili (URV), 43007 Tarragona, Spain

[c] Catalan Institution for Research and Advanced Studies (ICREA), Pg. Lluís Companys 23, 08010 Barcelona (Spain)

*to whom correspondence should be addressed: jberrocal@iciq.es

Contents

Experimental Section 3

Synthesis of **Dr-1** and **Dr-2** 6

Synthesis of **Dr-3** and **Dr-4** 7

Synthesis of **Dr-I**, **Dr-II**, **Dr-III**, and **Dr-IV** 8

(4-((2-hydroxyethyl)(methyl)amino)phenyl)(4-methoxyphenyl)methanone (**2**) 9

(4-methoxyphenyl)(4-(methyl(2-((4vinylbenzyl)oxy)ethyl)amino)phenyl)methanone (**3**) 12

(4-methoxyphenyl)(4-(methyl(2-((4-vinylbenzyl)oxy)ethyl)amino)phenyl)methanol (**4**) 15

bis(4-((2-hydroxyethyl)(methyl)amino)phenyl)methanone (**7**) 18

(4-((2-hydroxyethyl)(methyl)amino)phenyl)(4-(methyl(2-((4-vinylbenzyl)oxy)ethyl)amino)phenyl)methanone (**8**) 21

(4-((2-methoxyethyl)(methyl)amino)phenyl)(4-(methyl(2-((4-vinylbenzyl)oxy)ethyl)amino)phenyl)methanone (**9**) 24

(4-((2-methoxyethyl)(methyl)amino)phenyl)(4-(methyl(2-((4-vinylbenzyl)oxy)ethyl)amino)phenyl)methanol (**10**) 27

4-((4-methoxyphenyl)((4-vinylbenzyl)oxy)methyl)-N-methyl-N-(2-((4-vinylbenzyl)oxy)ethyl)aniline (**Dr-1**) 30

2-(4-vinylbenzyl)malononitrile (**5**) 34

2-((4-methoxyphenyl)(4-(methyl(2-((4-vinylbenzyl)oxy)ethyl)amino)phenyl)methyl)-2-(4-vinylbenzyl)malononitrile (**Dr-2**) 37

N-(2-methoxyethyl)-N-methyl-4-((4-(methyl(2-((4-vinylbenzyl)oxy)ethyl)amino)phenyl)((4-vinylbenzyl)oxy)methyl)aniline (**Dr-3**) 40

2-((4-((2-methoxyethyl)(methyl)amino)phenyl)(4-(methyl(2-((4-vinylbenzyl)oxy)ethyl)amino)phenyl)methyl)-2-(4-vinylbenzyl)malononitrile (**Dr-4**) 43

4-((benzyloxy)(4-methoxyphenyl)methyl)-N-methyl-N-(2-((4-vinylbenzyl)oxy)ethyl)aniline (**Dr-I**) 46

2-benzyl malononitrile (**12**) 49

2-benzyl-2-((4-methoxyphenyl)(4-(methyl(2-((4-vinylbenzyl)oxy)ethyl)amino)phenyl)methyl)malononitrile (**Dr-II**) 52

4-((benzyloxy)(4-((2-methoxyethyl)(methyl)amino)phenyl)methyl)-N-methyl-N-(2-((4-vinylbenzyl)oxy)ethyl)aniline (**Dr-III**) 55

2-benzyl-2-((4-((2-methoxyethyl)(methyl)amino)phenyl)(4-(methyl(2-((4-vinylbenzyl)oxy)ethyl)amino)phenyl)methyl)malononitrile (**Dr-IV**) 58

1,4-bis((4-vinylbenzyl)oxy)butane (**BVBB**) 61

^{1}H NMR spectra and UV-Vis spectra of carbocations in solution 64

Synthesis of polymer networks 67

Swelling index determination 70

Mechanoresponsive behavior of PMA and P(MA-*co*-HEA) networks under tensile deformation 72

Mechanical and thermal characterization of PMA and P(MA-*co*-HEA) networks 76

Tensile test 76

Thermogravimetric analysis (TGA) and Differential Scanning Calorimetry (DSC) 83

Electron Paramagnetic Resonance spectroscopy (EPR) 93

Computational results 99

Bibliography 138

Atomic coordinates of the calculated structures**Error! Bookmark not defined.**

Experimental Section

Materials. All reagents were used as purchased with no further purification. Anhydrous solvents were dried by passing through an activated alumina column on a PureSolvTM solvent purification system (Innovative Technologies, Inc., MA). Analytical thin layer chromatography (TLC) was carried out using aluminum sheets with 0.2 mm of silica gel, using UV light or treatment with a solution of potassium permanganate or iodine stain for revealing. Flash column chromatography was performed on silica gel (Aldrich, 230-400 mesh) or with Biotage Selekt flash chromatography apparatus using packed silica column Biotage® Sfär Silica - 60 µm with 10 g, 25g, 50g, or 100g of stationary phase. All reactions requiring anhydrous conditions were carried out using dried Schlenk glassware and under an inert Ar gas atmosphere. Yields refer to purified compounds unless otherwise noted.

Nuclear magnetic resonance (NMR) spectroscopy. NMR spectra were recorded at 298 K on a Bruker Avance 300 or Bruker Avance 400 Ultrashield apparatus. Data were evaluated with the MestReNova software suite (v 12.0), and all chemical shifts (δ) are reported in parts per million (ppm) relative to the residual solvent signals of the deuterated solvent used. Coupling constants (*J*) are reported in hertz (Hz). Multiplicity is reported with the following abbreviations: s = singlet, brs = broad singlet, d = doublet, t = triplet, q = quartet, dt = doublet of triplets, td = triplet of doublets, tt = triplet of triplets, sp = septet, m = multiplet.

Fourier Transformed Infrared (FT-IR). Infrared spectra were recorded on a Bruker Tensor 27.

Mass spectrometry (MS). Mass spectra were recorded on a HPLC-MS-TOF Waters LCT Premier spectrometer equipped with an ESI source.

UV-Vis spectroscopy. UV-vis spectra were acquired on a Shimadzu Corporation UV-1800PC spectrophotometer using a quartz cuvette (10 mm path length, 3.5 mL volume).

Fluorescence spectroscopy. Fluorescence spectra were acquired on a Fluorolog Horiba Jobin Yvon instrument using a quartz cuvette (10 mm path length, 3.5 mL volume).

Electron paramagnetic resonance spectroscopy (EPR). EPR experiments were conducted on an EMX Micro spectrometer equipped with an X-band bridge (9.1–9.9 GHz). Spectra were acquired from solid polymer films with a thickness of <0.5 mm and a length of 2.5 cm, under ambient conditions (298 K) and argon atmosphere. Spectra were recorded before and after stretching the samples, as well as from an additional sample irradiated in situ inside the microwave cavity with UV light (mercury lamp) to generate paramagnetic species. Specifications of the irradiation experiments using the mercury lamp: λ = 200-2000 nm, power intensity of 100W, irradiation time of 1 hour.

Thermogravimetric analyses (TGA) were performed with a Mettler-Toledo TGA/DSC 1 STARe System. The temperature ranged from 30 °C to 600 °C with a heating rate of 10 °C min^{-1}. Tests were carried out under nitrogen with a flow rate of 40 mL min^{-1}.

Differential scanning calorimetry (DSC) was conducted under nitrogen using a MettlerToledo STARe system (DSC822e) differential scanning calorimeter at a temperature range from -80 °C to 200 °C with a heating and cooling rate of 10 °C min^{-1}.

Tensile testing. Mechanical tests with in situ fluorescence and transmittance measurements were carried out using a Linkam TST350 microtensile stage equipped with a 200 N load cell and controlled by the accompanying Linksys32 software. The temperature was set to 25 °C. Data were collected using rectangular samples with dimensions of 40 × 5.0 × 0.25 mm (length × width × thickness) at a strain rate of 2.2% s^{-1} (0.375 µm s^{-1}), if not differently stated (e.g., **PMA-Dr-2**).

For in situ fluorescence measurements, an optical fiber (QR400-7-UV-BX, 400 µm core; Ocean Insight, Orlando, FL, USA) collected light emitted from the sample while irradiating with an LED source (365 nm, 12 % power, LDC-1C, Ocean Insights, Orlando, FL, USA).

For in situ transmittance measurements, an optical microscope (Axioscope 5, Carl Zeiss Microscopy GmbH, Germany) was equipped with an LED source (nominal power 10W) that illuminated the sample while stretching. An optical fiber (P200-2-VIS-NIR, 200 µm core; Ocean Insight, Orlando, FL, USA) collected the light transmitted from the sample with a measurement spot size of approximately 200 µm. The emission and transmission spectra were analyzed using a spectrometer (Maya 2000-Pro, Ocean Insights, Orlando, FL, USA).

Photopolymerizations were performed using a Dr. Hönle LED Cube 100 at 365 nm. At maximum power (100%), the typical irradiance at the sample position is approximately 1000 mW/cm^2, as specified by the manufacturer. The polymerization was performed using a sequence of six 20-second bursts at 50% power (≈500 mW/cm^2) with 5 seconds pause between each burst, followed by ten 30-second bursts at 20% power (≈200 mW/cm^2) with 5 seconds pause between each burst.

Processing of optical and tensile stage data. Tensile data and both emission and transmission spectra were aligned on a common time axis. The tensile trace was truncated shortly after sample failure, identified from the second derivative of the calculated stress. The optical trace was truncated before the transmittance in the absorbance band exceeded 100%. Each emission spectrum was smoothed (window size 7) and plotted by normalizing the intensity of the peak of interest to that of a reference peak in the resting state. This produced a normalized intensity. The relative change in the emission was then calculated as the ratio between the intensities at the two wavelengths ($Q = I_{490} / I_{585}$).

Each transmittance spectrum was smoothed (window size 61). The minimum transmittance in the visible range was located and averaged over 10 points centered on the minimum. The

relative change in transmittance in the absorbance band was computed as the difference between this averaged minimum and the spectral baseline averaged over 720 nm. Finally, these relative transmittance changes were mapped to the corresponding strain values to enable comparison with the sample's stress evolution.

Radical stability experiments. Radical stability toward oxygen was evaluated using two polymer strips (25 × 1.0 × 0.25 mm; length × width × thickness). One strip was stretched under ambient air, whereas the other was degassed by repeated argon/vacuum cycles over 4 h and subsequently stretched under a continuous argon flow. Photographs were acquired under 365 nm UV irradiation at regular time intervals to monitor the evolution of the fluorescence. The degassed sample was kept under an argon atmosphere throughout the experiment, except for the few seconds required to acquire each photograph.

Computational details. All calculations were carried out using Gaussian09.[1] Geometry optimizations were performed with B3LYP[2-4] and Grimme's D3 dispersion correction[5], with the 6-31G(d) basis set being used.[6] Accurate energies were obtained from single-point calculations with B3LYP-D3 and 6-311+G(d,p). The solvation environment was modelled with the implicit polarizable continuum model (PCM) in all calculations,[7] using ethanol, methyl propanoate, methyl butanoate, or approximation of poly(methyl methacrylate)[8]. All stationary points were characterized by the absence of imaginary vibrational frequencies. The reported free energies were calculated at 298 K and 1 M.
Additional computational calculations were performed in ORCA 6.1[9] with the inclusion of applied force with the EFEI method on mechanophores **Dr-A-D**. The geometry optimizations were carried out at B3LYP-D3(BJ)/def2-SVP, with tight convergence criteria for SCF and optimization, followed by single-point energy calculations at B3LYP-D3(BJ)/def2-TZVPPD[10] with a finer integration grid DefGrid3. The RIJCOSX approximation[11] was used using the def2/J auxiliary basis sets. Implicit solvation with CPCM for PMMA was modeled with the same parameters as the other calculations.
The computational data underlying this study have been made open access on ioChem-BD at https://doi.org/10.19061/iochem-bd-1-342. [12]

Synthesis and Characterization

Synthesis of Dr-1 and Dr-2

a)

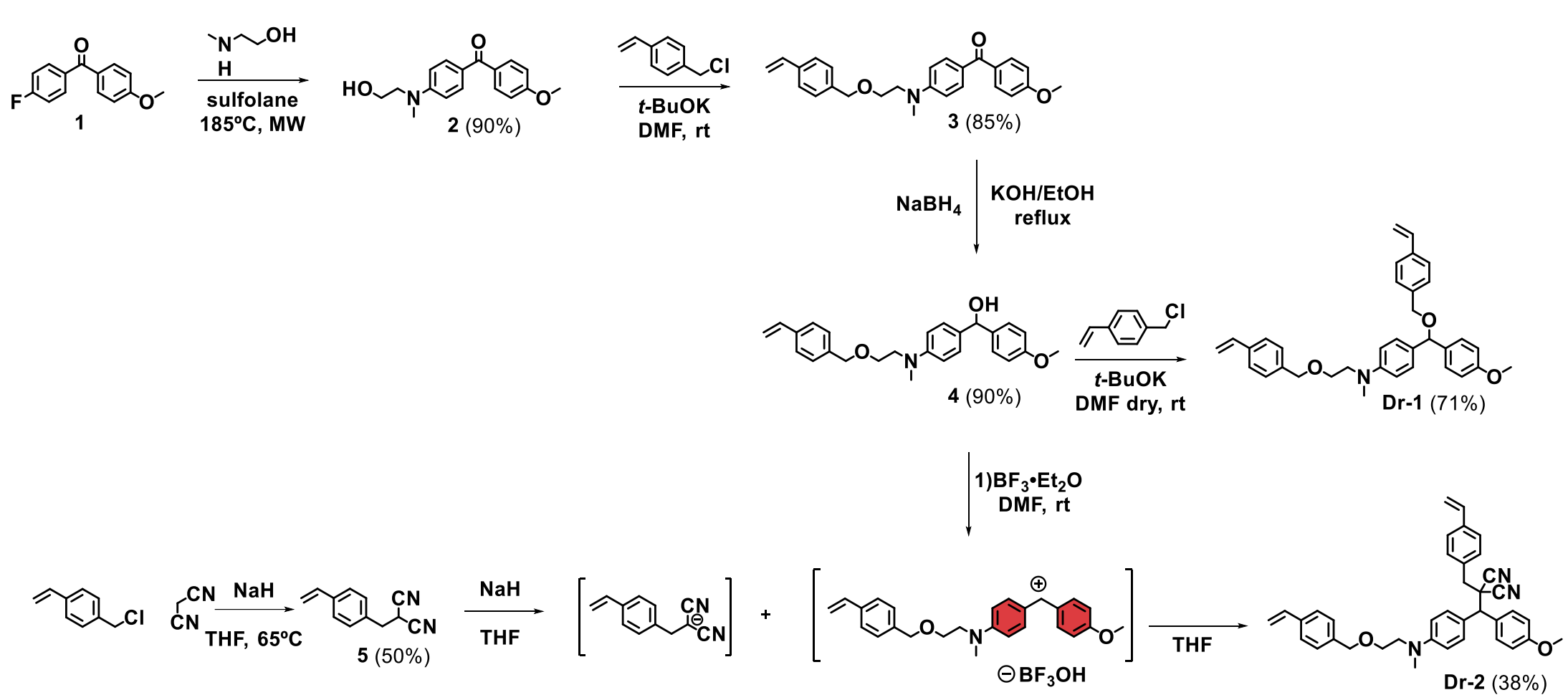

Scheme S1: Synthetic route to **Dr-1** and **Dr-2** mechanophores

Starting from *p*-substituted benzophenone **1**, a nucleophilic aromatic substitution with methyl ethanolamine was performed in sulfolane at 185 °C under microwave irradiation, yielding amino alcohol intermediate **2**. Then, the hydroxyl group of **2** was deprotonated with potassium *tert*-butoxide (*t*-BuOK) in DMF at room temperature, and the resulting deprotonated species was subsequently reacted with 4-vinylbenzyl chloride to give ketone **3**. Reduction of **3** with sodium borohydride afforded carbinol **4**. Alcohol **4** was deprotonated with potassium *tert*-butoxide in dry DMF and reacted with 4-vinylbenzyl chloride to yield **Dr-1**.

Dr-2 was prepared from **4** and malononitrile derivative **5**. The latter was synthesized by treating malononitrile with sodium hydride, followed by the addition of 4-vinylbenzyl chloride. Subsequently, **5** was deprotonated with sodium hydride under stirring. Once deprotonation was complete, compound **4** was added to the reaction mixture. Boron trifluoride diethyl etherate ($BF_3 \cdot OEt_2$, 46%) was added dropwise, which lead to the formation of **Dr-2**.

Synthesis of Dr-3 and Dr-4

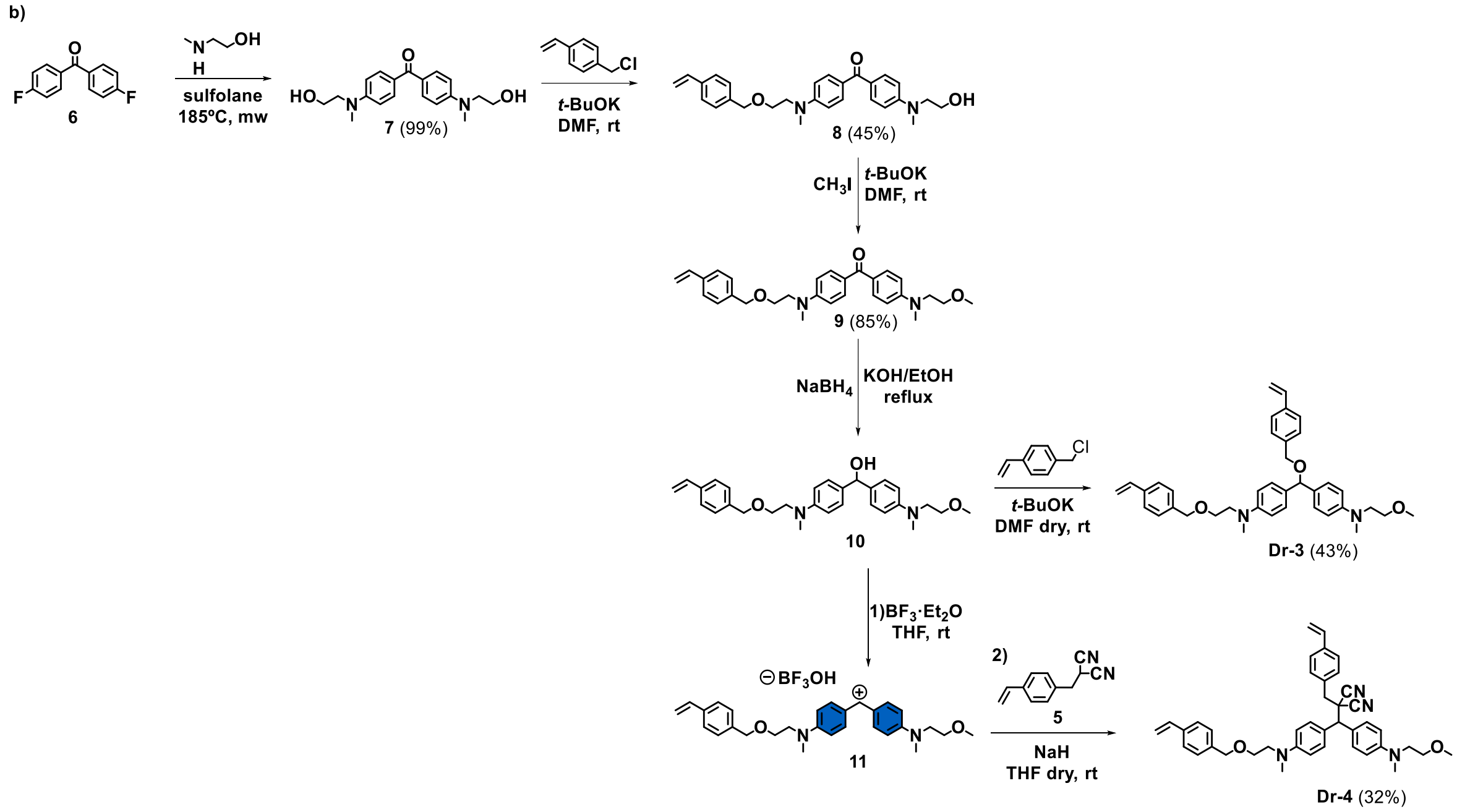

Scheme S2: Synthetic route to **Dr-3** and **Dr-4** mechanophores

Starting from *p*-substituted benzophenone **6**, a nucleophilic aromatic substitution with methyl ethanolamine was carried out in sulfolane at 185 °C under microwave conditions, affording amino alcohol intermediate **7**. The hydroxyl group of **7** was deprotonated with potassium *tert*-butoxide in DMF at room temperature, and the resulting deprotonated species was subsequently reacted with 4-vinylbenzyl chloride to yield ketone **8**. Next, the unreacted hydroxyl group of **8** was deprotonated with potassium *tert*-butoxide in dry DMF, followed by alkylation with methyl iodide to afford compound **9**. Reduction of **9** with sodium borohydride in refluxing ethanol gave carbinol **10**. Finally, alcohol **10** was deprotonated with potassium *tert*-butoxide in dry DMF and reacted with 4-vinylbenzyl chloride to afford the diarylmethane **Dr-3**.

Dr-4 was prepared from compounds **10** and **5**. Compound **10** was dissolved in dry THF and treated dropwise with boron trifluoride diethyl etherate ($BF_3{\cdot}OEt_2$, 46%) to promote the in situ formation of intermediate **11**. In parallel, compound **5** was deprotonated with sodium hydride to generate the corresponding carbanion, which was subsequently added to the reaction mixture containing **11**, affording **Dr-4**.

Synthesis of Dr-I, Dr-II, Dr-III and Dr-IV

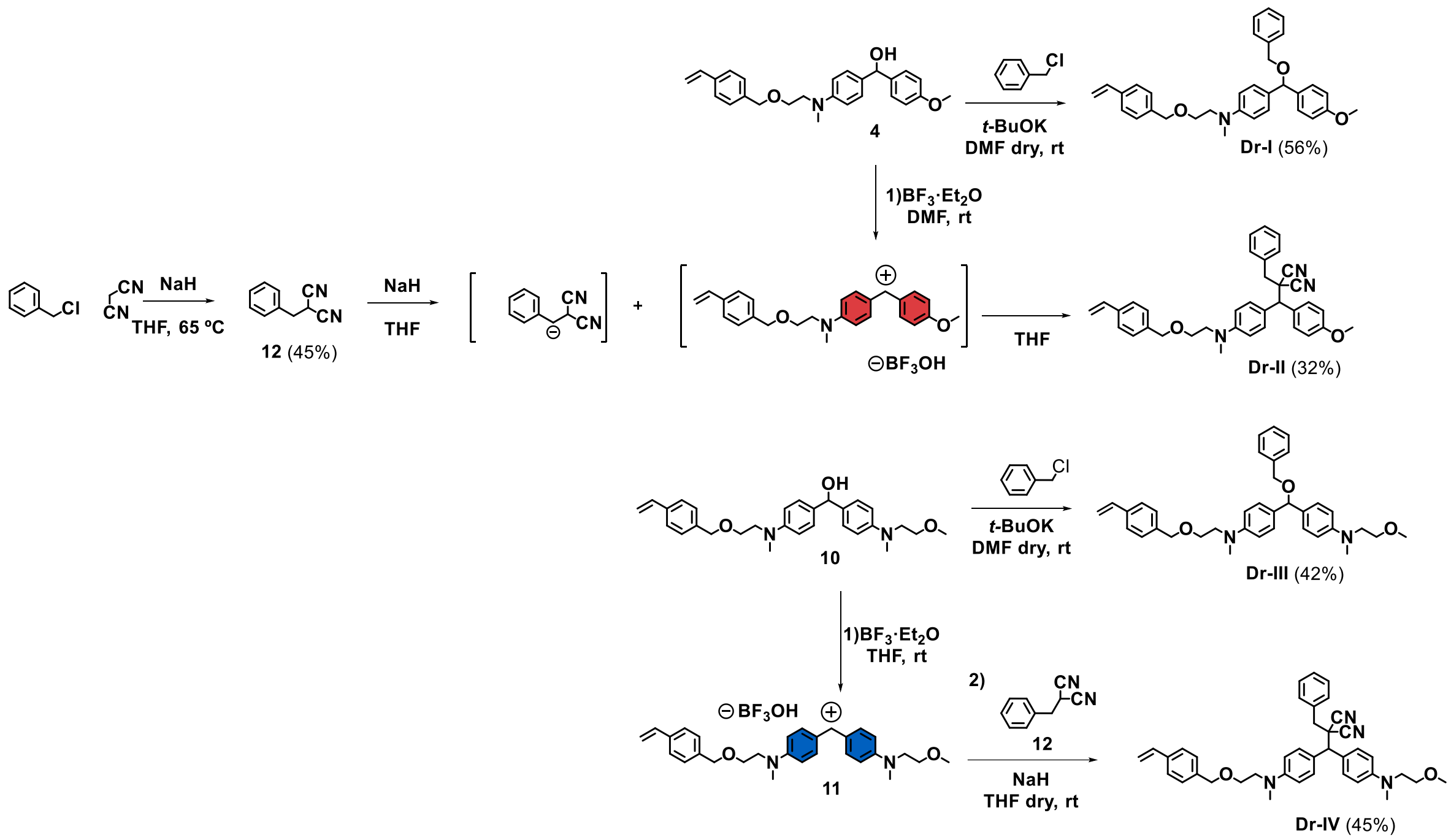

Scheme S3: Synthetic route to **Dr-I**–**IV** control molecules

Alcohol **4** was deprotonated using potassium *tert*-butoxide in dry DMF and subsequently reacted with benzyl chloride to yield the diarylmethane derivative **Dr-I**.

Compound **12** was synthesized by treating malononitrile with sodium hydride, followed by alkylation with benzyl chloride. Subsequently, **12** was deprotonated with sodium hydride under stirring. Once deprotonation was complete, compound **4** was added to the reaction mixture. Finally, boron trifluoride diethyl etherate ($BF_3 \cdot OEt_2$) was added dropwise to promote the formation of **Dr-II**.

Alcohol **10** was deprotonated with potassium *tert*-butoxide in dry DMF and reacted with benzyl chloride to obtain the fully functionalized compound **Dr-III**.

Compound **10** was dissolved in dry THF, and boron trifluoride diethyl etherate ($BF_3 \cdot OEt_2$) was added dropwise to promote the in situ formation of intermediate **11**. In a separate flask, compound **12** was deprotonated with sodium hydride to generate the corresponding carbanion, which was subsequently added to the reaction mixture containing **11**, leading to the formation of **Dr-IV**.

(4-((2-hydroxyethyl)(methyl)amino)phenyl)(4-methoxyphenyl)methanone (2)

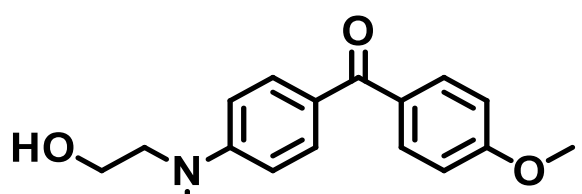

2

1 (2.0 g, 8.7×10^{-3} mol, 1 eq.) and *N*-methyl ethanolamine (1.3 g, 1.7×10^{-2} mol, 2 eq.) were combined in sulfolane (8 mL) in a 20 mL microwave vial. The vial was sealed and heated in a microwave at 185 °C for 4 hours under stirring. The mixture was poured into ice-cold water, resulting in the precipitation of a solid. The precipitate was collected by filtration, washed with water, and air-dried. The crude product was recrystallized from methanol, affording compound **2** as a pale yellow solid (2.2 g; yield: 90%).

^{1}H NMR (300 MHz, $CDCl_3$): δ/ppm 7.81–7.77 (m, 4H, Ar-H), 7.00–6.97 (m, 2H, Ar-H), 6.96–6.79 (m, 2H, Ar-H), 3.92–3.88 (m, 2H, CH_2–OH), 3.90 (s, 3H, OCH_3), 3.62 (t, *J* = 5.4 Hz, 2H, CH_2–N), 3.13 (s, 3H, NCH_3), 1.64 (brs, 1H, -OH).

^{13}C NMR (75 MHz, $CDCl_3$): δ/ppm 194.13, 162.41, 132.57, 131.96, 131.46, 113.34, 111.14, 60.12, 55.45, 54.78, 39.28.

FT-IR $(cm)^{-1}$: 3408, 2919, 1634, 1588, 1525, 1508, 1458, 1382, 1318, 1285, 1251, 1200, 1166, 1026, 926, 844, 767, 684.

HR-MS (m/z): $[M+H]^+$ calcd for $C_{17}H_{20}NO_3$ 286.1429; found 286.1438.

UV-Vis: Extinction coefficients (ε [$L\cdot mol^{-1}\cdot cm^{-1}$]) in the λ maxima ($\lambda_{1\text{-}2}$) of 3.2×10^{-5} M acetonitrile (MeCN) solution of **2**

λ_1, nm (ε): 273 (25396).

λ_2, nm (ε): 345 (38095).

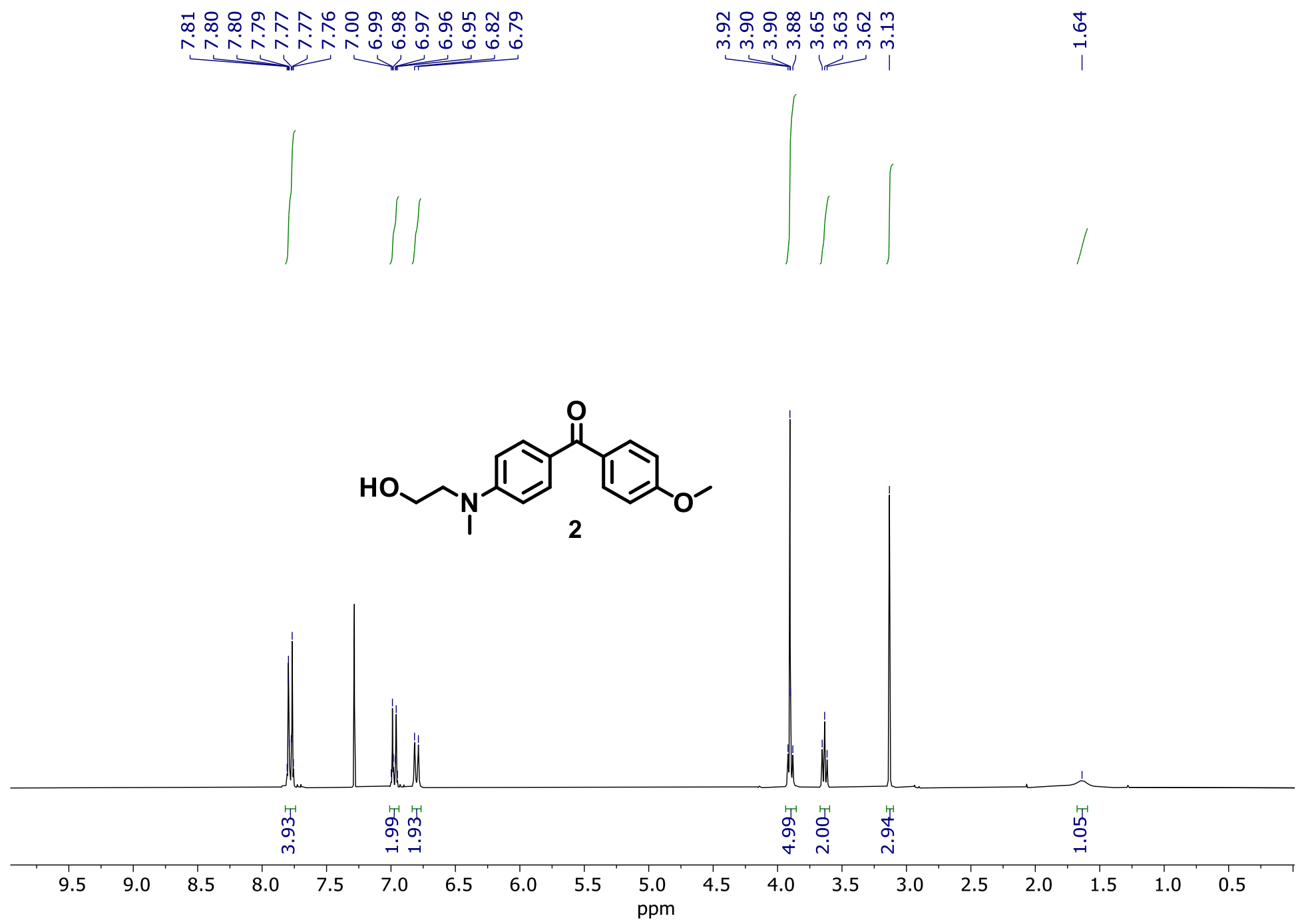

Figure S1: ^{1}H NMR spectrum of **2** (300 MHz, $CDCl_3$, 293 K).

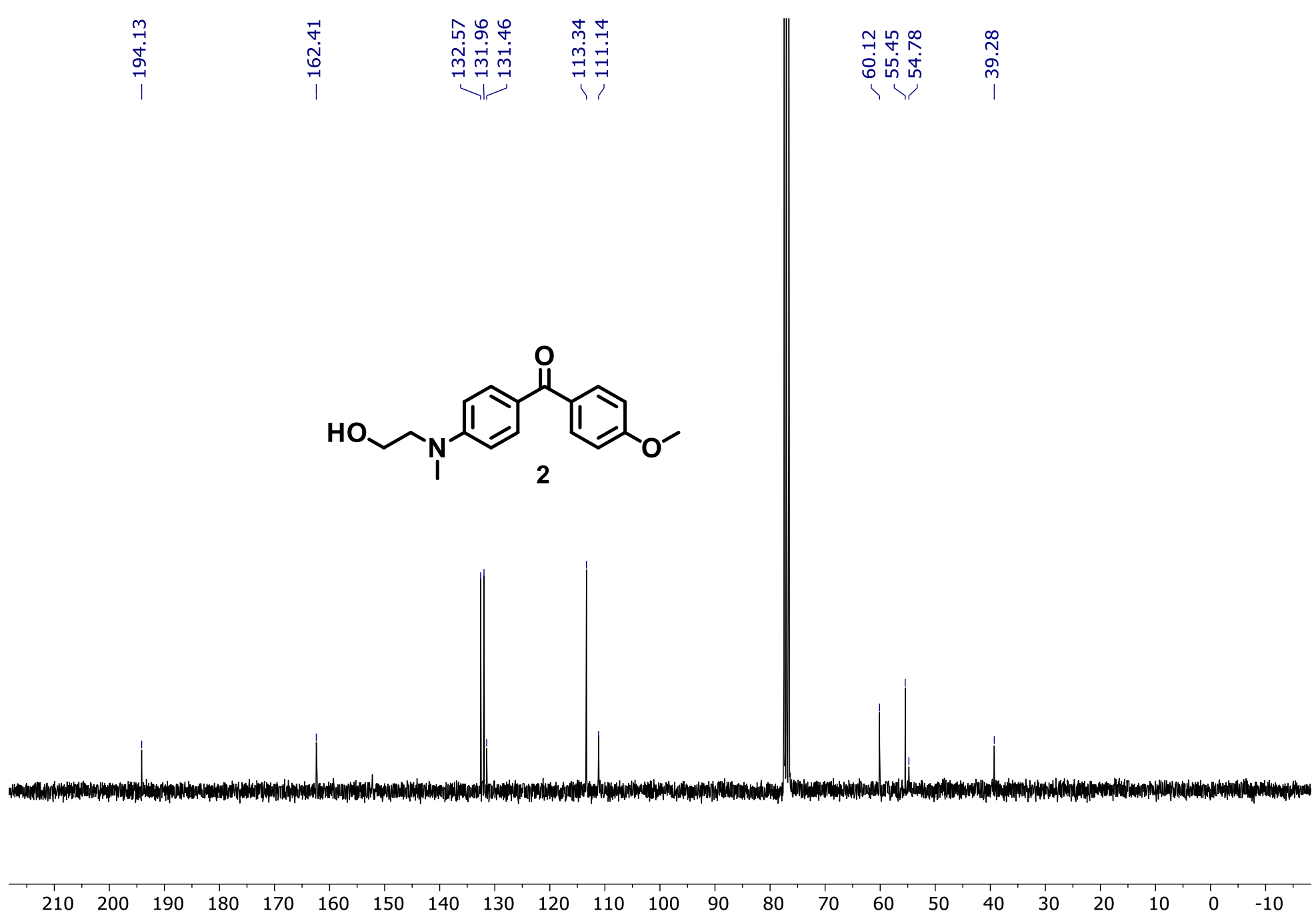

Figure S2: ^{13}C NMR spectrum of **2** (75 MHz, $CDCl_3$, 293 K).

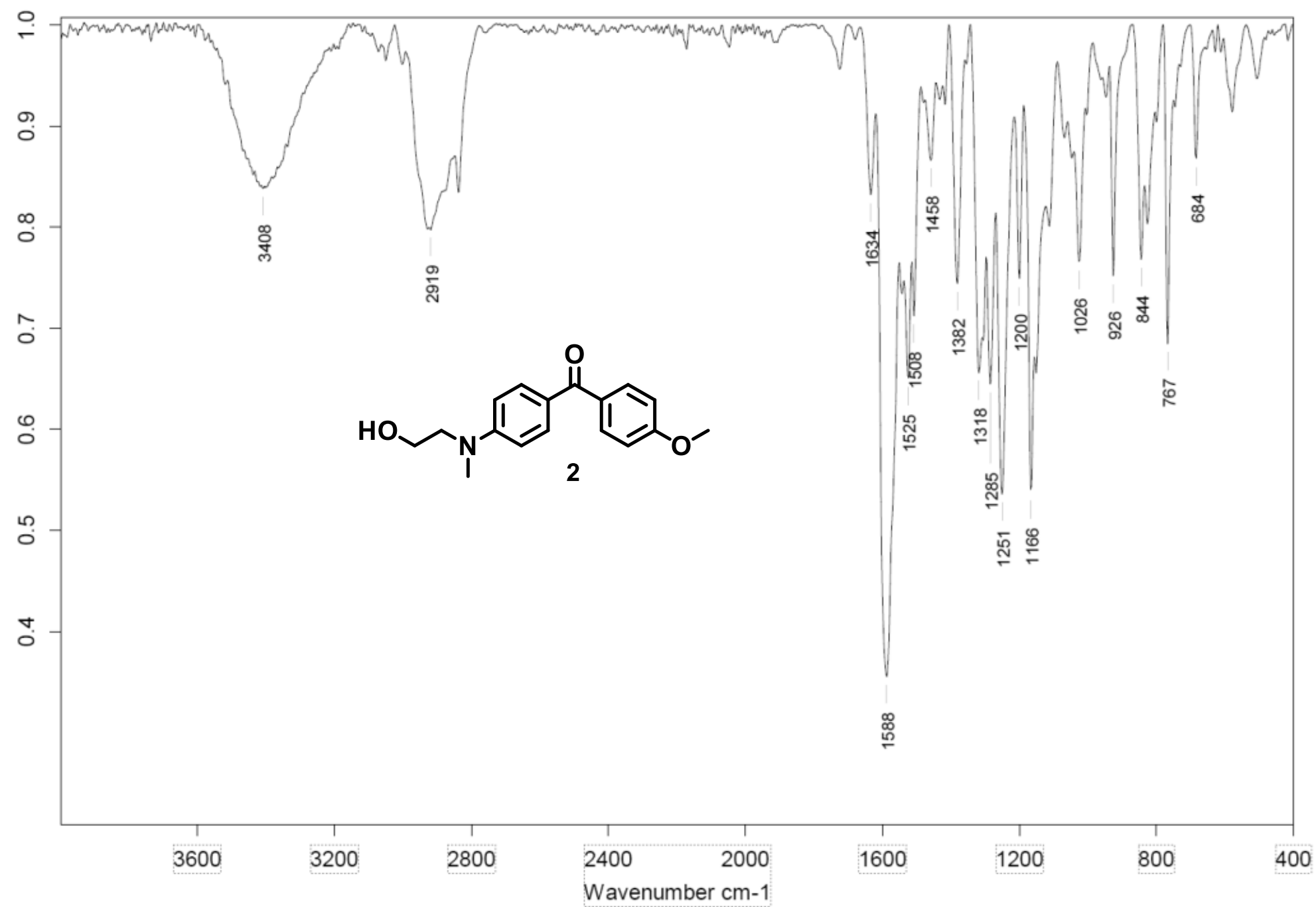

Figure S3: FT-IR spectrum of **2**

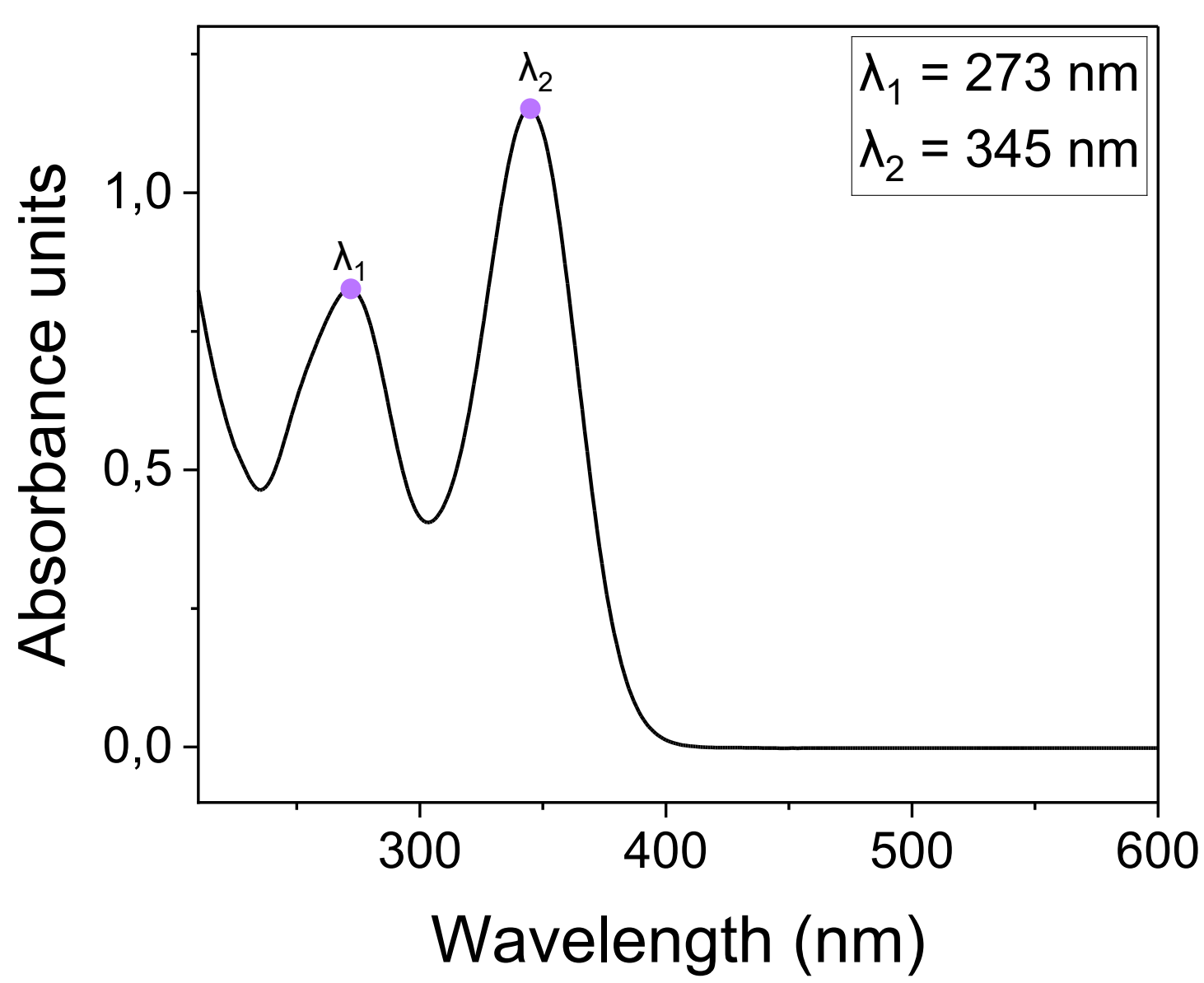

Figure S4: UV-Vis spectrum of **2** (MeCN, 3.2 × 10^{-5} M, 297 K).

(4-methoxyphenyl)(4-(methyl(2-((4vinylbenzyl)oxy)ethyl)amino)phenyl)methanone (3)

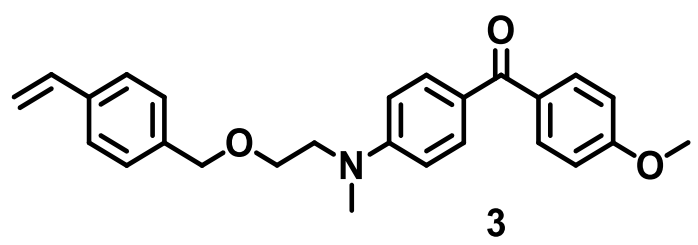

3

In a two-neck, flame-dried round-bottom flask, **2** (1.5 g, 5.2×10^{-3} mol, 1 eq.) was dissolved in dry DMF (10 mL) under an argon flow. When the compound was completely dissolved (yellow solution), potassium *tert*-butoxide (1.02 g, 8.7×10^{-3} mol, 1.6 equivalents) was added under argon. The solution turned orange immediately. The mixture was stirred for 30 minutes at room temperature. Next, 4-vinylbenzyl chloride (1.083 g/mL, 1.11 mL, 7.9×10^{-3} mol, 1.5 equivalents) was added dropwise via syringe through a septum. The reaction was left to proceed for 6 hours, after which it was quenched with saturated aqueous ammonium chloride (NH_4Cl). The mixture was extracted with ethyl acetate (80 mL) and washed sequentially with saturated aqueous NH_4Cl (30 mL), water (30 mL), and brine (30 mL, 4 times). The organic layer was dried over anhydrous Na_2SO_4. The solvent was removed, and the crude product was purified by filtration over a silica gel pad, affording a yellow oil (1.8 g, yield: 85%).

^{1}H NMR (300 MHz, $CDCl_3$): δ/ppm 7.80–7.77 (m, 4H, Ar–H), 7.41–7.38 (m, 2H, Ar–H), 7.28–7.26 (m, 2H, Ar–H), 7.00–6.95 (m, 2H, Ar–H), 6.77–6.68 (m, 3H, Ar–H superimposed with CH_2=CH), 5.79 (dd, J = ~10.3, 17.1 Hz, 1H, CH_2=CH–), 5.28 (dd, J = ~1.2, 10.3 Hz, 1H, CH_2=CH–), 4.53 (s, 2H, O–CH_2–Ar), 3.90 (s, 3H, OCH_3), 3.70–3.68 (m, 4H, $N(CH_3)_2$), 3.11 (s, 3H, N–CH_3).

^{13}C NMR (75 MHz, $CDCl_3$): δ/ppm 194.09, 162.28, 152.11, 137.65, 137.08, 136.48, 132.59, 131.90, 131.70, 127.74, 126.28, 125.40, 113.93, 113.30, 110.55, 73.10, 67.54, 55.44, 52.12, 39.20.

FT-IR $(cm)^{-1}$: 2857, 1634, 1524, 1457, 1318, 1300, 1285, 1260, 1200, 1167, 1100, 1028, 981, 926, 843, 824, 787, 684, 679, 507.

HR-MS (m/z): $[M+H]^+$ calcd for $C_{26}H_{28}NO_3$ 402.2080; found 402.2064.

UV-Vis: Extinction coefficients (ε [$L\cdot mol^{-1}\cdot cm^{-1}$]) in the λ maxima ($\lambda_{1-2}$) of 2.6×10^{-5} M MeCN solution of **3**

λ_1, nm (ε): 254 (32692).

λ_2, nm (ε): 345 (24615).

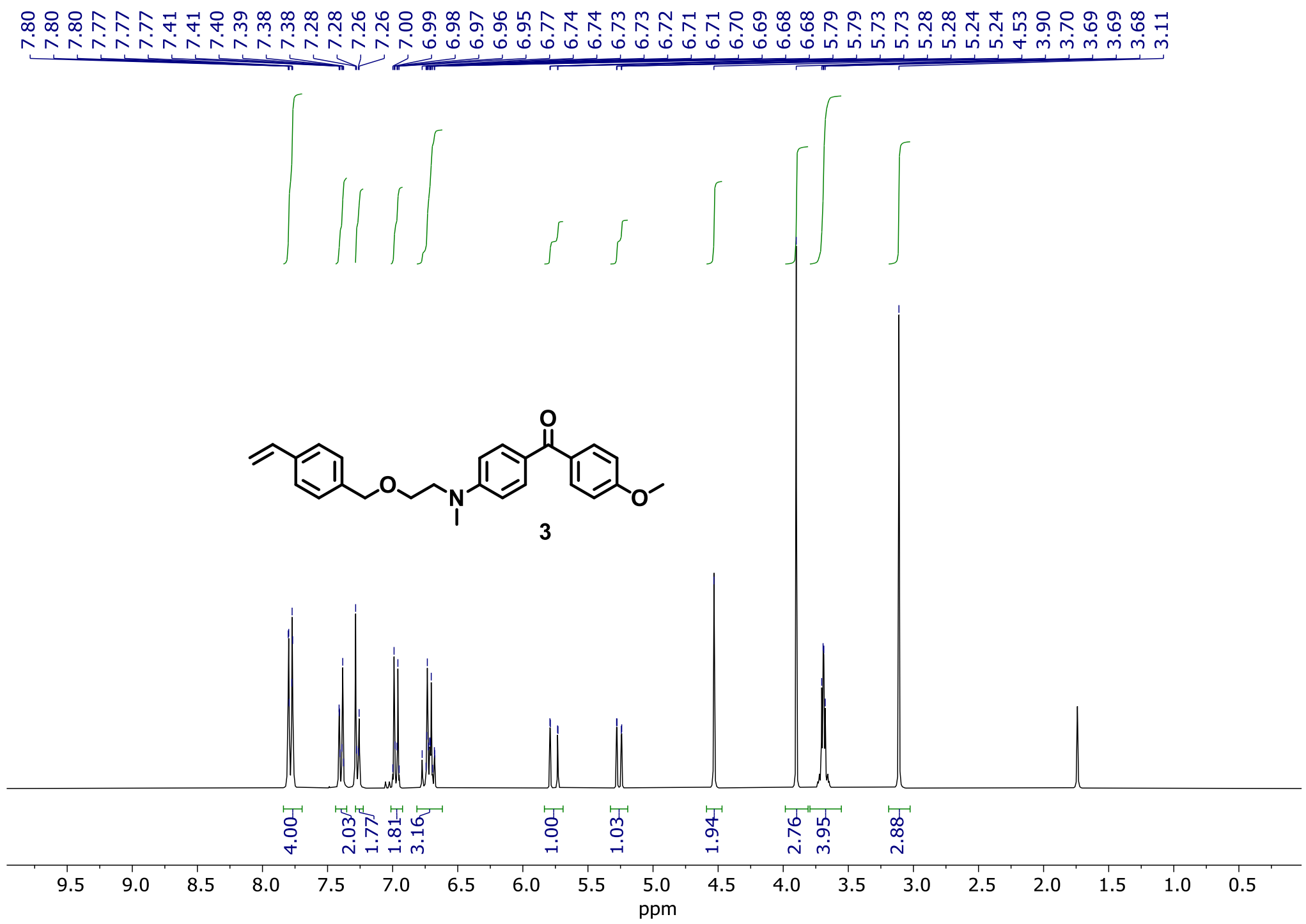

Figure S5: ^{1}H NMR spectrum of **3** (300 MHz, $CDCl_3$, 293 K).

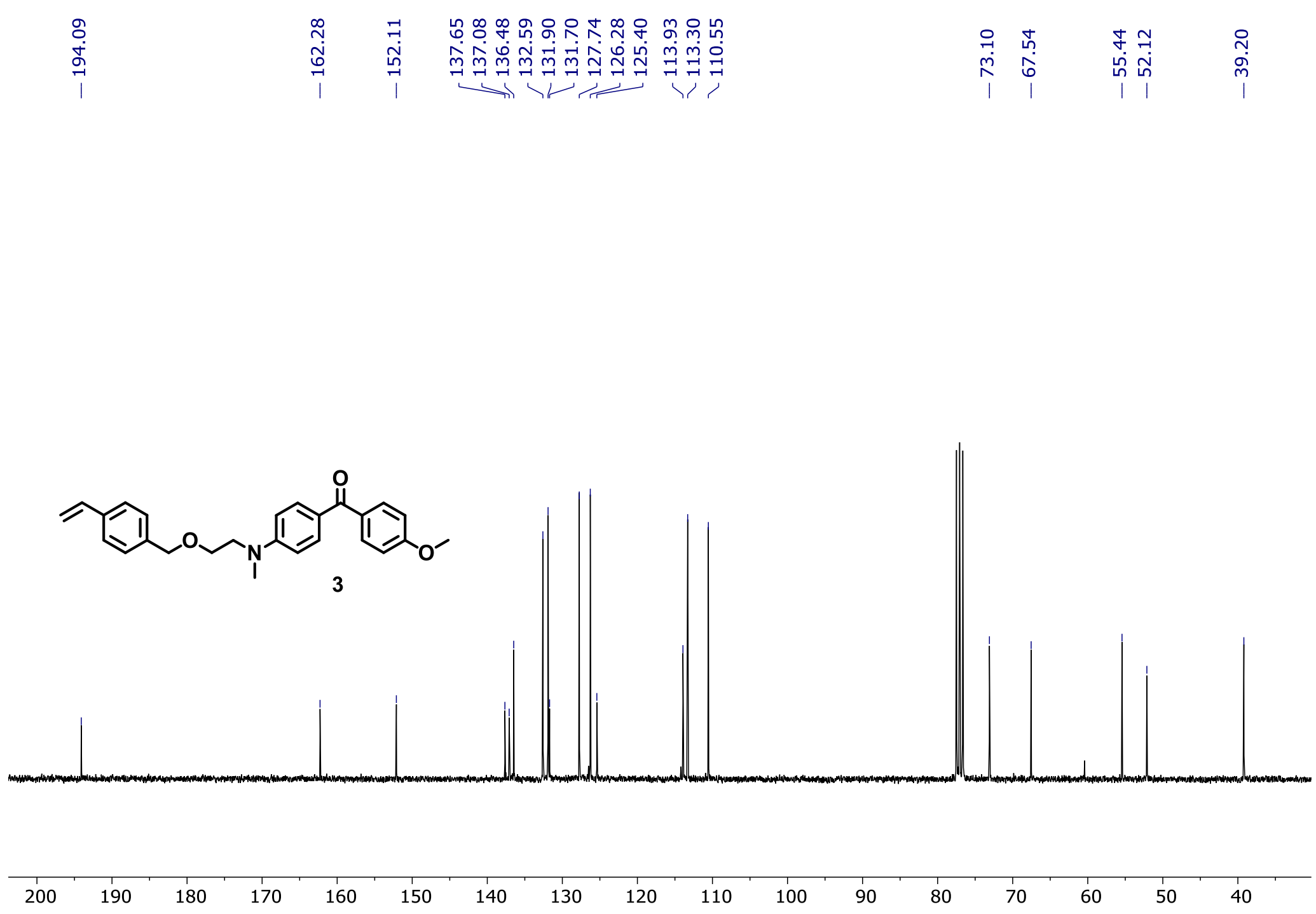

Figure S6: ^{13}C NMR spectrum of **3** (75 MHz, $CDCl_3$, 293 K).

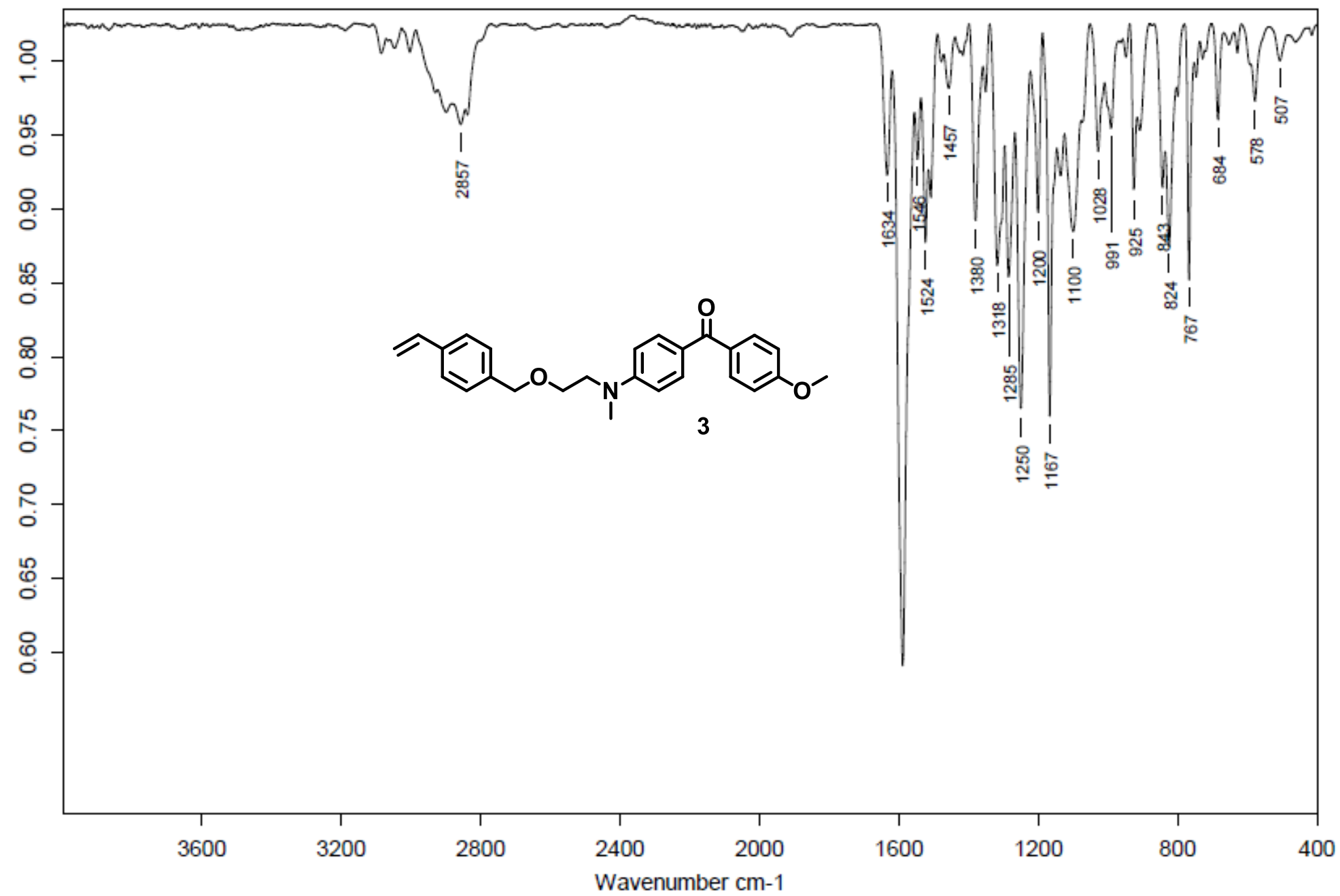

Figure S7: FT-IR spectrum of **3**

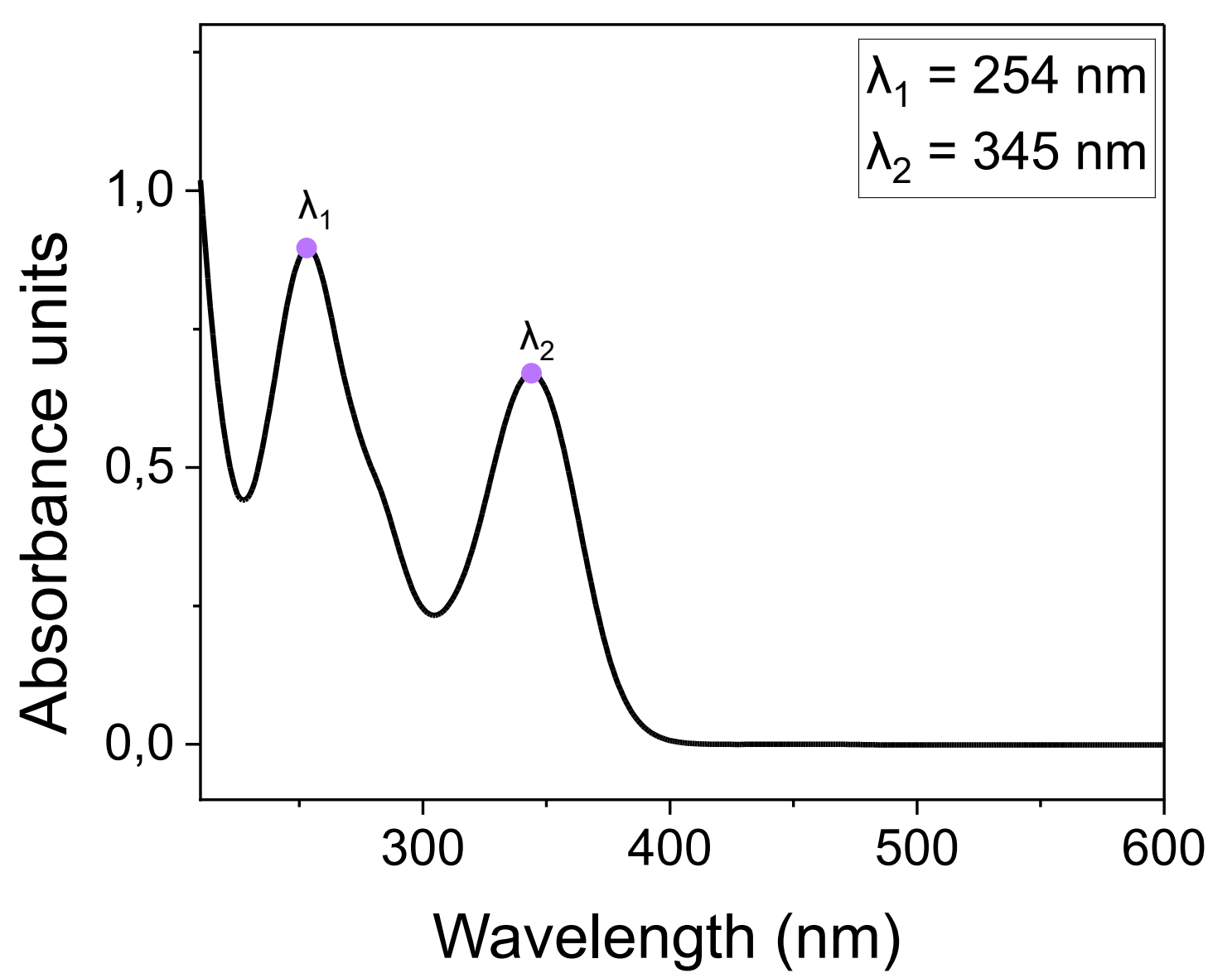

Figure S8: UV-Vis spectrum of **3** (MeCN, 2.6 × 10^{-5} M, 297 K).

(4-methoxyphenyl)(4-(methyl(2-((4-vinylbenzyl)oxy)ethyl)amino)phenyl)methanol (4)

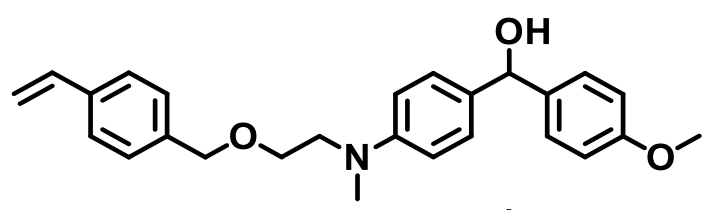

4

In a round-bottom flask, potassium hydroxide (14.0 mg, 0.25 $\times 10^{-3}$ mol, 0.1 eq.) was dissolved in ethanol (8 mL). Compound **3** (1.0 g, 2.5 $\times 10^{-3}$ mol, 1 eq.) was added, affording a yellow suspension. Sodium borohydride (47.1 mg, 1.25 $\times 10^{-3}$ mol, 0.5 mol eq.) was added in small portions, and the reaction mixture was heated at reflux for 3 h. Two additional portions of sodium borohydride were subsequently added (I: 47.1 mg, 1.25 $\times 10^{-3}$ mol, 0.5 mol eq.; II: 47.1 mg, 1.25 $\times 10^{-3}$ mol, 0.5 mol eq.), and the mixture was heated at reflux for 3 h after each addition. After cooling to room temperature, the solvent was removed under reduced pressure. The residue was dissolved in dichloromethane (30 mL) and washed with water (10 mL) and brine (10 mL). The organic layer was dried over anhydrous Na_2SO_4, filtered, and concentrated under reduced pressure to afford the title compound as a yellow oil (0.9 g, yield: 90%).

^{1}H NMR (300 MHz, $CDCl_3$): δ/ppm 7.44–7.22 (m, 9H, Ar–H), 6.94–6.71 (m, 3H, Ar–H superimposed with CH_2=CH), 5.83–5.76 (dd, *J* = ~10.3, 17.1 Hz, 1H, CH_2=CH–), 5.32–5.28 (dd, *J* = ~1.2, 10.3 Hz, 1H, CH_2=CH–), 4.54 (s, 2H, O–CH_2–Ar), 3.83 (s, 3H, OCH_3), 3.70–3.57 (m, 4H, $N(CH_3)_2$), 3.01 (s, 3H, N–CH_3), 2.73 (brd. 1H, OH).

^{13}C NMR (75 MHz, $CDCl_3$): δ/ppm 158.78, 148.69, 137.91, 137.01, 136.60, 131.98, 127.80, 127.78, 127.72, 126.29, 113.87, 113.84, 113.73, 112.02, 75.54, 73.04, 67.73, 55.31, 52.50, 39.11.

FT-IR $(cm)^{-1}$: 3428, 3085, 2857, 1610, 1580, 1567, 1477, 1450, 1405, 1351, 1278, 1209, 1181, 1095, 1015, 990, 905, 813, 730, 559.

HR-MS (m/z): $[M+H]^+$ calcd for $C_{26}H_{30}NO_3$ 404.2222; found 404.2220.

UV-Vis: Extinction coefficients (ε [$L \cdot mol^{-1} \cdot cm^{-1}$]) in the λ maxima ($\lambda_1$) of 2.3 × 10^{-5} M MeCN solution of **4**

λ_1, nm (ε): 257 (33913).

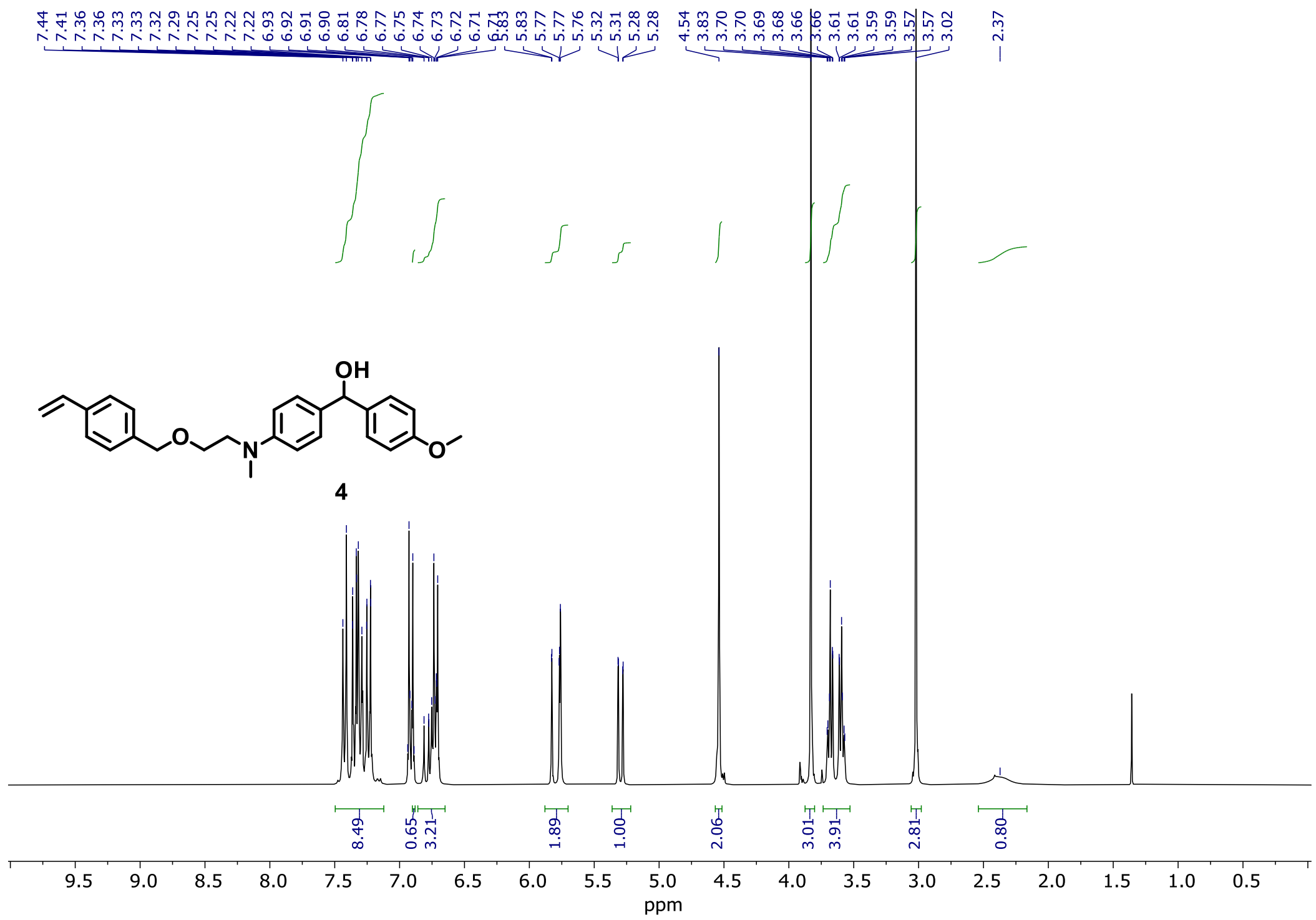

Figure S9: ^{1}H NMR spectrum of **4** (300 MHz, $CDCl_3$, 293 K).

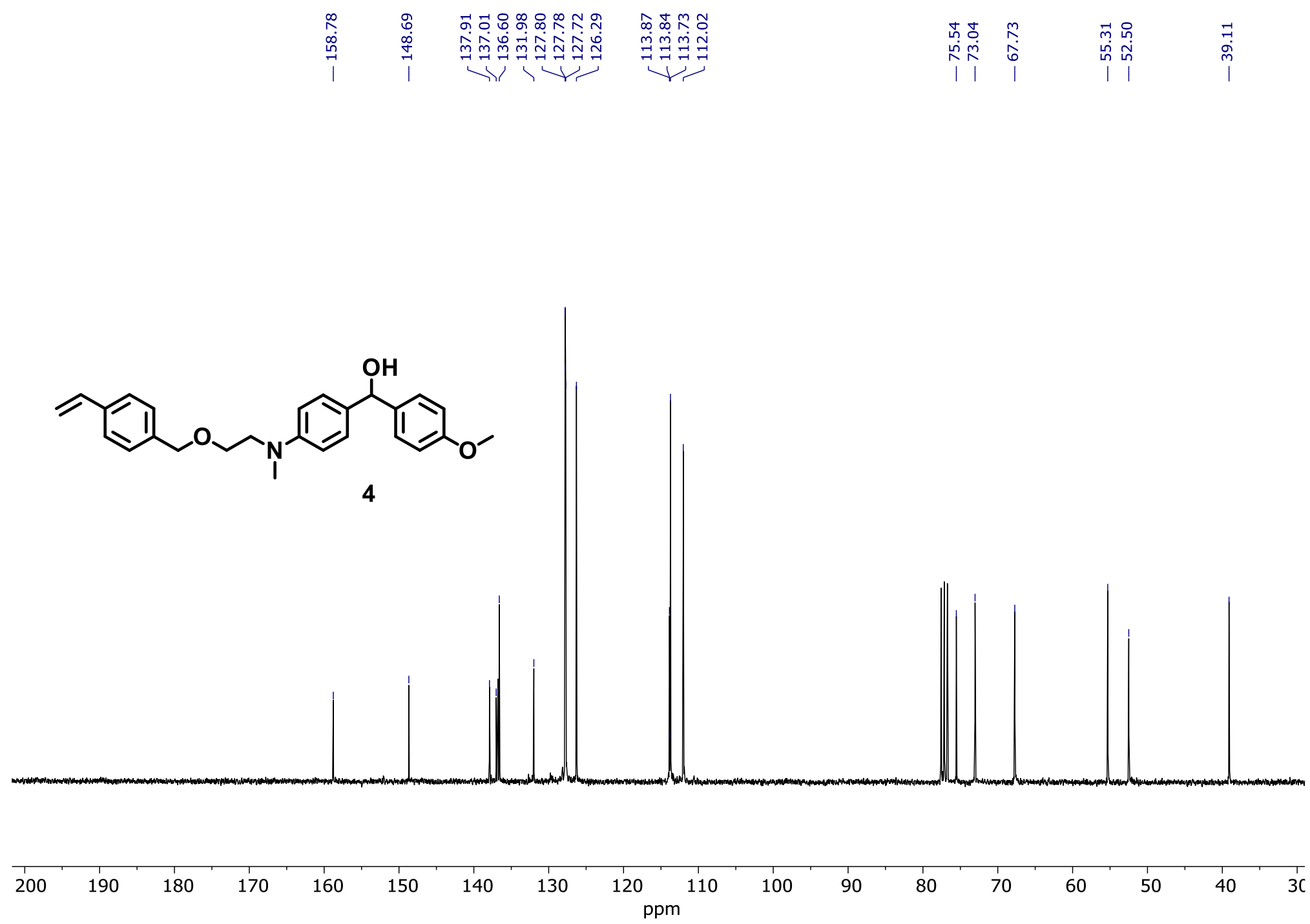

Figure S10: ^{13}C NMR spectrum of **4** (75 MHz, $CDCl_3$, 293 K).

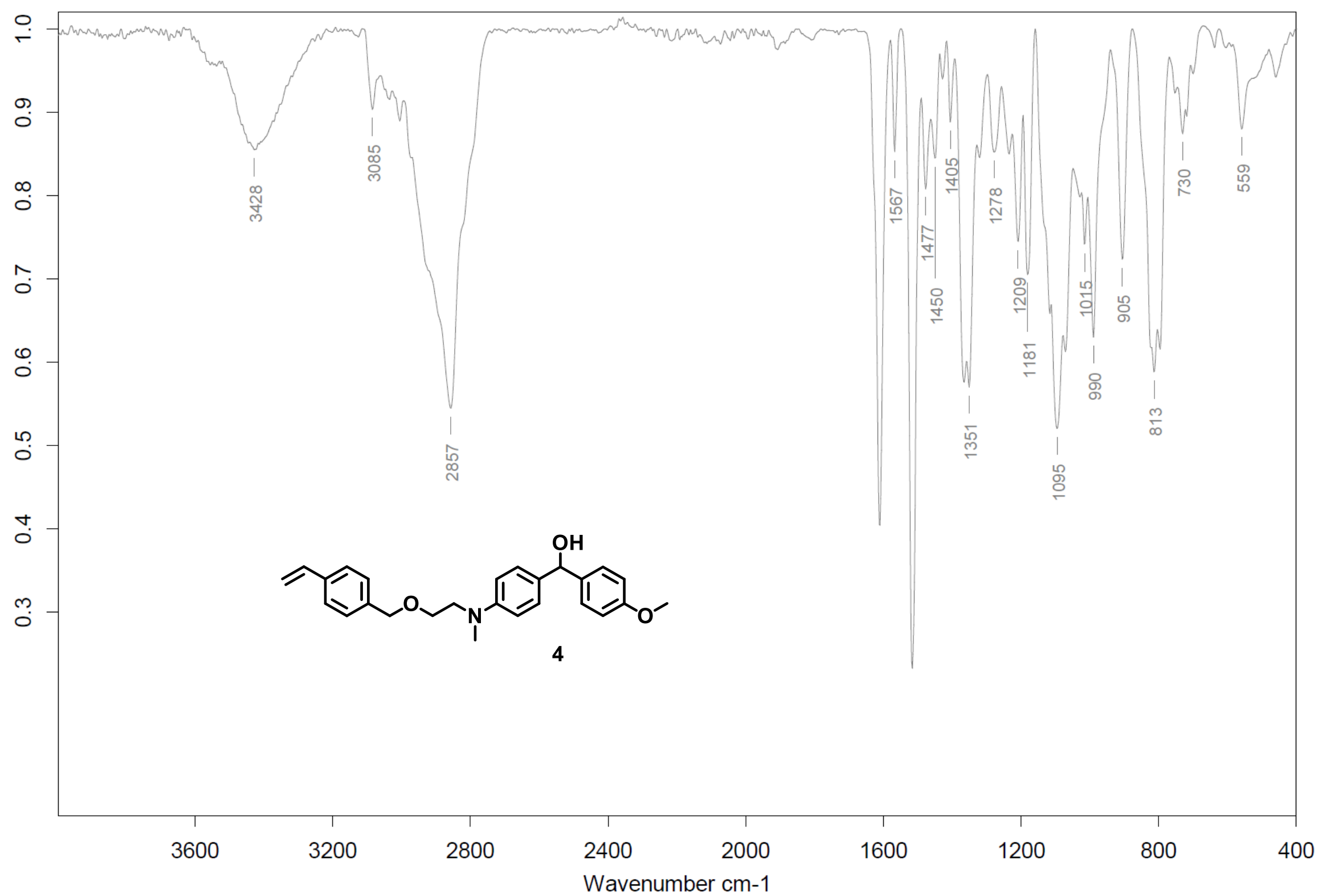

Figure S11: FT-IR spectrum of **4**.

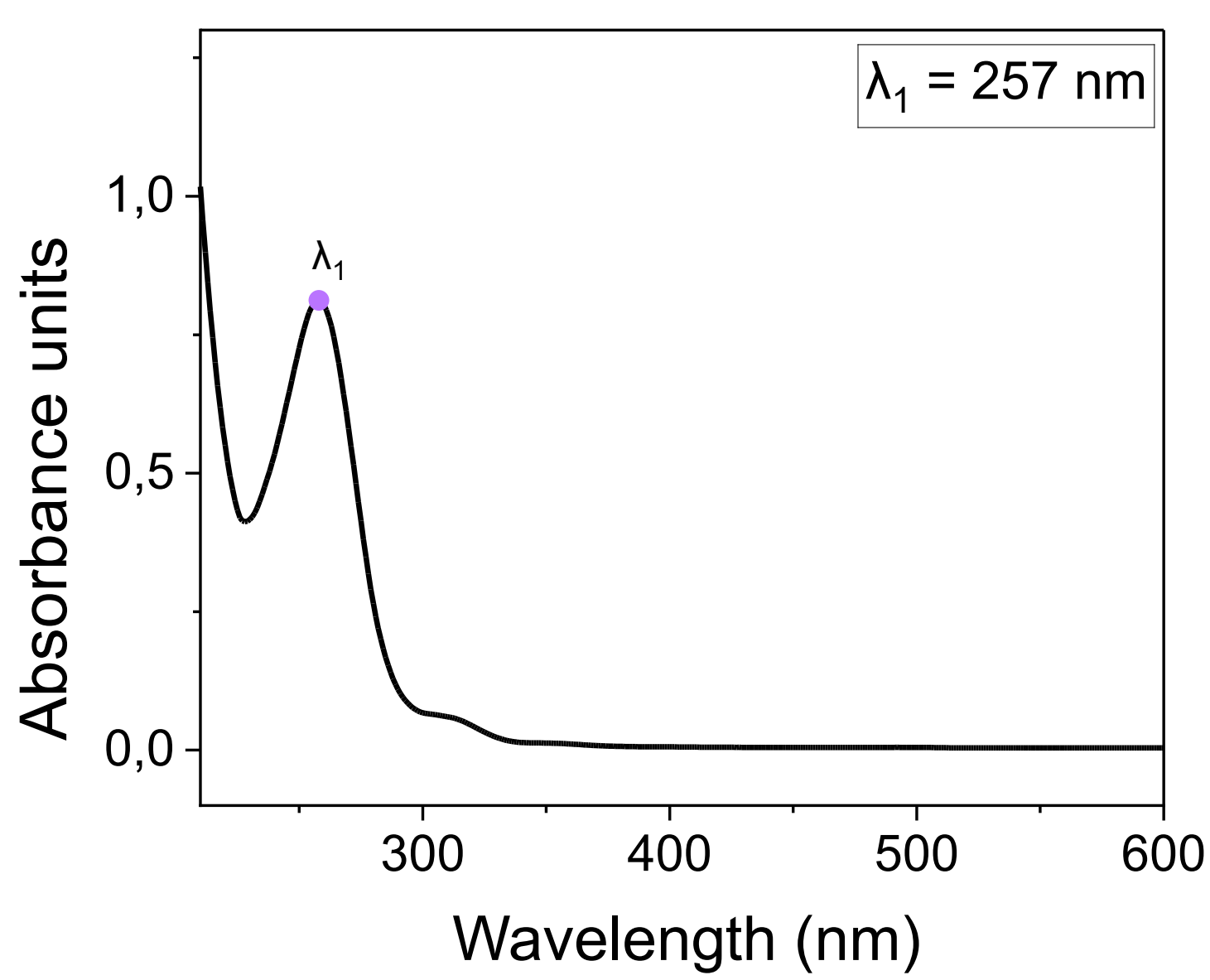

Figure S12: UV-Vis spectrum of **4** (MeCN, 2.3 × 10^{-5} M, 297 K).

bis(4-((2-hydroxyethyl)(methyl)amino)phenyl)methanone (7)

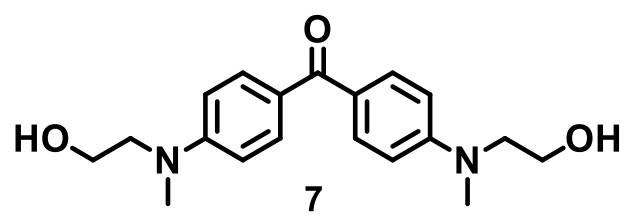

1 (1.0 g, 4.6×10^{-3} mol, 1 eq.) and *N*-methyl ethanolamine (1.4 g, 1.8×10^{-2} mol, 4 eq.) were combined in sulfolane (8 mL) in a microwave vial. The vial was sealed and heated in a microwave at 185 °C for 4 hours, with stirring. The mixture was poured into ice-cold water, resulting in the precipitation of a solid. The precipitate was collected by filtration, washed with water, and air-dried. The crude product was recrystallized from methanol, affording the pure compound as an intense yellow solid (1.5 g, yield: 99%).

^{1}H-NMR (300 MHz, CD_3OD): δ/ppm 7.76–7.73 (m, 4H, Ar-H), 6.90–6.86 (m, 4H, Ar-H), 3.85–3.82 (t, *J* = 5.5 Hz, 4H, -N-CH_2-CH_2-OH), 3.70–3.66 (m, 4H, -N-CH_2-CH_2-OH), 3.19 (s, 6H, -N-CH_3).

^{13}C-NMR (75 MHz, CD_3OD): δ/ppm 195.34, 152.51, 132.10, 125.23, 110.24, 58.80, 53.80, 38.00.

FT-IR $(cm)^{-1}$: 3367, 3298, 2907, 1673, 1602, 1519, 1390, 1267, 1220, 1174, 1112, 1056, 987

HR-MS (m/z): $[M+H]^+$ calcd for $C_{19}H_{25}N_2O_3$ 329.1844; found 329.1860.

UV-Vis: Extinction coefficients (ε [$L\cdot mol^{-1}\cdot cm^{-1}$]) in the λ maxima ($\lambda_1$) of 3.9×10^{-5} M MeCN solution of **7**

λ_1, nm (ε): 354 (38461).

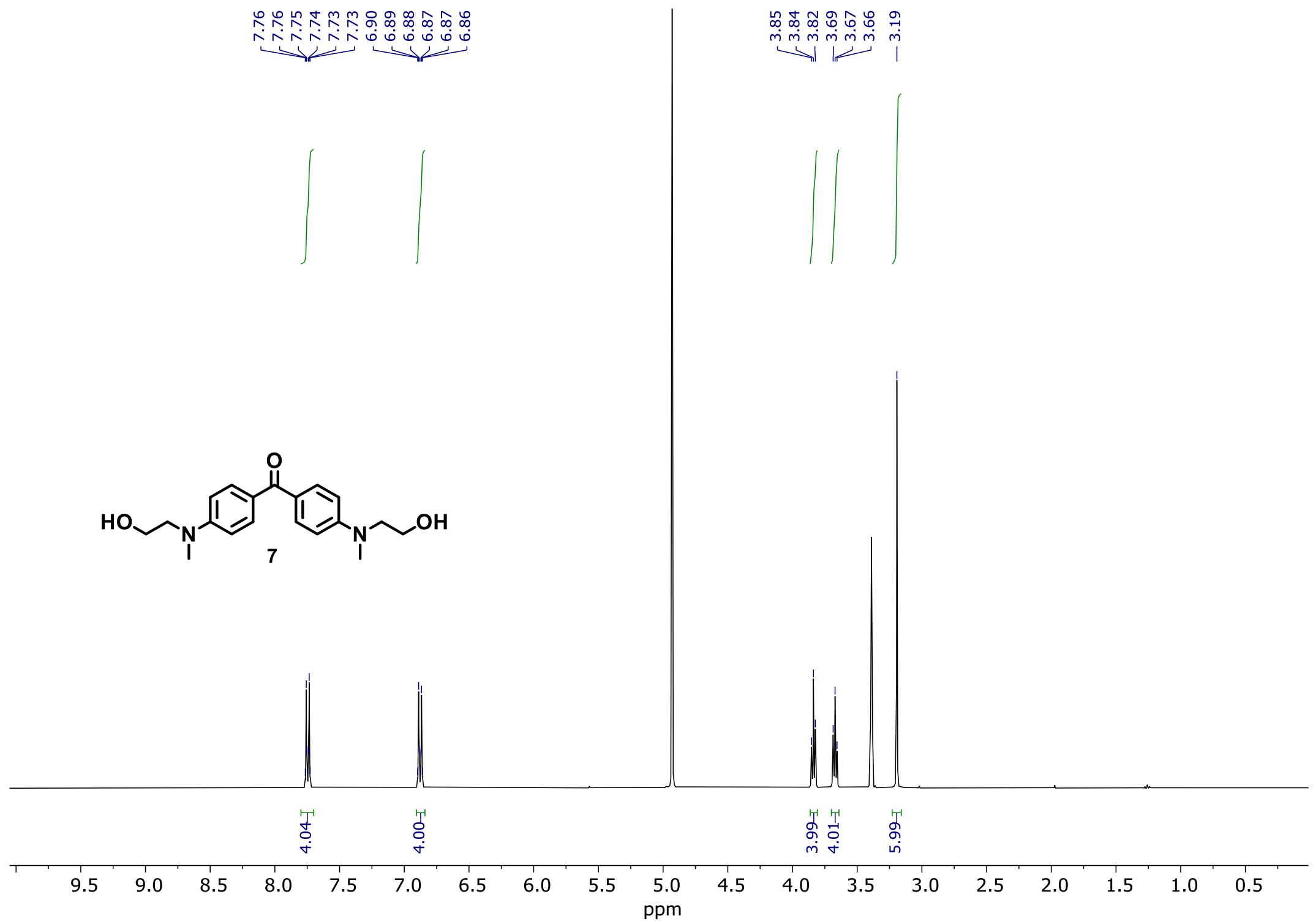

Figure S13: ^{1}H NMR spectrum of **7** (300 MHz, CD_3OD, 293 K).

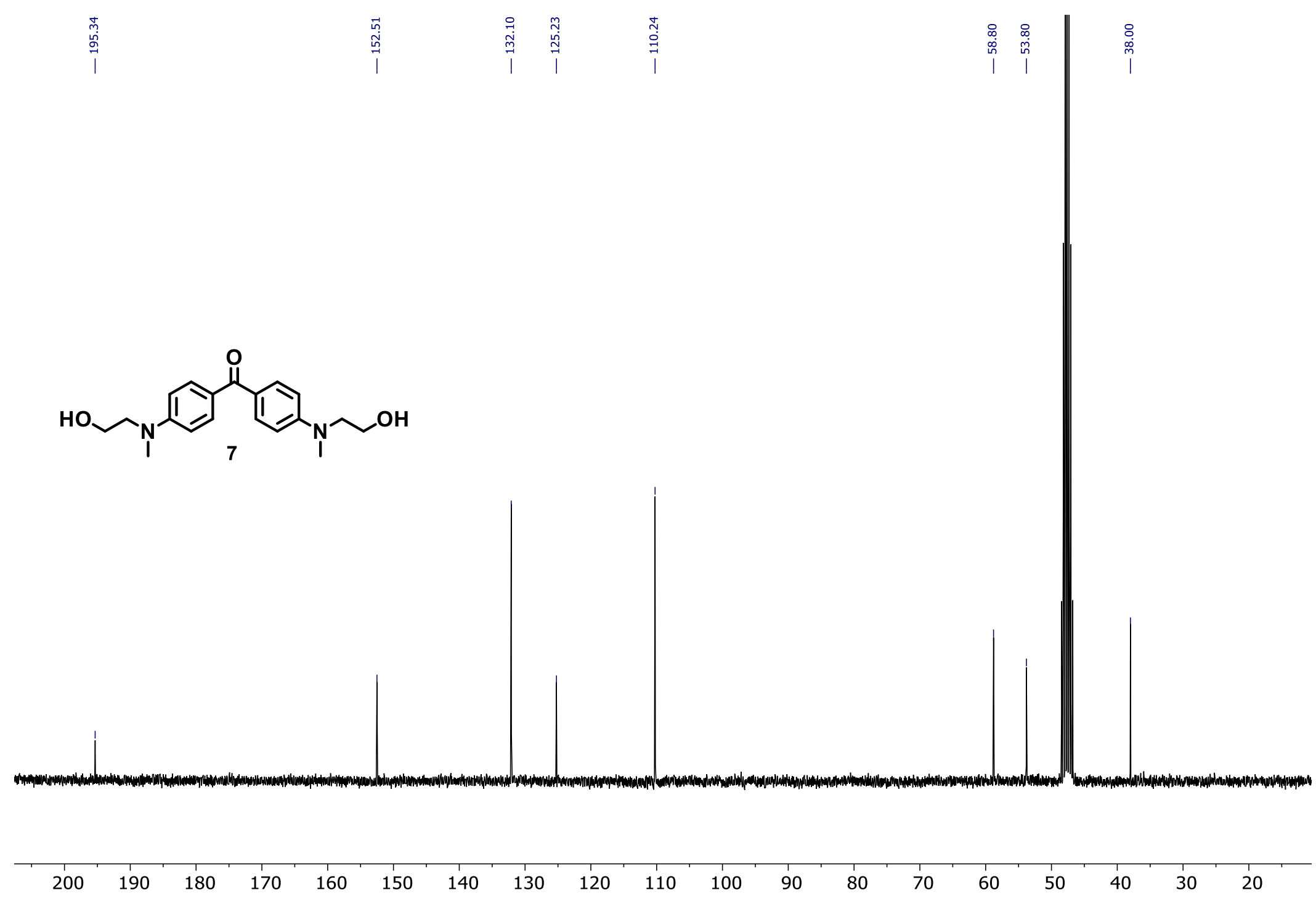

Figure S14: ^{13}C NMR spectrum of **7** (75 MHz, CD_3OD, 293 K).

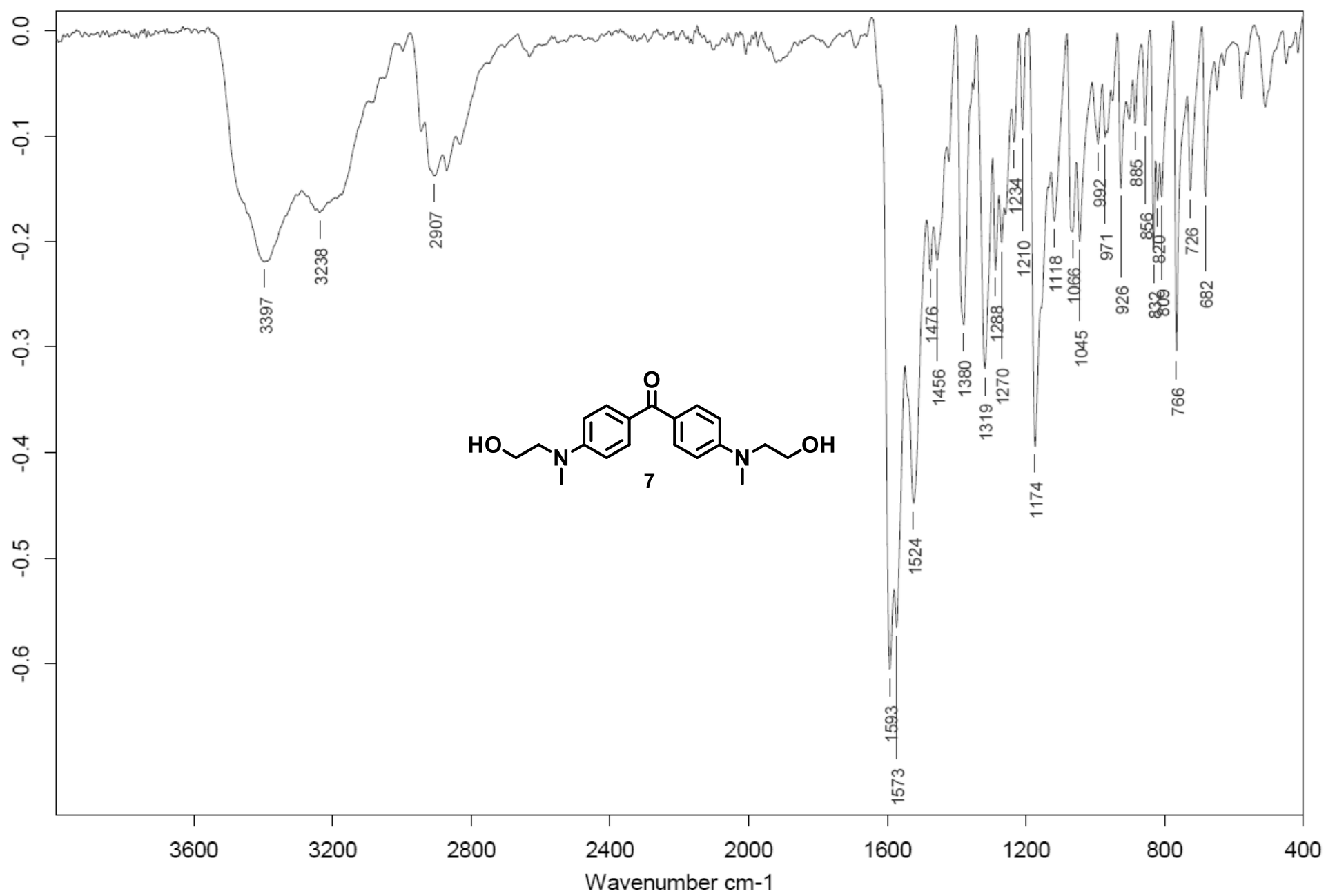

Figure S15: FT-IR spectrum of **7**

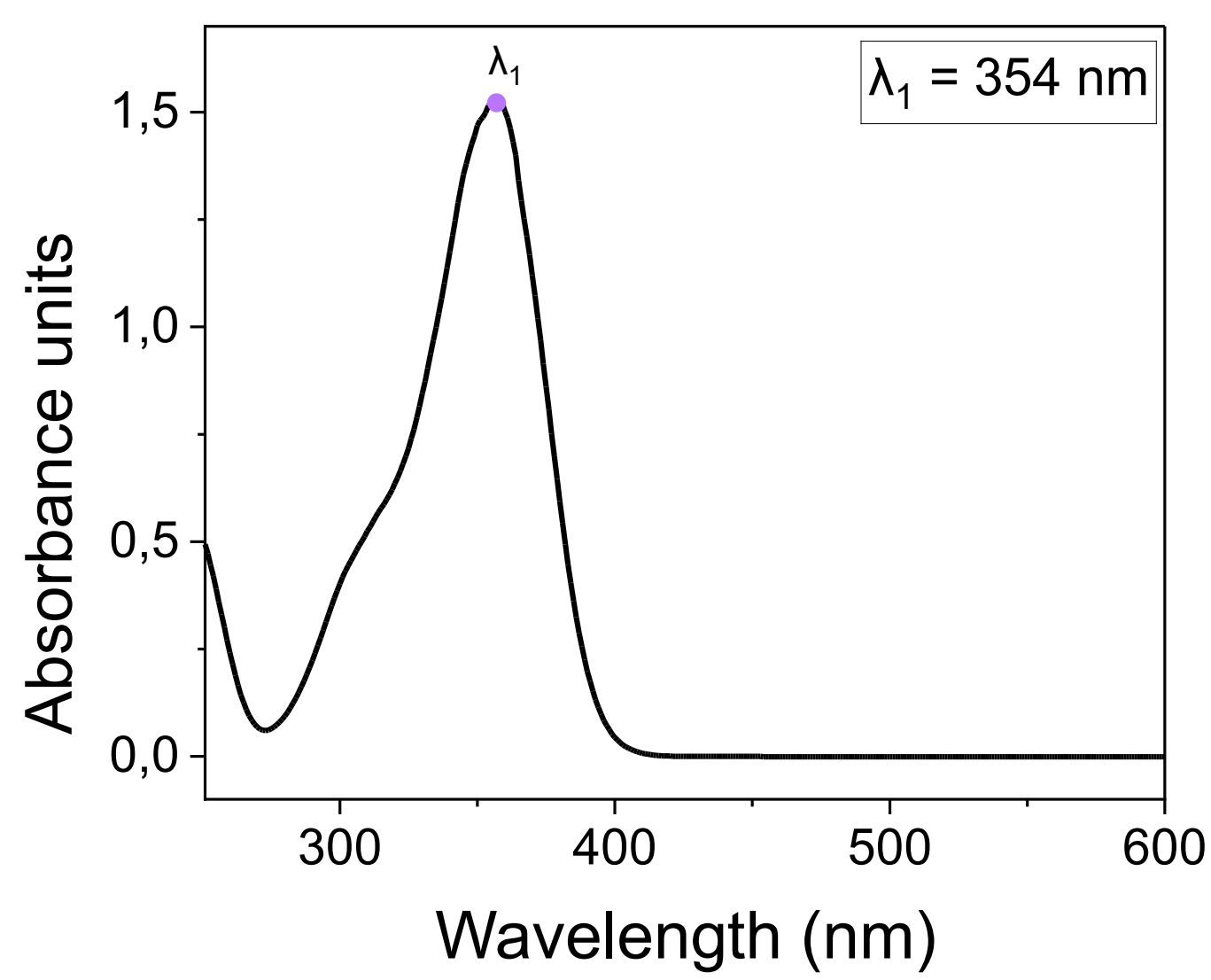

Figure S16: UV-Vis spectrum of **7** (MeCN, 3.9 × 10^{-5} M, 297 K).

(4-((2-hydroxyethyl)(methyl)amino)phenyl)(4-(methyl(2-((4-vinylbenzyl)oxy)ethyl)amino)phenyl)methanone (8)

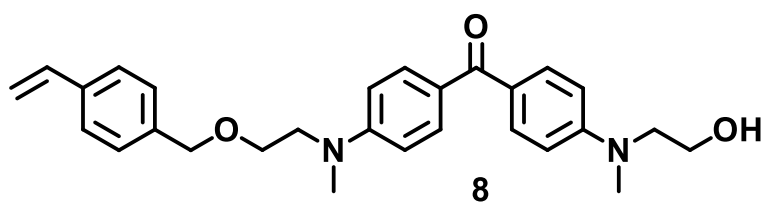

In a two-neck, flame-dried round-bottom flask, **7** (0.6 g, 1.8 $\times 10^{-3}$ mol, 1 eq.) was dissolved in dry DMF (10 mL) under argon. After complete dissolution, potassium *tert*-butoxide (0.4 g, 3.7 $\times 10^{-3}$ mol, 2.1 eq.) was added under argon, and the solution immediately turned from yellow to orange. The reaction mixture was stirred for 30 min. 4-vinylbenzyl chloride (1.083 g/mL, 0.28 mL, 2.0 $\times 10^{-3}$ mol, 1.1 eq.) was then added dropwise via syringe through a septum, and the reaction was stirred for 3 h. The reaction was quenched with saturated aqueous ammonium chloride (10 mL). The mixture was extracted with ethyl acetate (80 mL) and washed sequentially with saturated aqueous NH_4Cl (20 mL), water (30 mL), and brine (30 mL, 4 times). The organic layer was dried over anhydrous Na_2SO_4, filtered, and concentrated under reduced pressure. The crude product was purified by column chromatography to afford compound **8** (0.34 g, yield: 45%).

^{1}H NMR (300 MHz, $CDCl_3$): δ/ppm 7.77–7.74 (m, 4H, Ar–H), 7.41–7.38 (m, 2H, Ar–H), 7.26 (m, 2H, Ar–H), 6.77–6.68 (m, 5H, Ar–H superimposed with CH_2=CH), 5.79-5.73 (dd, $J \approx 1.2$, 1H, CH_2=CH–), 5.27-5.24 (dd, $J \approx 1.2$, 1H, CH_2=CH–), 4.53 (s, 2H, O–CH_2–Ar), 3.89–3.59 (m, 8H, –N(CH_3)CH_2CH_2OH), 3.10 (s, 3H, N–CH_3), 2.17 (brs, 1H, OH).

^{13}C NMR (75 MHz, $CDCl_3$): δ/ppm 193.93, 152.11, 151.74, 137.69, 137.06, 136.49, 132.35, 132.31, 127.74, 126.63, 126.27, 126.15, 113.90, 110.77, 110.50, 73.10, 67.59, 60.13, 54.58, 52.14, 39.18, 39.03.

FT-IR (cm)$^{-1}$: 3376, 2916, 1658, 1629, 1598, 1470, 1424, 1370, 1294, 1234, 1193, 1178, 1116, 965, 925, 796, 693.

HR-MS (m/z): $[M+H]^+$ calcd for $C_{28}H_{33}N_2O_3$ 445.2492; found 445.2486.

UV-Vis: Extinction coefficients (ε [L·mol^{-1}·cm^{-1}]) in the λ maxima ($\lambda_{1\text{-}2}$) of 2.4 × 10^{-5} M MeCN solution of **8**

λ_1, nm (ε): 254 (37525).

λ_2, nm (ε): 356 (31250).

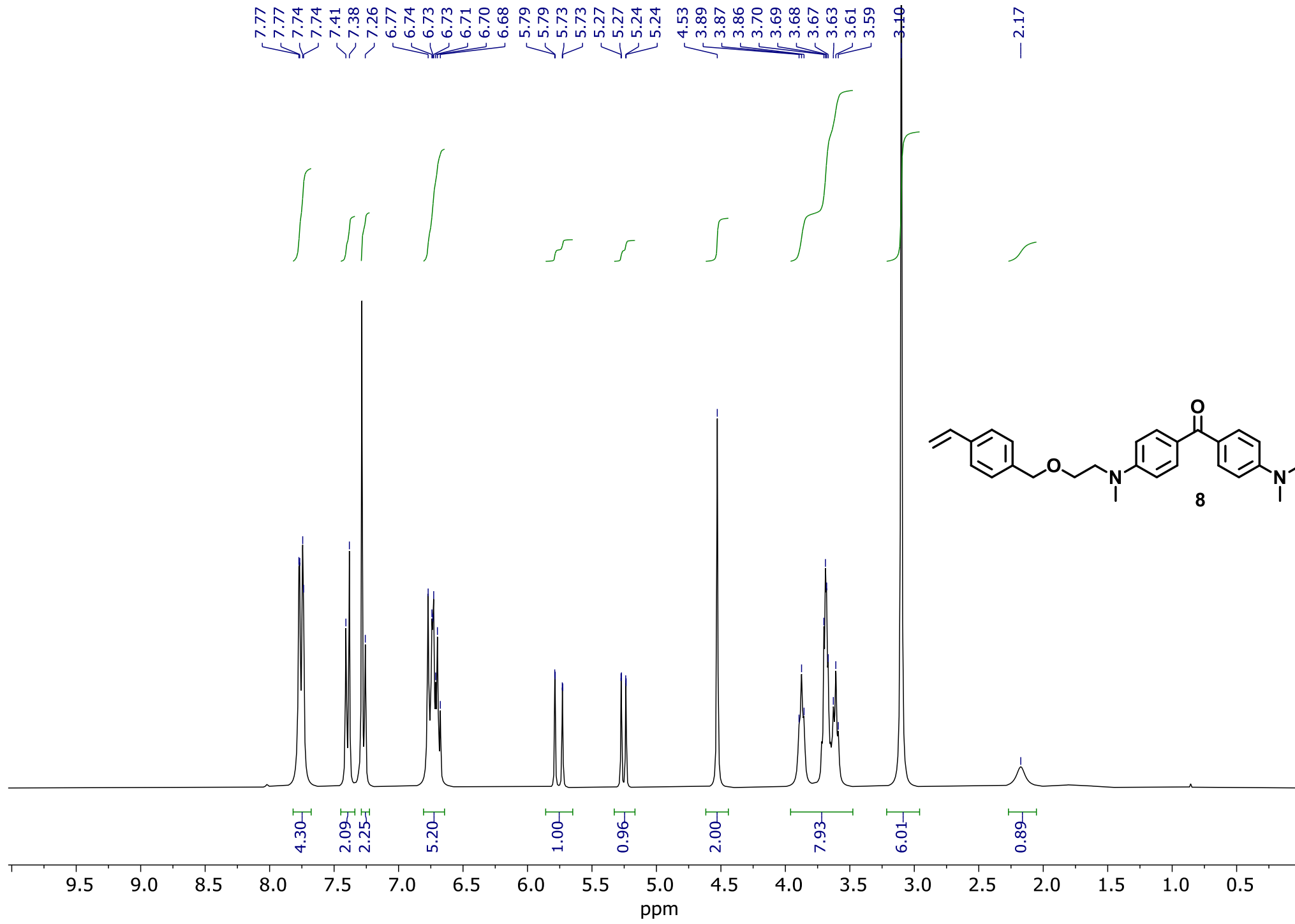

Figure S17: ^{1}H NMR spectrum of **8** (300 MHz, $CDCl_3$, 293 K).

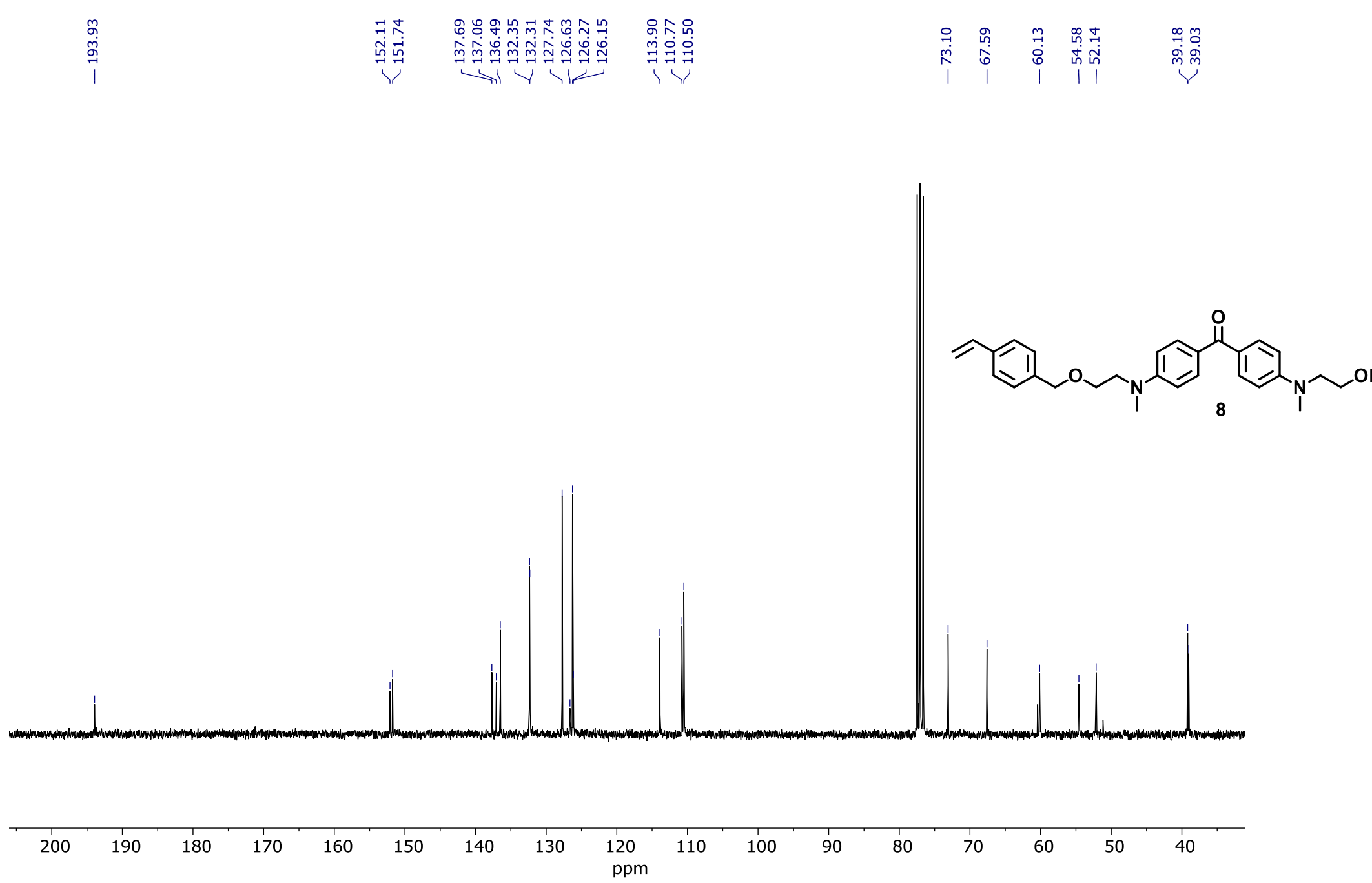

Figure S18: ^{13}C NMR spectrum of **8** (75 MHz, $CDCl_3$, 293 K).

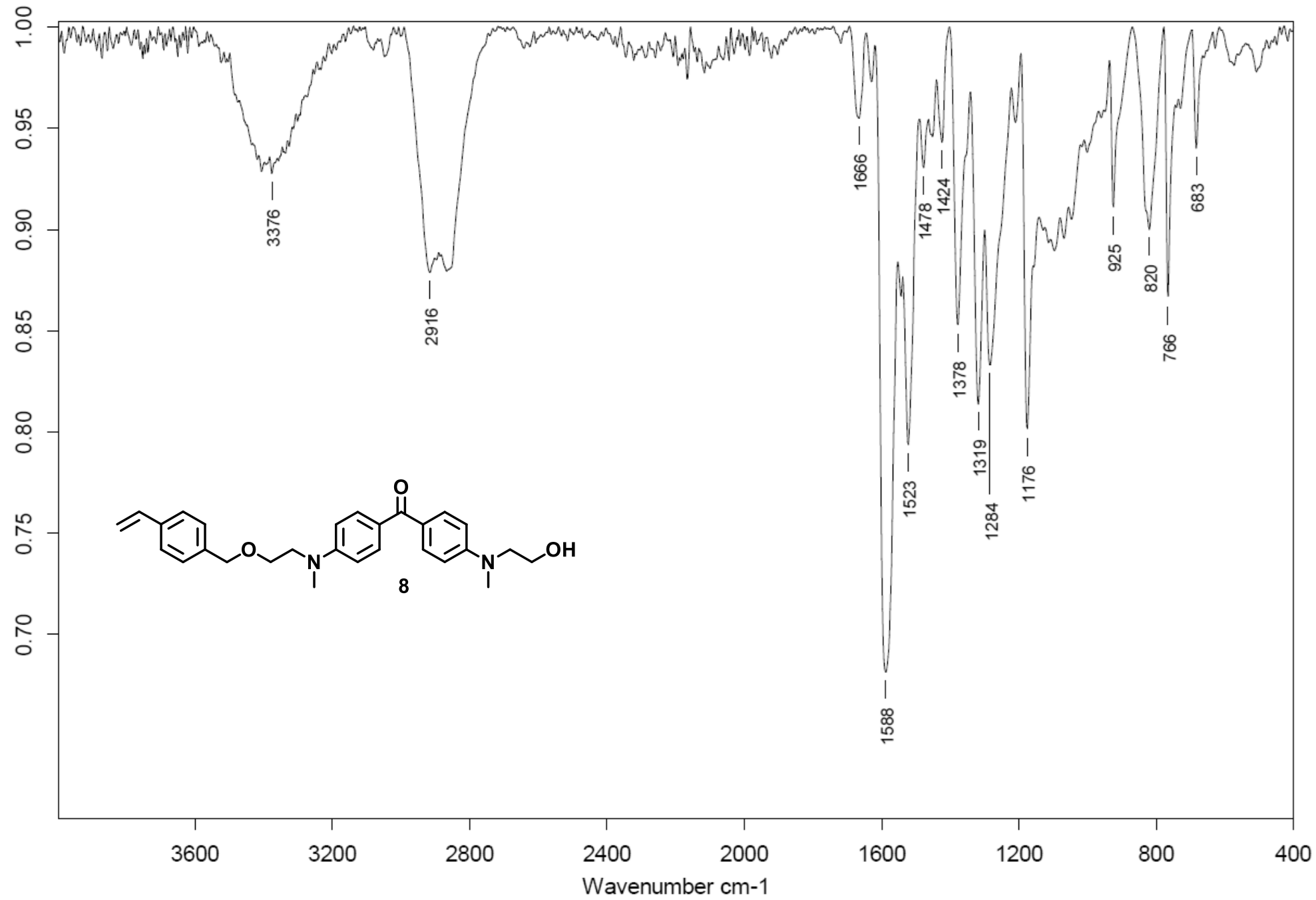

Figure S19: FT-IR spectrum of **8**

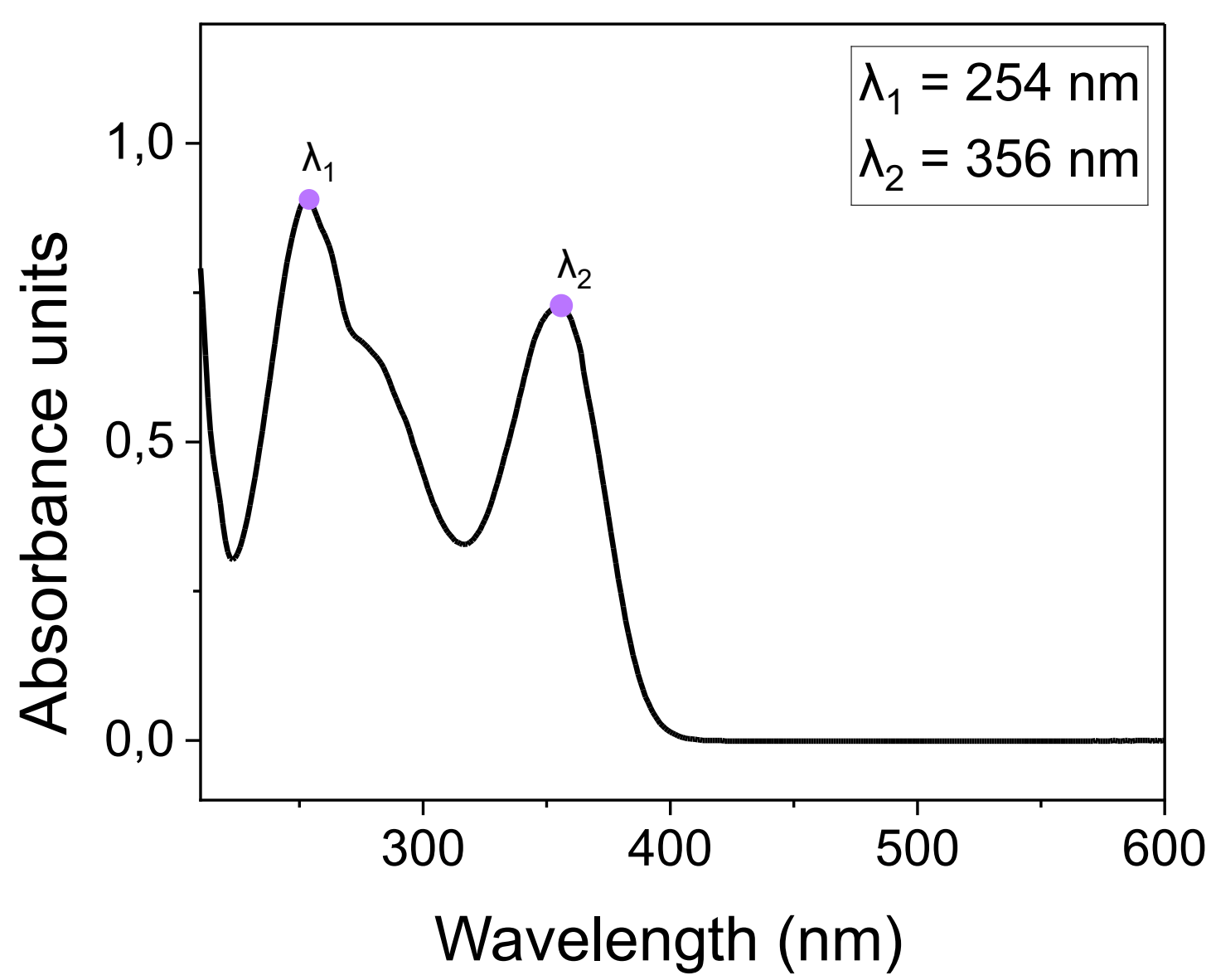

Figure S20: UV-Vis spectrum of **8** (MeCN, 2.4×10^{-5} M, 297 K).

(4-((2-methoxyethyl)(methyl)amino)phenyl)(4-(methyl(2-((4-vinylbenzyl)oxy)ethyl)amino)phenyl)methanone (9)

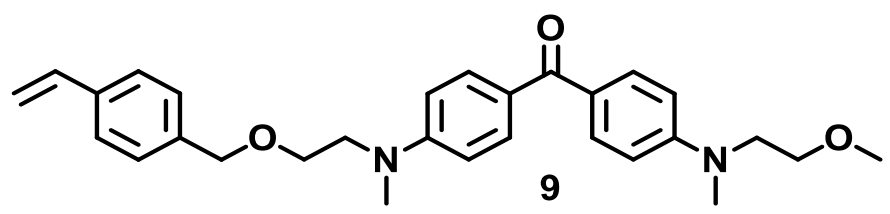

In a two-neck, flame-dried round-bottom flask under a nitrogen atmosphere, **8** (0.7 g, 2.1×10^{-3} mol, 1 eq.) was dissolved in dry DMF (15 mL). Potassium *tert*-butoxide (0.3 g, 2.7×10^{-3} mol, 1.3 eq.) was added in one portion, and the reaction mixture was stirred at room temperature for 15 min. A solution of iodomethane (0.3 g, 2.4×10^{-3} mol, 1.1 eq.) in dry DMF (2 mL) was added dropwise, and the reaction mixture was stirred at room temperature for an additional 30 min. The solvent was removed under reduced pressure. The residue was dissolved in ethyl acetate and filtered to remove inorganic salts, affording **9** as pure (0.75 g, yield: 85%).

^{1}H NMR (300 MHz, $CDCl_3$): δ/ppm 7.79–7.26 (m, 8H, Ar–H), 7.29–6.71 (m, 4H, Ar–H), 6.74–6.68 (dd, 1H, CH=, *J* ≈ 17.5, 10.8 Hz), 5.79–5.73 (dd, 1H, CH_2=, *J* ≈ 17.5, 1.2 Hz), 5.28–5.24 (dd, 1H, CH_2=, *J* ≈ 10.8, 1.2 Hz), 4.53 (s, 2H, O–CH_2–Ar), 3.71–3.61 (m, 8H, –O–CH_2–CH_2–N–), 3.39 (s, 3H, OCH_3), 3.10 (s, 6H, N–CH_3).

^{13}C NMR (75 MHz, $CDCl_3$): δ/ppm 193.80, 151.65, 151.63, 137.71, 137.05, 136.50, 132.30, 127.74, 126.43, 126.38, 126.27, 113.89, 110.55, 73.09, 70.08, 67.59, 59.13, 52.18, 52.10, 39.20, 39.13.

FT-IR $(cm)^{-1}$: 2921, 1608, 1503, 1446, 1365, 1277, 1216, 1176, 1105, 919, 706.

HR-MS (m/z): $[M+H]^+$ calcd for $C_{29}H_{35}N_2O_3$ 459.2632; found 459.2642.

UV-Vis: Extinction coefficients (ε [$L \cdot mol^{-1} \cdot cm^{-1}$]) in the λ maxima ($\lambda_{1\text{-}2}$) of 2.8×10^{-5} M MeCN solution of **9**

λ_1, nm (ε): 246 (30714).

λ_2, nm (ε): 354 (27857).

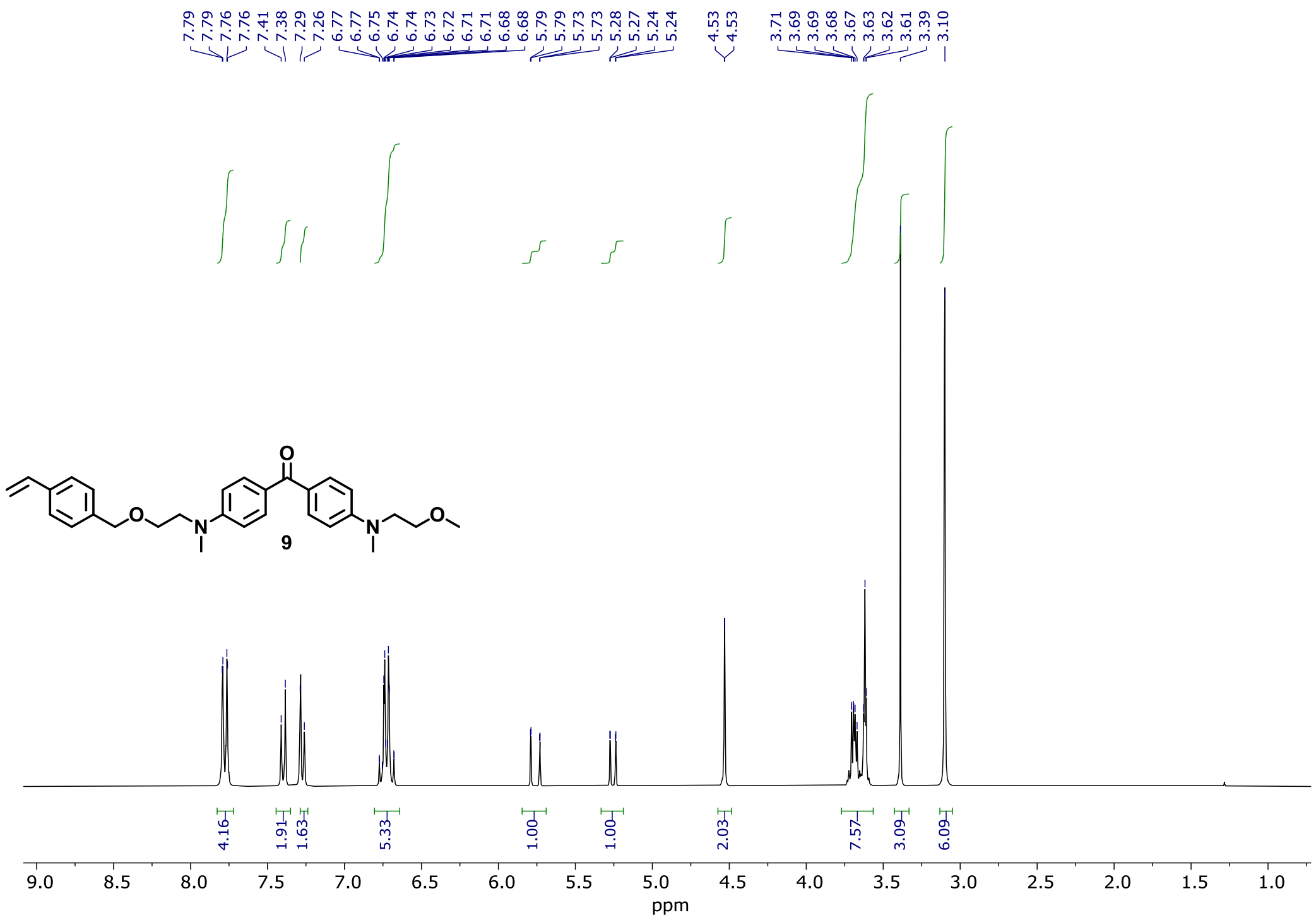

Figure S21: ^{1}H-NMR spectrum of **9** (300 MHz, $CDCl_3$, 293 K).

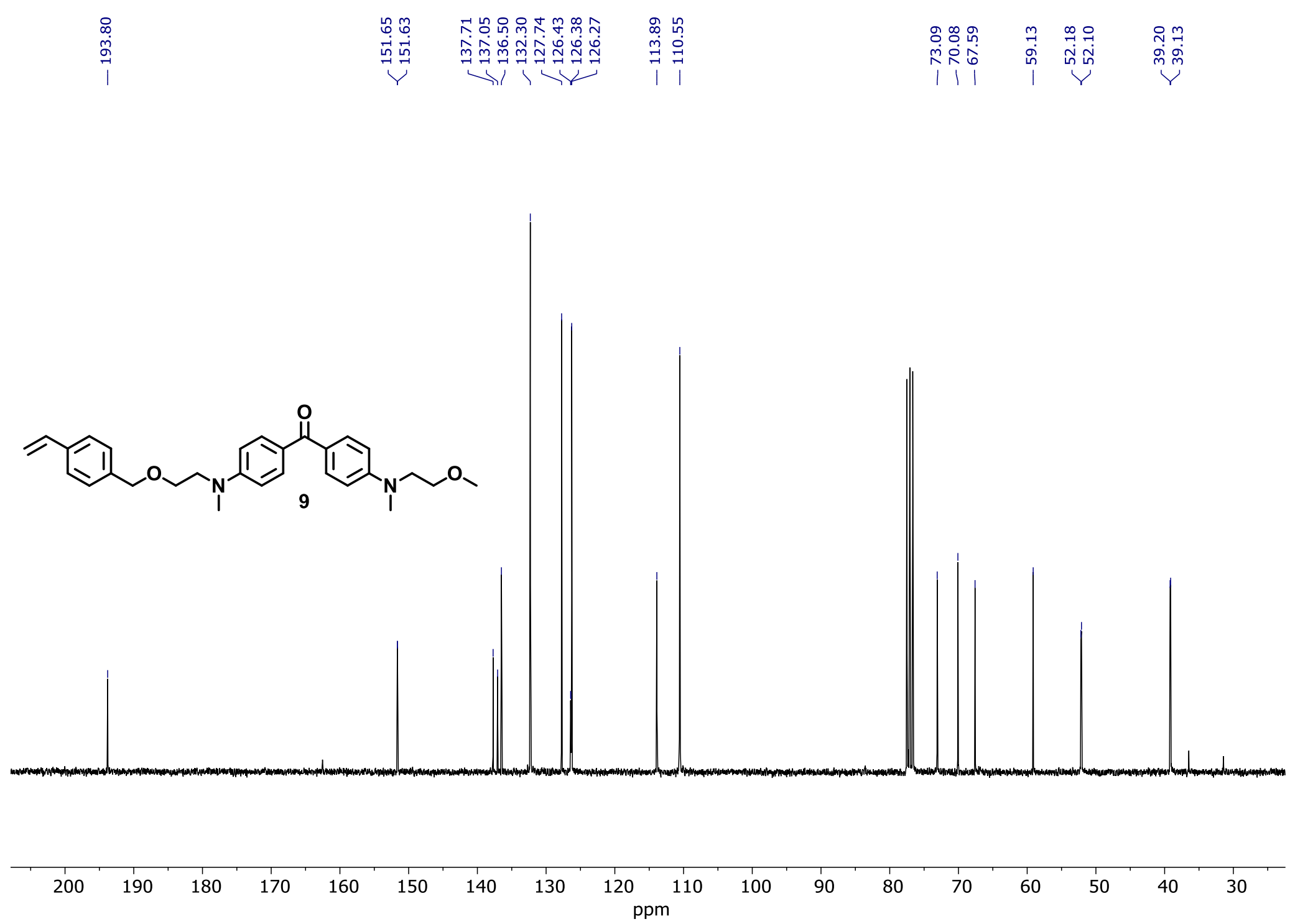

Figure S22: ^{13}C-NMR spectrum of **9** (75 MHz, $CDCl_3$, 293 K).

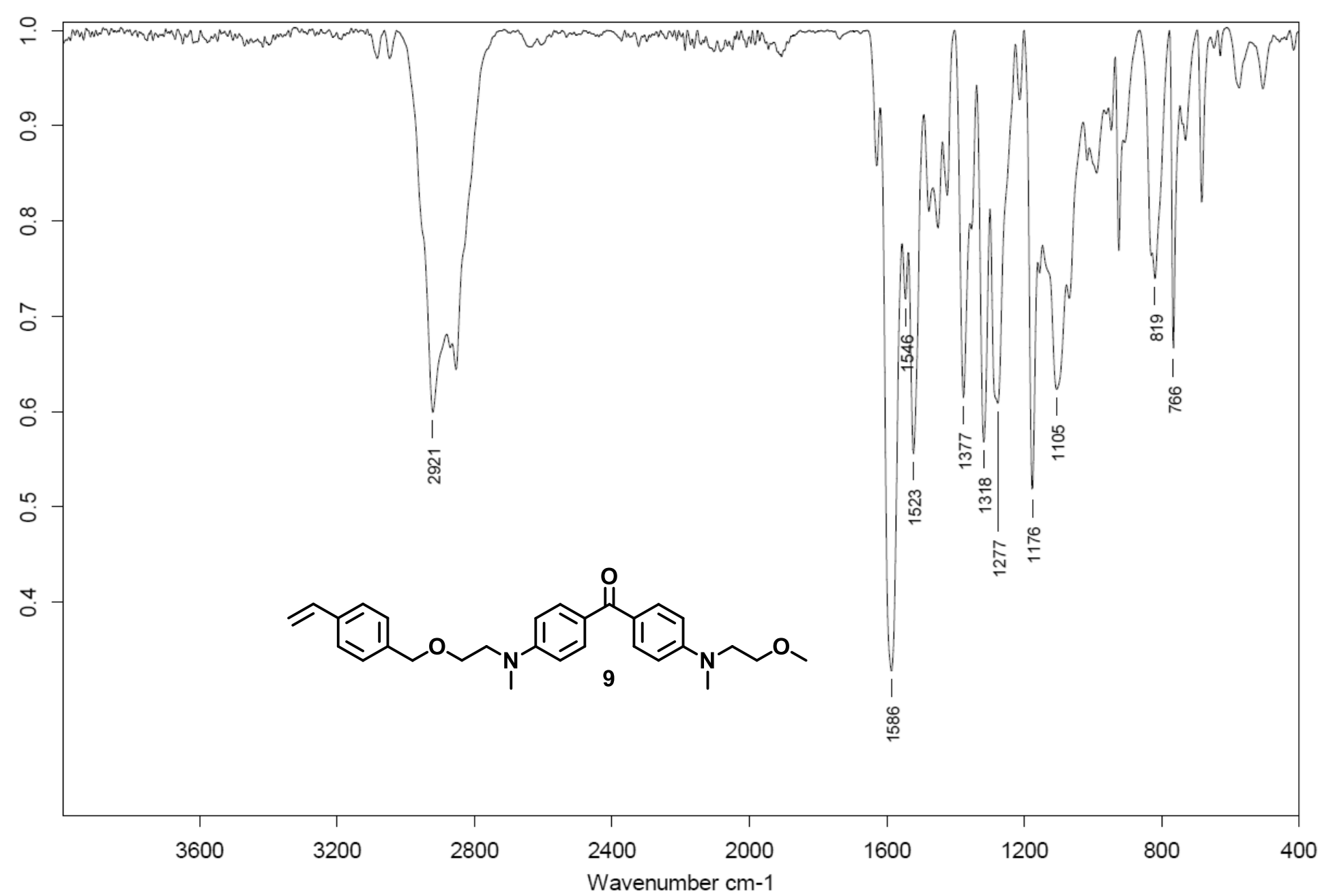

Figure S23: FT-IR spectrum of **9**

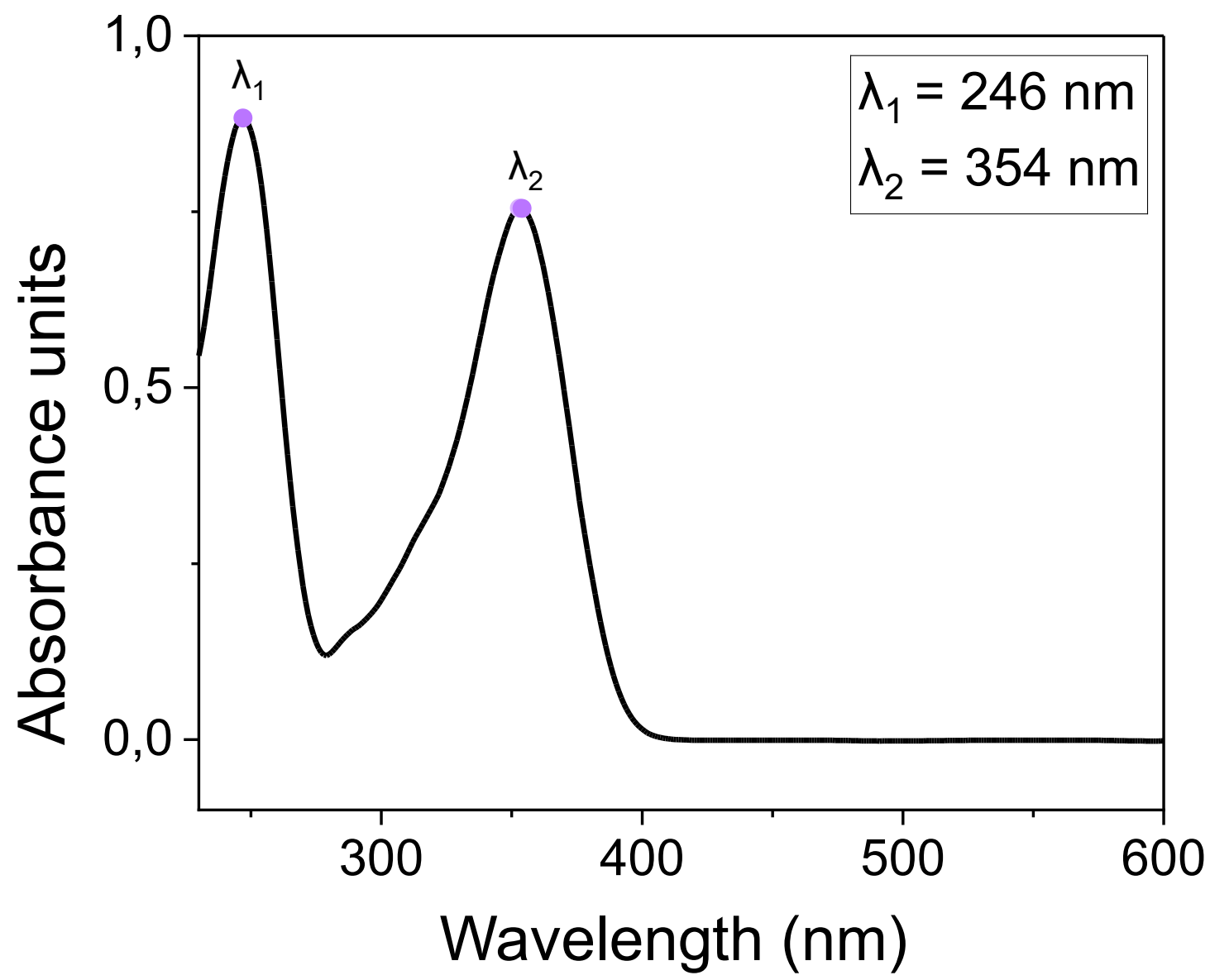

Figure S24: UV-Vis spectrum of **9** (MeCN, 2.8 × 10^{-5} M, 297 K).

(4-((2-methoxyethyl)(methyl)amino)phenyl)(4-(methyl(2-((4-vinylbenzyl)oxy)ethyl)amino)phenyl)methanol (10)

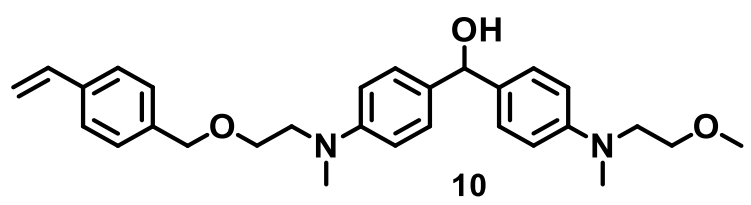

In a round-bottom flask, potassium hydroxide (8.5 mg, 1.5 $\times 10^{-4}$ mol, 0.1 eq.) was dissolved in ethanol (8 mL). **9** (0.7 g, 1.5 $\times 10^{-3}$ mol, 1 eq.) was dissolved in ethanol (4 mL) and added to the KOH solution, affording a yellow suspension. Sodium borohydride (86.6 mg, 2.3 $\times 10^{-3}$ mol, 1.5 mol eq.) was added in three equal portions (28.9 mg, 7.6 $\times 10^{-4}$ mol, 0.5 mol eq. each), and the reaction mixture was heated at reflux for 3 h after each addition. After cooling to room temperature, the reaction mixture was concentrated under reduced pressure. The residue was dissolved in dichloromethane (70 mL) and washed with water (25 mL), followed by brine (25 mL). The organic layer was dried over anhydrous Na_2SO_4, filtered, and concentrated under reduced pressure to afford the crude product. Due to the lability and sensitivity of the product to acidic conditions and light, compound **10** was employed without additional purification steps. Nevertheless, the ^{1}H and ^{13}C NMR spectra (and related assignments) of the crude product are reported below.

^{1}H NMR (300 MHz, $CDCl_3$): δ/ppm 7.40–7.22 (m, Ar–H), 6.80–6.67 (m, Ar–H including vinyl phenyl protons), 5.79 (dd, 1H, $CH{=}CH_2$), 5.73 (s, 1H, benzylic CHOH), 5.28–5.24 (dd, 1H, $CH{=}CH_2$), 4.51 (s, 2H, OCH_2Ph), 4.10–3.95 (m, 4H, NCH_2CH_2O), 3.68–3.46 (m, OCH_2, NCH_2, overlapping with impurities), 3.33 (s, 3H, OCH_3, partially overlapped), 3.0–2.9 (s, 6H, NCH_3).

^{13}C NMR (101 MHz, $CDCl_3$): δ/ppm 148.53, 137.93, 136.96, 136.57, 132.29, 132.21, 128.43, 128.01, 127.74, 127.63, 126.24, 113.77, 112.02, 111.88, 75.70, 73.02, 73.00, 70.28, 70.19, 67.74, 59.00, 52.48, 39.01.

FT-IR $(cm)^{-1}$: 3439, 2869, 1595, 1517, 1477, 1374, 1320, 1286, 1179, 1211, 1104, 989, 906, 925, 817, 767, 684, 561.

HR-MS (m/z): $[M+H]^+$ calcd for $C_{29}H_{35}N_2O_3$ 459.2632; found 459.2642.

UV-Vis: 2.6 × 10^{-5} M MeCN solution of **10** showed a λ maxima (λ_1) at 256 nm.

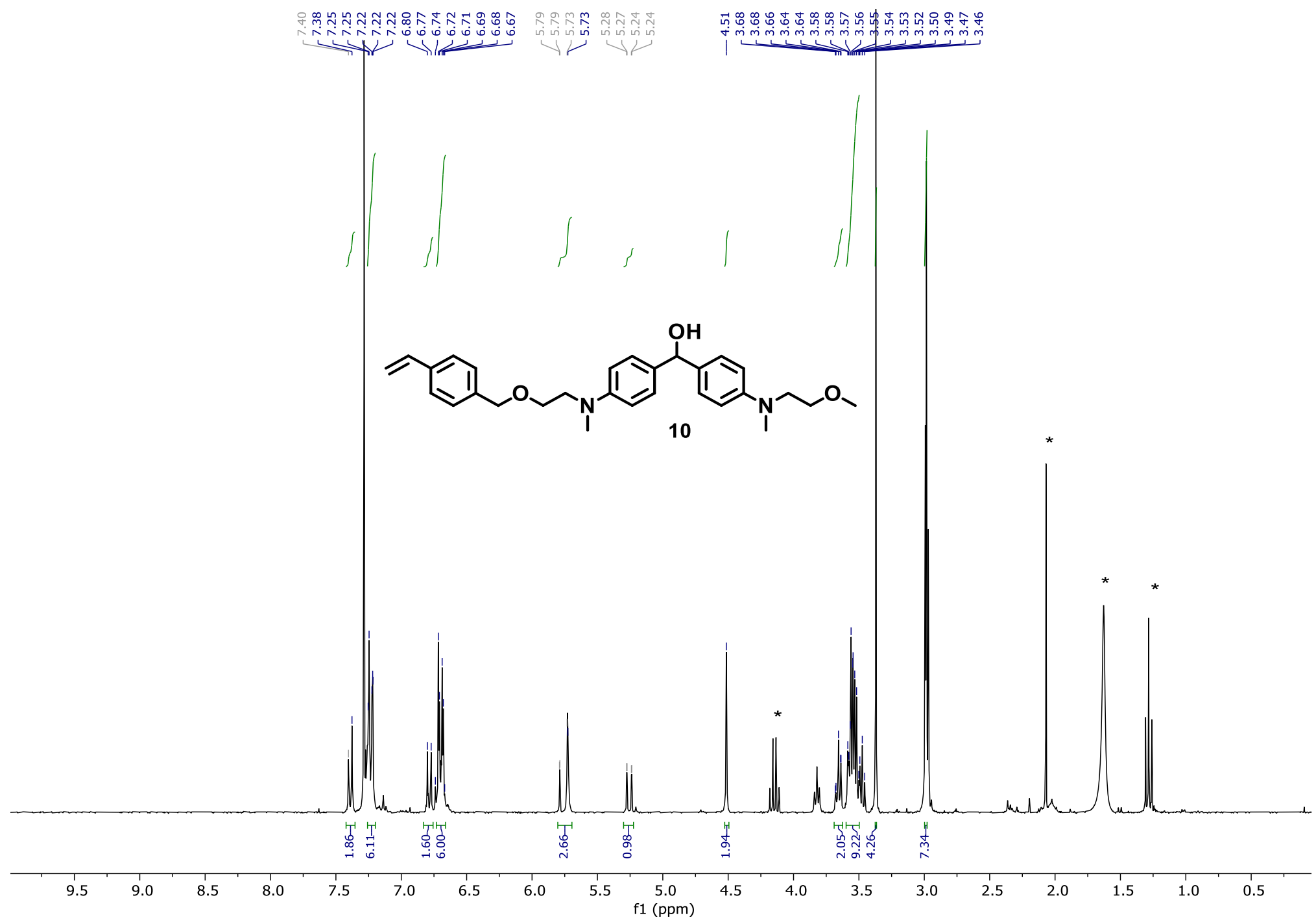

Figure S25: ^{1}H-NMR spectrum of crude **10** (300 MHz, $CDCl_3$, 293 K). Signals marked by the asterisk are related to traces of EtOAc (t, δ 1.26; s, δ 2.05; q, δ 4.12), and H_2O (brs, δ 1.58).

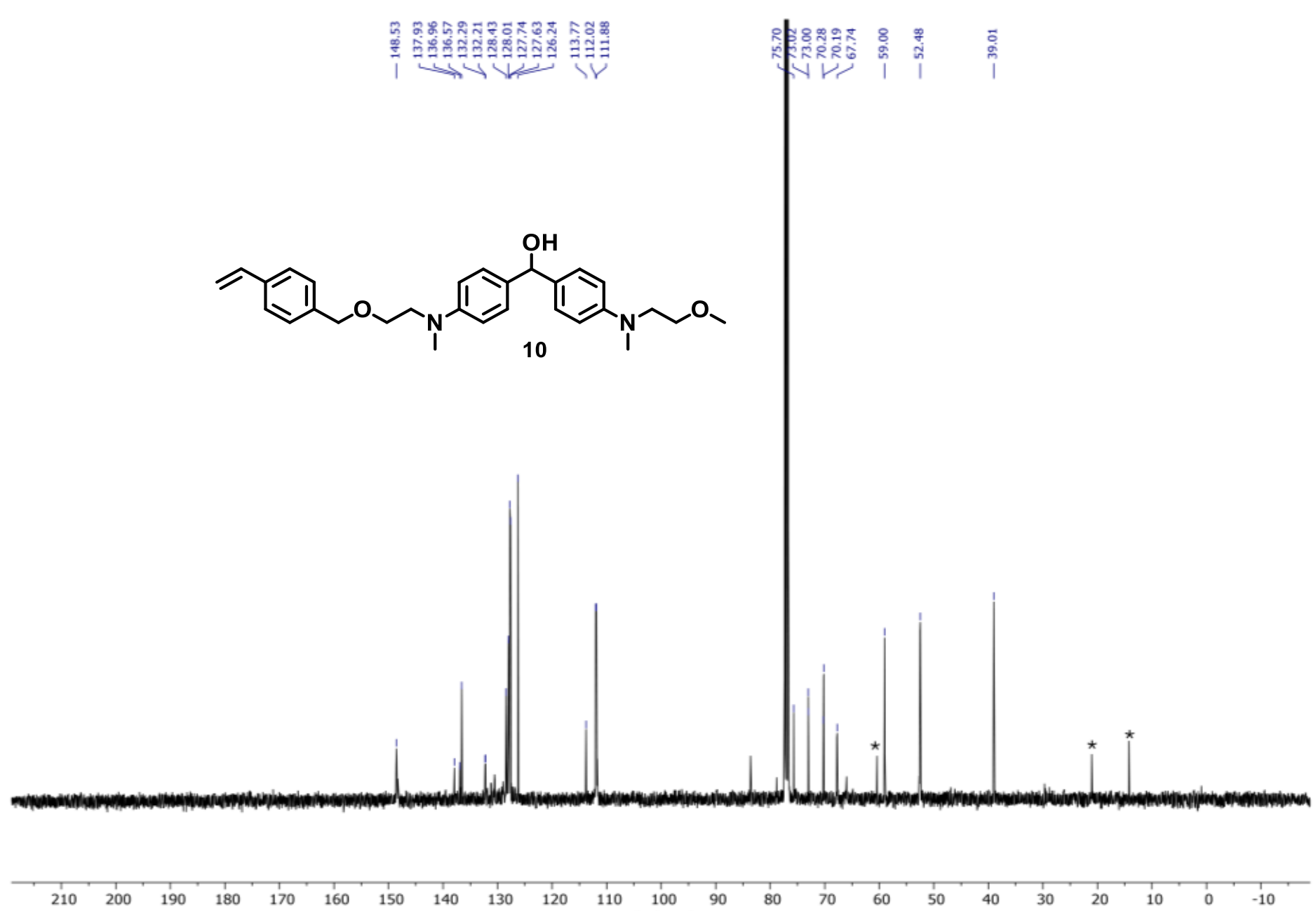

Figure S26: ^{13}C-NMR spectrum of crude **10** (75 MHz, $CDCl_3$, 293 K). Signals marked by the asterisk are related to traces of EtOAc (δ 60.5; δ 21.04; δ 14.23).

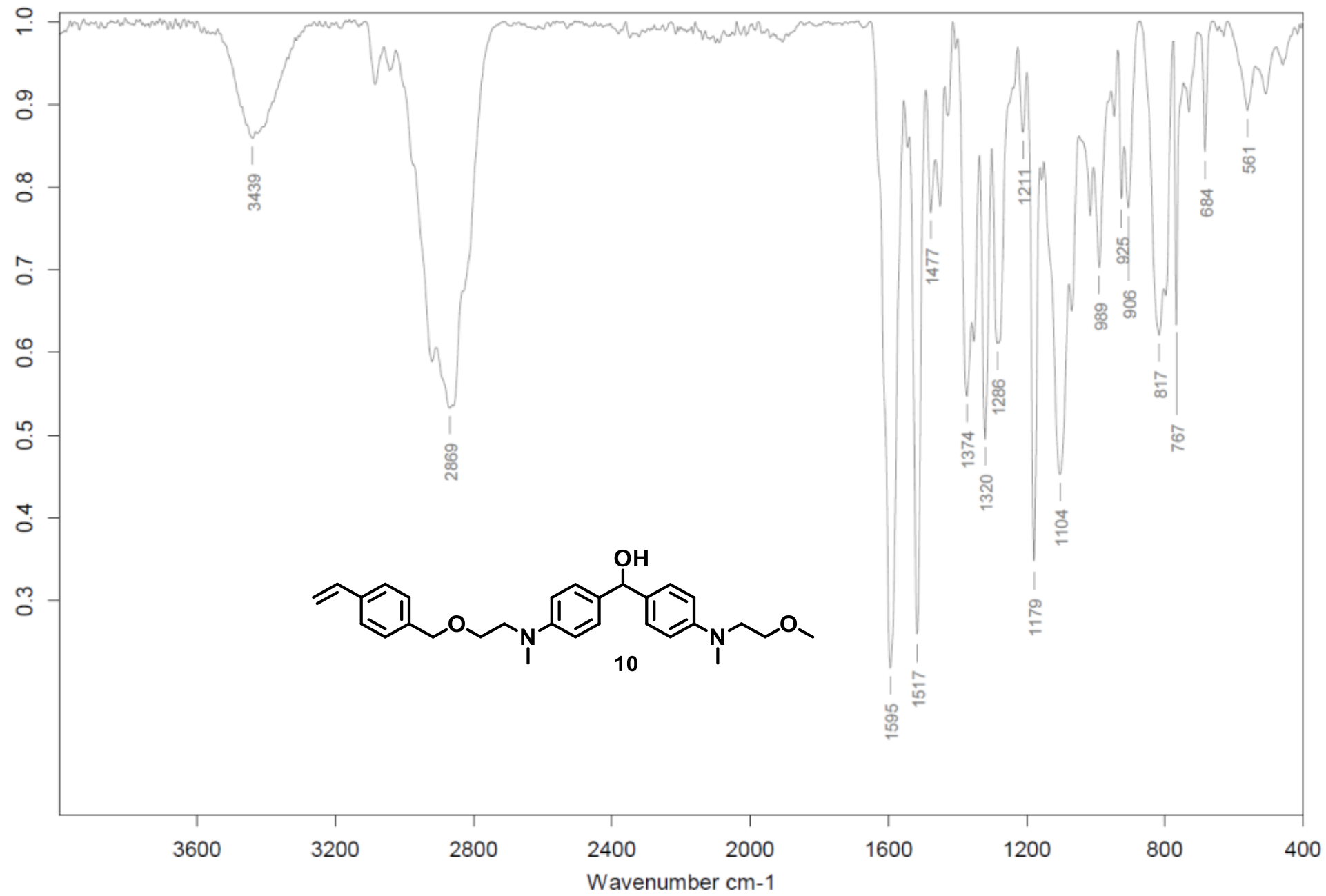

Figure S27: FT-IR spectrum of crude compound **10** (not purified).

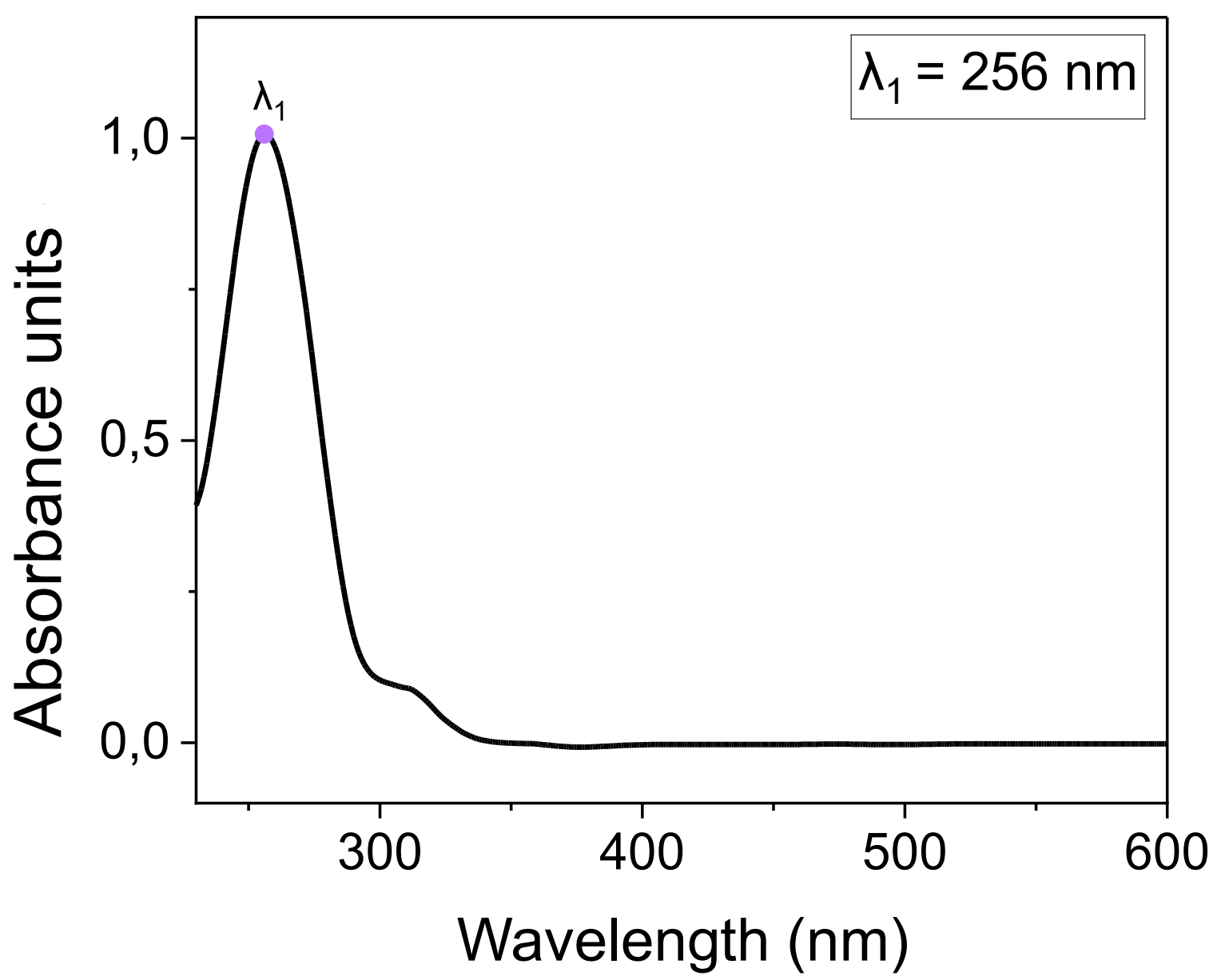

Figure S28: UV-Vis spectrum of crude compound **10** (not purified, MeCN, 297 K).

4-((4-methoxyphenyl)((4-vinylbenzyl)oxy)methyl)-N-methyl-N-(2-((4-vinylbenzyl)oxy)ethyl)aniline (Dr-1)

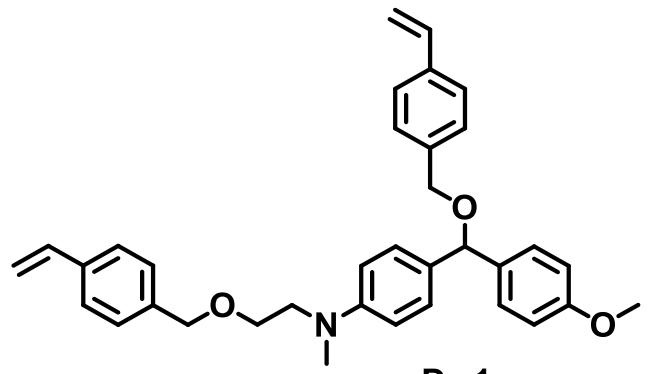

Dr-1

In a flame-dried two-neck round-bottom flask, **4** (0.6 g, 1.5 $\times10^{-3}$ mol, 1 eq.) was dissolved in dry DMF (5 mL). Potassium *tert*-butoxide (0.3 g, 2.2 $\times10^{-3}$ mol, 1.5 eq.) was added, and the reaction mixture was stirred at room temperature for 20 min. 4-vinylbenzyl chloride (1.083g/mL, 0.28 mL, 2 $\times10^{-3}$ mol, 1.5 eq.) was then added dropwise, and the reaction mixture was stirred at room temperature for 12 h. The reaction was cooled to 0 °C and quenched with distilled water (5 mL). The mixture was stirred for an additional 10 min and extracted with ethyl acetate (2 × 20 mL). The combined organic layers were washed with water (10 mL) and brine (3 × 10 mL), dried over anhydrous Na_2SO_4, filtered, and concentrated under reduced pressure. The crude product was purified by flash column chromatography (flash SiO_2, hexane/ethyl acetate gradient containing 2% triethylamine) to afford **Dr-1** as a pure product (0.6 g, yield: 72%).

^{1}H NMR (300 MHz, $CDCl_3$): δ/ppm 7.45–7.23 (m, 12H, Ar–H), 6.93–6.71 (m, 4H, Ar–H), 6.81–6.70 (dd, 2H, CH=, $J \approx 17.5$, 10.8 Hz), 5.82–5.75 (dd, 4H, CH_2=, $J \approx 17.5$, 1.2 Hz), 5.38–5.26 (s, 1H, Ar_2CH–O–), 4.55 (s, 4H, O–CH_2–Ar), 3.82 (s, 3H, OCH_3), 3.69–3.59 (m, 4H, –O–CH_2–CH_2–N–), 3.02 (s, 3H, N–CH_3).

^{13}C NMR (75 MHz, $CDCl_3$): δ/ppm 158.81, 148.66, 138.57, 137.97, 137.01, 136.80, 136.73, 136.61, 135.10, 129.80, 128.38, 128.34, 127.99, 127.79, 126.30, 126.24, 113.85, 113.72, 113.65, 111.95, 81.77, 73.05, 69.90, 67.84, 55.30, 52.55, 39.10.

FT-IR $(cm)^{-1}$: 2856, 1610, 1509, 1462, 1350, 1301, 1244, 1171, 1070, 1033, 989, 906, 824.

HR-MS (m/z): $[M+H]^+$ calcd for $C_{35}H_{38}NO_3$ 520.2852; found 520.2846.

UV-Vis: Extinction coefficients (ε [$L\cdot mol^{-1}\cdot cm^{-1}$]) in the λ maxima ($\lambda_1$) of 2.3 × 10^{-5} M MeCN solution of **Dr-1**

λ_1, nm (ε): 261 (34782).

Fluorescence spectrum: Acquired for a MeCN solution of 2.3 × 10^{-6} M irradiating at 365 nm. Fluorescence quantum yields were not determined; emission intensities are reported in arbitrary units.

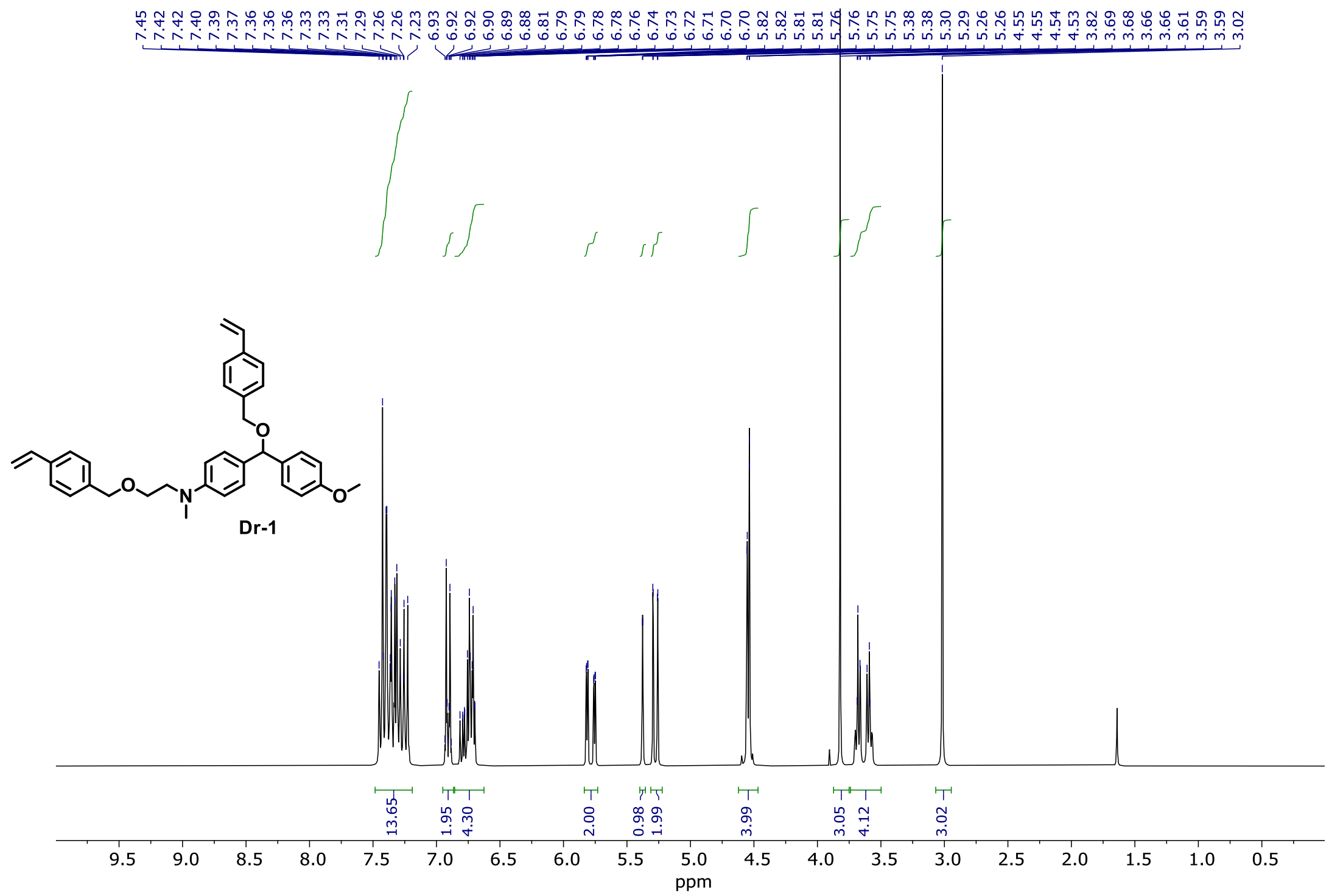

Figure S29: ^{1}H NMR spectrum of **Dr-1** (300 MHz, $CDCl_3$, 293 K).

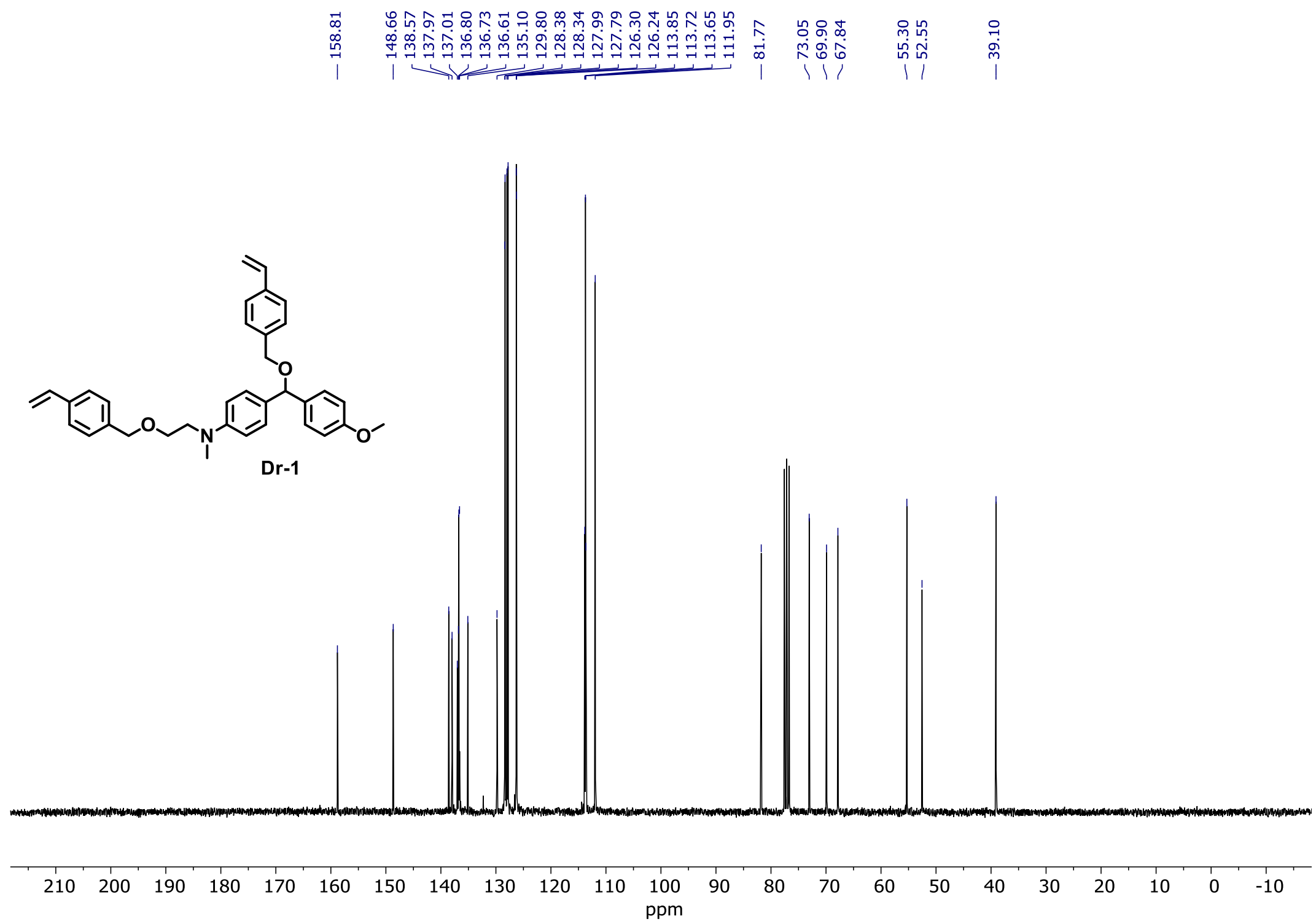

Figure S30: ^{13}C-NMR spectrum of **Dr-1** (75 MHz, $CDCl_3$, 293 K).

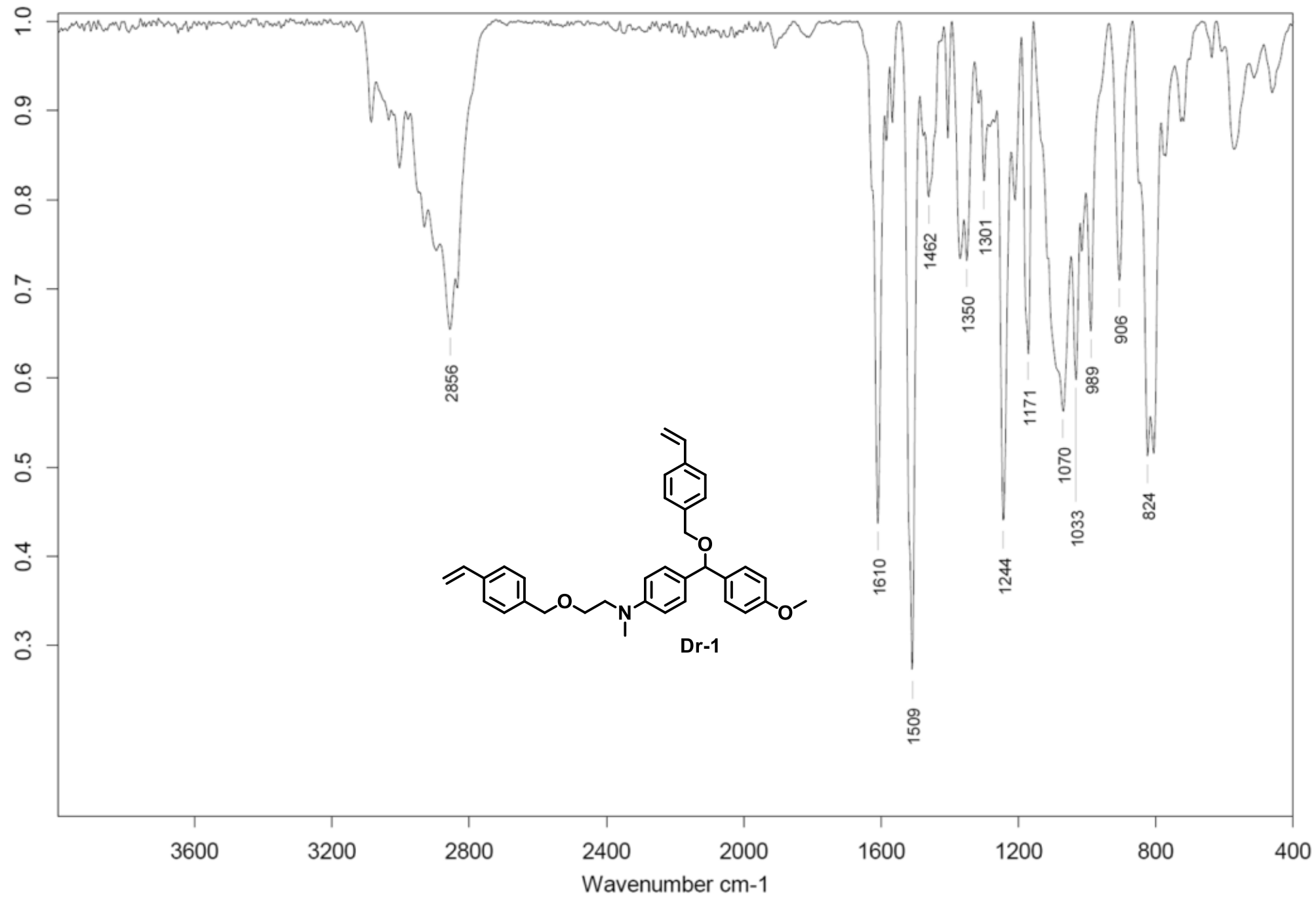

Figure S31: FT-IR spectrum of **Dr-1**

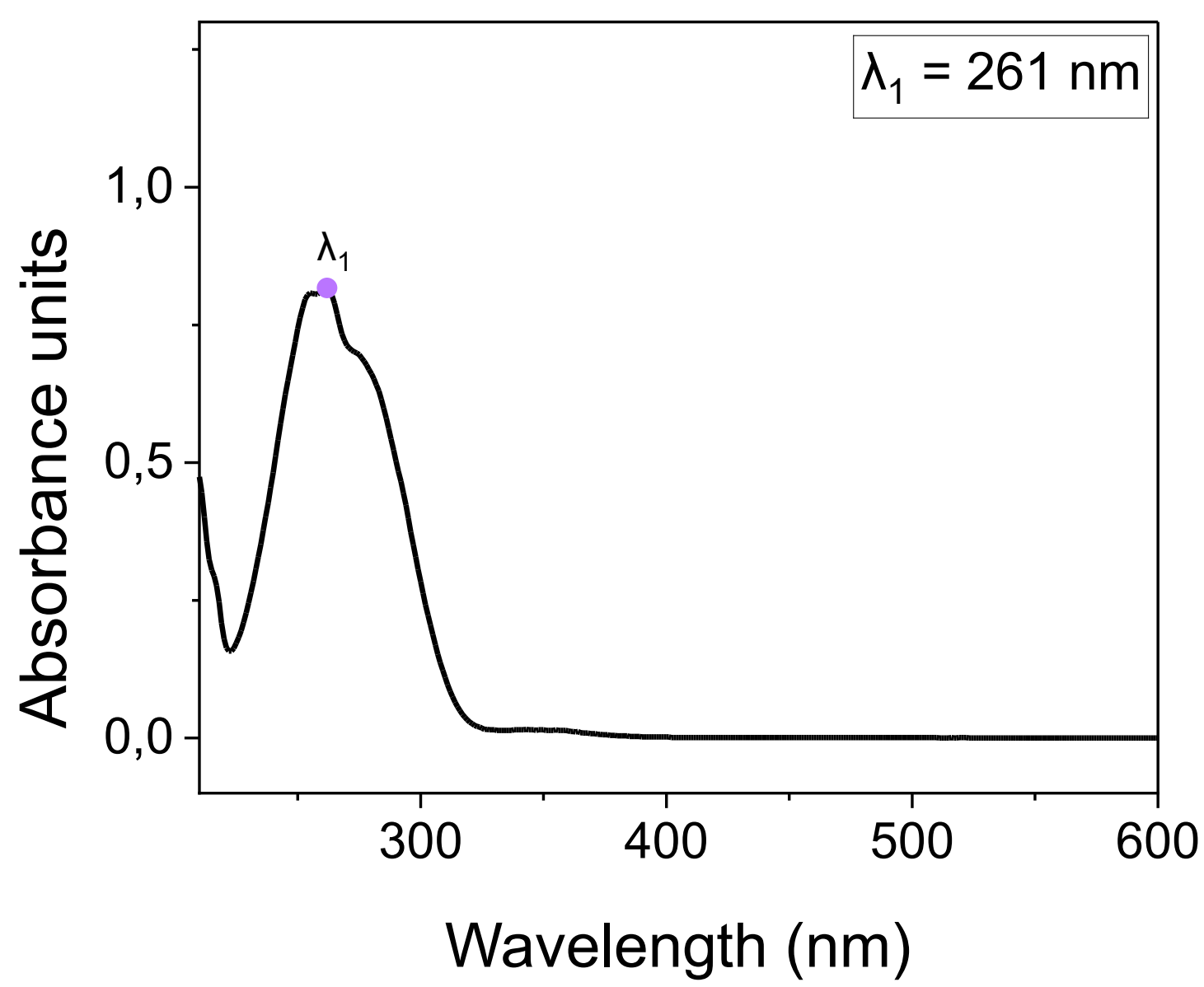

Figure S32: UV-Vis spectrum of **Dr-1** (MeCN, 2.3 × 10^{-5} M, 297 K).

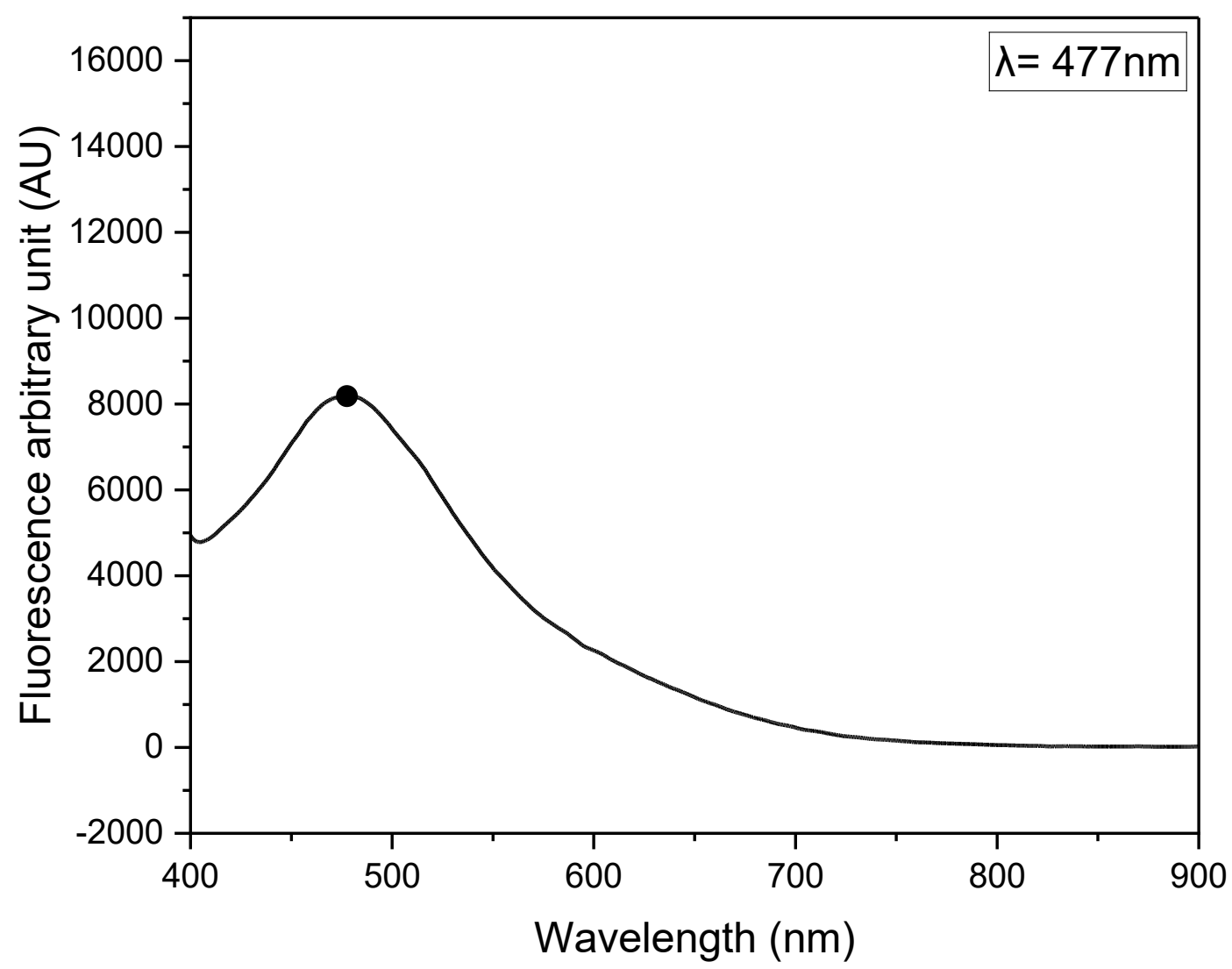

Figure S33: Fluorescence spectrum of **Dr-1** (MeCN, 2.3 × 10^{-6} M, 297 K) excited at 365 nm.

2-(4-vinylbenzyl)malononitrile (5)

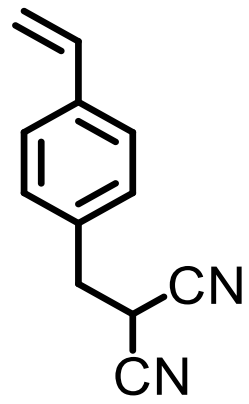

5

In a flame-dried round-bottom flask, malononitrile (1.1 g, 1.6 ×10^{-2} mol, 1 eq.) and 1-(chloromethyl)-4-vinylbenzene (1.9 g, 1.3 ×10^{-2} mol, 0.8 eq.) were dissolved in dry THF (20 mL) under stirring at room temperature. Sodium hydride (0.6 g, 1.6 ×10^{-2} mol, 1 eq., 60% dispersion in mineral oil) was added in small portions over 5 min. The reaction mixture was heated to 65 °C for 4 h, while stirring. The reaction mixture was cooled to room temperature and quenched with water (15 mL). The aqueous phase was extracted with dichloromethane (3 × 20 mL). The combined organic layers were dried over anhydrous Na_2SO_4, filtered, and concentrated under reduced pressure. The crude product was purified by column chromatography to afford compound **12** (1,16 g, yield: 50%).

^{1}H NMR (300 MHz, $CDCl_3$): δ/ppm 7.48–7.29 (m, 4H, Ar–H), 6.79–6.69 (dd, 1H, CH=, *J* ≈ 17.5, 10.8 Hz), 5.84–5.77 (dd, 1H, CH_2=, *J* ≈ 17.5, 1.2 Hz), 5.35–5.31 (dd, 1H, CH_2=, *J* ≈ 10.8, 1.2 Hz), 3.95–3.90 (s, 1H, $CH(CN)_2$), 3.32–3.29 (s, 2H, Ar–CH_2–).

^{13}C NMR (75 MHz, $CDCl_3$): δ/ppm 138.20, 135.95, 132.25, 129.37, 127.07, 115.05, 112.12, 36.49, 24.97.

FT-IR $(cm)^{-1}$: 3087, 3054, 3007, 2926, 2257, 1826, 1628, 1513, 1450, 1408, 1426, 1256, 1284, 1163, 1113, 1021, 995, 912, 849, 835, 801, 731, 564, 497, 468.

HR-MS (m/z): $[M+Na]^+$ calcd for $C_{12}H_{10}N_2Na$ 205.0736; found 205.0736

UV-Vis: Extinction coefficients (ε [L·mol^{-1}·cm^{-1}]) in the λ maxima ($λ_1$) of 8.6 × 10^{-5} M MeCN solution of **5.**

$λ_1$, nm (ε): 261 (17441).

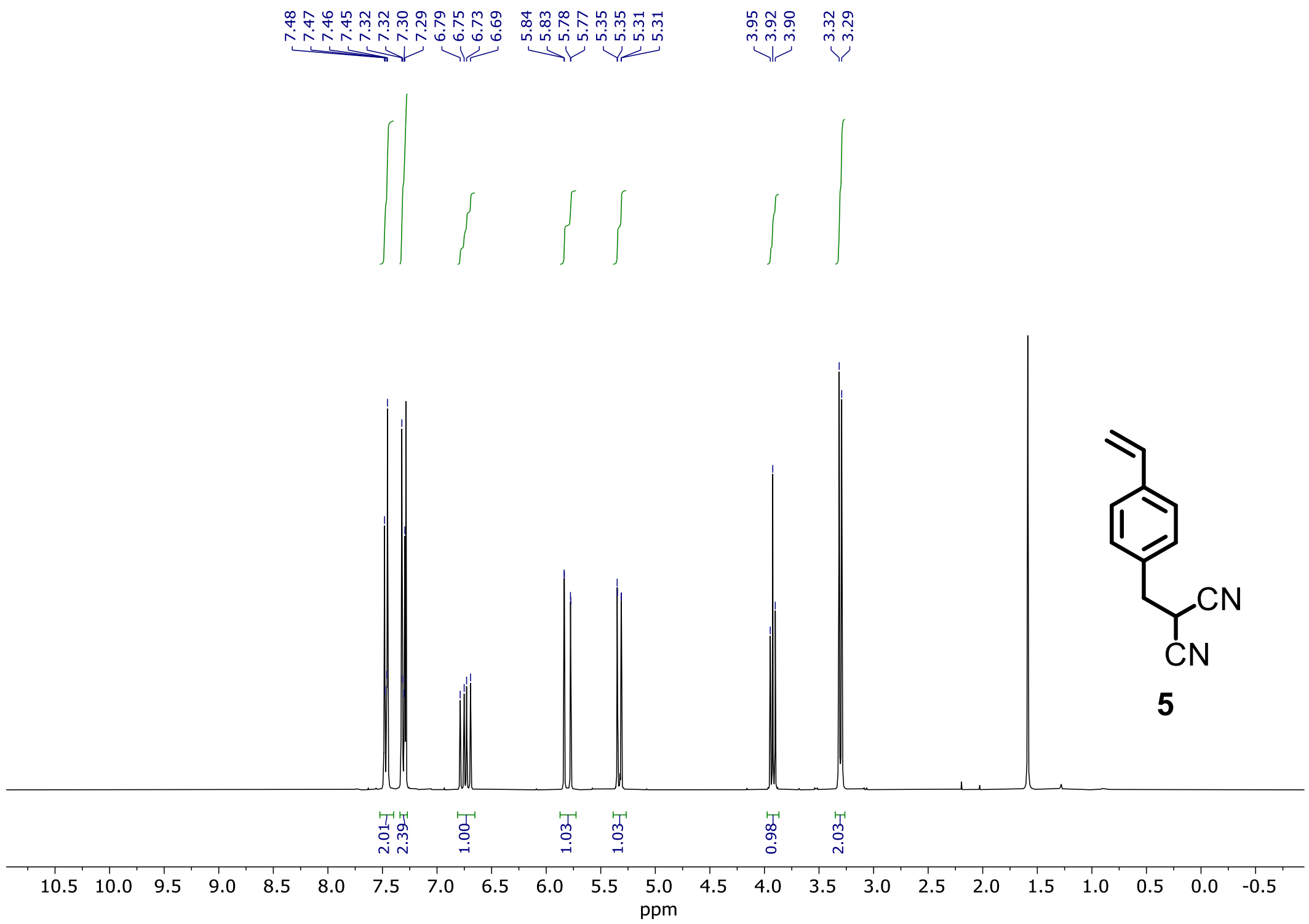

Figure S34: ^{1}H-NMR spectrum of **5** (300 MHz, $CDCl_3$, 293 K).

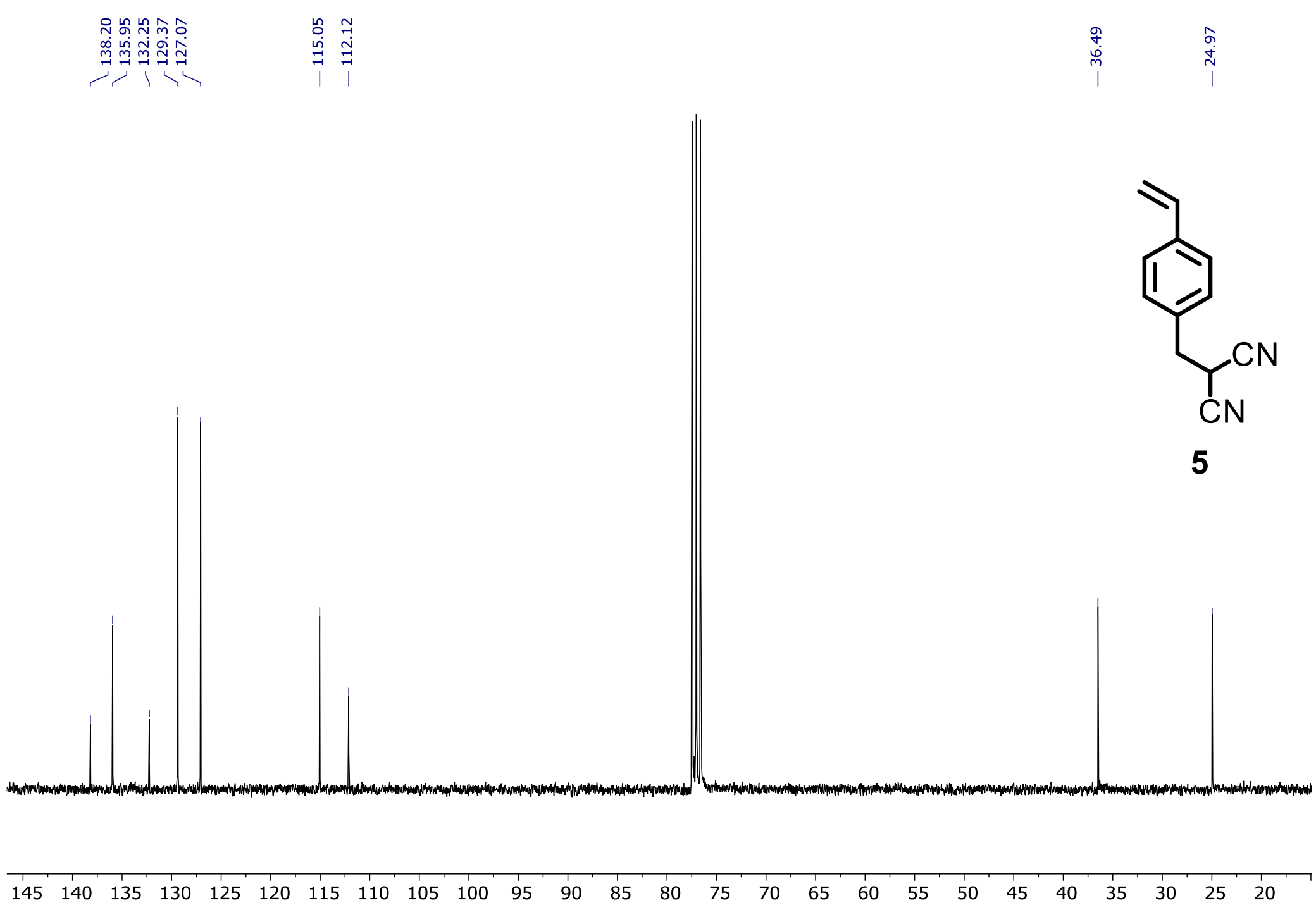

Figure S35: ^{13}C-NMR spectrum of **5** (75 MHz, $CDCl_3$, 293 K).

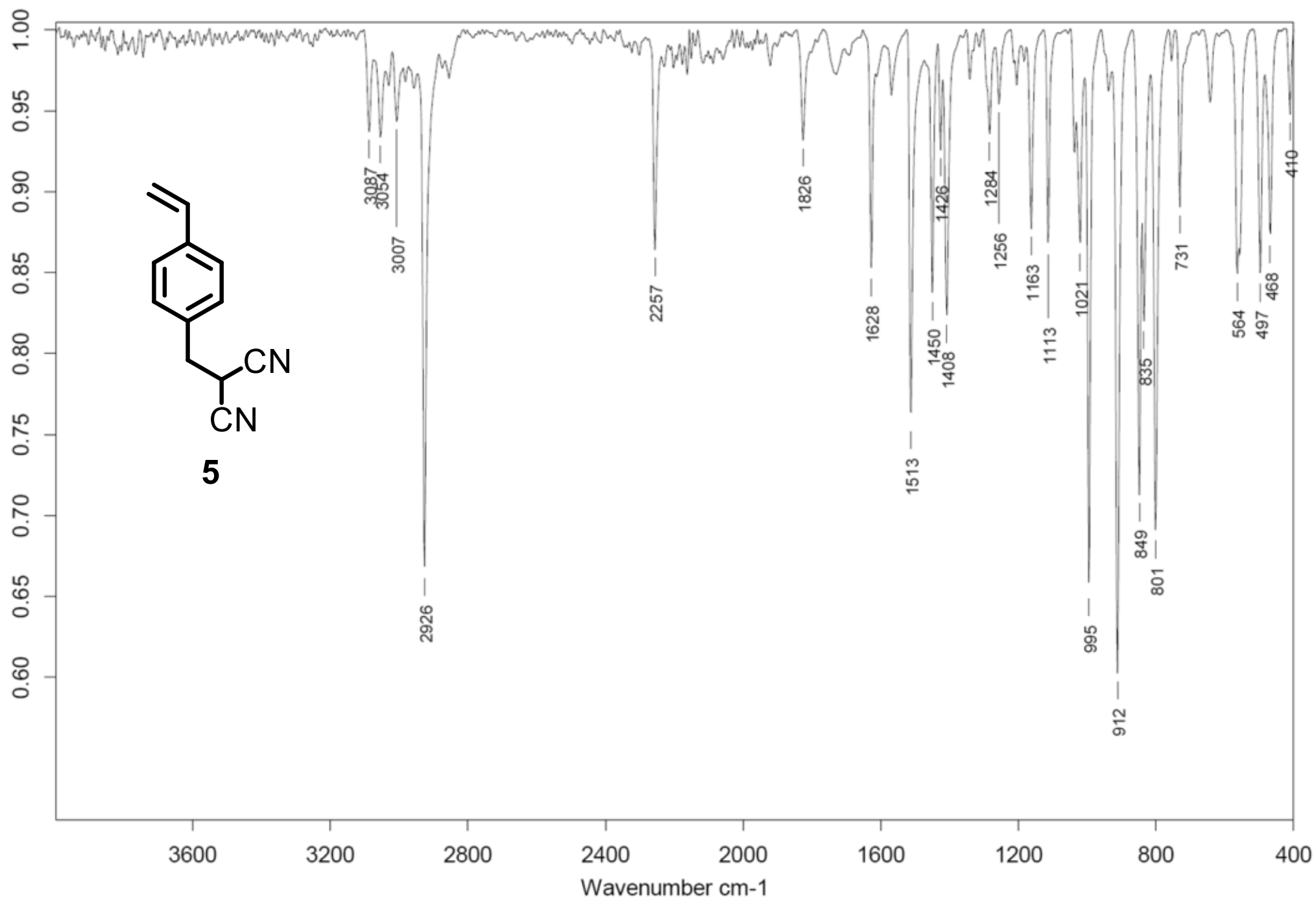

Figure S36: FT-IR spectrum of **5**

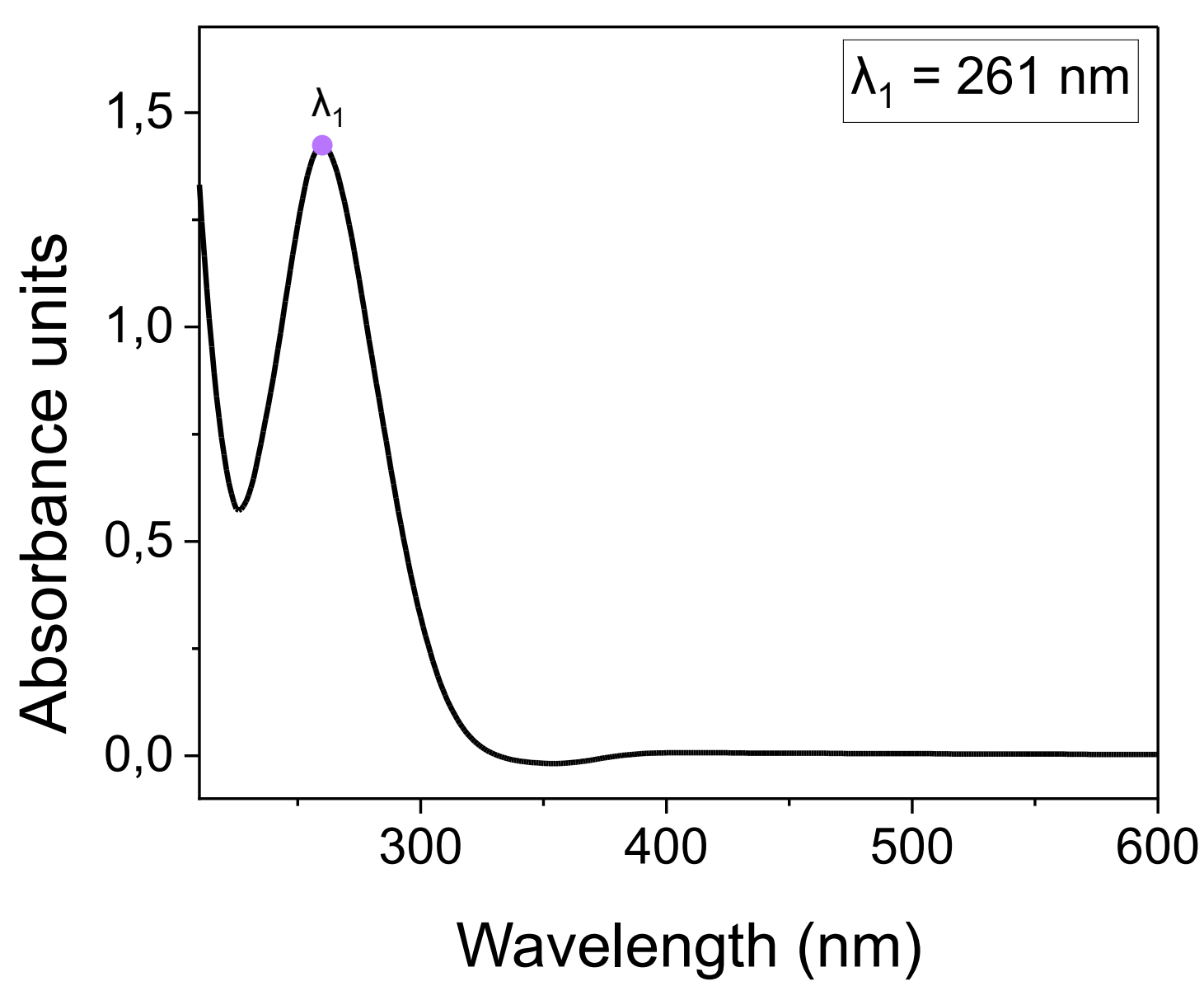

Figure S37: UV-Vis spectrum of **5** (MeCN, 8.6 × 10^{-5} M, 297 K).

2-((4-methoxyphenyl)(4-(methyl(2-((4-vinylbenzyl)oxy)ethyl)amino)phenyl)methyl)-2-(4-vinylbenzyl)malononitrile (Dr-2)

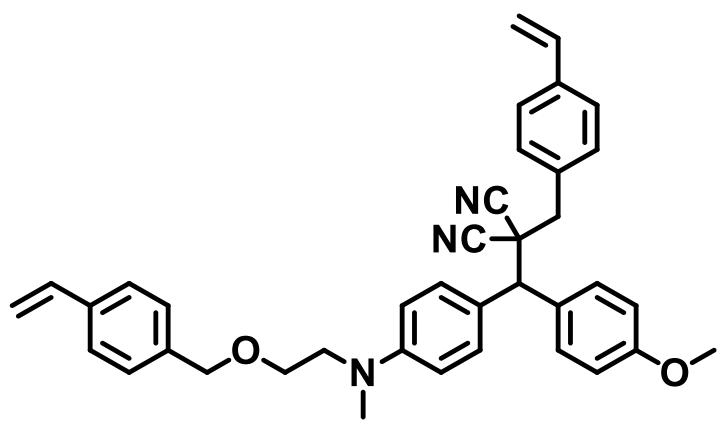

Dr-2

In a round-bottom flask, sodium hydride (20.4 mg, 0.5 $\times 10^{-3}$ mol, 1.1 eq., 60% dispersion in mineral oil) was suspended in dry DMF (2 mL) at room temperature under stirring. Compound **5** (84.7 mg, 0.5 $\times 10^{-3}$ mol, 1.1 eq.) was added in small portions under a positive argon flow, and the reaction mixture was stirred for 10 min until a homogeneous solution was obtained. A solution of **4** (0.2 g, 0.37 $\times 10^{-3}$ mol, 1 eq.) in dry DMF (2 mL) was added dropwise under stirring. Subsequently, boron trifluoride diethyl etherate (46%, 0.1 mL, 0.9 $\times 10^{-3}$ mol, 2.5 eq.) was added slowly via syringe. The reaction mixture was stirred at room temperature for 1 h. The solvent was removed under reduced pressure. The crude product was purified by flash column chromatography (flash SiO_2, hexane/ethyl acetate gradient containing 2% triethylamine) to afford **Dr-2** (63 mg, yield: 38%).

^{1}H NMR (300 MHz, $CDCl_3$): δ/ppm 7.54–6.67 (m, 16H, Ar–H), 6.78–6.69 (dd, 2H, CH=, *J* ≈ 17.5, 10.8 Hz), 5.82–5.72 (dd, 2H, CH_2=, *J* ≈ 17.5, 1.2 Hz), 5.30 (s, 1H, $Ar_2CH–C(CN)_2$), 5.32–5.23 (dd, 2H, CH_2=, *J* ≈ 10.8, 1.2 Hz), 4.52 (s, 2H, O–CH_2–Ar), 4.22 (s, 2H, Ar–CH_2–$C(CN)_2$), 3.83 (s, 3H, OCH_3), 3.70–3.56 (m, 4H, –O–CH_2–CH_2–N–), 3.17 (s, 2H, Ar–CH_2–$C(CN)_2$), 3.02 (s, 3H, N–CH_3).

^{13}C NMR (75 MHz, $CDCl_3$): δ/ppm 159.39, 137.86, 137.75, 137.02, 136.51, 136.22, 131.84, 130.60, 130.06, 130.03, 129.84, 129.80, 127.75, 126.55, 126.47, 126.26, 115.49, 115.46, 115.29, 114.63, 114.41, 114.19, 113.84, 73.05, 67.60, 56.48, 55.29, 45.32, 43.02.

FT-IR $(cm)^{-1}$: 2925, 1673, 1609, 1377, 1407, 1251, 1181, 1210, 1100, 1030, 990, 909, 826, 771, 577.

HR-MS (m/z): $[M+H]^+$ calcd for $C_{38}H_{38}N_3O_2$ 568.2947; found 568.2959

UV-Vis: Extinction coefficients (ε [$L\cdot mol^{-1}\cdot cm^{-1}$]) in the λ maxima ($\lambda_1$) of 2.5 × 10^{-5} M MeCN solution of **10**

λ_1, nm (ε): 259 (38456).

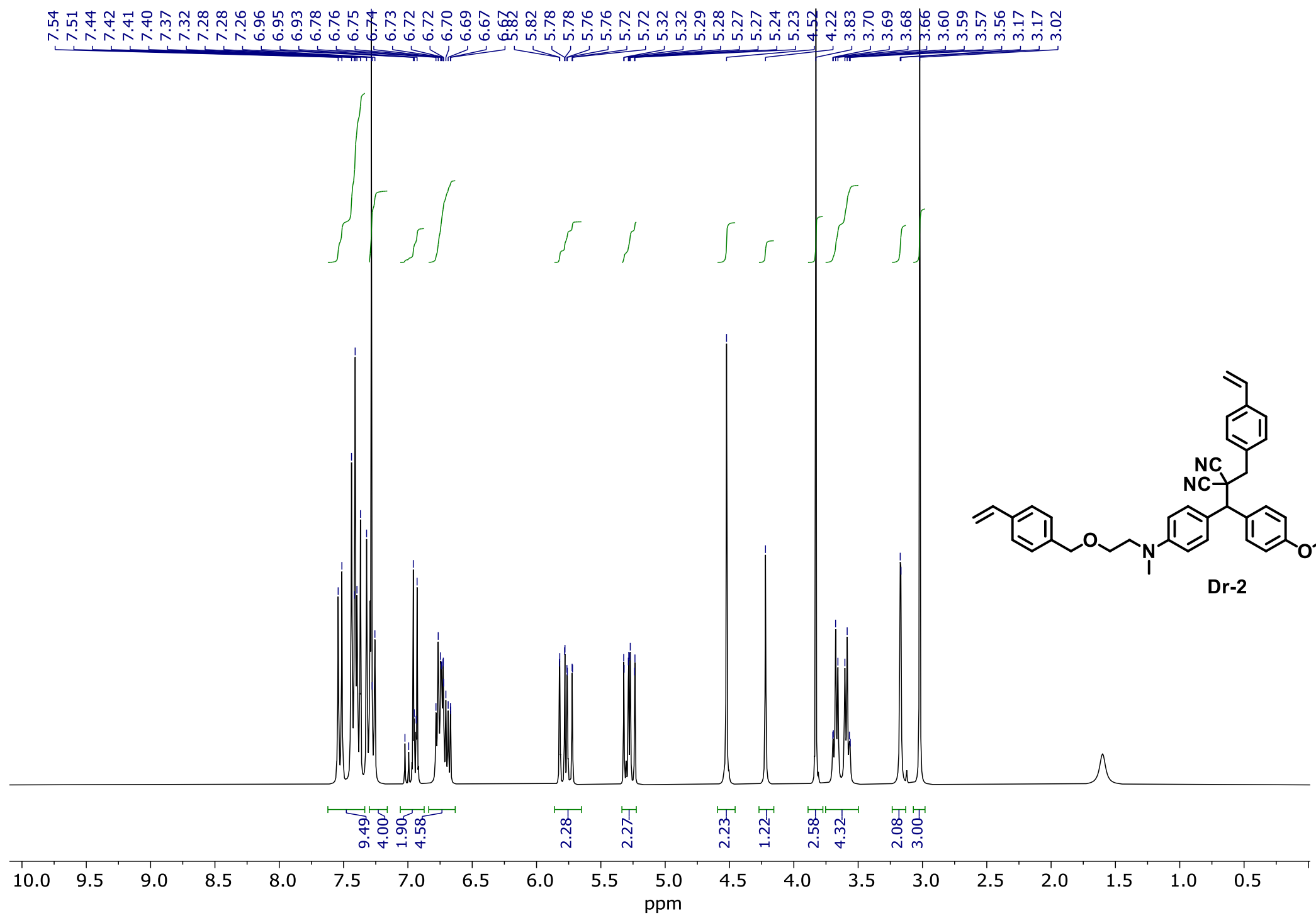

Figure S38: ^{1}H-NMR spectrum of **Dr-2** (300 MHz, $CDCl_3$, 293 K).

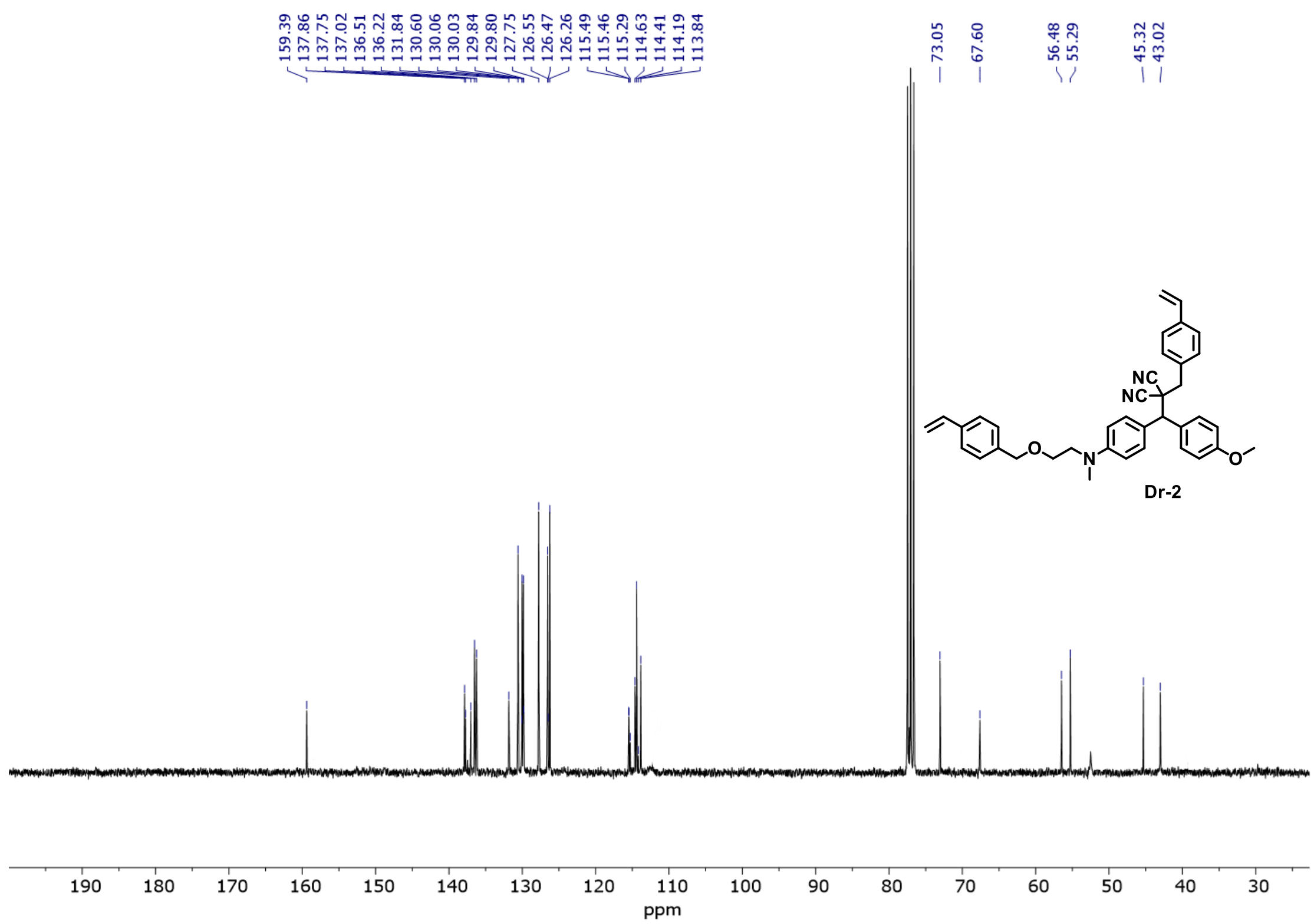

Figure S39: ^{13}C-NMR spectrum of **Dr-2** (75 MHz, $CDCl_3$, 293 K).

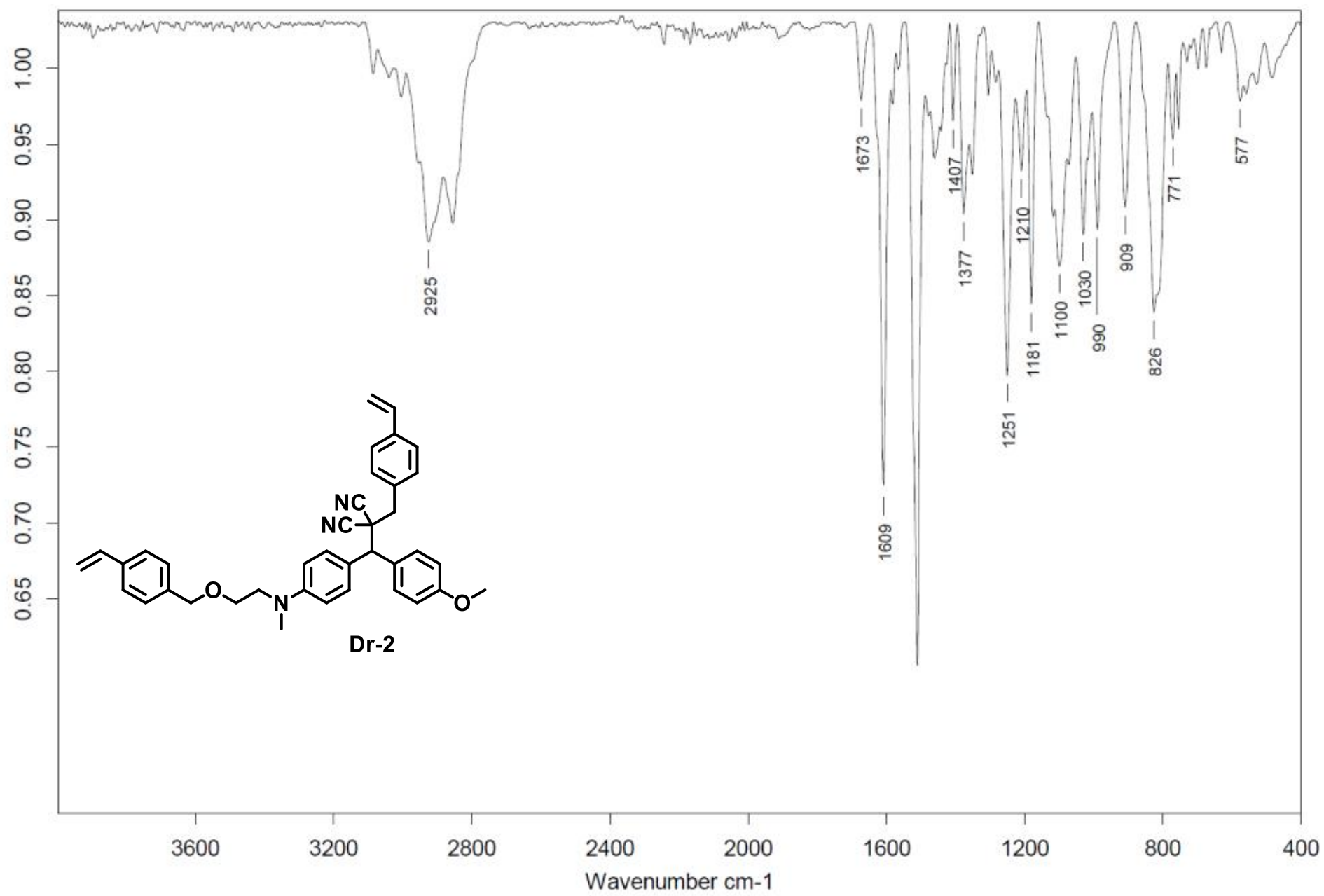

Figure S40: FT-IR spectrum of **Dr-2**

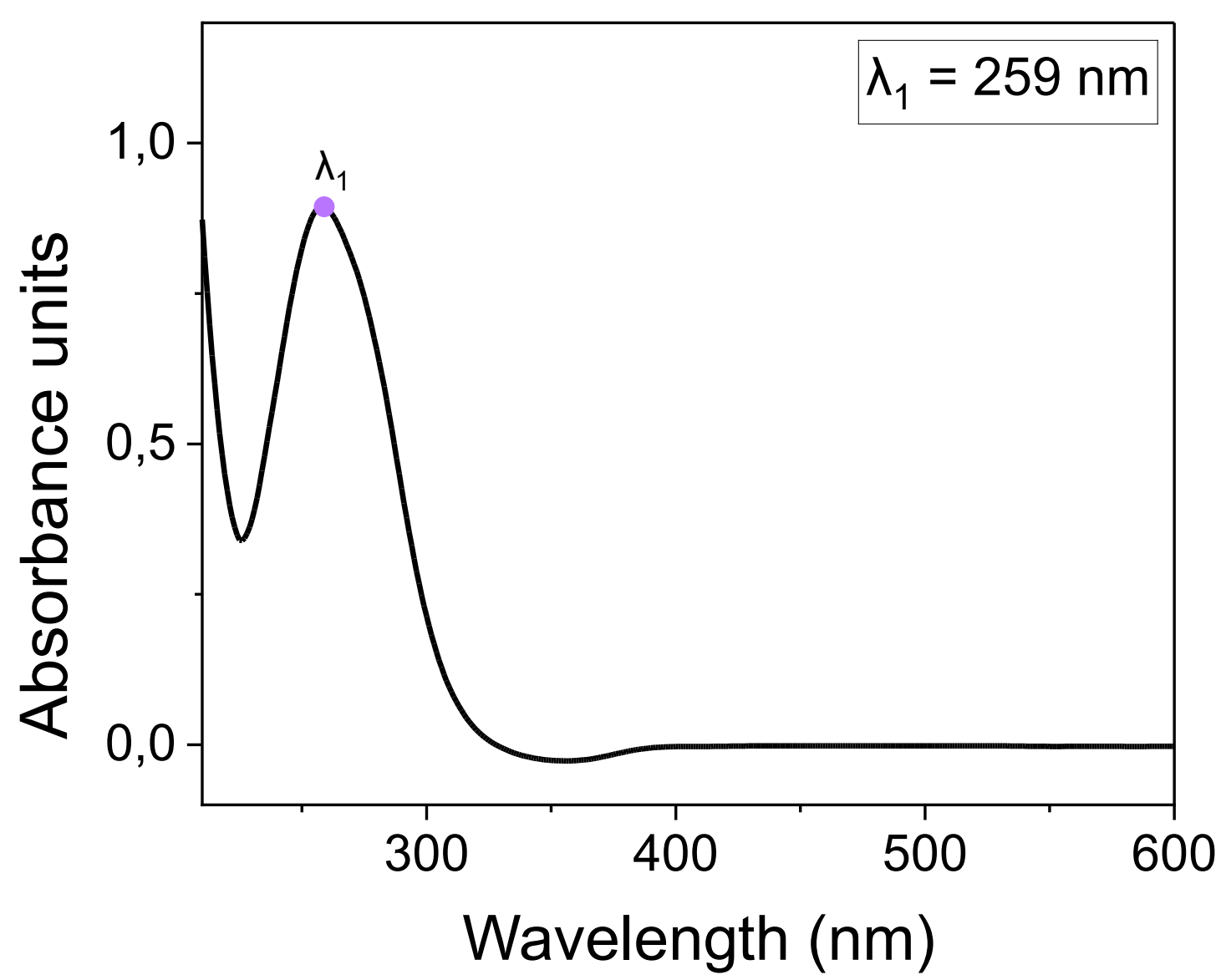

Figure S41: UV-Vis spectrum of **Dr-2** (MeCN, 2.5 × 10^{-5} M, 297 K).

N-(2-methoxyethyl)-N-methyl-4-((4-(methyl(2-((4-vinylbenzyl)oxy)ethyl)amino)phenyl)((4-vinylbenzyl)oxy)methyl)aniline (Dr-3)

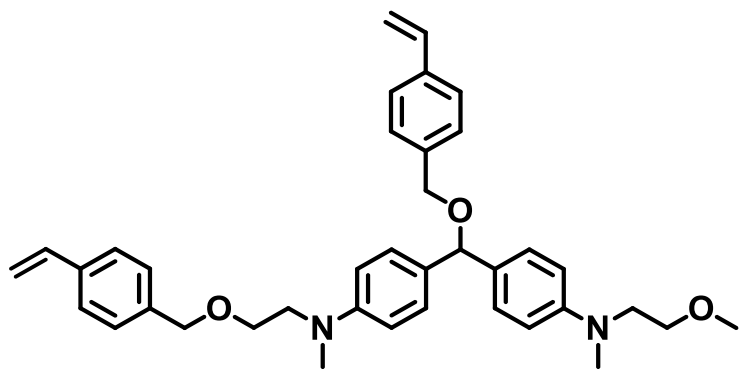

Dr-3

In a flame-dried two-neck round-bottom flask, **10** (70 mg, 0.15 $\times 10^{-3}$ mol, 1 eq.) was dissolved in dry DMF (2 mL) at room temperature under stirring. Potassium *tert*-butoxide (26 mg, 0.23 $\times$ 10^{-3} mol, 1.5 eq.) was added, and the reaction mixture was stirred for 20 min. 1-(Chloromethyl)-4-vinylbenzene (35 mg, 0.23 $\times 10^{-3}$ mol, 1.5 eq.) was then added dropwise, and the reaction mixture was stirred at room temperature for 12 h. The reaction mixture was cooled to 0 °C and quenched with distilled water (2 mL). After stirring for an additional 10 min, the aqueous phase was extracted with ethyl acetate (2 × 5 mL). The combined organic layers were washed with water (5 mL) and brine (3 × 5 mL), dried over anhydrous Na_2SO_4, filtered, and concentrated under reduced pressure. The crude product was purified by flash column chromatography (flash SiO_2, hexane/ethyl acetate gradient containing 2% triethylamine) to afford the title compound as a pure product (44 mg, yield: 42%).

^{1}H NMR: (300 MHz, $CDCl_3$): δ/ppm 7.42–7.20 (m, 16H, Ar–H), 6.79–6.68 (dd, 2H, CH=), 5.79–5.72 (dd, 2H, CH_2=), 5.31–5.23 (dd, 2H, CH_2=), 4.52 (s, 4H, O–CH_2–Ar), 3.69–3.56 (m, 8H, –O–CH_2–CH_2–N–), 3.37 (s, 3H, OCH_3), 2.99 (s, 3H, N–CH_3), 2.98 (s, 3H, N–CH_3).

^{13}C NMR (75 MHz, $CDCl_3$): δ/ppm 148.53, 148.46, 138.78, 137.92, 136.96, 136.72, 136.56, 130.38, 130.28, 128.25, 127.91, 127.75, 127.63, 127.21, 126.43, 126.24, 126.12, 113.77, 113.47, 111.93, 111.89, 81.89, 70.21, 69.73, 67.79, 59.03, 52.50, 39.07, 39.01.

FT-IR $(cm)^{-1}$: 3086, 2858, 1723, 1611, 1568, 1451, 1406, 1367, 1319, 1276, 1183, 1210, 1152, 1070, 989, 1016, 906, 825, 718, 568, 458.

HR-MS (m/z): $[M+H]^+$ calcd for $C_{38}H_{45}N_2O_3$ 577.3422; found 577.3425

UV-**Vis**: Extinction coefficients (ε [$L \cdot mol^{-1} \cdot cm^{-1}$]) in the λ maxima ($\lambda_I$) of 1.4 × 10^{-5} M MeCN solution of **Dr-3**

λ_I, nm (ε): 256 (39654).

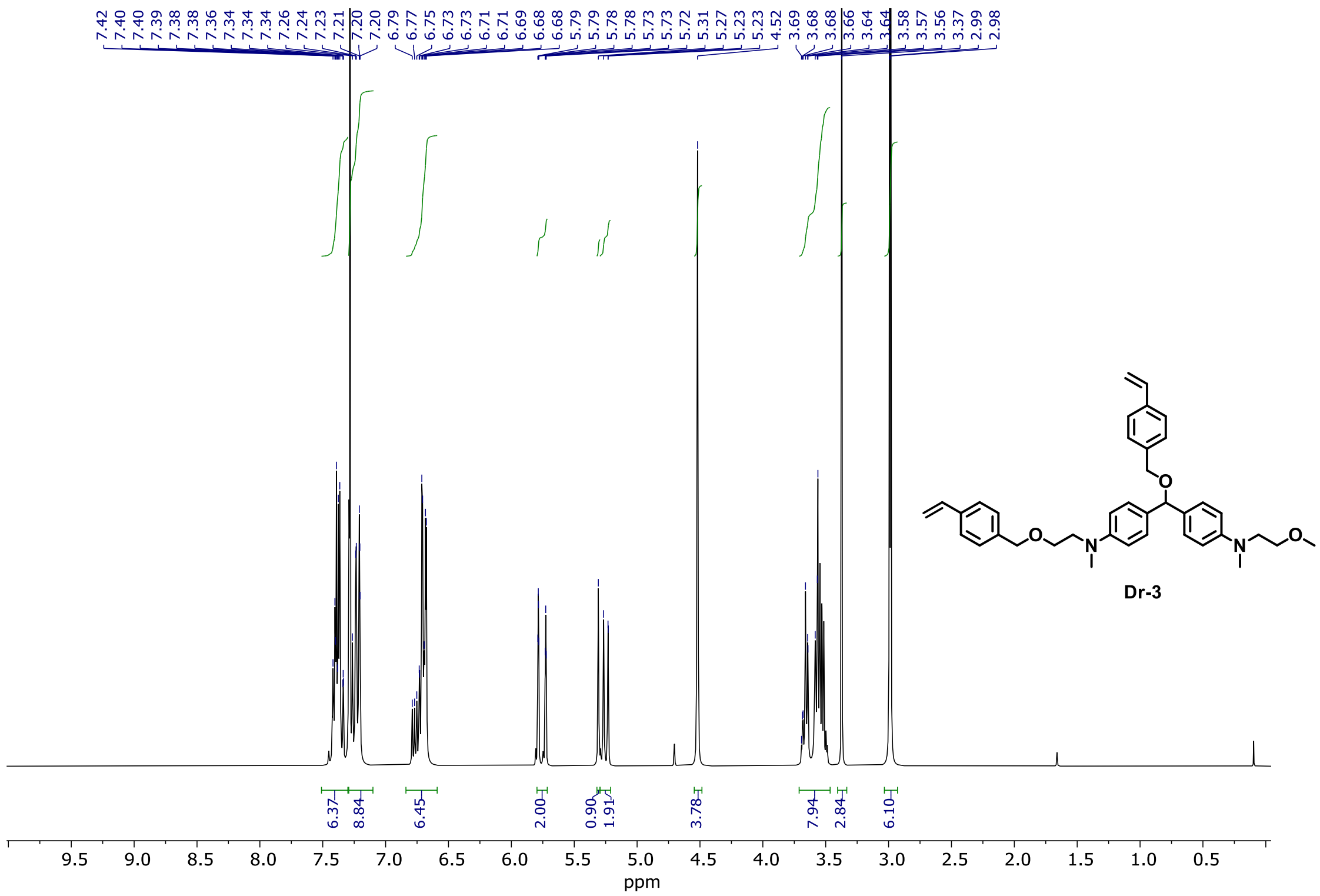

Figure S42: ^{1}H-NMR spectrum of **Dr-3** (300 MHz, $CDCl_3$, 293 K).

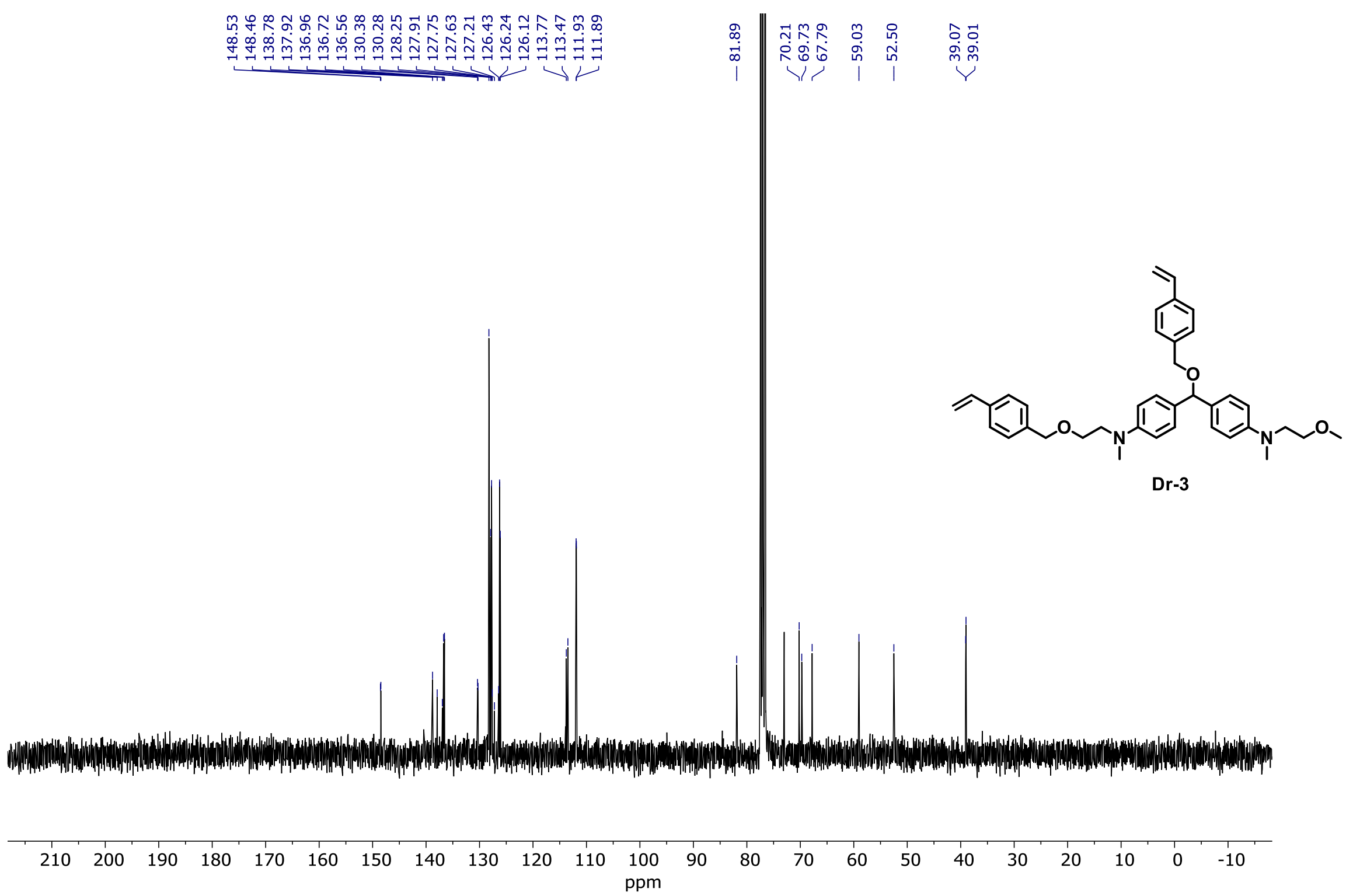

Figure S43: ^{13}C-NMR spectrum of **Dr-3** (75 MHz, $CDCl_3$, 293 K).

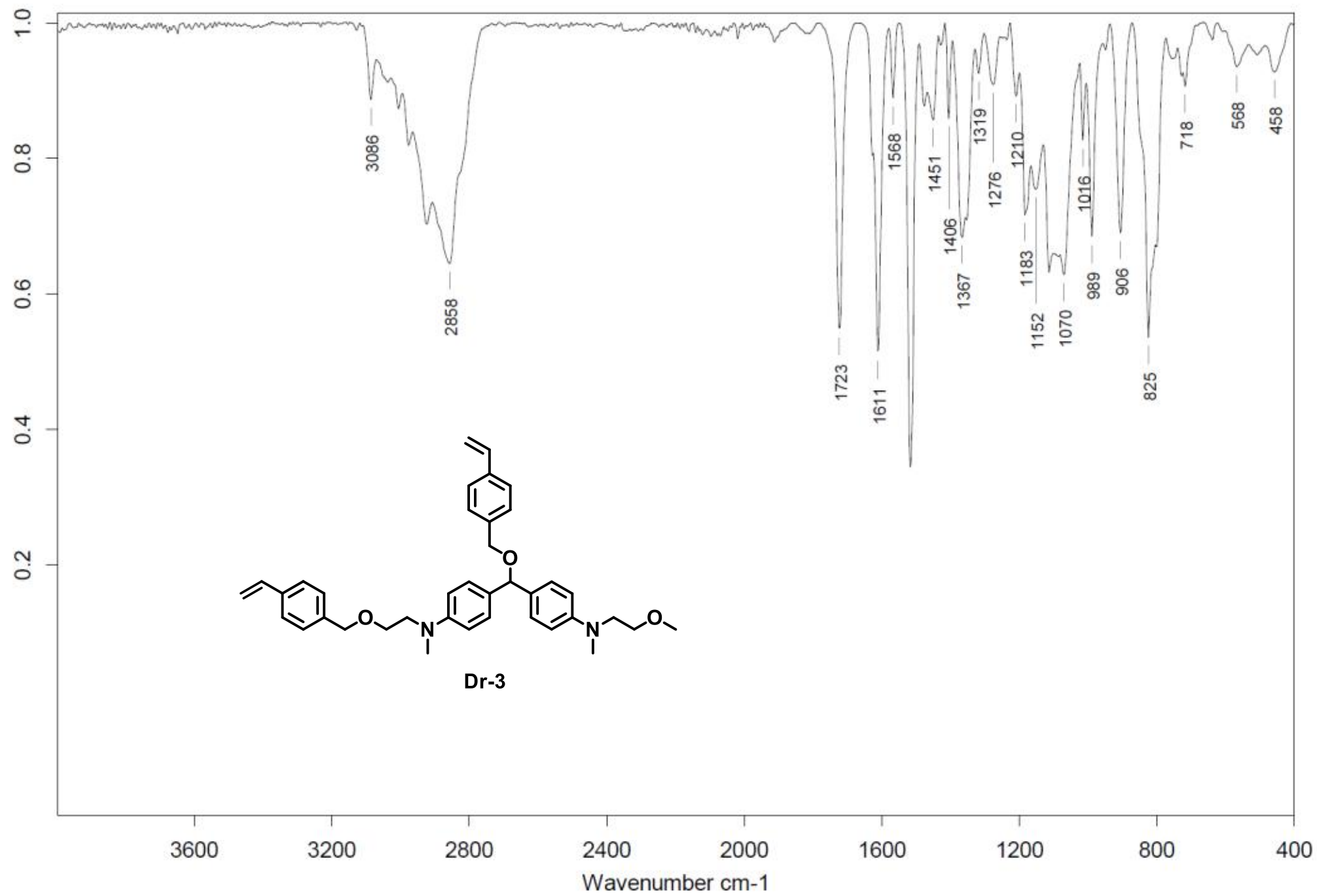

Figure S44: FT-IR spectrum of **Dr-3**

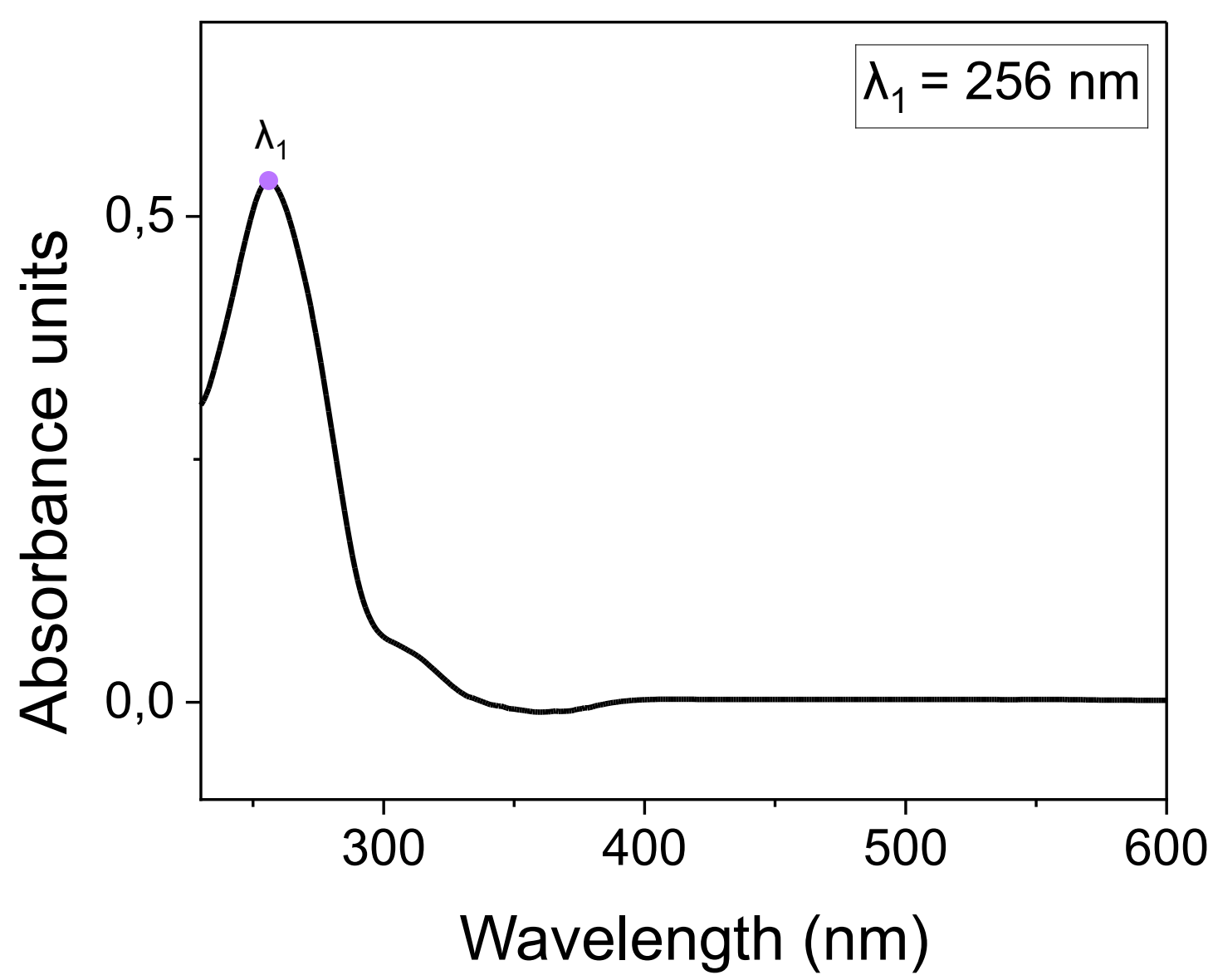

Figure S45: UV-Vis spectrum of **Dr-3** (MeCN, 1.4 × 10^{-5} M, 297 K).

2-((4-((2-methoxyethyl)(methyl)amino)phenyl)(4-(methyl(2-((4-vinylbenzyl)oxy)ethyl)amino)phenyl)methyl)-2-(4-vinylbenzyl)malononitrile (Dr-4)

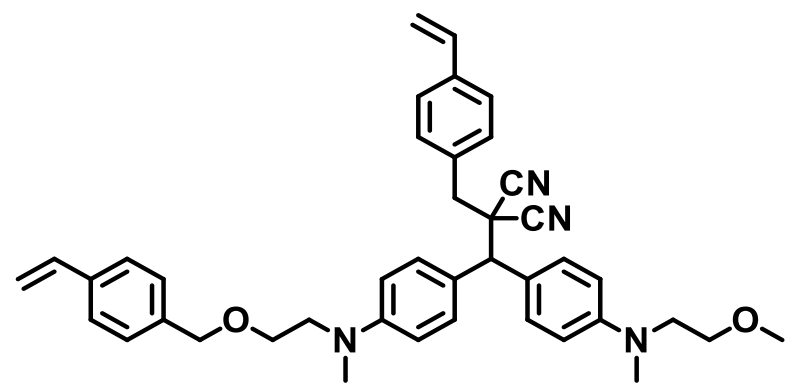

Dr-4

In a round-bottom flask, sodium hydride (18.7 mg, 0.5 $\times 10^{-3}$ mol, 1.2 eq., 60% dispersion in mineral oil) was suspended in dry DMF (2 mL) at room temperature under stirring. 2-(4-vinylbenzyl)malononitrile (71.1 mg, 0.4 $\times 10^{-3}$ mol, 1 eq.) was added in small portions, and the reaction mixture was stirred for 10 min until a homogeneous solution was obtained. A solution of 10 (0.2 g, 0.4 $\times 10^{-3}$ mol, 1 eq.) in dry DMF (2 mL) was added dropwise under stirring. Subsequently, boron trifluoride diethyl etherate ($BF_3 \cdot OEt_2$) (46%, 0.1 mL, 0.9 $\times 10^{-3}$ mol, 2.4 eq.) was added slowly via syringe. The reaction mixture was stirred at room temperature for 1 h. The solvent was removed under reduced pressure. The crude product was purified by flash column chromatography (Flash SiO_2, hexane/ethyl acetate gradient containing 2% triethylamine) to afford the title compound (103 mg, yield: 32%).

^{1}H NMR (300 MHz, $CDCl_3$): δ/ppm 7.46–7.27 (m, 16H, Ar–H), 6.79–6.68 (dd, 2H, CH=, *J* ≈ 17.5, 10.8 Hz), 5.82–5.73 (dd, 2H, CH_2=, *J* ≈ 17.5, 1.2 Hz), 5.32–5.24 (dd, 2H, CH_2=, *J* ≈ 10.8, 1.2 Hz), 5.30 (s, 1H, $Ar_2CH–C(CN)_2$), 4.53 (s, 2H, O–CH_2–Ar), 4.18 (s, 2H, Ar–CH_2–$C(CN)_2$), 3.68–3.58 (m, 8H, –O–CH_2–CH_2–N–), 3.38 (s, 3H, OCH_3), 3.01 (2s, 6H, N–CH_3).

^{13}C NMR (75 MHz, $CDCl_3$): δ/ppm 148.92, 148.84, 137.85, 137.74, 136.99, 136.56, 136.29, 132.17, 130.62, 129.78, 127.76, 126.52, 126.27, 124.87, 124.78, 115.71, 114.52, 113.80, 112.17, 112.15, 73.05, 70.20, 67.74, 60.43, 59.08, 56.64, 52.31, 52.28, 45.57, 43.09, 38.93, 38.86.

FT-IR $(cm)^{-1}$: 2872, 1609, 1518, 1450, 1374, 1190, 1108, 989, 908, 814, 730.

HR-MS (m/z): $[M+H]^+$ calcd for $C_{41}H_{45}N_4O_2$ 624.8262; found 624.8241

UV-Vis: Extinction coefficients (ε [$L \cdot mol^{-1} \cdot cm^{-1}$]) in the λ maxima ($\lambda_1$) of 3.3 × 10^{-5} M MeCN solution of **Dr-4**

λ_1, nm (ε): 273 (37326).

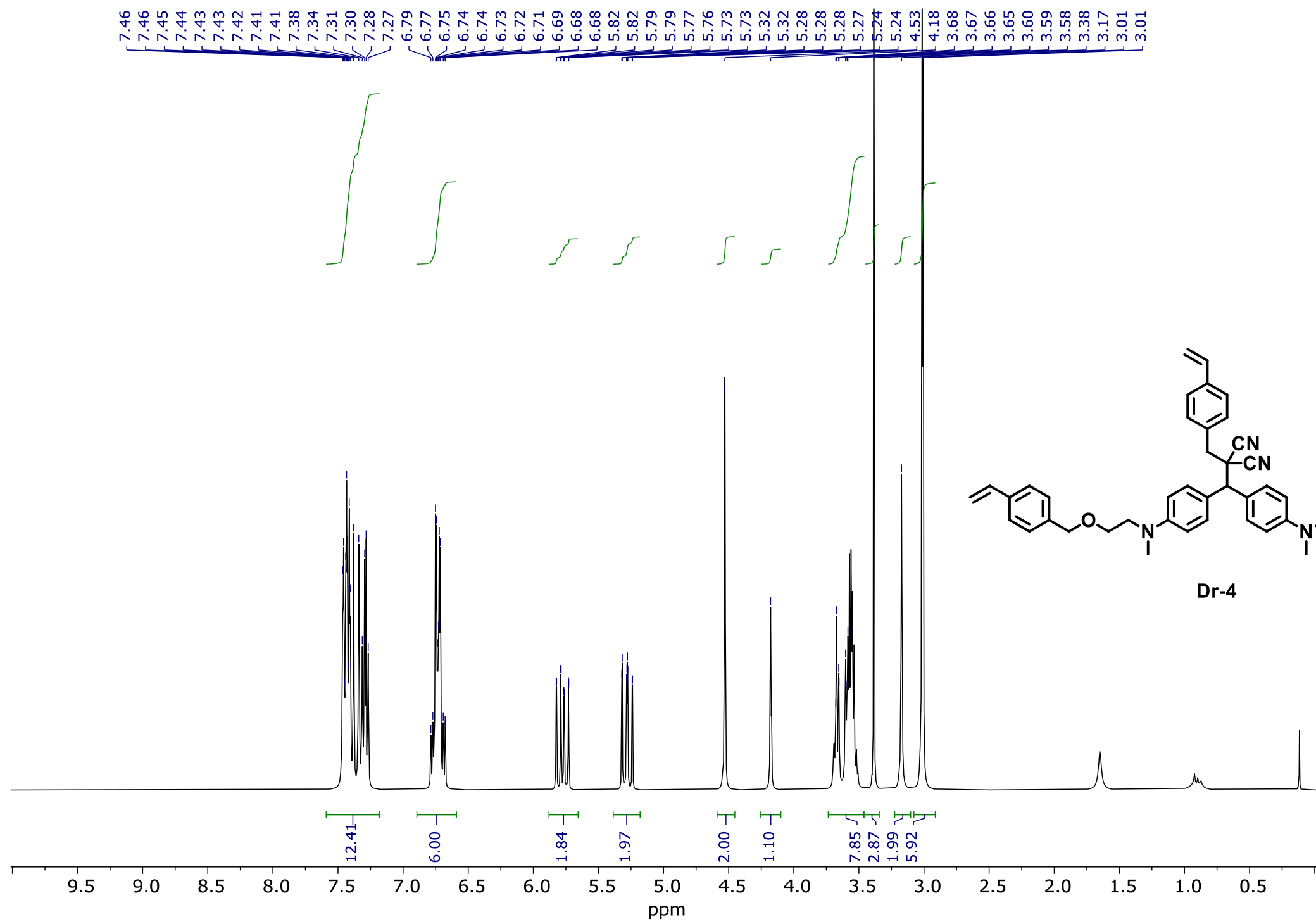

Figure S46: ^{1}H-NMR spectrum of **Dr-4** (300 MHz, $CDCl_3$, 293 K).

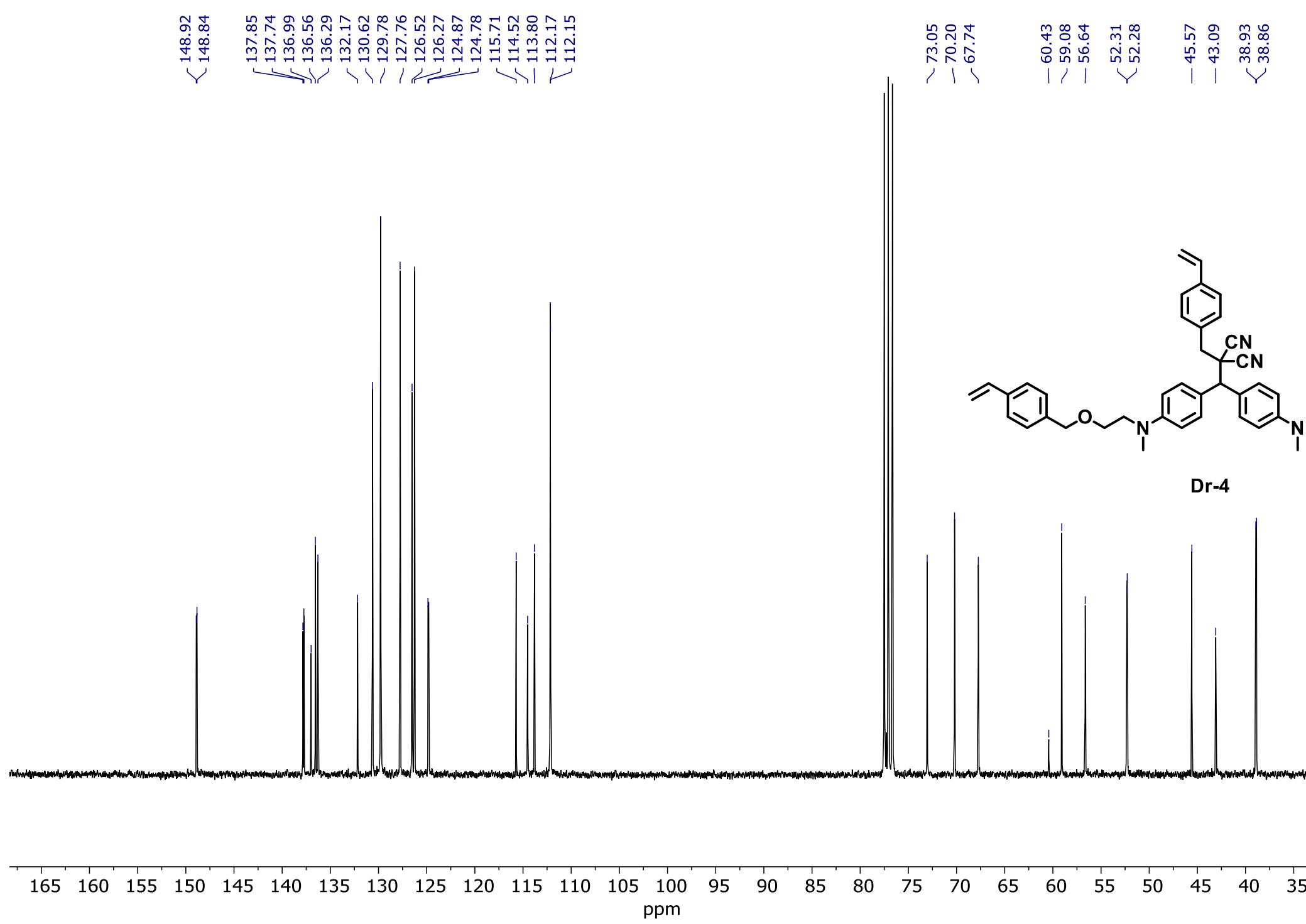

Figure S47: ^{13}C-NMR spectrum of **Dr-4** (75 MHz, $CDCl_3$, 293 K).

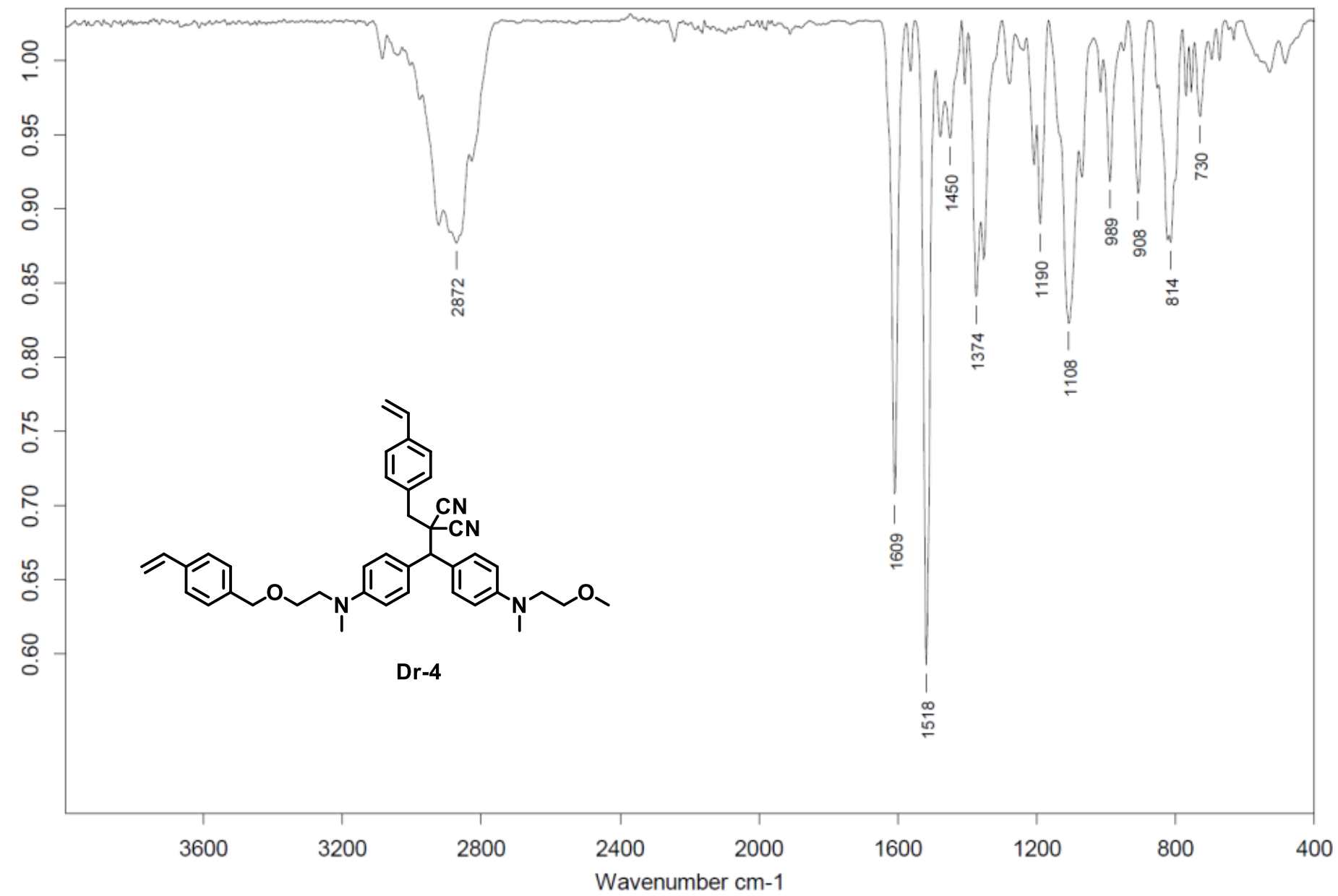

Figure S48: FT-IR spectrum of **Dr-4**.

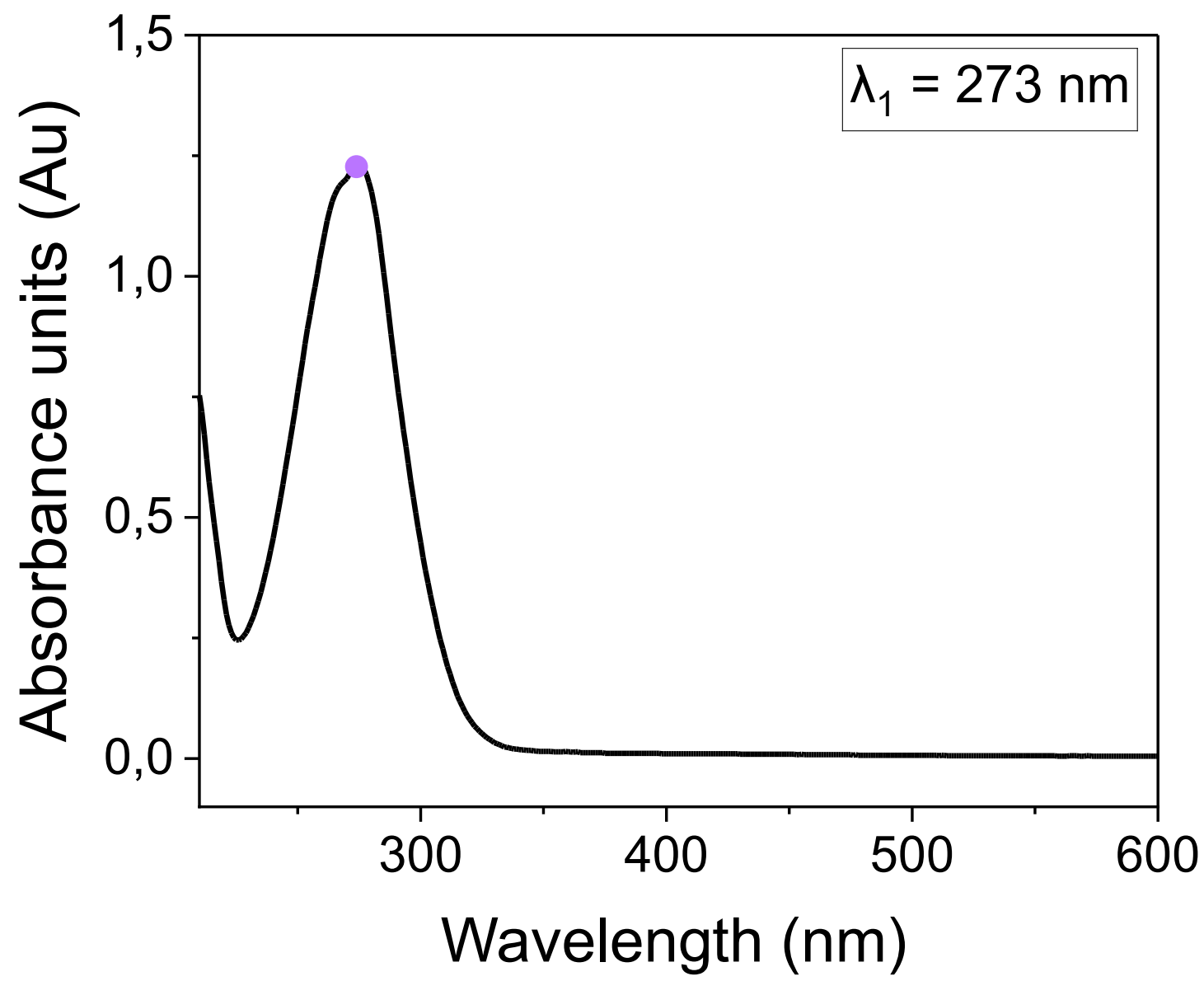

Figure S49: UV-Vis spectrum of **Dr-4** (MeCN, 3.3×10^{-5} M, 297 K).

4-((benzyloxy)(4-methoxyphenyl)methyl)-N-methyl-N-(2-((4-vinylbenzyl)oxy)ethyl)aniline (Dr-I)

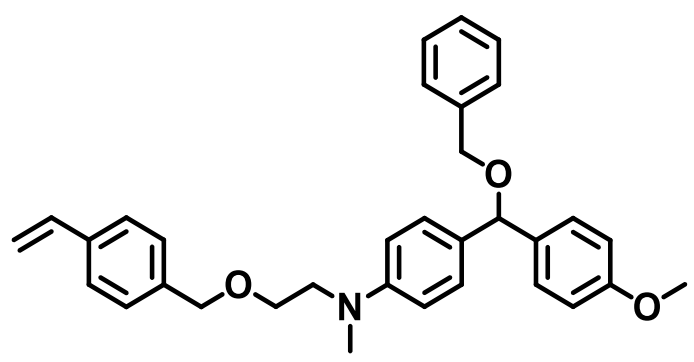

Dr-I

In a two-neck round-bottom flask, compound **4** (160 mg, 0.4 $\times 10^{-3}$ mol, 1 eq.) was dissolved in dry DMF (12 mL). Potassium *tert*-butoxide (89 mg, 0.8 $\times 10^{-3}$ mol, 2 eq.) was added, and the reaction mixture was stirred for 10 min. (Chloromethyl)benzene (100 mg, 0.8 $\times 10^{-3}$ mol, 2 eq.) was added dropwise via syringe through a septum, and the reaction mixture was stirred at room temperature for 12 h. The reaction was quenched with ice-cold water (0 °C) and stirred for an additional 10 min. The mixture was extracted with ethyl acetate (2 × 15 mL). The combined organic layers were washed with brine (3 × 20 mL), dried over anhydrous Na_2SO_4, filtered, and concentrated under reduced pressure. The crude product was purified by flash column chromatography (SiO_2, hexane/ethyl acetate gradient containing 2% triethylamine) to afford **Dr-I** as a pure product (110 mg, yield: 56%).

^{1}H NMR (300 MHz, $CDCl_3$): δ/ppm 7.41–7.20 (m, 12H, Ar–H), 6.90–6.68 (m, 4H, Ar–H), 6.77–6.71 (dd, 2H, CH=, *J* ≈ 17.5, 10.8 Hz), 5.79–5.73 (dd, 2H, CH_2=, *J* ≈ 17.5, 1.2 Hz), 5.36 (s, 1H, Ar_2CH–O), 5.28–5.24 (dd, 2H, CH_2=, *J* ≈ 10.8, 1.2 Hz), 4.55–4.52 (s, 4H, O–CH_2–Ar), 3.82 (s, 3H, OCH_3), 3.69–3.55 (m, 4H, –O–CH_2–CH_2–N–), 3.00 (s, 3H, N–CH_3).

^{13}C NMR (75 MHz, $CDCl_3$): δ/ppm 158.74, 148.60, 138.87, 137.90, 136.98, 136.55, 135.08, 129.79, 128.32, 128.29, 127.74, 127.72, 127.36, 126.25, 113.79, 113.66, 111.89, 81.77, 73.02, 70.13, 67.78, 55.27, 52.51, 39.05.

HR-MS (m/z): $[M+H]^+$ calcd for $C_{38}H_{37}NO_3$ 494.2786; found 494.2732

FT-IR $(cm)^{-1}$: 2856, 1610, 1509, 1462, 1406, 1370, 1244, 1171, 1070, 1033, 990, 906, 824.

UV-Vis: Extinction coefficients (ε [$L \cdot mol^{-1} \cdot cm^{-1}$]) in the λ maxima ($\lambda_1$) of 2.6 × 10^{-5} M MeCN solution of **Dr-I**

λ_1, nm (ε): 268 (30249).

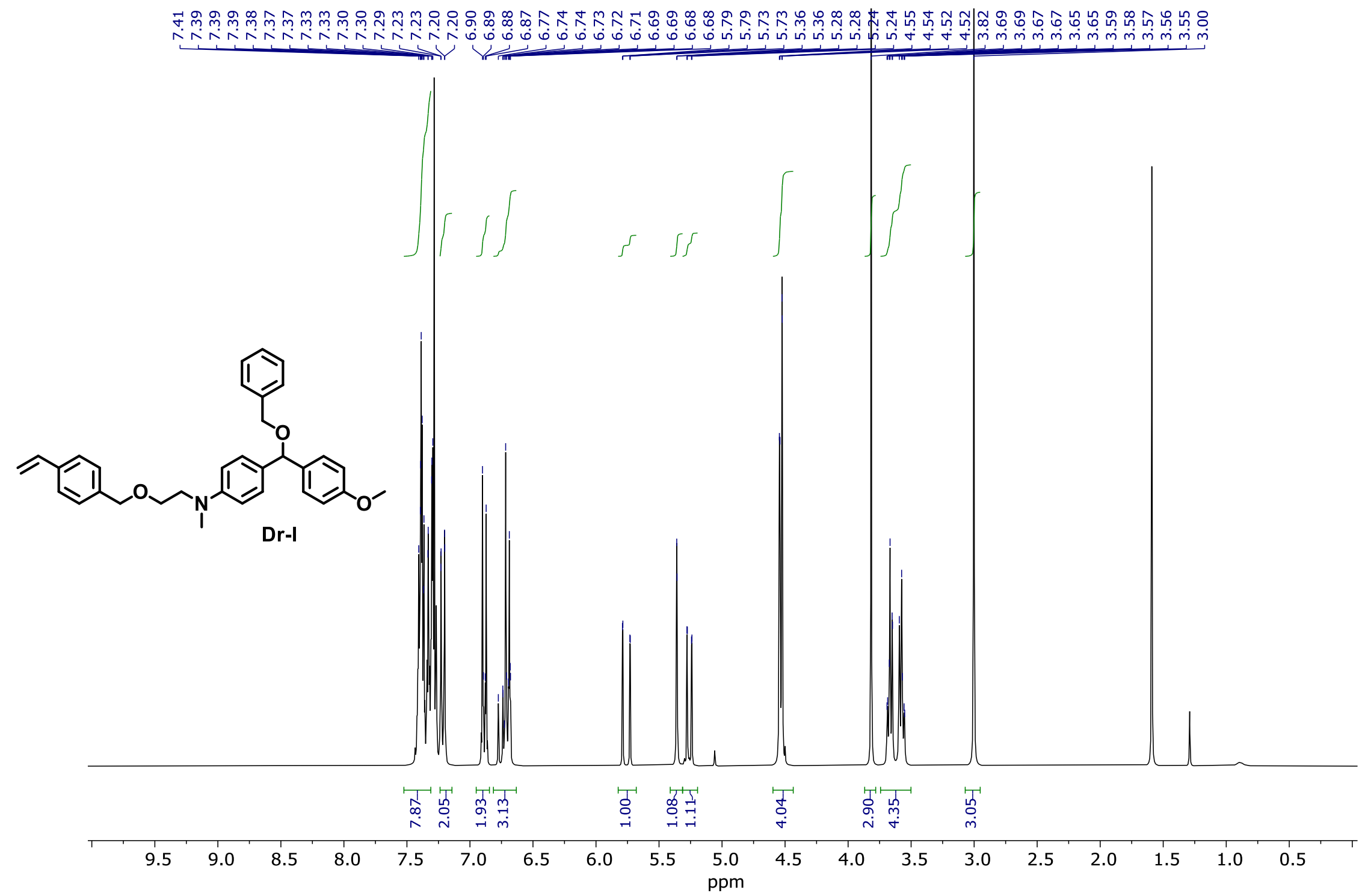

Figure S50: ^{1}H-NMR spectrum of **Dr-I** (300 MHz, $CDCl_3$, 293 K).

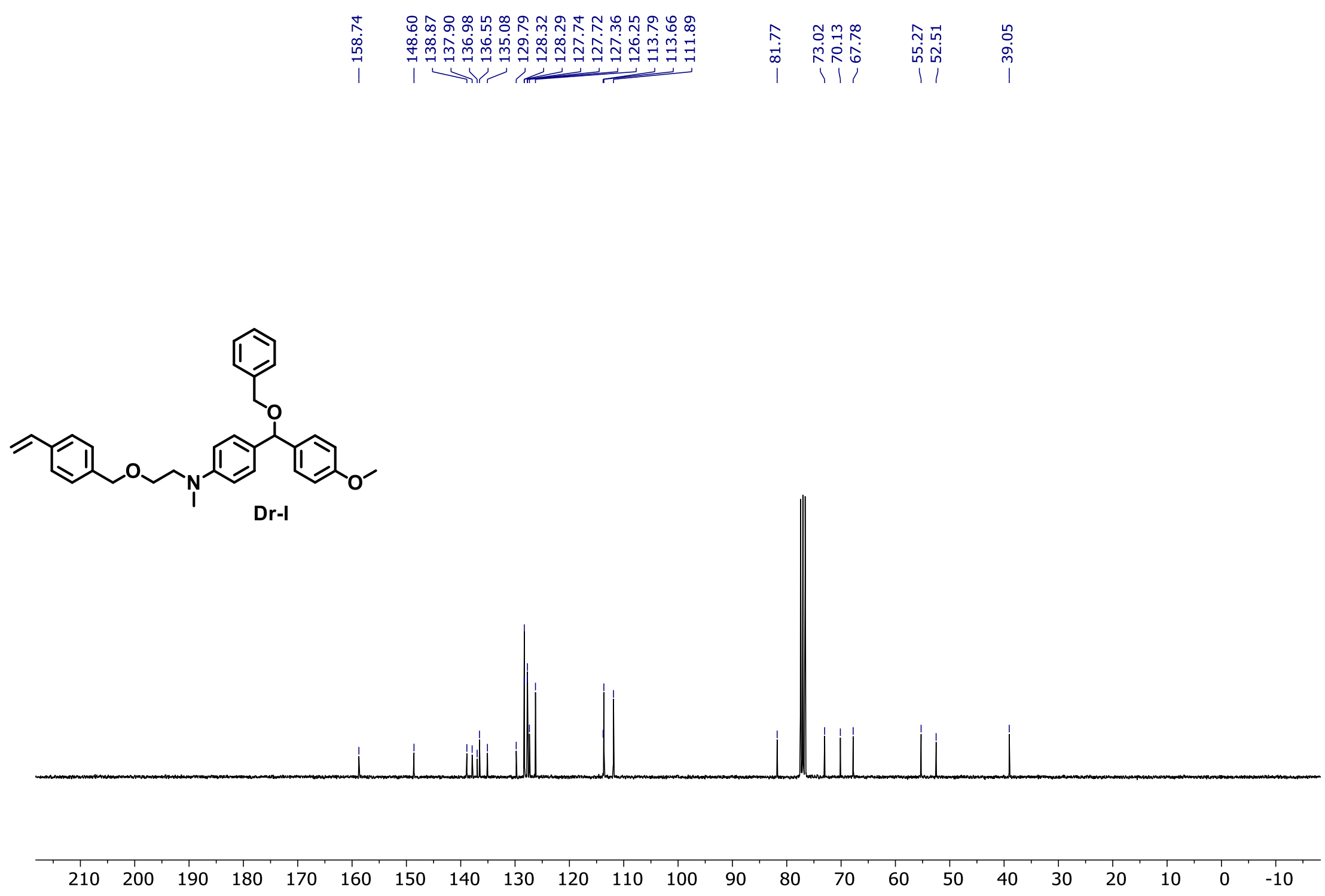

Figure S51: ^{13}C-NMR spectrum of **Dr-I** (75 MHz, $CDCl_3$, 293 K).

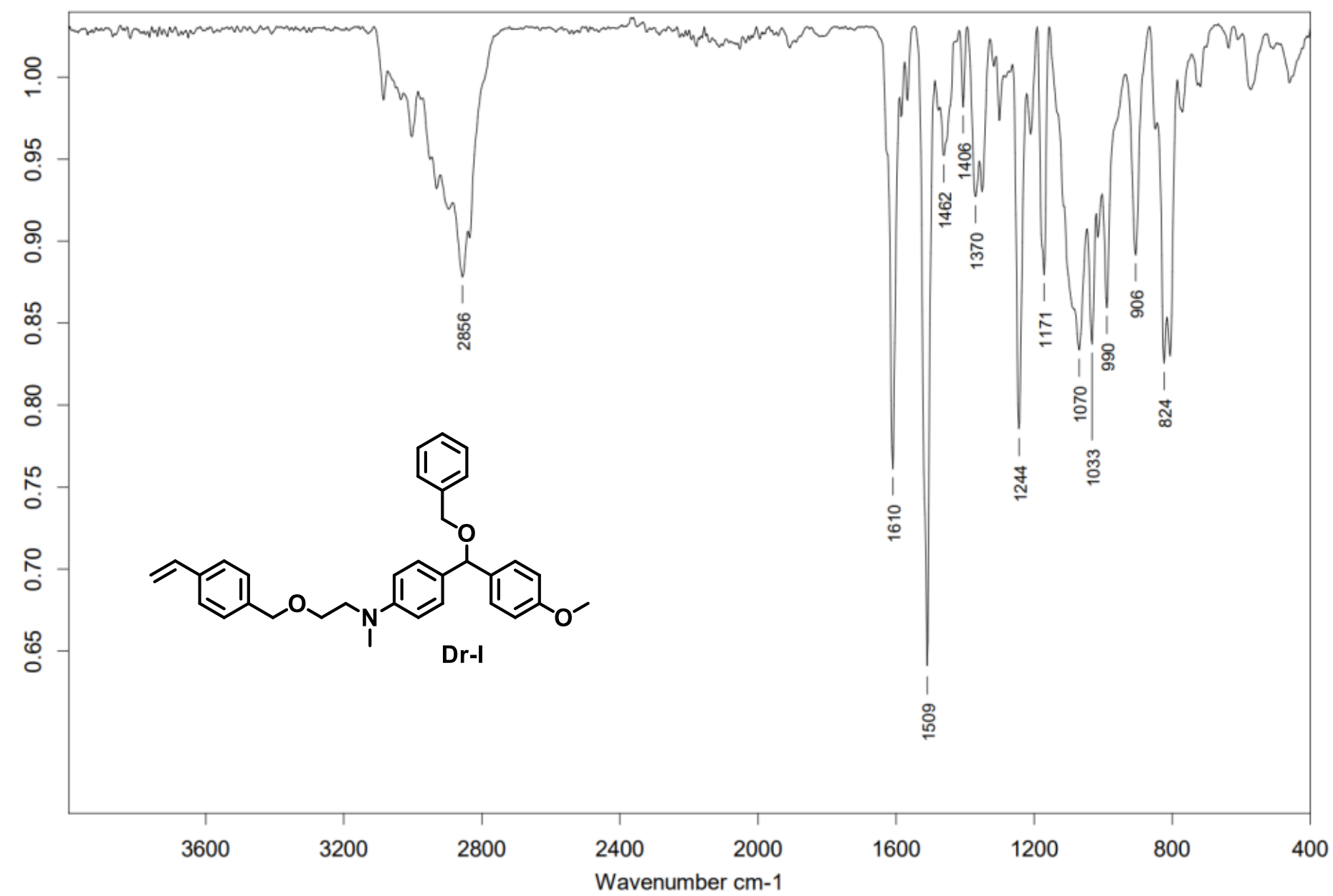

Figure S52: FT-IR spectrum of **Dr-I**.

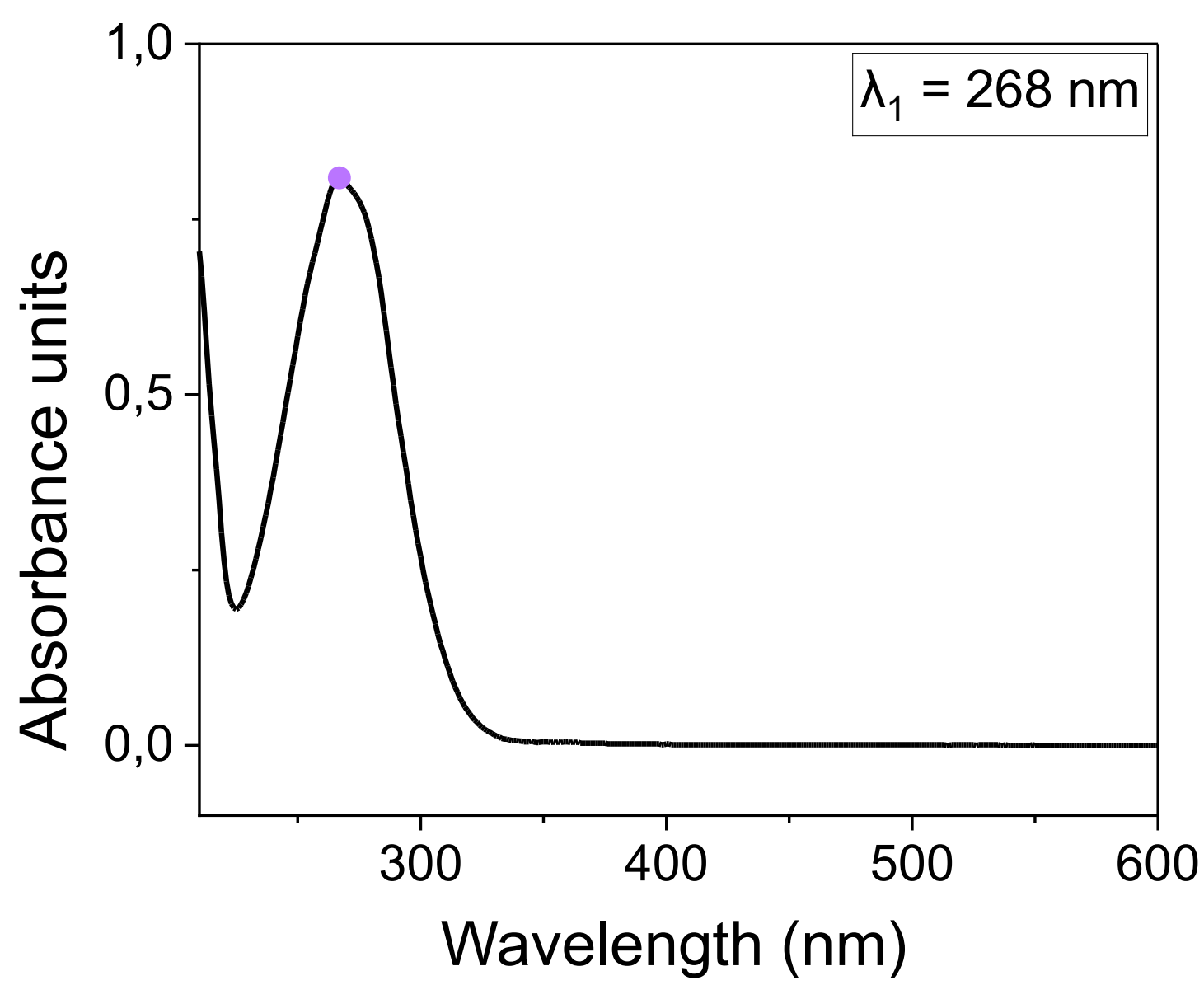

Figure S53: UV-Vis spectrum of **Dr-I** (MeCN, 2.6 × 10^{-5} M, 297 K).

2-benzyl malononitrile (12)

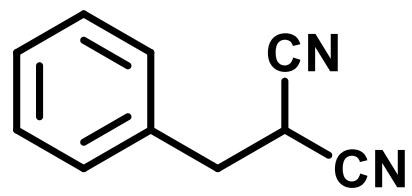

12

Compound **12** was prepared as reported in literature.[13] In a round-bottom flask, malononitrile (0.9 g, 1.3 $\times 10^{-2}$ mol, 1.5 eq.) and benzyl chloride (1.1 g, 8.7 $\times 10^{-2}$ mol, 1 eq.) were dissolved in dry THF (20 mL) and stirred at room temperature. Sodium hydride (0.5 g, 1.3 $\times 10^{-2}$ mol, 1.5 eq., 60% w/w dispersion in mineral oil) was added in small portions over 5 min under an argon flow. The reaction mixture was heated to 65 °C and stirred for 4 h. The reaction was quenched with water, and the aqueous phase was extracted with dichloromethane (3 × 20 mL). The combined organic layers were dried over anhydrous Na_2SO_4, filtered, and concentrated under reduced pressure. The crude product was purified by flash column chromatography to afford **12** (0.9 g, yield: 45%). The spectral data are in accordance with the literature.[14]

^{1}H NMR (300 MHz, $CDCl_3$): δ/ppm 7.45–7.34 (m, 5H, Ar–H), 3.96–3.91 (s, 1H, $CH(CN)_2$), 3.33–3.30 (s, 2H, Ph–CH_2–).

^{13}C NMR (75 MHz, $CDCl_3$): δ/ppm 132.95, 129.33, 129.15, 128.85, 112.17, 36.77, 25.02.

FT-IR (cm)$^{-1}$: 3067, 3090, 3031, 2914, 2258, 1603, 1496, 1446, 1454, 1329, 1251, 1206, 1163, 1075, 1031, 746, 699, 566, 591, 481.

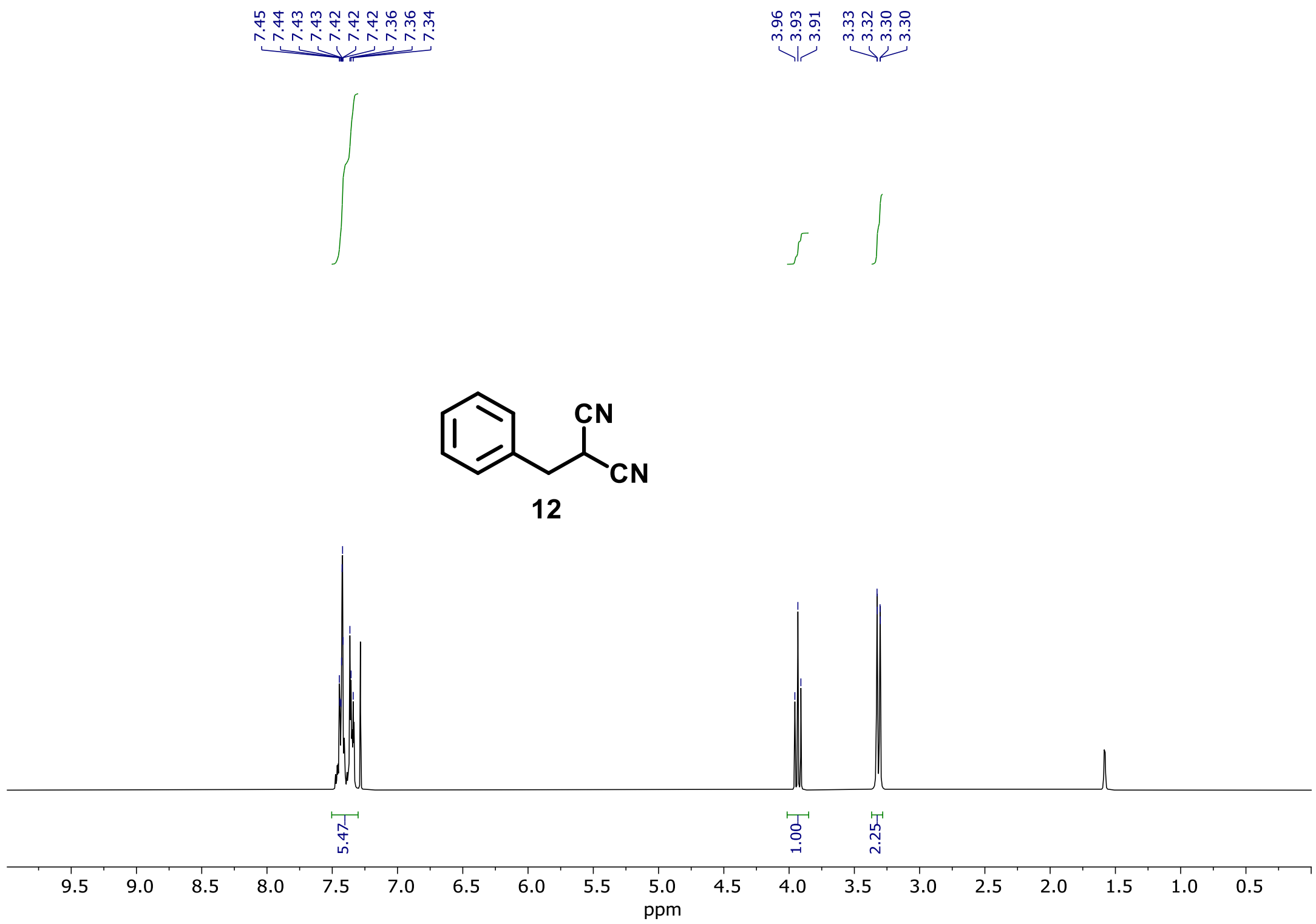

Figure S54: ^{1}H-NMR spectrum of **12** (300 MHz, $CDCl_3$, 293 K).

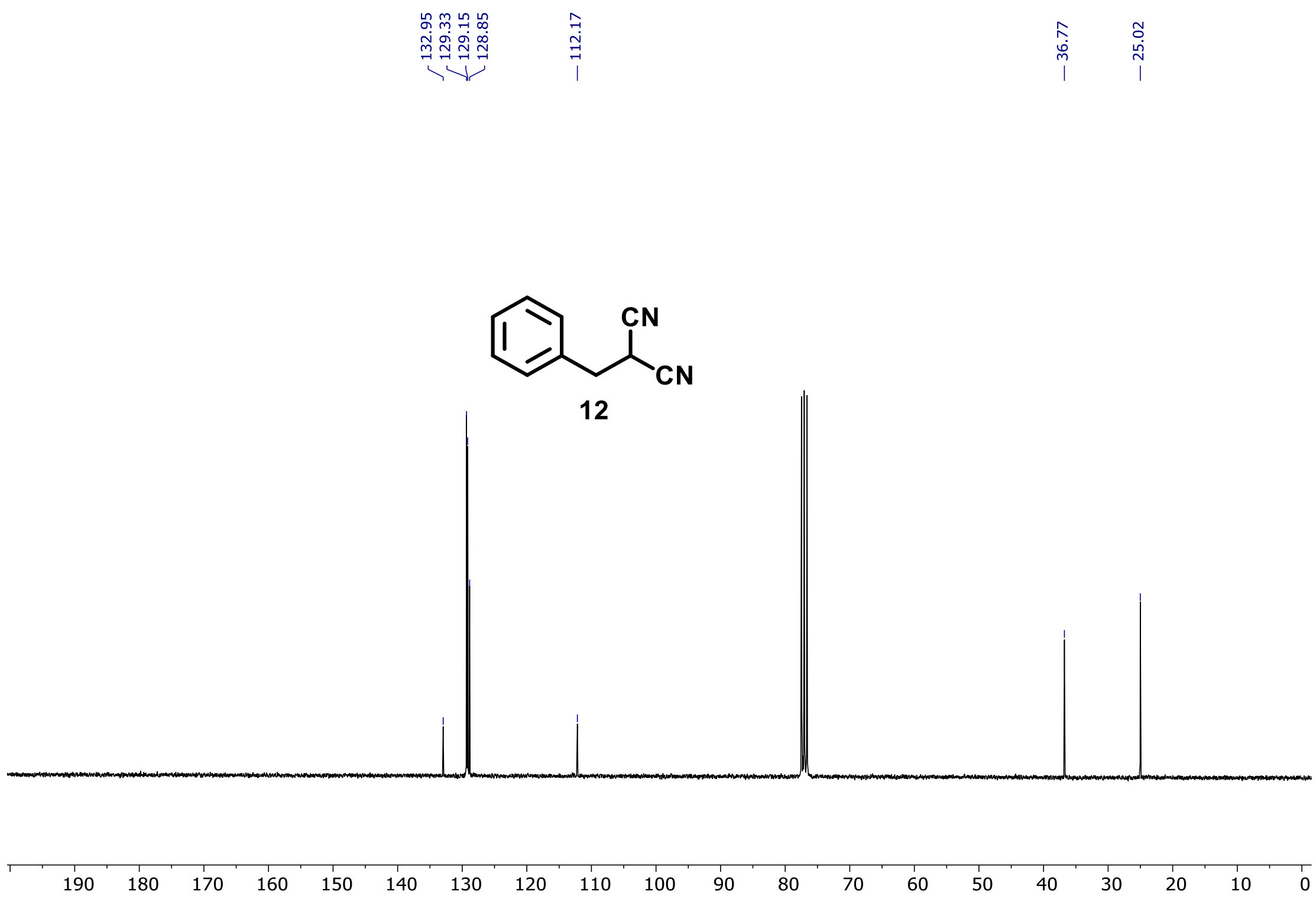

Figure S55:^{13}C-NMR spectrum of **12** (75 MHz, $CDCl_3$, 293 K).

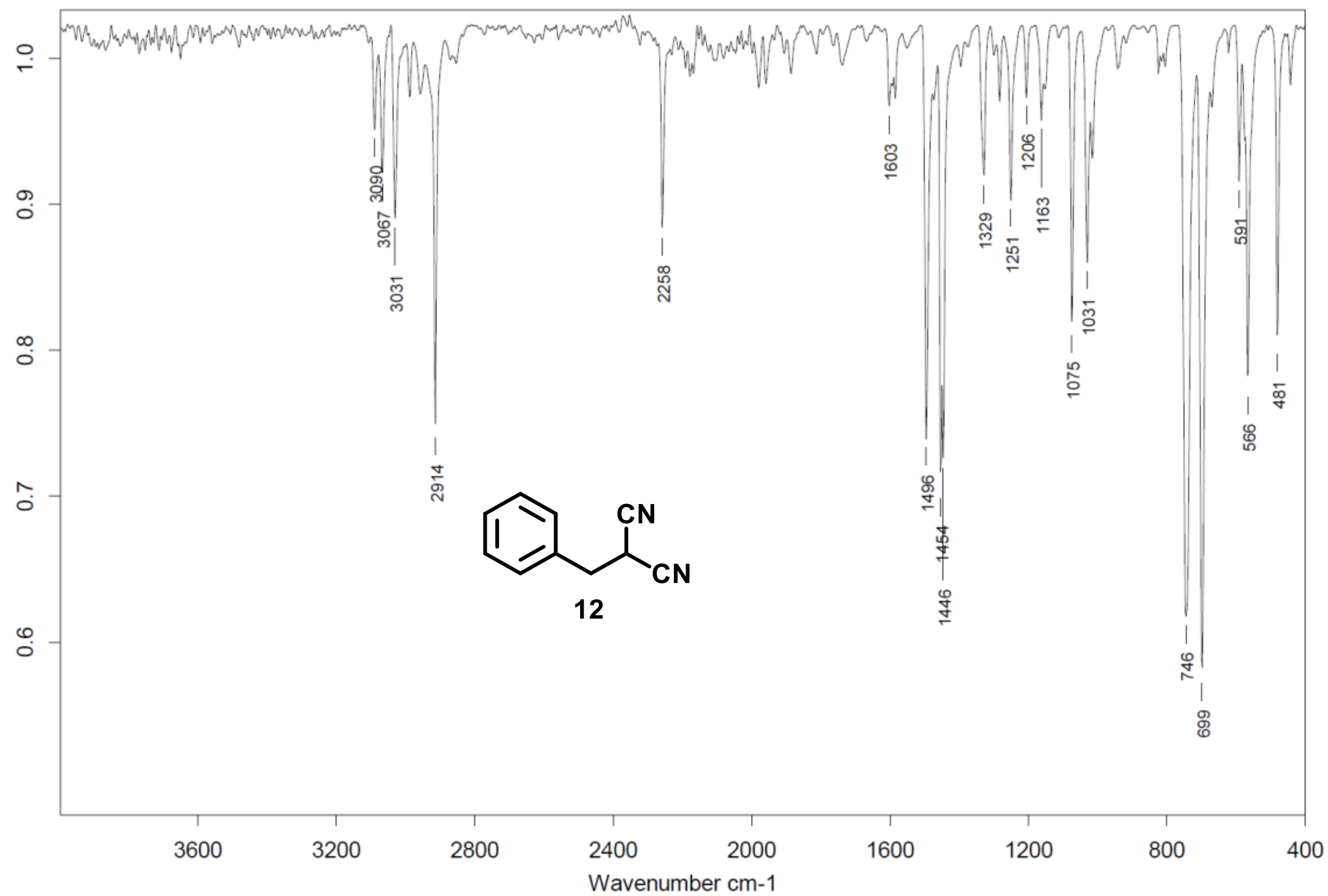

Figure S56: FT-IR spectrum of **12**.

2-benzyl-2-((4-methoxyphenyl)(4-(methyl(2-((4-vinylbenzyl)oxy)ethyl)amino)phenyl)methyl)malononitrile (Dr-II)

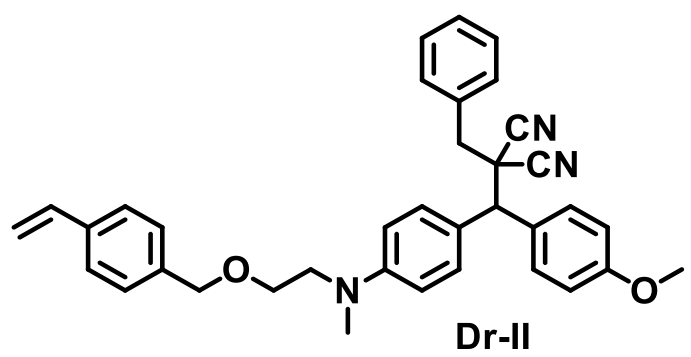

In a round-bottom flask, sodium hydride (21.8 mg, 0.5 $\times 10^{-3}$ mol, 2.2 eq., 60% dispersion in mineral oil) was suspended in dry DMF (2 mL) at room temperature under stirring. 2-benzylmalononitrile (77.4 mg, 0.5 $\times 10^{-3}$ mol, 2.0 eq.) was added in small portions under an argon flow, and the reaction mixture was stirred for 10 min to allow complete deprotonation. A solution of **12** (102 mg, 0.3 $\times 10^{-3}$ mol, 1 eq.) in dry DMF (2 mL) was added dropwise under stirring. Subsequently, boron trifluoride diethyl etherate ($BF_3 \cdot OEt_2$) (46%, 50 mg, 0.4 $\times 10^{-3}$ mol, 1.5 eq.) was added slowly via syringe. The reaction mixture was stirred at room temperature until completion. The reaction was quenched, and the solvent was removed under reduced pressure. The crude mixture was purified by flash column chromatography (Flash SiO_2, hexane/ethyl acetate gradient containing 2% triethylamine) to afford **Dr-II** (85 mg, yield: 32%).

^{1}H NMR (300 MHz, $CDCl_3$): δ/ppm 7.54–7.25 (m, ~9H, Ar–H), 6.95–6.66 (m, ~8H, Ar–H), 6.77–6.70 (dd, 1H, CH_2=CH–Ph, *J* ≈ 17.5, 10.8 Hz), 5.79–5.71 (dd, 1H, $CH_2=_{trans}$, *J* ≈ 17.5, 1–1.5 Hz), 5.28–5.23 (dd, 1H, $CH_2=_{cis}$, *J* ≈ 10.8, 1–1.5 Hz), 4.52 (s, 2H, O–CH_2–), 4.22 (s, 2H, Ph–CH_2–C(CN)$_2$), 3.83 (s, 3H, OCH_3), 3.68–3.56 (m, 2H, –O–CH_2–CH_2–N–), 3.18–3.17 (m, 2H, –O–CH_2–CH_2–N–), 3.02 (s, 3H, N–CH_3).

^{13}C NMR (126 MHz, $CDCl_3$): δ/ppm 159.38, 137.73, 137.02, 136.50, 132.92, 132.41, 130.40, 130.02, 129.84, 129.33, 129.14, 128.75, 128.60, 127.75, 126.25, 115.44, 114.40, 113.83, 112.12, 73.05, 67.58, 56.55, 55.29, 45.34, 43.29, 36.80, 25.02.

FT-IR (cm)$^{-1}$: 2924, 1732, 1609, 1511, 1455, 1376, 1250, 1181, 1094, 1030, 910, 814, 759, 700, 554

HR-MS (m/z): $[M+H]^+$ calcd for $C_{36}H_{36}N_3O_2$ 542.2799; found 542.2802

UV-Vis: Extinction coefficients (ε [L·mol^{-1}·cm^{-1}]) in the λ maxima (λ_1) of 2.9 × 10^{-5} M MeCN solution of **Dr-II**.

λ_1, nm (ε): 257 (31378).

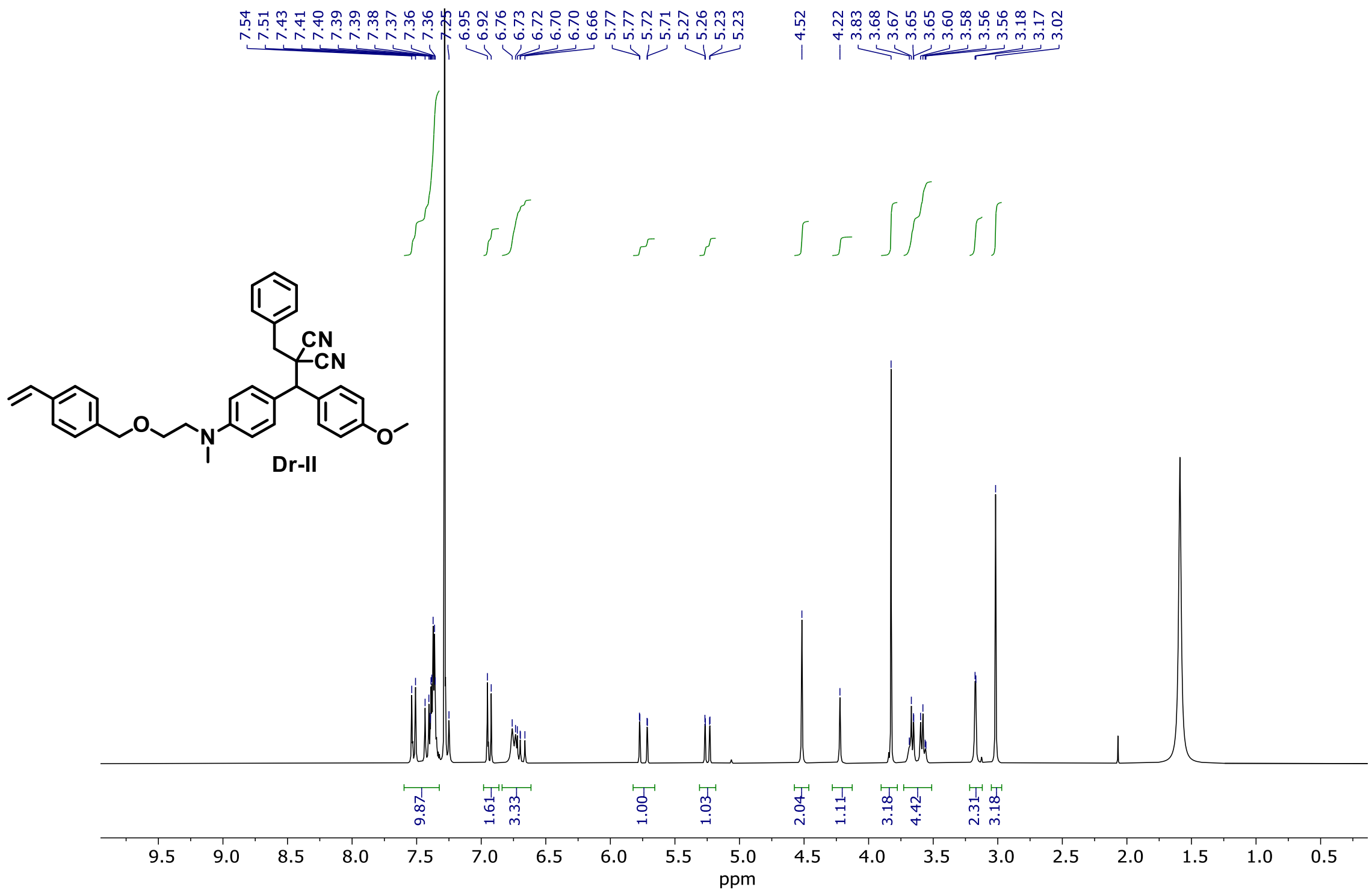

Figure S57: ^{1}H-NMR spectrum of **Dr-II** (300 MHz, $CDCl_3$, 293 K).

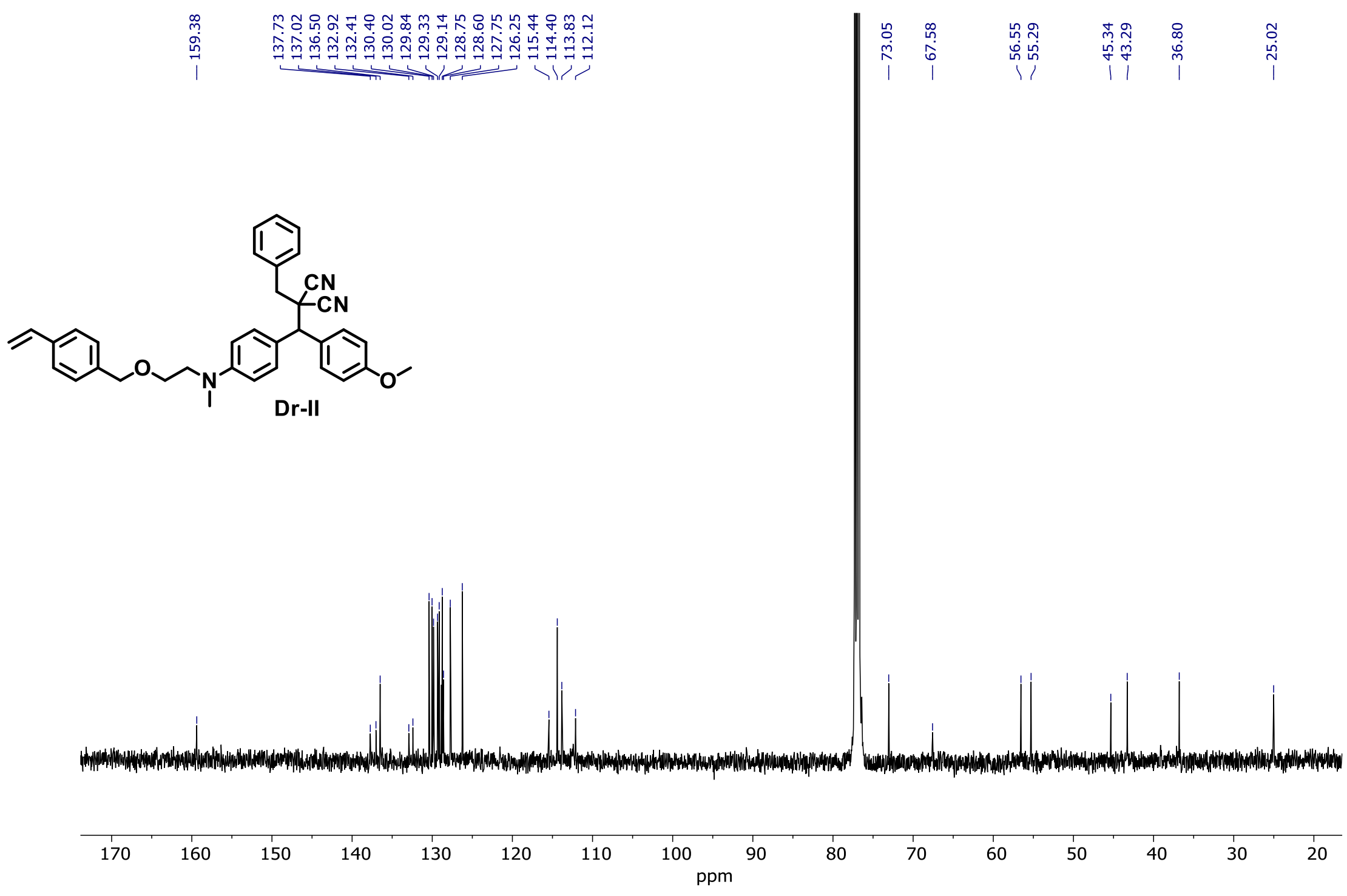

Figure S58: ^{13}C-NMR spectrum of **Dr-II** (75 MHz, $CDCl_3$, 293 K).

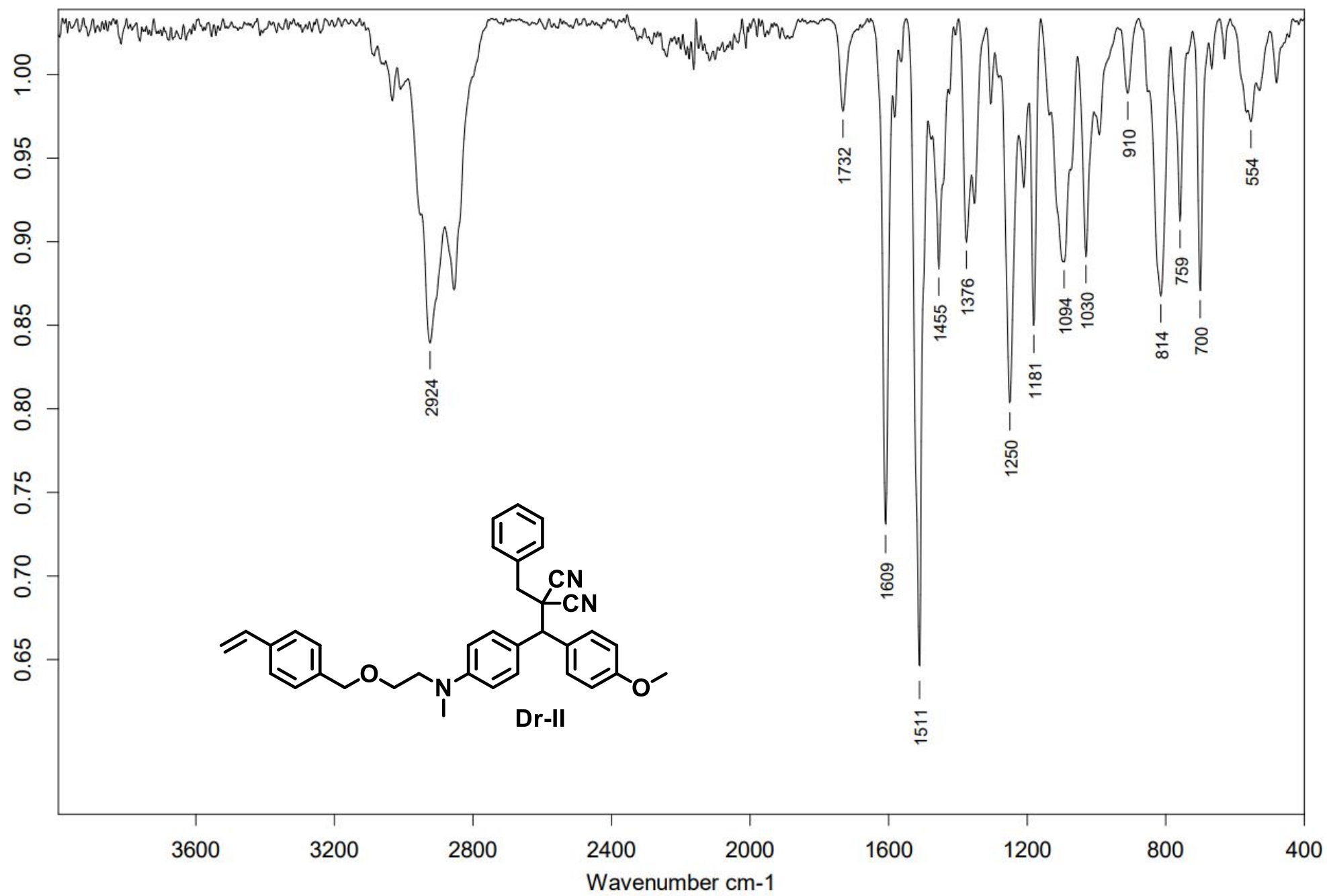

Figure S59: FT-IR spectrum of **Dr-II**.

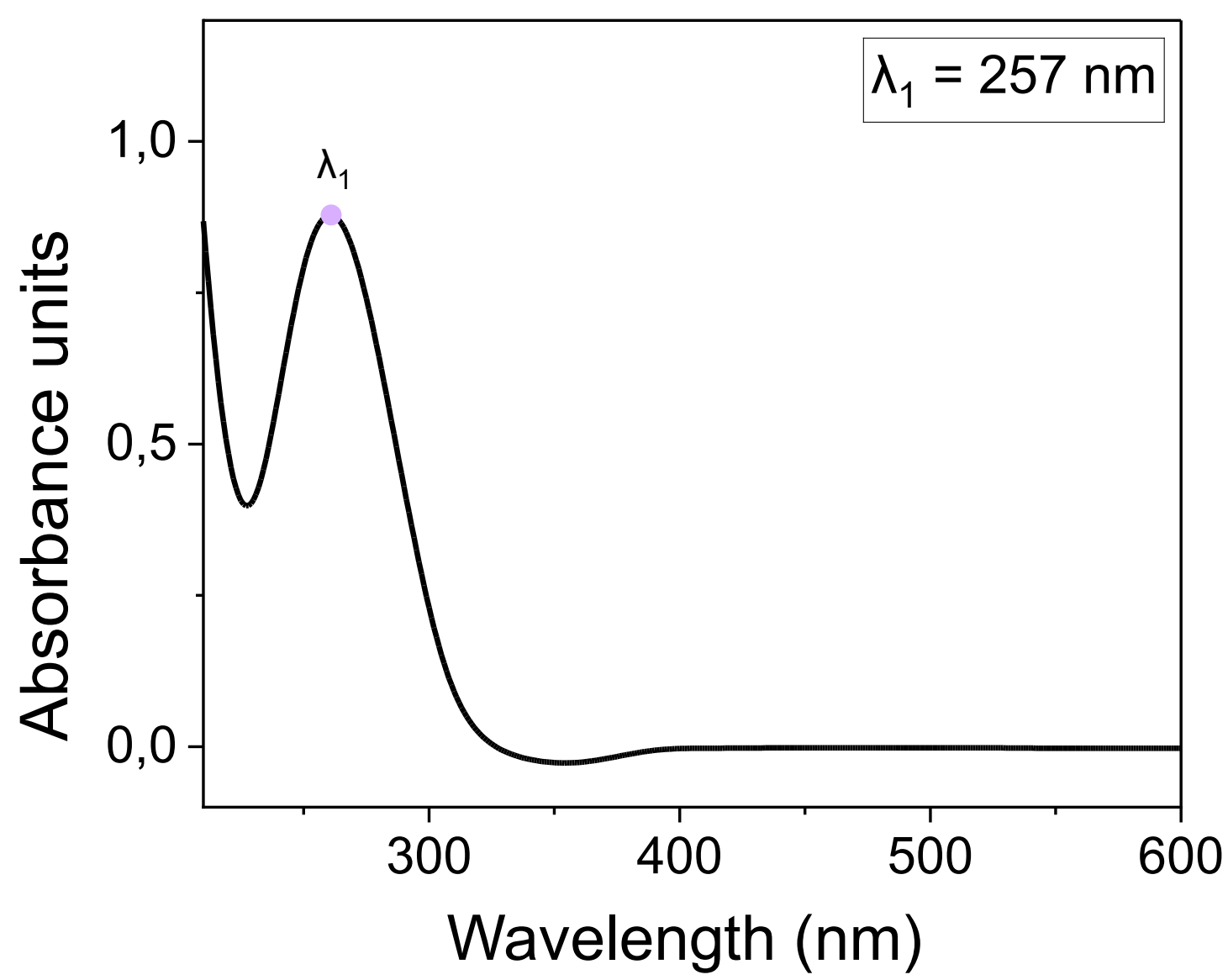

Figure S60: UV-Vis spectrum of **Dr-II** (MeCN, 2.9 × 10^{-5} M, 297 K).

4-((benzyloxy)(4-((2-methoxyethyl)(methyl)amino)phenyl)methyl)-N-methyl-N-(2-((4-vinylbenzyl)oxy)ethyl)aniline (Dr-III)

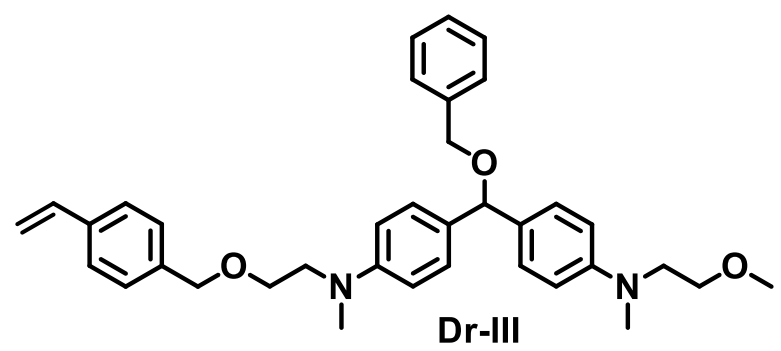

In a flame-dried two-neck round-bottom flask, **10** (80 mg, 1.7 ×10^{-4} mol, 1 eq.) was dissolved in dry DMF (2 mL) at room temperature under stirring. Potassium *tert*-butoxide (39 mg, 3.5 ×10^{-4} mol, 2.0 eq.) was added, and the reaction mixture was stirred for 20 min. (Chloromethyl)benzene (44 mg, 3.5 ×10^{-4} mol, 2.0 eq.) was added dropwise, and the reaction mixture was stirred at room temperature for 12 h under an inert atmosphere. The reaction mixture was cooled to 0 °C and quenched with distilled water (2 mL). After stirring for an additional 10 min, the aqueous phase was extracted with ethyl acetate (2 × 5 mL). The combined organic layers were washed with water and brine (3 × 5 mL), dried over anhydrous Na_2SO_4, filtered, and concentrated under reduced pressure. The crude product was purified by flash column chromatography (Flash SiO_2, hexane/ethyl acetate gradient containing 2% triethylamine) to afford **Dr-III** as a pure product (44 mg, yield: 42%).

^{1}H NMR (300 MHz, $CDCl_3$): δ/ppm 7.41–7.20 (m, 13H, Ar–H), 6.90–6.68 (m, 4H, Ar–H), 6.77–6.71 (dd, 1H, CH=, *J* ≈ 17.5, 10.8 Hz), 5.79–5.73 (dd, 1H, CH_2=, *J* ≈ 17.5, 1.2 Hz), 5.32–5.24 (dd, 1H, CH_2=, *J* ≈ 10.8, 1.2 Hz), 5.28 (s, 1H, Ar_2CH–O), 4.54 (s, 2H, O–CH_2–Ar), 4.52 (s, 2H, O–CH_2–Ar), 3.69–3.55 (m, 8H, –O–CH_2–CH_2–N– ×2), 3.38 (s, 3H, OCH_3), 3.00 (s, 3H, N–CH_3), 2.99 (s, 3H, N–CH_3).

^{13}C NMR (75 MHz, $CDCl_3$): δ/ppm 148.52, 148.45, 139.13, 137.93, 136.96, 136.56, 130.43, 130.33, 128.25, 127.75, 127.71, 127.23, 126.24, 113.77, 111.93, 111.88, 81.96, 73.02, 70.22, 70.02, 67.79, 59.04, 52.55, 52.50, 39.08, 39.02.

FT-IR $(cm)^{-1}$: 2858, 1611, 1567, 1517, 1452, 1368, 1183, 1106, 1066, 989, 906, 811, 734, 697.

HR-MS (m/z): $[M+H]^+$ calcd for $C_{36}H_{43}N_2O_3$ 551.3258; found 551.3268

UV-Vis: Extinction coefficients (ε [L·mol^{-1}·cm^{-1}]) in the λ maxima (λ_1) of 3.1 × 10^{-5} M MeCN solution of **Dr-III**

λ_1, nm (ε): 263 (29648).

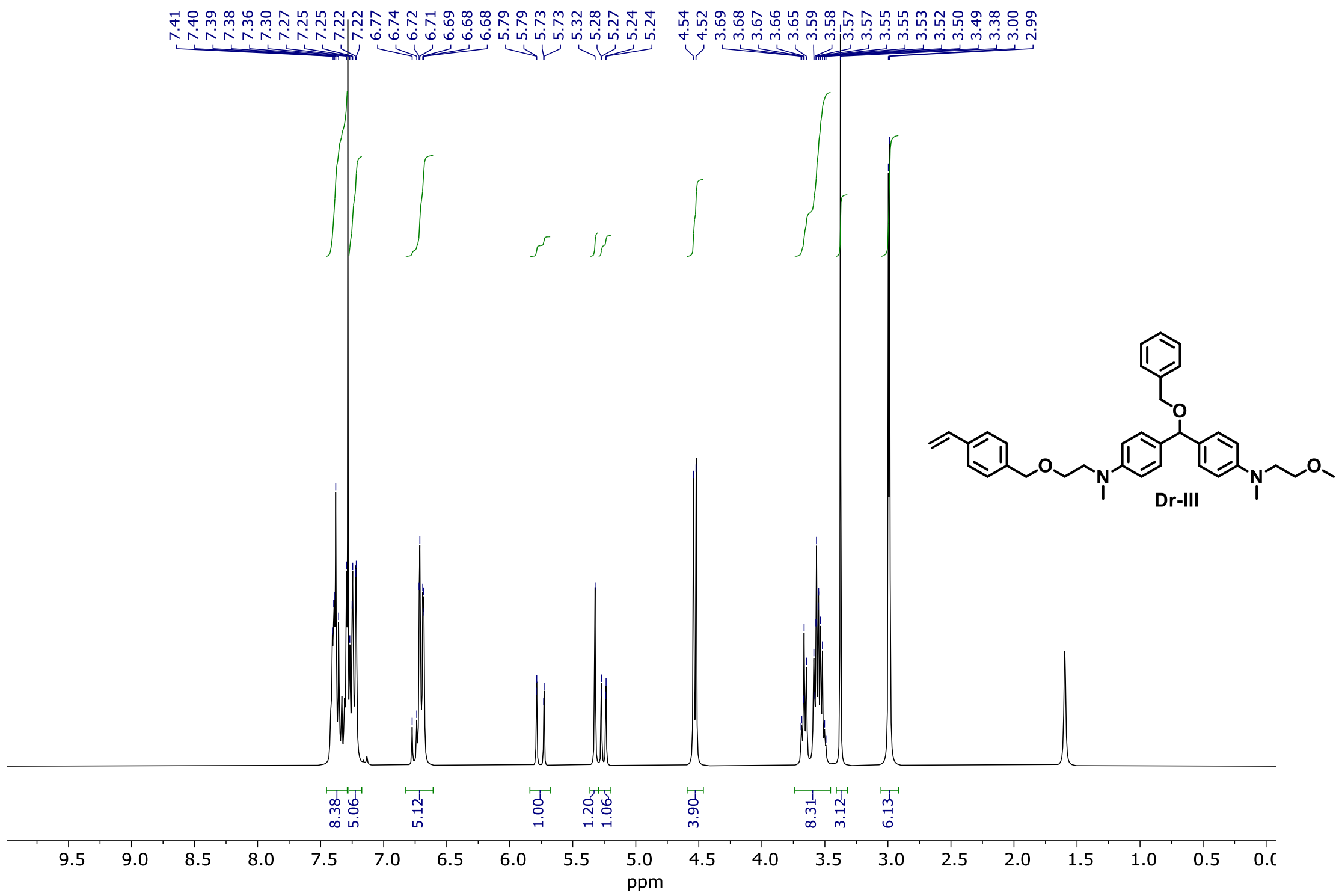

Figure S61: ^{1}H-NMR spectrum of **Dr-III** (300 MHz, $CDCl_3$, 293 K).

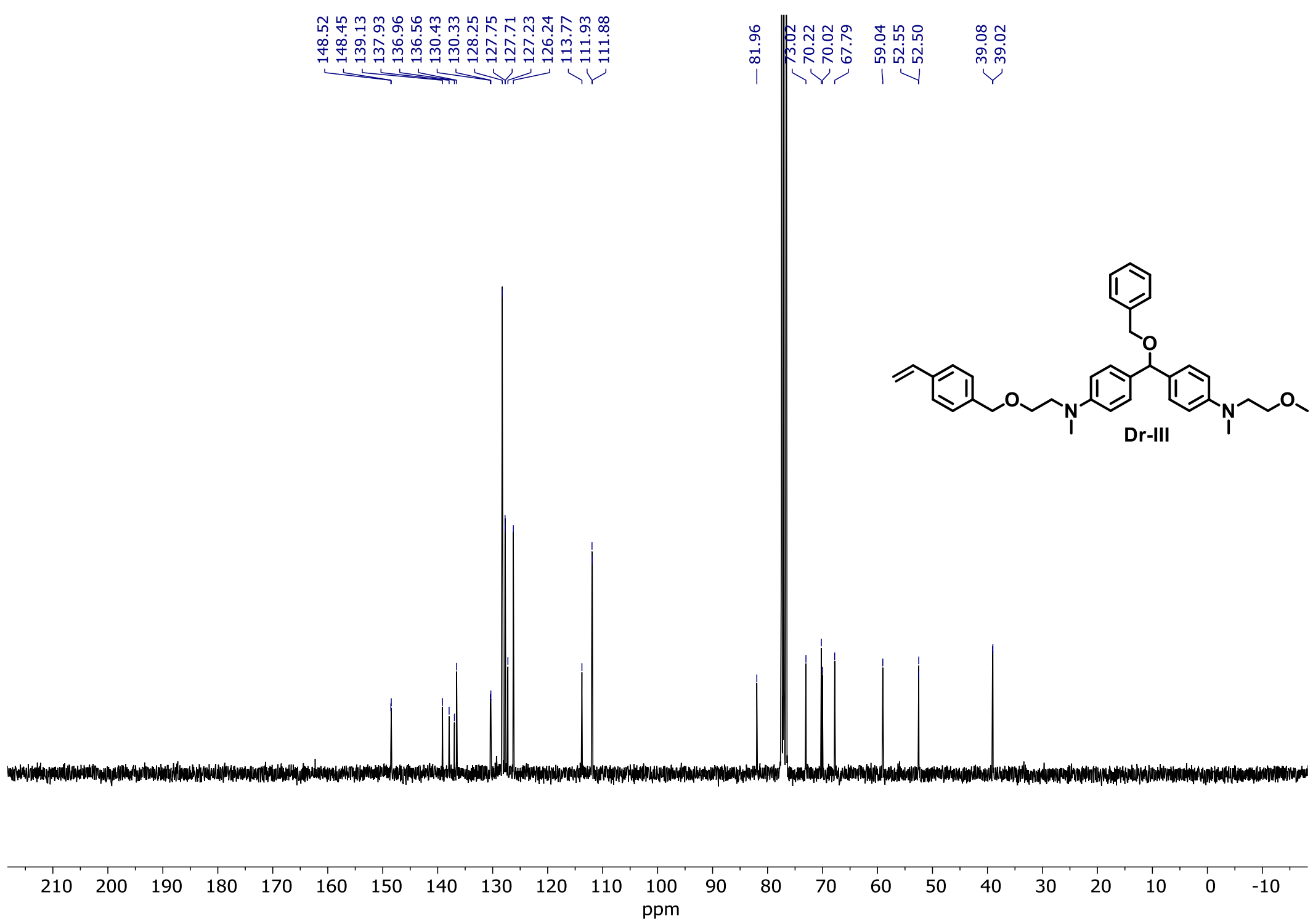

Figure S62: ^{13}C-NMR spectrum of **Dr-III** (75 MHz, $CDCl_3$, 293 K).

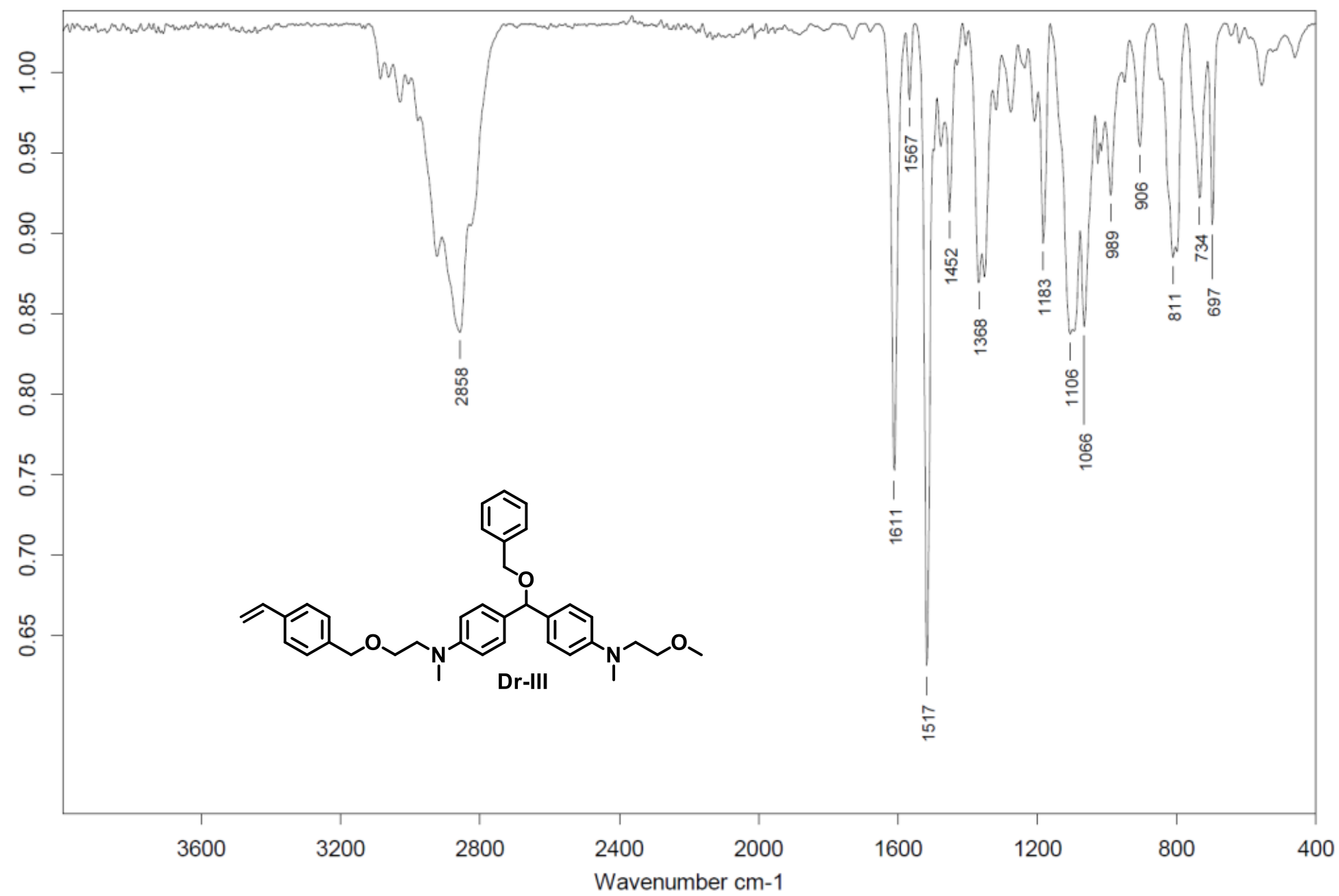

Figure S63: FT-IR spectrum of **Dr-III**.

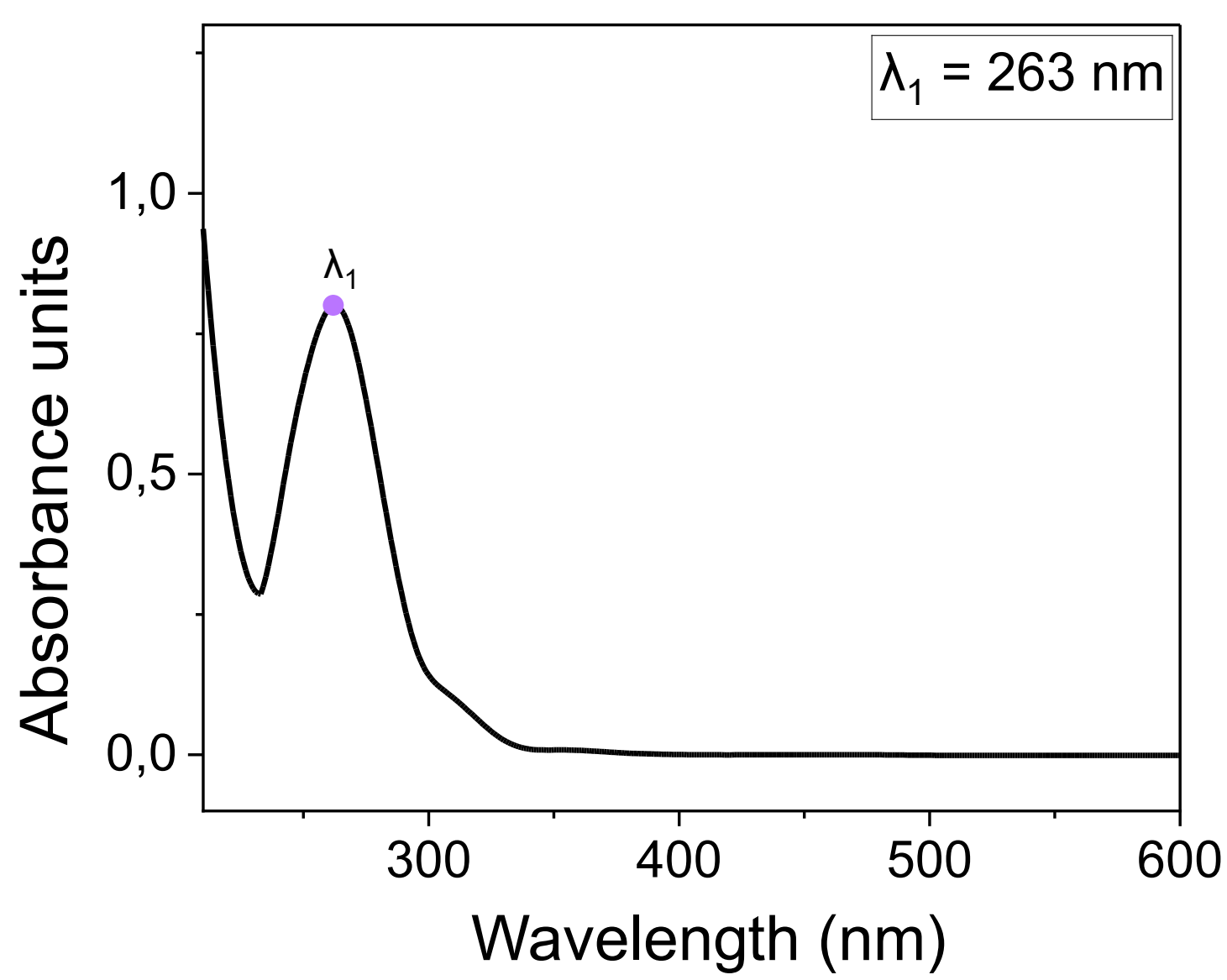

Figure S64: UV-Vis spectrum of **Dr-III** (MeCN, 3.1 × 10^{-5} M, 297 K).

2-benzyl-2-((4-((2-methoxyethyl)(methyl)amino)phenyl)(4-(methyl(2-((4-vinylbenzyl)oxy)ethyl)amino)phenyl)methyl)malononitrile (Dr-IV)

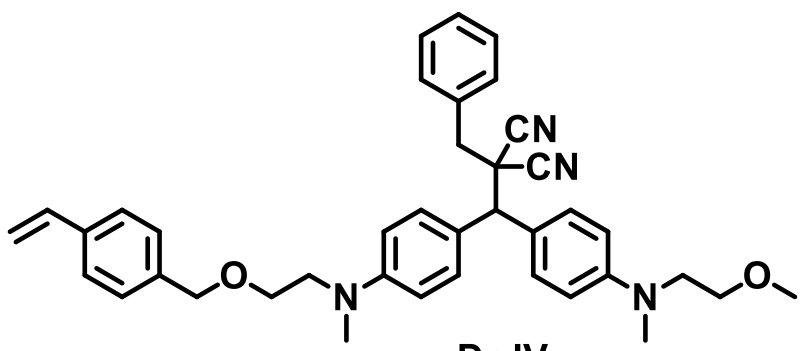

Dr-IV

In a round-bottom flask, sodium hydride (19.1 mg, 0.5×10^{-3} mol, 2.2 eq., 60% dispersion in mineral oil) was suspended in dry DMF (2 mL) at room temperature under stirring. **12** (67.8 mg, 0.4×10^{-3} mol, 2.0 eq.) was added slowly, and the mixture was stirred for 10 min to allow complete deprotonation. A solution of **10** (104 mg, 0.22×10^{-3} mol, 1 eq.) in dry DMF (2 mL) was added dropwise under stirring. Boron trifluoride diethyl etherate ($BF_3\cdot OEt_2$) (46%, 0.07 g, 0.22×10^{-3} mol, 1 eq.) was added slowly via syringe. The reaction mixture was stirred at room temperature until completion. The solvent was removed under reduced pressure, and the crude residue was filtered through a short pad of silica gel. The product was purified by flash column chromatography to afford **Dr-IV** (75 mg, yield: 45%).

^{1}H NMR (300 MHz, $CDCl_3$): δ/ppm 7.46–7.26 (m, 17H, Ar–H), 6.77–6.71 (dd, 1H, CH=, J ≈ 17.5, 10.8 Hz), 5.79–5.72 (dd, 1H, CH_2=, J ≈ 17.5, 1.2 Hz), 5.27–5.23 (dd, 1H, CH_2=, J ≈ 10.8, 1.2 Hz), 4.52 (s, 2H, O–CH_2–Ar), 4.18 (s, 2H, Ar–CH_2–$C(CN)_2$), 3.68–3.56 (m, 8H, –O–CH_2–CH_2–N– ×2), 3.38 (s, 3H, OCH_3), 3.18 (s, 2H, Ph–CH_2–$C(CN)_2$), 3.01 (2s, 6H, N–CH_3).

^{13}C NMR (75 MHz, $CDCl_3$): δ/ppm 148.78, 137.82, 137.00, 136.54, 132.69, 130.43, 129.79, 128.71, 128.49, 127.76, 126.26, 124.93, 115.68, 113.80, 112.25, 73.05, 70.15, 67.69, 59.07, 56.70, 52.38, 45.57, 43.36, 39.00, 38.94.

FT-IR $(cm)^{-1}$: 2872, 1634, 1454, 1372, 1353, 1278, 1208, 1190, 1108, 980, 813, 808, 759, 700, 666, 480.

HR-MS (m/z): $[M+H]^+$ calcd for $C_{39}H_{43}N_4O_2$ 599.3373; found 599.3381

UV-Vis: Extinction coefficients (ε $[L\cdot mol^{-1}\cdot cm^{-1}]$) in the λ maxima ($\lambda_1$) of 3.2×10^{-5} M MeCN solution of **Dr-IV**

λ_1, nm (ε): 273 (32679).

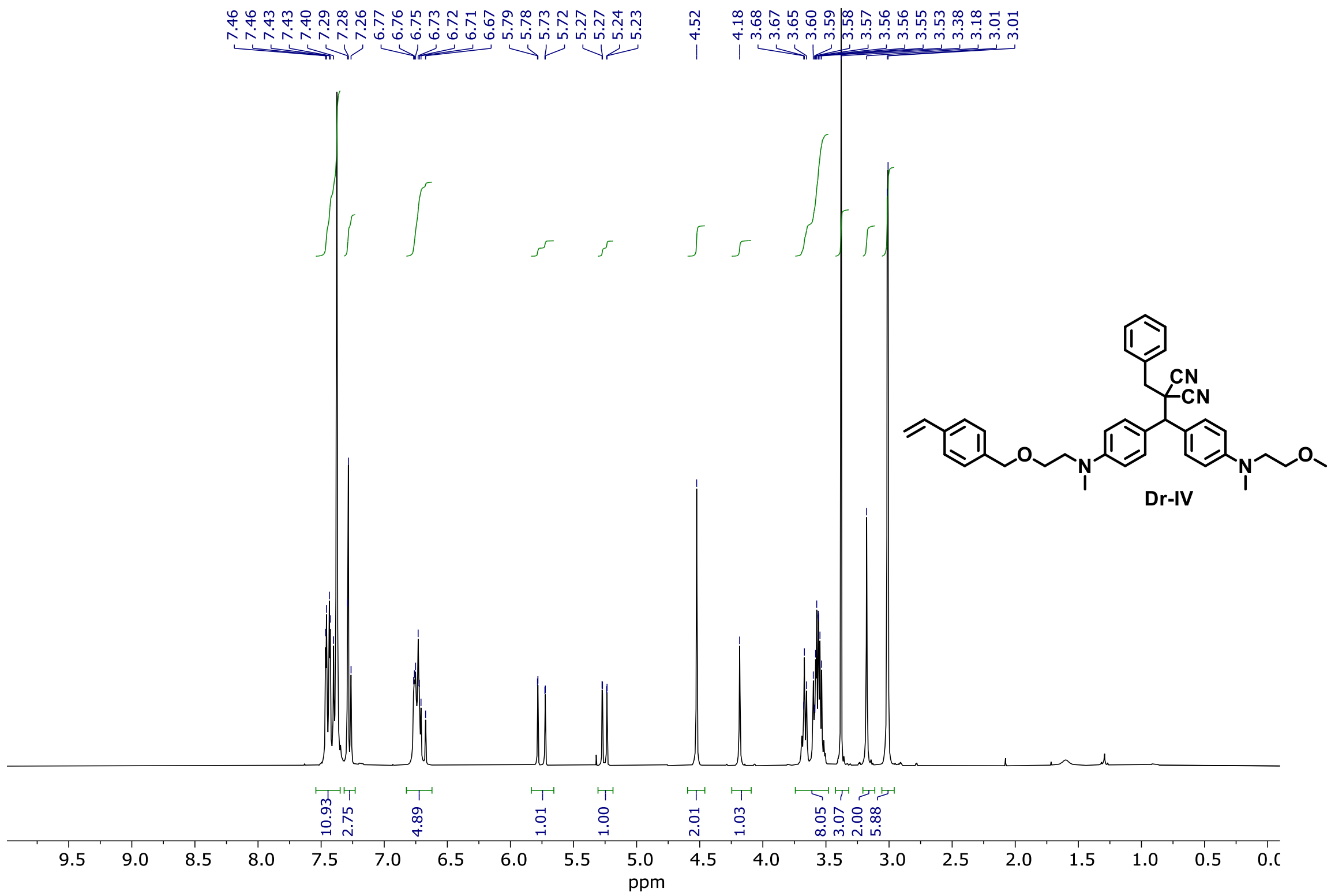

Figure S65: ^{1}H-NMR spectrum of **Dr-IV** (300 MHz, $CDCl_3$, 293 K).

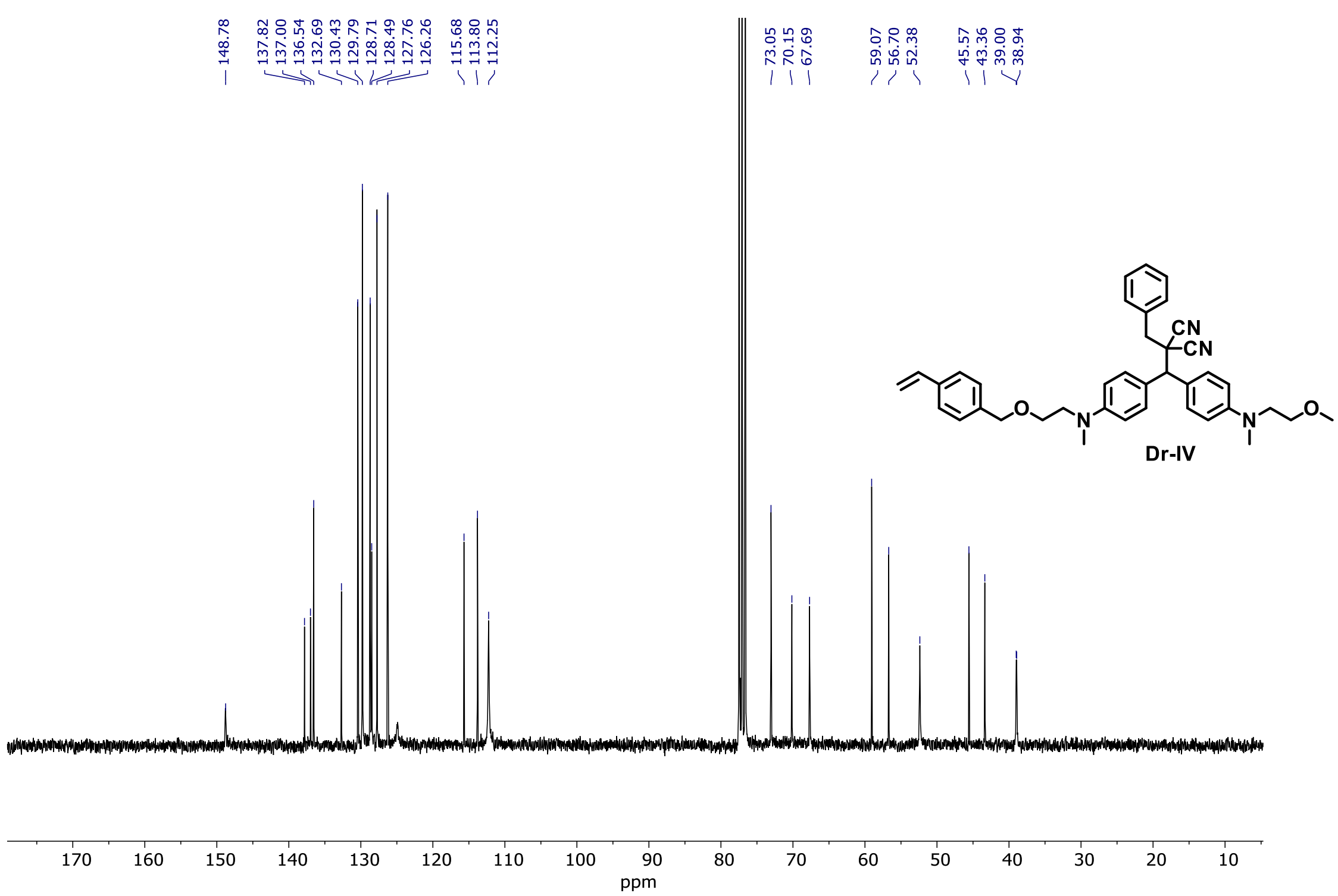

Figure S66: ^{13}C-NMR spectrum of **Dr-IV** (75 MHz, $CDCl_3$, 293 K).

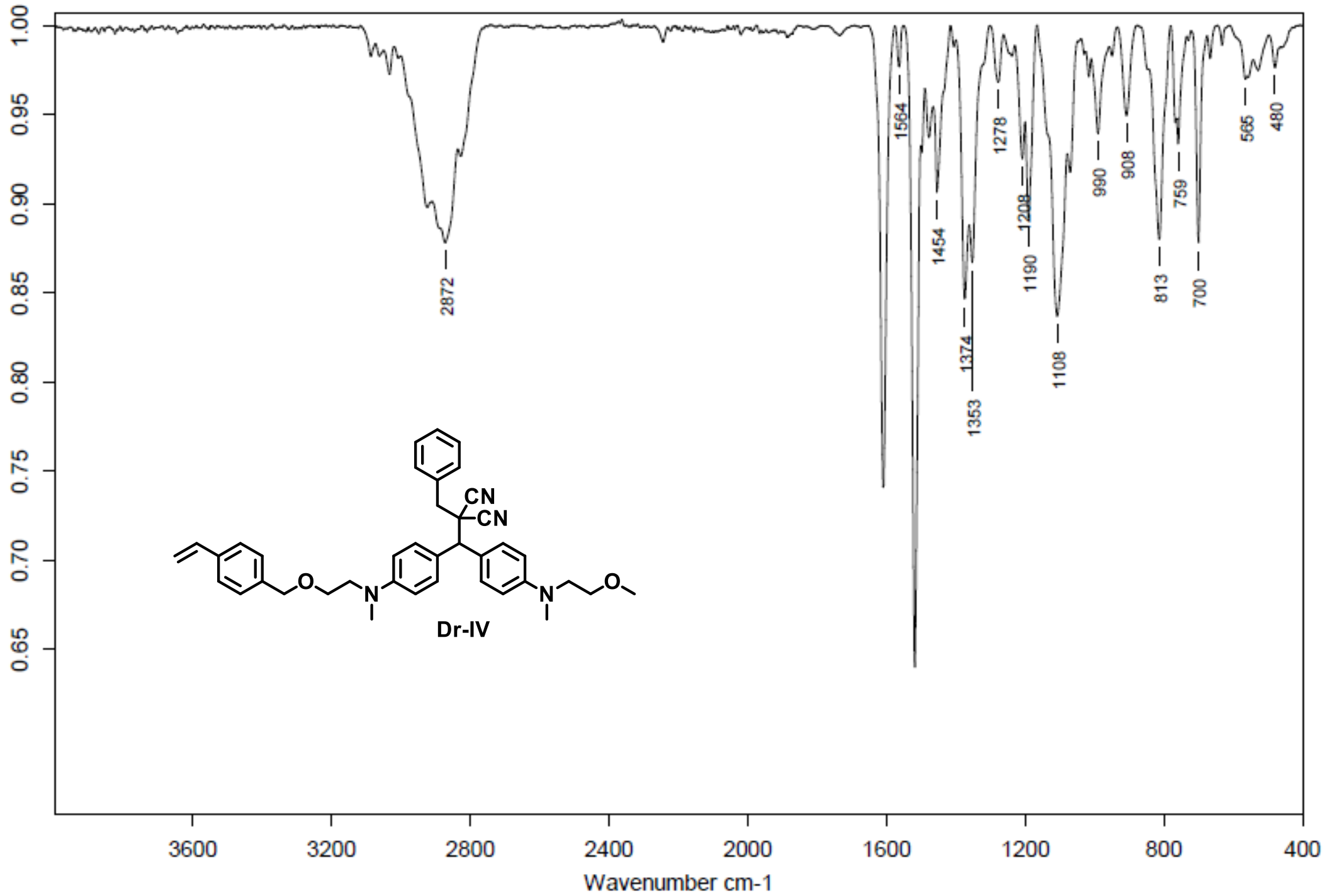

Figure S67: FT-IR spectrum of **Dr-IV**.

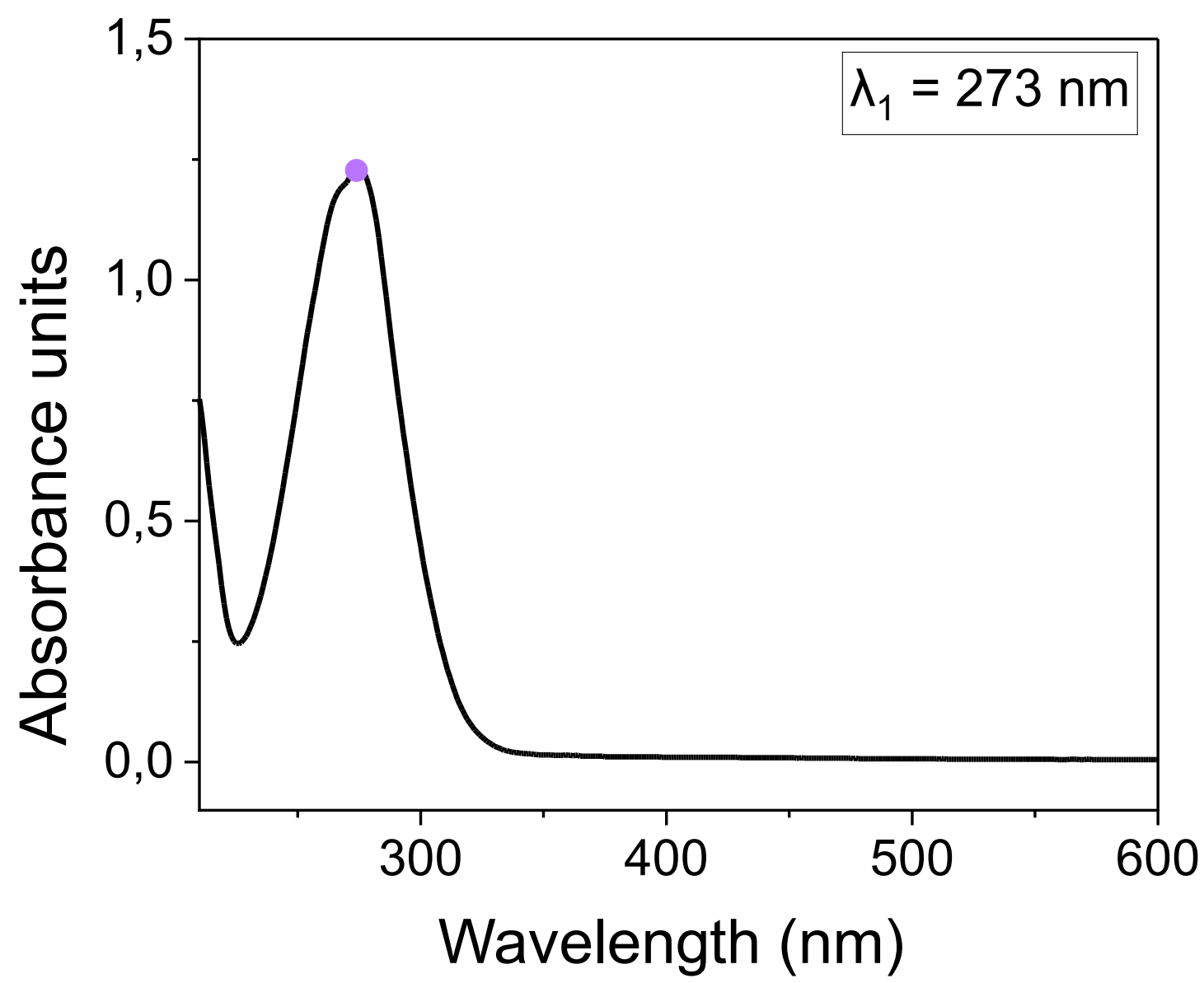

Figure S68: UV-Vis spectrum of **Dr-IV** (MeCN, 3.2×10^{-5} M, 297 K).

1,4-bis((4-vinylbenzyl)oxy)butane (BVBB)

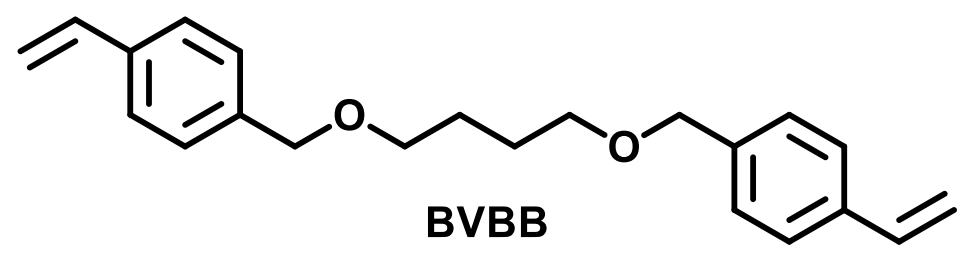

In a round-bottom flask, 1,4-butanediol (1.0 g, 1.1 $\times 10^{-2}$ mol) was dissolved in dry DMF (15 mL) under an inert atmosphere. Sodium hydride (1.1 g, 3.3 $\times 10^{-2}$ mol, 3 eq., 60% w/w dispersion in mineral oil) was added, and the mixture was stirred for 15 min. 1-(Chloromethyl)-4-vinylbenzene (5.1 g, 3.3 $\times 10^{-2}$ mol, 3 eq.) was added, and the reaction mixture was stirred for 12 hours at room temperature. The reaction was quenched with water (150 mL) and extracted with ethyl acetate (3 × 50 mL). The combined organic layers were washed with water (multiple portions) and brine, dried over anhydrous Na_2SO_4, filtered, and concentrated under reduced pressure. The crude product was purified by flash column chromatography to afford **BVBB** (3.0 g, yield: 84%) as a white crystalline solid.

^{1}H NMR (400 MHz, $CDCl_3$): δ/ppm 7.42–7.30 (m, 8H, Ar–H), 6.77–6.70 (m, 2H, CH=), 5.79 (d, *J* = 17.6 Hz, 2H, CH_2=, trans), 5.24 (d, *J* = 10.9 Hz, 2H, CH_2=, cis), 4.50 (s, 4H, Ar–CH_2–O), 3.52–3.49 (m, 4H, –O–CH_2–), 1.74–1.71 (m, 4H, –CH_2–CH_2).

^{13}C NMR (100 MHz, $CDCl_3$): δ/ppm 138.27, 136.88, 136.59, 127.81, 126.21, 113.68, 72.58, 70.10, 26.53.

FT-IR $(cm)^{-1}$: 2938, 2851, 1652, 1585, 1518, 1465, 1397, 1357, 1326, 1262, 1189, 1160, 1109, 1089, 1038, 985, 901, 865, 825, 798, 738, 696, 562, 482.

HR-MS (m/z): $[M+Na]^+$ calcd for $C_{22}H_{26}NaO_2$ 345.1825; found 345.1827

UV-Vis: Extinction coefficients (ε [$L \cdot mol^{-1} \cdot cm^{-1}$]) in the λ maxima ($\lambda_1$) of 3.5 × 10^{-5} M MeCN solution of **BVBB.**

λ_1, nm (ε): 238 (14285).

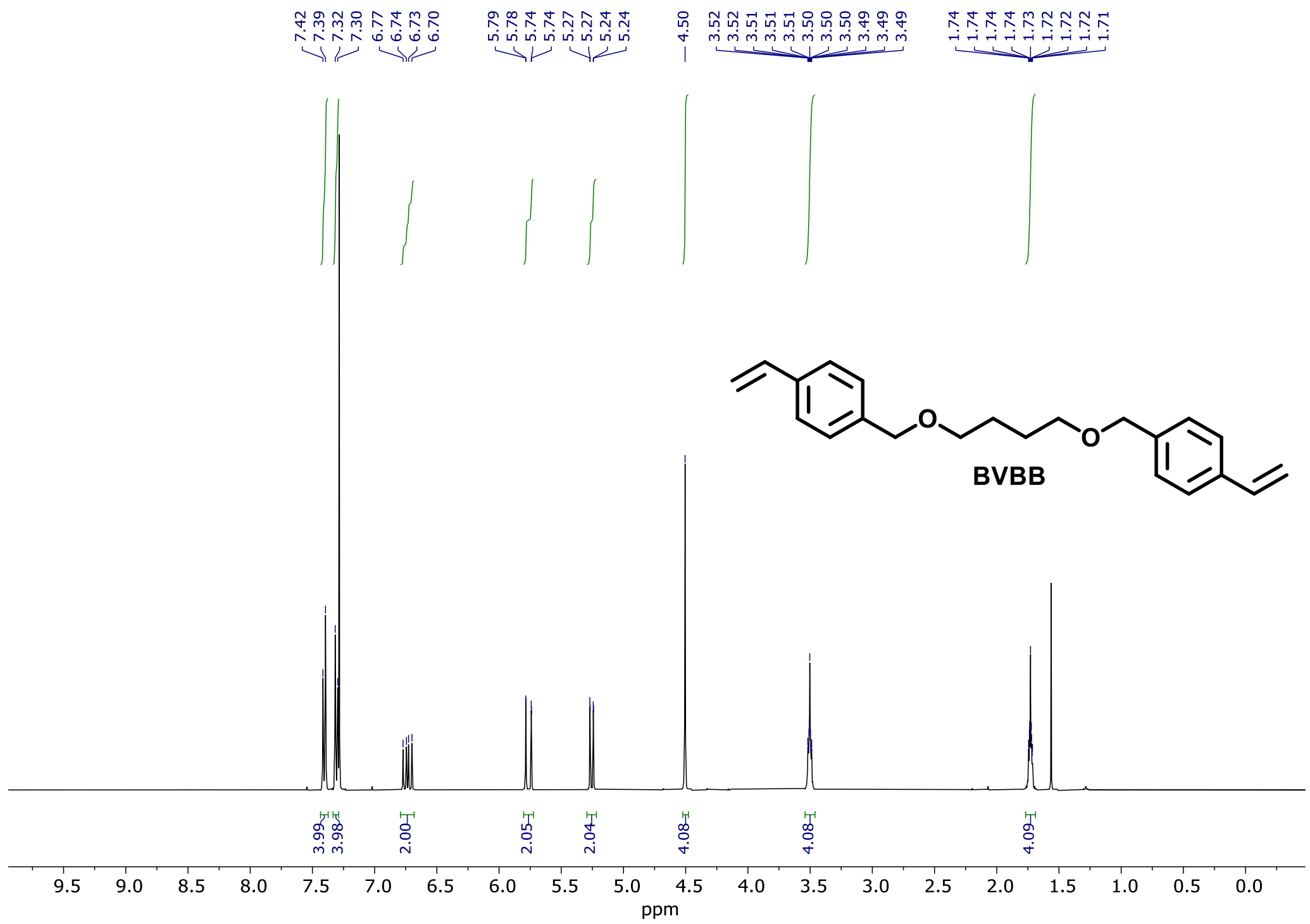

Figure S69: ^{1}H-NMR spectrum of **BVBB** (400 MHz, $CDCl_3$, 293 K).

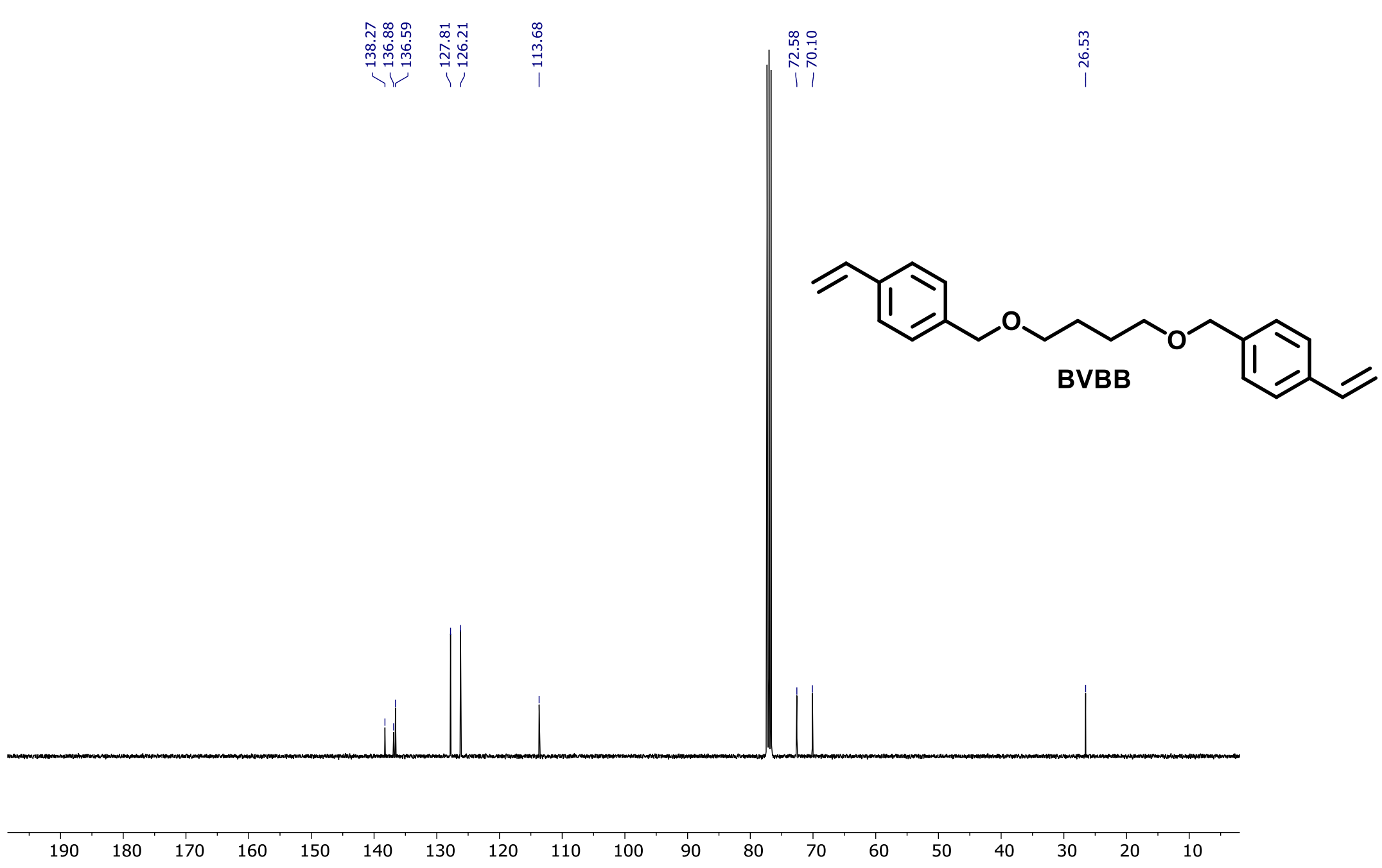

Figure S70: ^{13}C-NMR spectrum of **BVBB** (100 MHz, $CDCl_3$, 293 K).

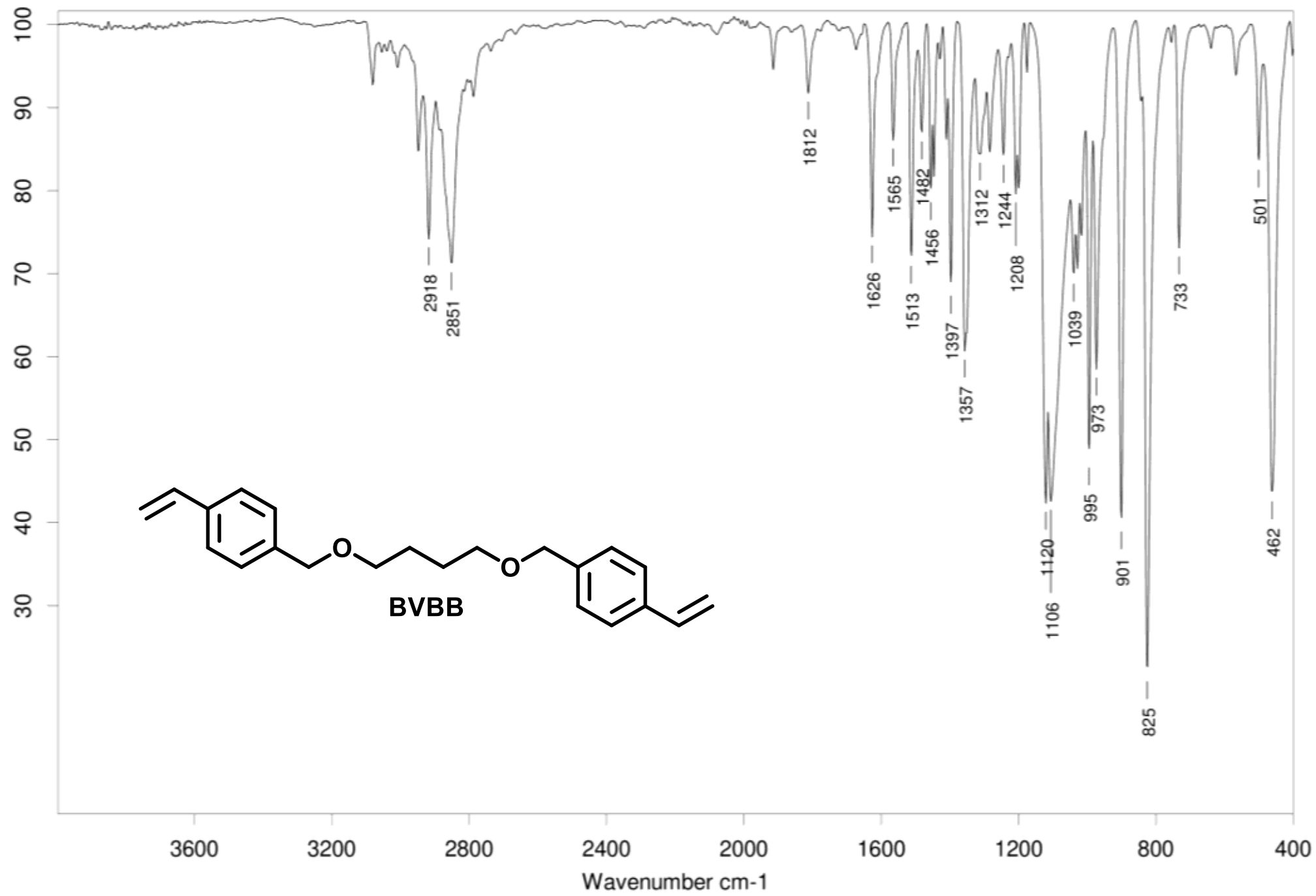

Figure S71: FT-IR spectrum of **BVBB**.

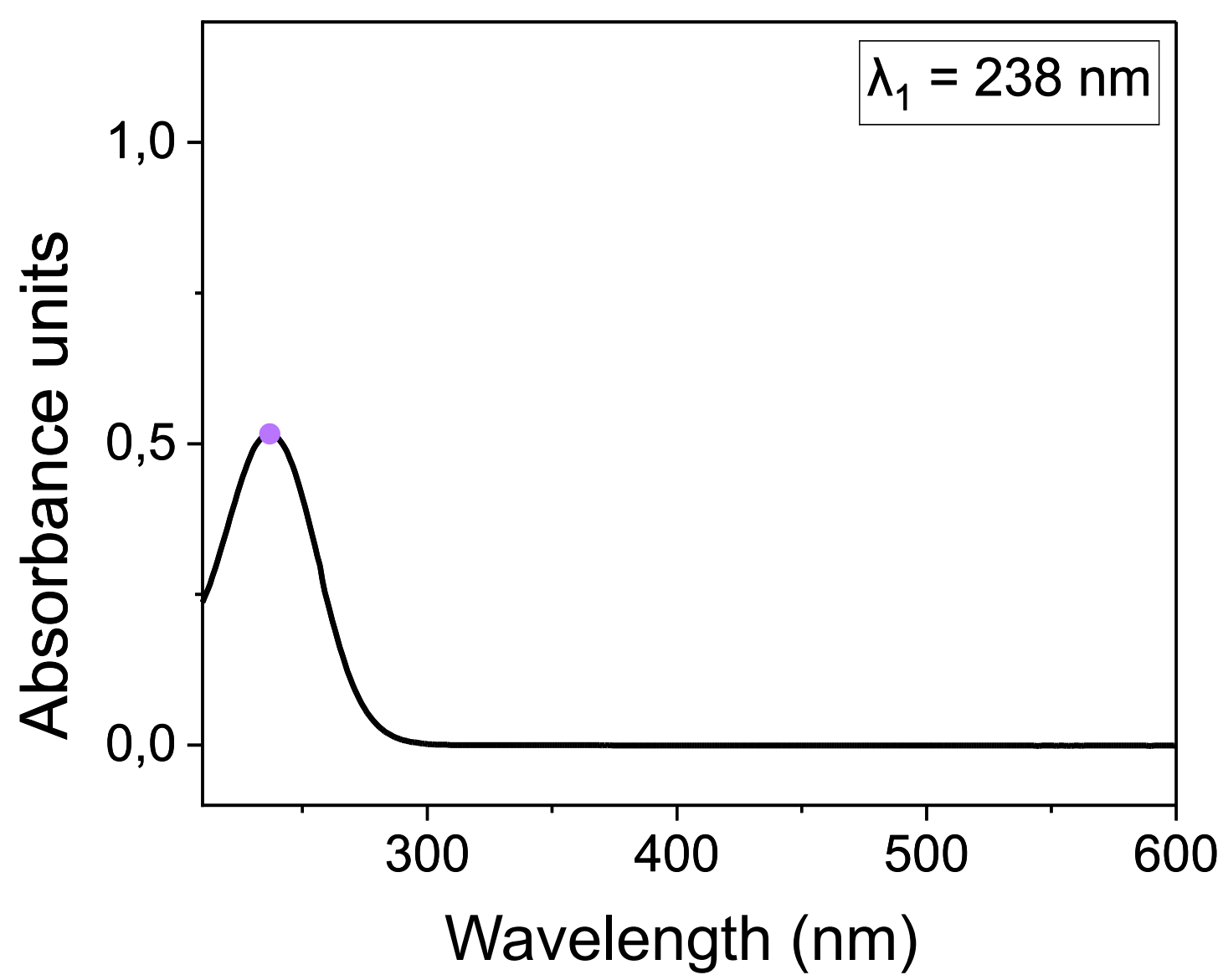

Figure S72: UV-Vis spectrum of **Dr-BVBB** (MeCN, 3.5 × 10^{-5} M, 297 K).

^{1}H NMR spectra and UV-Vis spectra of carbocations in solution

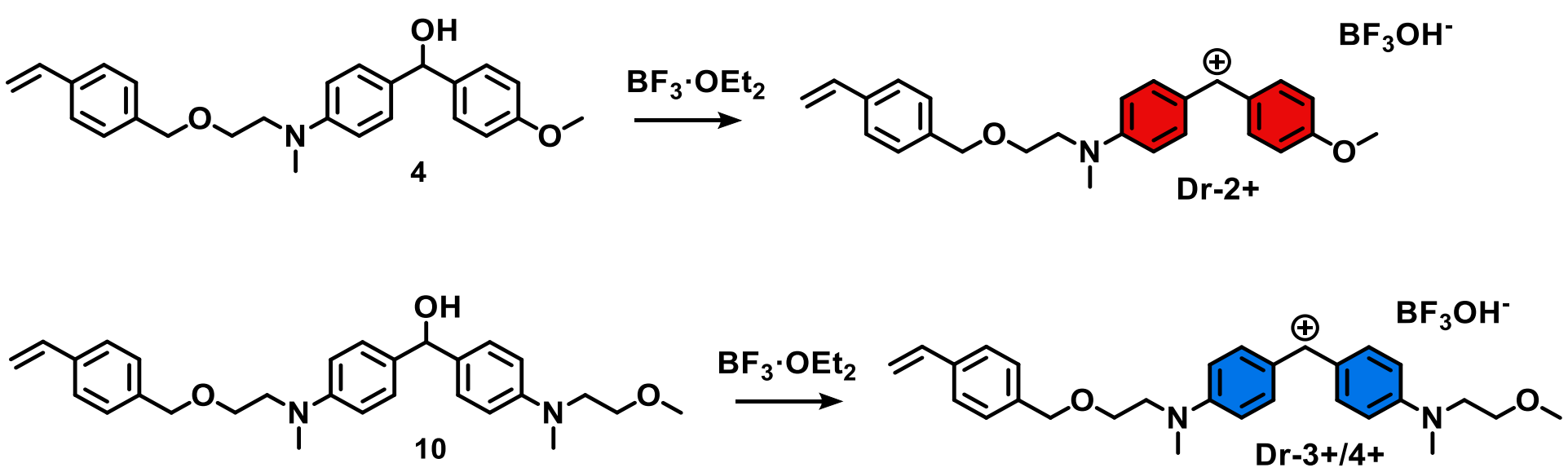

Scheme S4: Preparation of **Dr-2**$^+$ and **Dr-3**$^+$**/4**$^+$ from their respective carbinols **4** and **10**.

To further support the assignment of the mechanically generated colored species as diarylcarbenium ions, the corresponding carbocations were independently generated in solution by treatment of the carbinol precursors with boron trifluoride diethyl etherate ($BF_3 \cdot OEt_2$). Due to its limited stability, **Dr-2**$^+$ was generated in situ and immediately analyzed by ^{1}H NMR. In contrast, the higher stability of **Dr-3**$^+$/**Dr-4**$^+$ allowed their isolation prior to NMR characterization. Formation of the carbocations was confirmed by the characteristic downfield shift of the benzylic proton, from 5.77 to 7.97 for **Dr-2**$^+$, and from 5.70 ppm to 7.78 ppm for **Dr-3**$^+$/**Dr-4**$^+$and the neighboring aromatic resonances. The UV–Vis absorption spectra of these solution-generated carbocations are also reported.

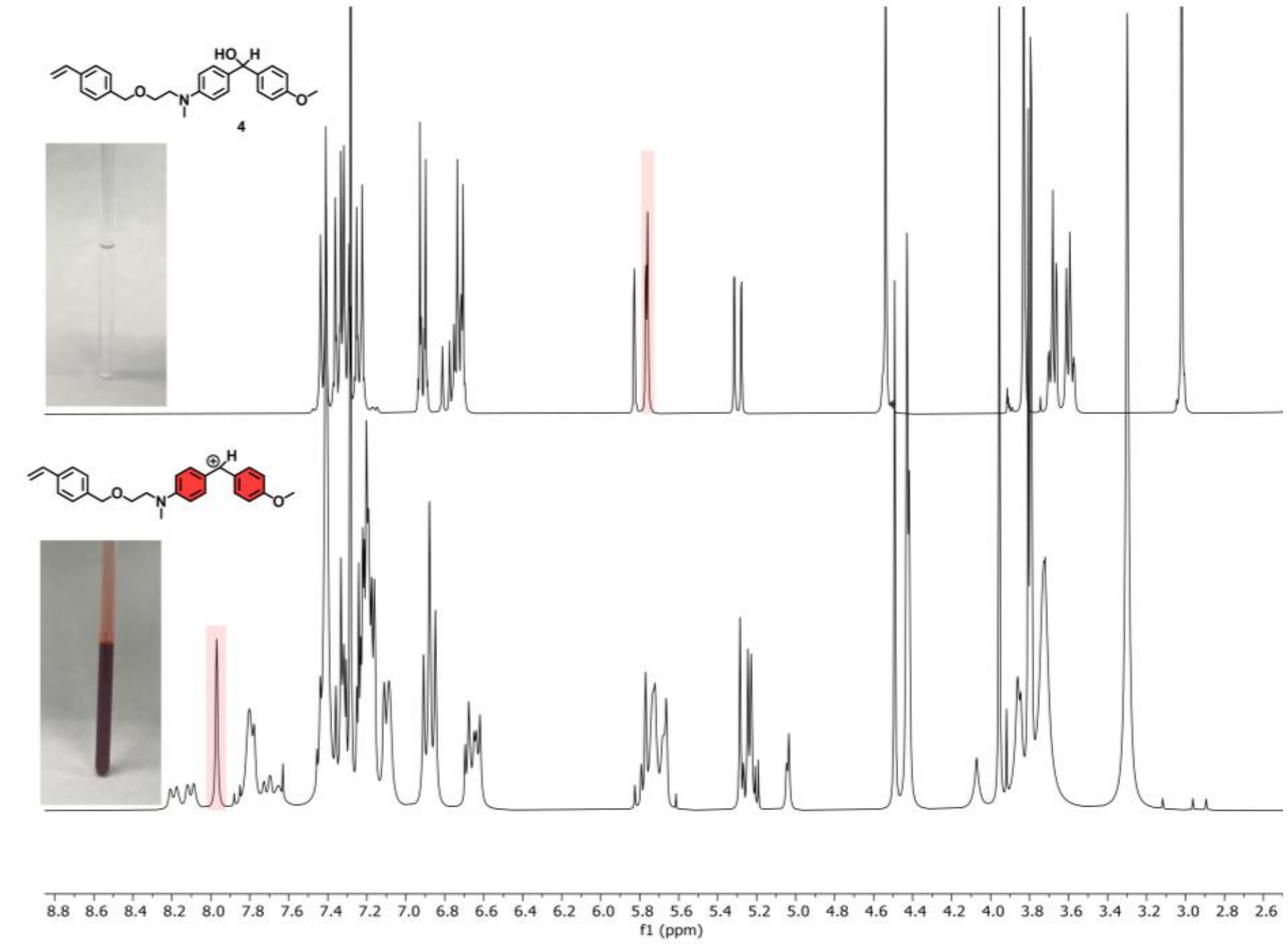

Figure S73: Comparison of the ^{1}H NMR spectra ($CDCl_3$, 300 MHz) of the neutral carbinol precursor **4** (top) and the corresponding carbocation **Dr2^{+}** generated in situ upon treatment with $BF_3 \cdot OEt_2$ (bottom).

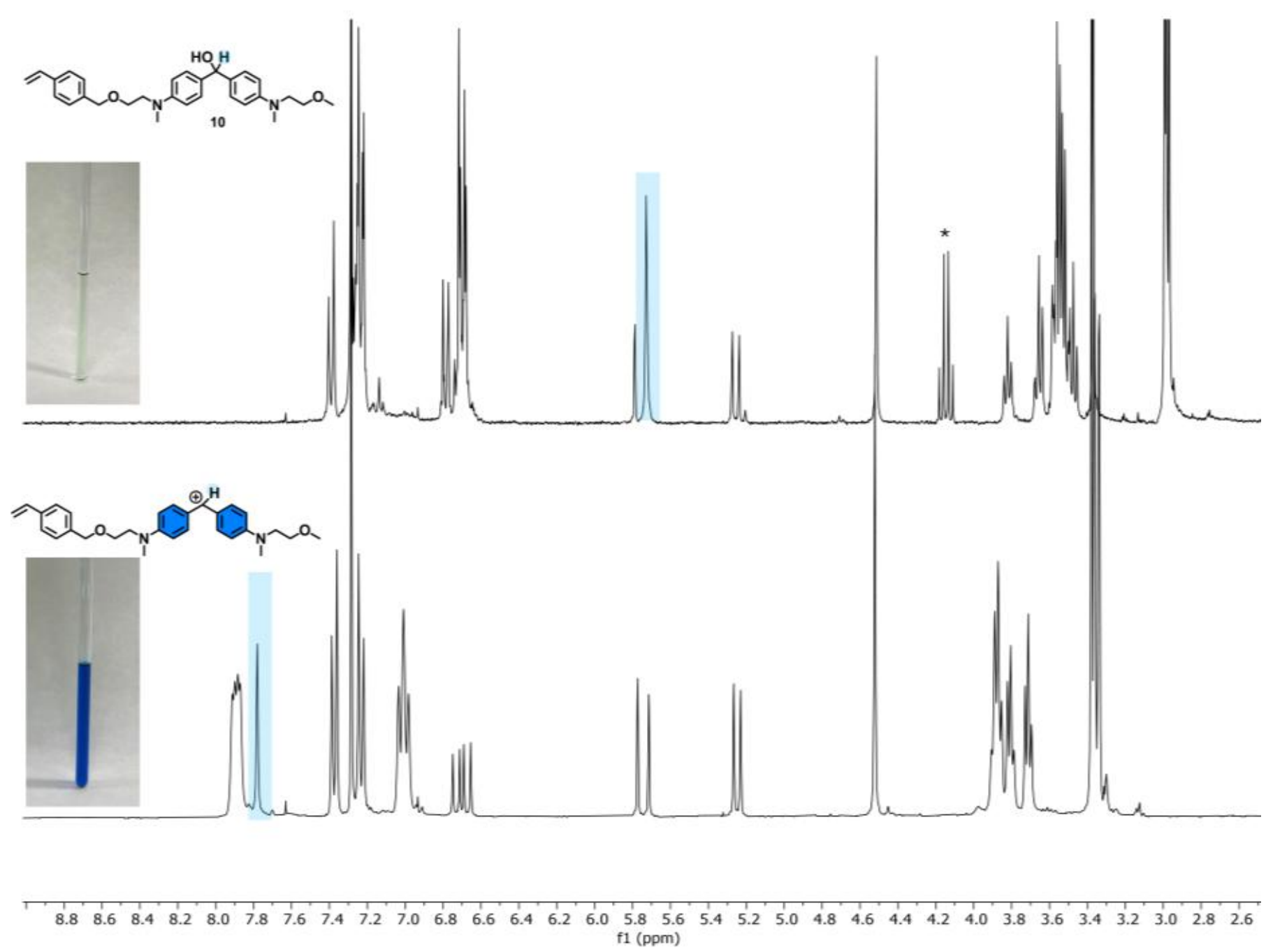

Figure S74: Comparison of the ^{1}H NMR spectra ($CDCl_3$, 300 MHz) of the neutral carbinol precursor **10** (top) and the corresponding isolated carbocation **Dr3^{+}/Dr4^{+}** upon treatment with $BF_3 \cdot OEt_2$ (bottom). Signals marked by the asterisk are related to traces of EtOAc (q, δ 4.12).

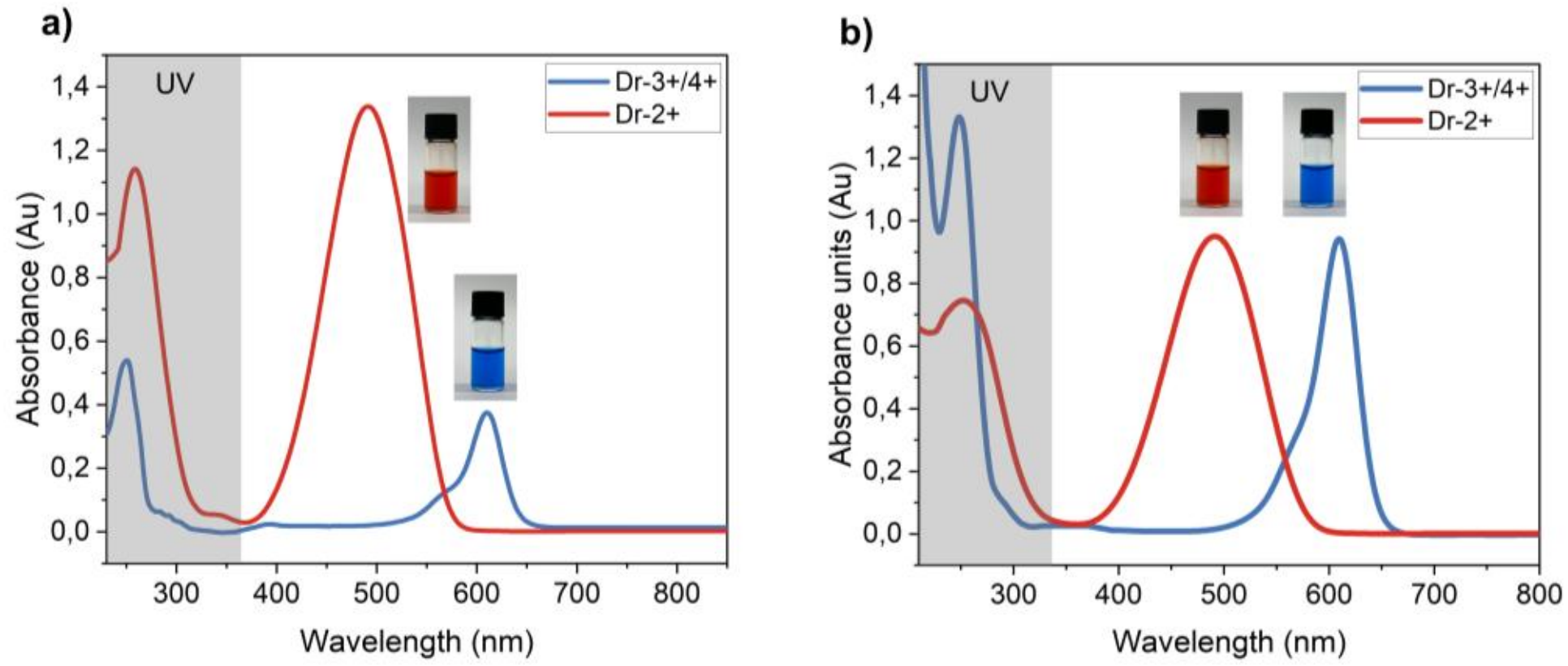

Figure S75: (a) UV-Vis spectra and (b) normalized UV-Vis spectra of MeCN solutions of **Dr-2**$^+$ (3.7×10^{-5} M) and **Dr-3**$^+$/**4**$^+$ (3.6×10^{-6} M).

Extinction coefficients (ε [$L \cdot mol^{-1} \cdot cm^{-1}$]) determined for **Dr-2**$^+$ and **Dr-3**$^+$/**4**$^+$ at their respective absorption maxima (λ_{max}) in MeCN solutions (3.7×10^{-5} M for **Dr-2**$^+$, and 3.6×10^{-6} M for **Dr-3**$^+$/**4**$^+$).

Dr-2$^+$ λ_{max}, nm (ε): 490 (35486).

Dr-3$^+$/**Dr-4**$^+$ λ_{max}, nm (ε): 610 (97222)

Synthesis of polymer networks

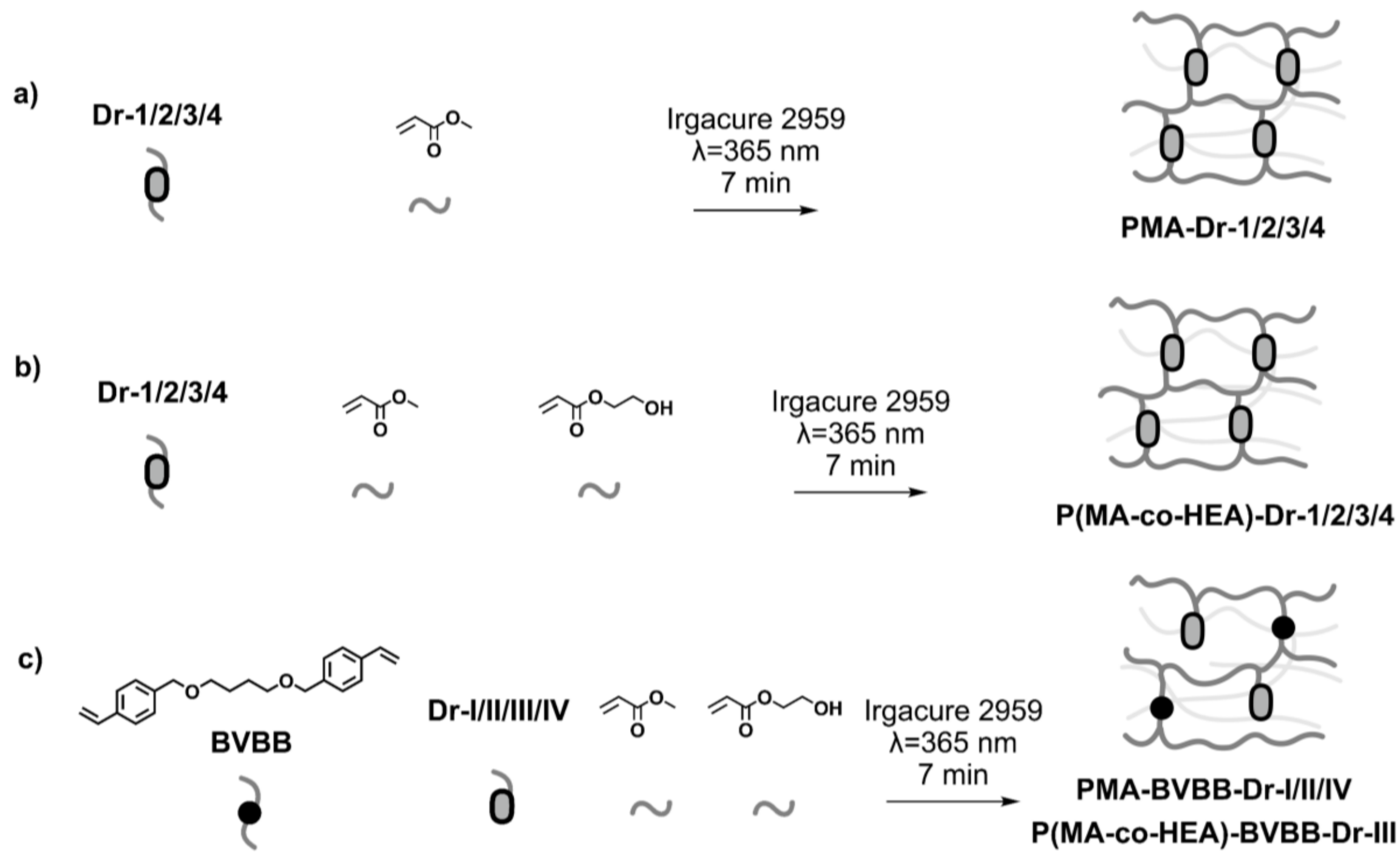

Scheme S5: (a) Reaction scheme for the PMA network series. (b) Reaction scheme for the P(MA-*co*-HEA) network series. (c) Reaction scheme for the control polymers series with the non-mechanoresponsive crosslinker BBVB

Procedure for polymerization of PMA-Dr-1–4. In a 4 mL vial, Irgacure 2959 (1.6 mg, 7.0 $\times10^{-6}$ mol) and the chosen mechanophore (0.15 $\times10^{-3}$ mol (1mol%); **Dr-1**: 78.0 mg, **Dr-2**: 86.5 mg, **Dr-3**: 85.2 mg, **Dr-4**: 93.7 mg) were combined with methyl acrylate (MA) (1.37 mL, 1.15 g, 15 $\times10^{-3}$ mol). The vial was fitted with a semi-permeable Teflon® cap, and the mixture was bubbled with nitrogen for 5 minutes. Two glass microscope slides were prepared by covering one side of each with clear adhesive tape. Folded pieces of parafilm were used as spacers and positioned between the slides to define the film thickness (approximately 250 µm). The slides were then clamped together using office clamps, and the monomer solution was introduced into the gap using a pipette. The assembly was placed in a UV light box equipped with 365 nm LEDs (Dr. Hönle LED Cube) and irradiated until polymerization was complete. The polymerization was performed using a sequence of six 20-second bursts at 50% power, with 5 seconds pause between each burst, followed by ten 30-second bursts at 20% power, with 5 seconds pause between each burst. Afterward, the slides were carefully separated, and the resulting polymer film was transferred to a vacuum oven and dried overnight at 60 °C.

Procedure for polymerization of P(MA-*co*-HEA)-Dr-1–4. In a 4 mL vial, Irgacure 2959 (1.6 mg, 7.0 ×10⁻⁶ mol) and the chosen mechanophore (0.15 ×10⁻³ mol; **Dr-1**: 78.0 mg, **Dr-2**: 86.5 mg, **Dr-3**: 85.2 mg, **Dr-4**: 93.7 mg) were combined with methyl acrylate (1.20 mL, 1.15 g, 13.5 ×10⁻³ mol) and 2-hydroxyethyl acrylate (HEA) (0.16 mL, 0.17 g, 01.5 ×10⁻³ mol). The vial was fitted with a semi-permeable Teflon® cap, and the mixture was bubbled with nitrogen for 5 minutes. Two glass microscope slides were prepared by covering one side of each with clear adhesive tape. Folded pieces of parafilm were used as spacers and positioned between the slides to define the film thickness (approximately 250 µm). The slides were then clamped together using office clamps, and the monomer solution was introduced into the gap using a pipette. The assembly was placed in a UV light box equipped with 365 nm LEDs (Dr. Hönle LED Cube) and irradiated until polymerization was complete. The polymerization was performed using a sequence of six 20-second bursts at 50% power, with 5 seconds pause between each burst, followed by ten 30-second bursts at 20% power, with 5 seconds pause between each burst. Afterward, the slides were carefully separated, and the resulting polymer film was transferred to a vacuum oven and dried overnight at 60 °C.

Procedure for polymerization of PMA-Dr-I/II/IV. In a 4 mL vial, Irgacure 2959 (1.6 mg, 7.0 ×10⁻⁶ mol) and the chosen diarylmethane derivative (0.15 ×10⁻³ mol; **Dr-I**: 78.0 mg, **Dr-III:** 85.2 mg, **Dr-IV**: 93.7 mg) were combined with methyl acrylate (1.37 mL, 1.15 g, 15 ×10⁻³ mol) and **BVBB** (48.4 mg, 0.15 ×10⁻³ mol, 1 wt%). The vial was fitted with a semi-permeable Teflon® cap, and the mixture was bubbled with nitrogen for 5 minutes. Two glass microscope slides were prepared by covering one side of each with clear adhesive tape. Folded pieces of parafilm were used as spacers and positioned between the slides to define the film thickness (approximately 250 µm). The slides were then clamped together using office clamps, and the monomer solution was introduced into the gap using a pipette. The assembly was placed in a UV light box equipped with 365 nm LEDs (Dr. Hönle LED Cube) and irradiated until polymerization was complete. The polymerization was performed using a sequence of six 20-second bursts at 50% power, with 5 seconds pause between each burst, followed by ten 30-second bursts at 20% power, with 5 seconds pause between each burst. Afterward, the slides were carefully separated, and the resulting polymer film was transferred to a vacuum oven and dried overnight at 60 °C.

Procedure for polymerization of P(MA-*co*-HEA)-Dr-III. In a 4 mL vial, Irgacure 2959 (1.6 mg, 7.0 ×10⁻⁶ mol) and **Dr-III** (86.5 mg, 0.15 ×10⁻³ mol) were combined with methyl acrylate (1.20 mL, 1.15 g, 13.5 ×10⁻³ mol), 2-hydroxyethyl acrylate (0.16 mL, 0.17 g, 1.5 ×10⁻³ mol), and **BVBB** (48.4 mg, 0.15 ×10⁻³ mol, 1 wt%). The vial was fitted with a semi-permeable Teflon® cap, and the mixture was bubbled with nitrogen for 5 minutes. Two glass microscope slides were prepared by covering one side of each with clear adhesive tape. Folded pieces of parafilm were used as spacers and positioned between the slides to define the film thickness (approximately 250 µm). The slides were then clamped together using office clamps, and the monomer solution was introduced into the gap using a pipette. The

assembly was placed in a UV light box equipped with 365 nm LEDs (Dr. Hönle LED Cube) and irradiated until polymerization was complete. The polymerization was performed using a sequence of six 20-second bursts at 50% power, with 5 seconds pause between each burst, followed by ten 30-second bursts at 20% power, with 5 seconds pause between each burst. Afterward, the slides were carefully separated, and the resulting polymer film was transferred to a vacuum oven and dried overnight at 60 °C.

Swelling index determination

Sample preparation

Polymer specimens were dried under vacuum at 60 ºC for 24 hours. Then, they were cut into rectangles approximately 5 × 10 mm with a mass of 15–20 mg. Each sample was accurately weighed to obtain the initial dry mass (m_0). All measurements were performed in five replicates. Swelling experiments were conducted only on the main network formulations, and control samples were excluded from this analysis.

Swelling experiment

Samples were immersed in 2 mL of tetrahydrofuran (THF), selected as a good solvent for the corresponding uncross-linked polymer, in 4 mL sealed vials at room temperature. The specimens were allowed to swell without agitation for 24 h to reach equilibrium. After this time, the swollen samples were quickly removed from the solvent, gently blotted with filter paper to remove surface solvent, and immediately weighed to obtain the swollen mass (m_1). The swelling index (SI) was calculated as:

$$SI = (m_1 - m_0) / m_0 \times 100\% \qquad \text{eq. 1}$$

Gel fraction determination

After swelling, the samples were dried under vacuum at 60 ºC for 24 hours to remove residual solvent. The final dry mass was recorded as (m_2), corresponding to the mass of polymer incorporated in the network. The gel content (GC) was calculated as:

$$GC = (m_2 / m_0) \times 100\% \qquad \text{eq. 2}$$

Table S1: Table for the swelling index (SI) and gel content (GC) of the PMA and P(MA-*co*-HEA) crosslinked polymer networks.

**Polymer**	SI (%)	GC (%)
**PMA-Dr-1**	186±10	97±1
**PMA-Dr-2**	190±12	97±2
**PMA-Dr-3**	188 ± 8	98±1
**PMA-Dr-4**	196±8	97±1
**P(MA-*co*-HEA)-Dr-1**	310 ± 12	96±1
**P(MA-*co*-HEA)-Dr-2**	284 ± 11	98±2
**P(MA-*co*-HEA)-Dr-3**	298±15	97±3
**P(MA-*co*-HEA)-Dr-4**	271±12	98±1

Mechanoresponsive behavior of PMA and P(MA-*co*-HEA) networks under tensile deformation

As mentioned in the main text, **PMA-Dr-1** exhibits both mechanical and photoactivation. This dual behavior arises from the technique used to assess fluorescence, which employs an LED light source that can also activate the mechanophore via irradiation. To decouple the two activation pathways, control experiments were carried out on a **PMA-BVBB-Dr-I** crosslinked network in which **Dr-I**, i.e., the **Dr-1** control molecule, was incorporated as a pendent group. In this configuration, mechanical force cannot be transmitted to the mechanophore because it lacks a second connection to the polymer backbone, leaving only light irradiation as the activation mechanism. Tensile experiments on this control system clearly showed activation over time, as monitored by the evolution of Q, i.e., the ratio of emission intensities at 490 nm and 585 nm. As shown in Figure S33, activation was confined to the irradiated region, while the rest of the polymer remained non-activated after stretching. The maximum normalized intensity of the emission band in this control experiment reached 2, whereas it reached up to 6 in pulled **PMA-Dr-1**. These results suggest that although photoactivation during fluorescence measurement can contribute to the signal, the predominant cause of activation in **PMA-Dr-1** is mechanical force.

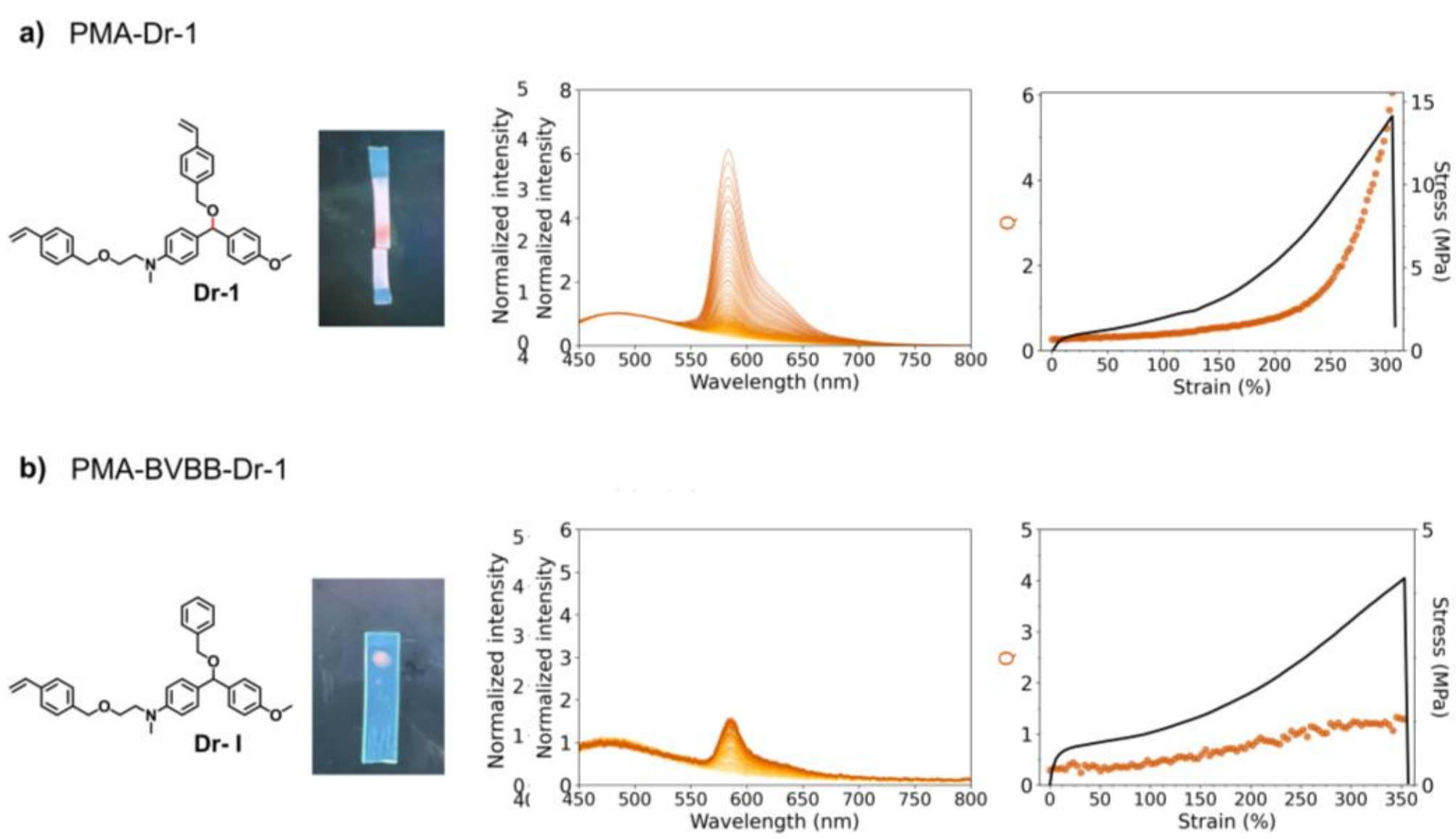

Figure S76**:** Photoactivation of **PMA-Dr-1** and **PMA-Dr-I** during tensile deformation (strain rate 2.2% s^{-1} or 375 µm s^{-1}).

Furthermore, the stability of the mechanically generated radicals was evaluated by monitoring the fluorescence decay over time under ambient atmosphere and under argon (Figure S77). While the fluorescence of the air-exposed samples rapidly disappeared within approximately 10 min (Figure S77 top), a significantly slower decay was observed for the

samples degassed for 3 h prior to stretching and maintained under an argon atmosphere (Figure S77 bottom). These observations are consistent with the well-known oxygen sensitivity of diarylmethyl radicals and support oxygen-mediated quenching as the dominant deactivation pathway.[15]

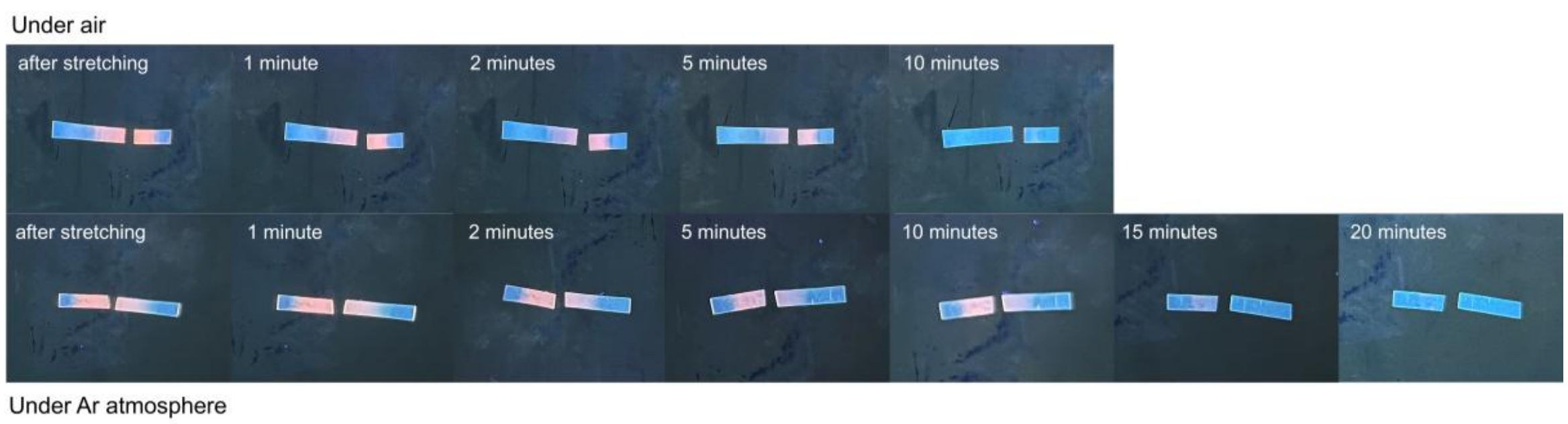

Figure S77: Time-dependent fluorescence decay of mechanically activated **PMA-Dr-1** films under ambient atmosphere (top) and under an argon atmosphere (bottom).

Both **PMA-Dr-2** and **P(MA-*co*-HEA)-Dr-2** exhibited heterolytic cleavage under mechanical stress. Treating a 3.7 × 10^{-5} M MeCN solution of **Dr-2** with *p*-toluenesulfonic acid showed an intense red color due to the formation of the related carbocation, as confirmed by ^{1}H NMR (Figure S73). Notably, the color observed in solution matches that of the stretched films, supporting the hypothesis that heterolytic cleavage also occurs in the polymer matrices under mechanical stress.

P(MA-*co*-HEA)-Dr-3, **PMA-Dr-4**, and **P(MA-*co*-HEA)-Dr-4** also exhibited signatures of heterolytic cleavage upon mechanical activation. To further support this observation, we note that treating a 3.6 × 10^{-6} M MeCN solution of **Dr-4** with *p*-toluenesulfonic acid provides a blue coloration, easily attributed to the formation of the relative diarylcarbenium ion through ^{1}H NMR (Figure S74). This color closely matches that observed in the stretched polymer films.

In **PMA-Dr-2**, the red coloration disappears within a few seconds after unloading, whereas the blue coloration observed for **P(MA-*co*-HEA)-Dr-3** and **PMA-Dr-4** gradually fades over the course of approximately 1 hour and 12 hours, respectively, under ambient conditions Nevertheless, the loading–unloading cycle can typically be repeated two to three times before sample failure, as only a fraction of the mechanophores are activated during each cycle, leaving intact mechanophores available for subsequent activation.

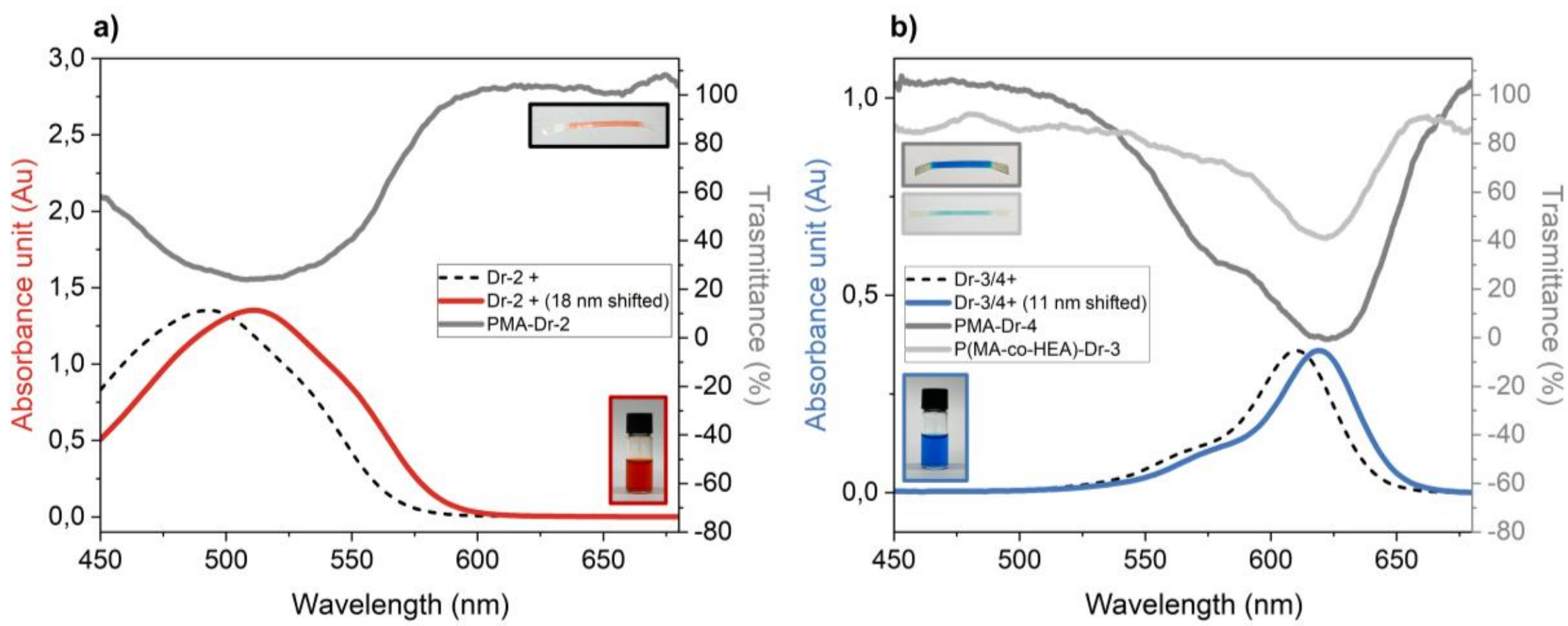

Figure S78: Comparison between (a) the UV-Vis spectrum of **Dr-2**$^+$ and the transmittance spectrum of uniaxially stretched **PMA-Dr-2** film before rupture (strain rate 30%·s^{-1} or 5000 μm s^{-1}) and (b) the UV-Vis spectrum of **Dr-3**$^+$**/4**$^+$ and the transmittance spectrum of uniaxially stretched **P(MA-*co*-HEA)-Dr-3** and **PMA-Dr-4** films before rupture (strain rate 2.2%·s^{-1} or 375 μm s^{-1}). The slight shift of a few nanometers is due to slightly different conditions encountered between the solution and solid states.

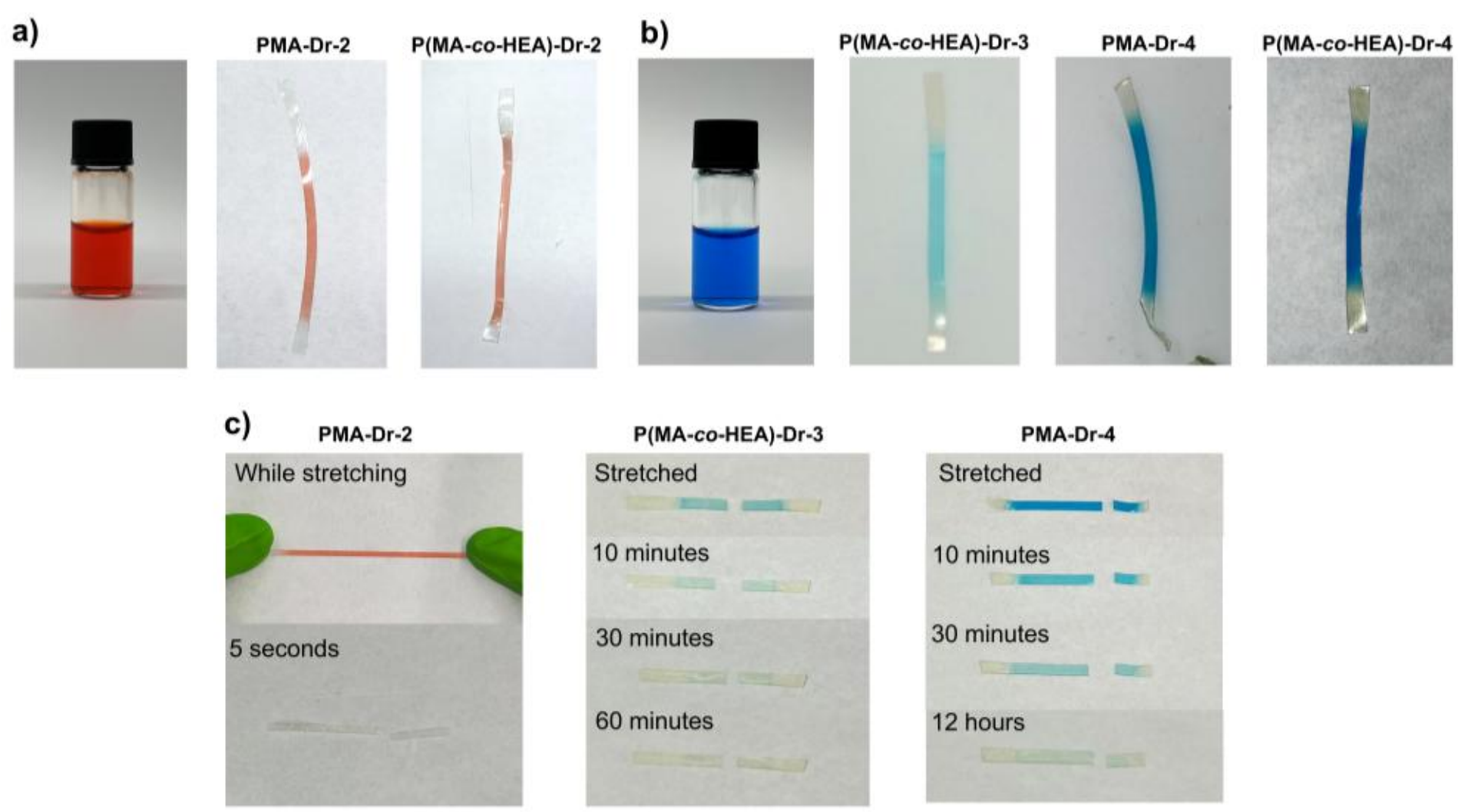

Figure S79: a) Color comparison between **Dr-2**$^+$ in MeCN solution with manually pulled **PMA-Dr-2** and **P(MA-*co*-HEA)-Dr-2**. b) Color comparison between **Dr-3**$^+$**/4**$^+$ in MeCN solution with manually pulled **P(MA-*co*-HEA)-Dr-3**, **PMA-Dr-4**, and **P(MA-*co*-HEA)-Dr-4.** c) Representative photographs illustrating the temporal evolution of the mechanochromic response after unloading for **PMA-Dr-2**, **P(MA-co-HEA)-Dr-3**, and **PMA-Dr-4** under ambient conditions.

In contrast, **P(MA-*co*-HEA)-Dr-1** shows no noticeable evidence of mechanochromism, either to the naked eye or upon irradiation with 254 nm or 366 nm UV light (lamps used to reveal spots on TLC plates). Only the overstressed region exhibited a faint fluorescence near the cut. Similarly, **PMA-Dr-3** showed no mechanochromism. The absence of hydrogen bonding in the matrix prevents stabilization of the negatively charged product.

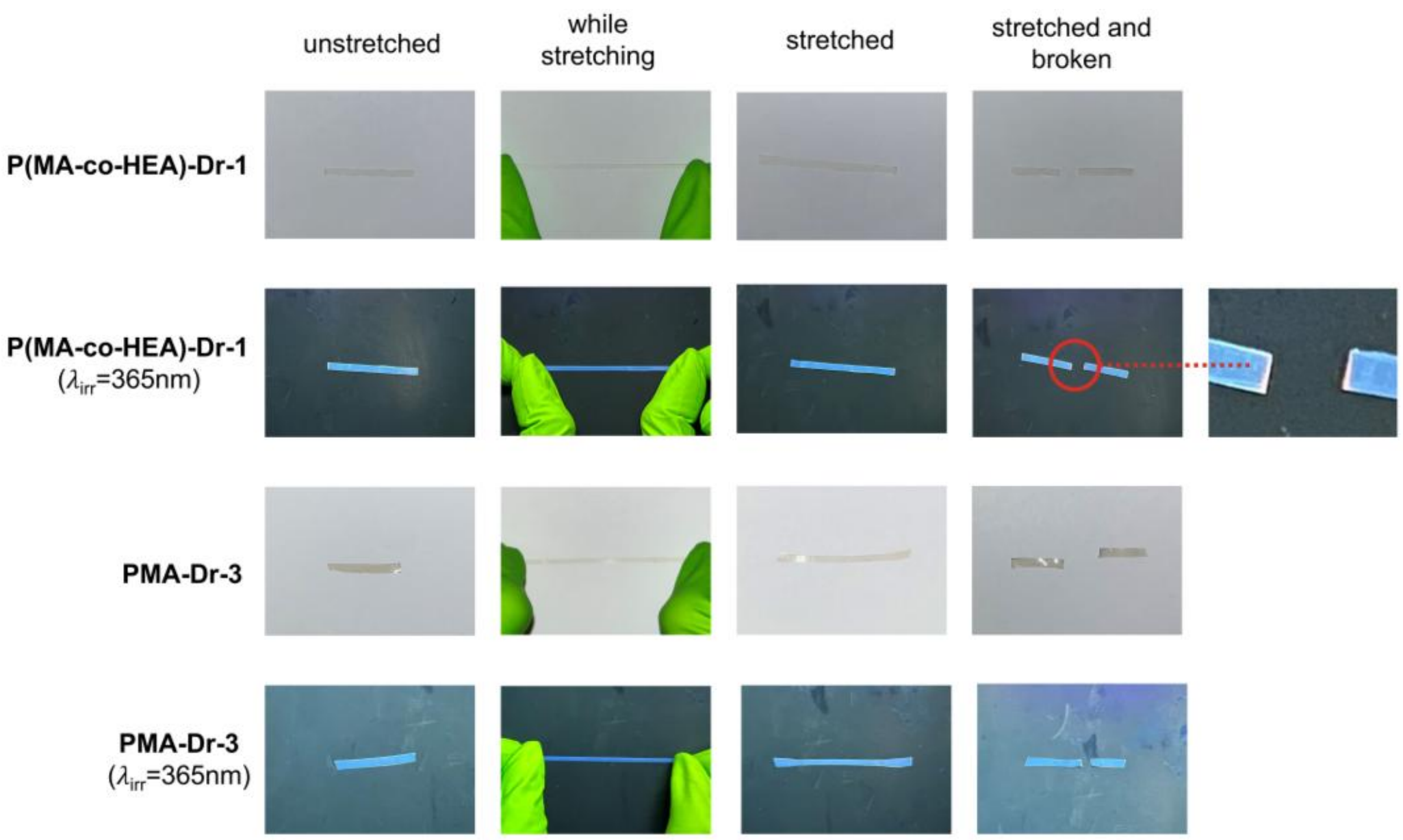

Figure S80: Photos of the stretching process of **P(MA-*co*-HEA)-Dr-1** and **PMA-Dr-3.**

All the control polymer networks **PMA-BVBB-Dr-I/II/IV** and **P(MA-*co*-HEA)-BVBB-Dr-III** did not exhibit mechanochromism.

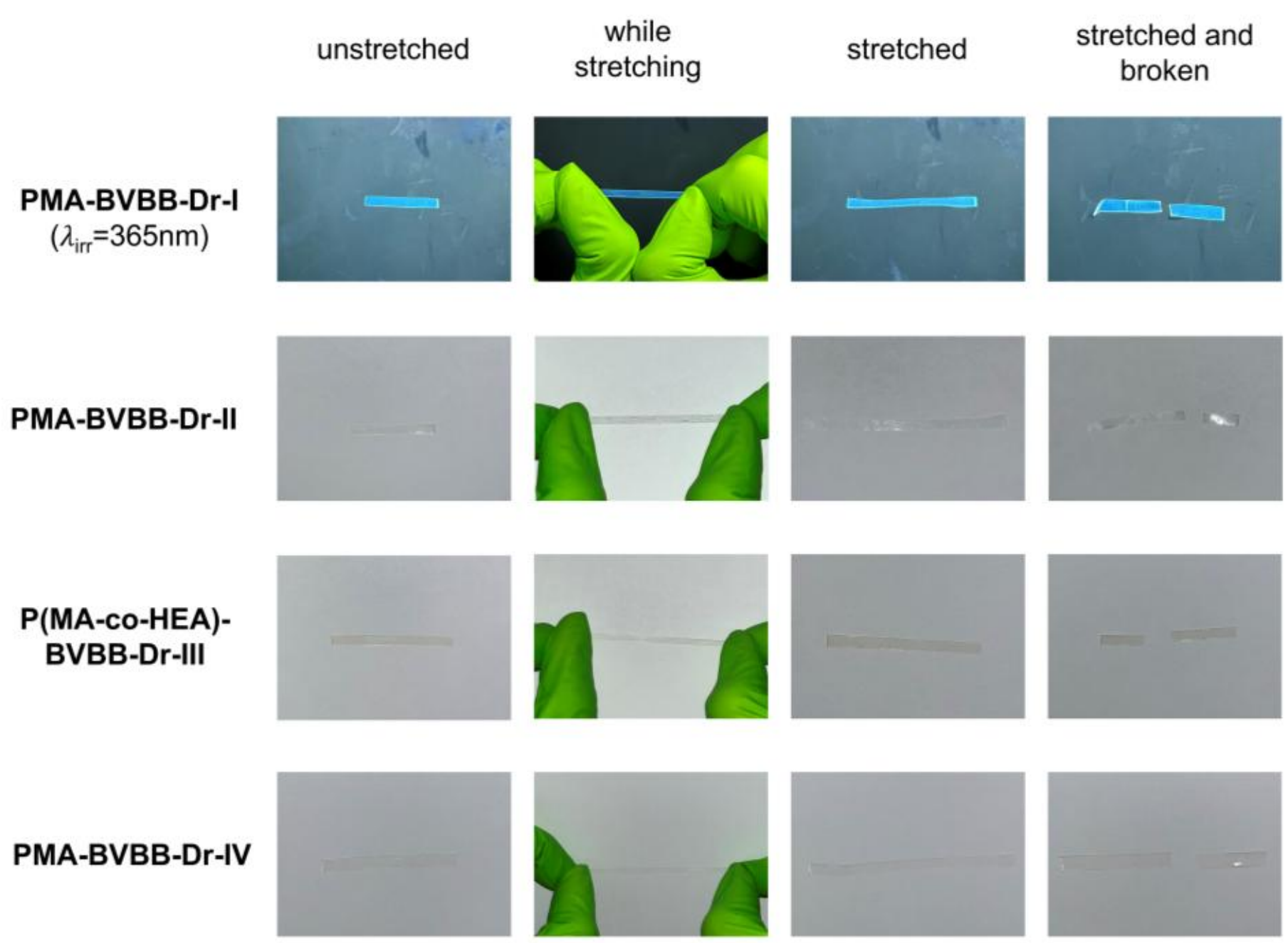

Figure S81: Photos of the stretching process of the control polymer networks.

Mechanical and thermal characterization of PMA and P(MA-*co*-HEA) networks

Tensile test

All polymer networks were subjected to uniaxial tensile deformation at a strain rate of 2.2%·s^{-1} (375 µm·s^{-1}) at 25 °C, and every run was repeated at least in triplicate (Figures S82–93, black, red, and blue traces). The films were cut into rectangular shapes with a width of 5.0 mm and a thickness of 250 µm. In mechanoresponsive polymer networks, the ultimate stress and strain depended on the polymer composition and the nature of the mechanophores, reflecting their intrinsic activation capabilities.

PMA-Dr-2 was tested at 30%·s^{-1} (5000 µm·s^{-1}) due to the transient nature of the released carbocations. This higher strain rate was employed exclusively to experimentally capture the change in optical transmission and to construct a ΔT versus stress–strain curve (cf. Main text, Results and Discussion). In the case of **P(MA-*co*-HEA)-Dr-2**, manual elongation resulted in the activation of the mechanophore. Thus, tensile testing was performed directly at 2.2%·s^{-1} (375 µm·s^{-1}) (Figure S86).

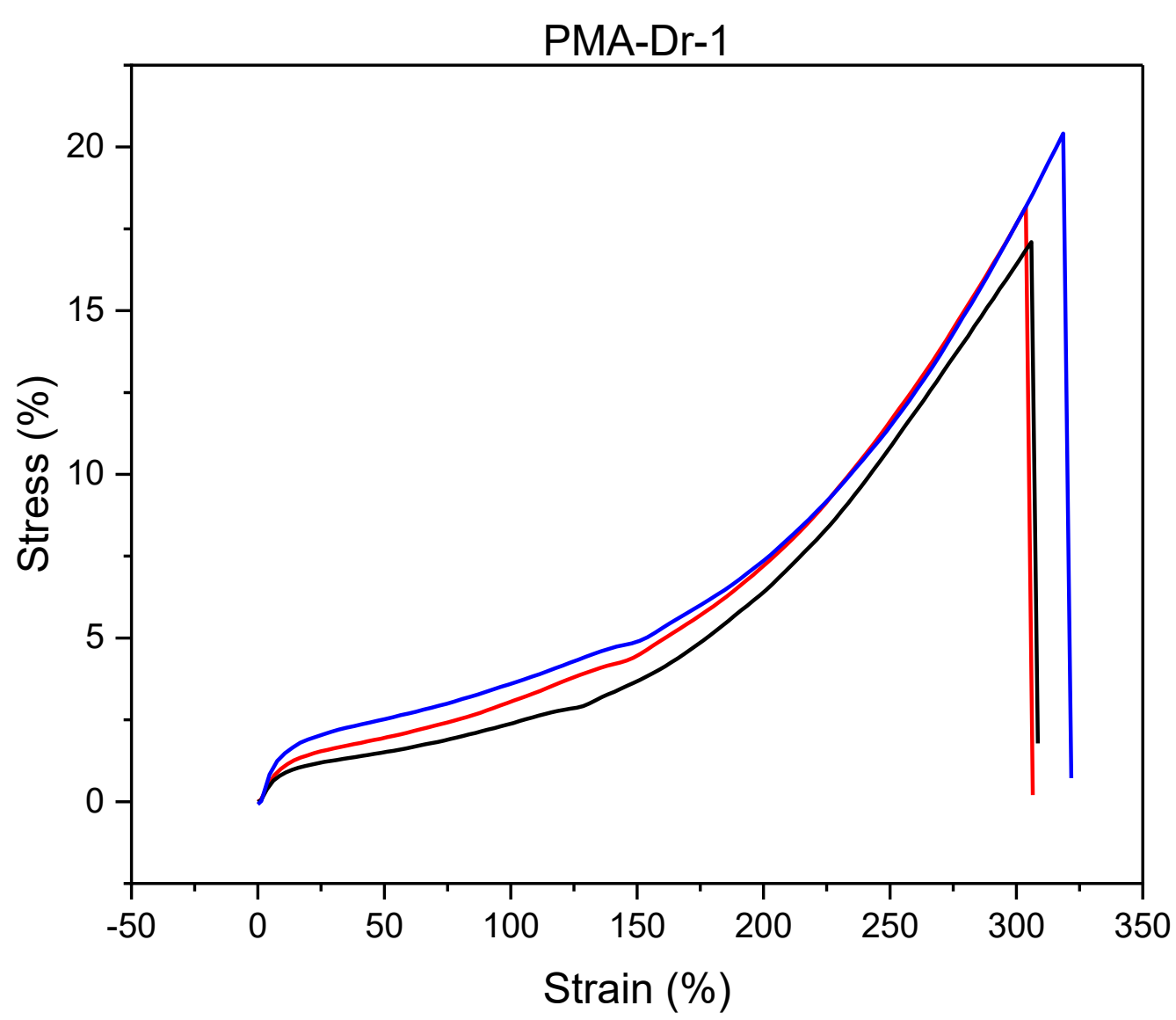

Figure S82: Stress-strain curves of **PMA-Dr-1** obtained from three separate tensile experiments at 2.2%·s^{-1} (375 µm·s^{-1}) strain rate.

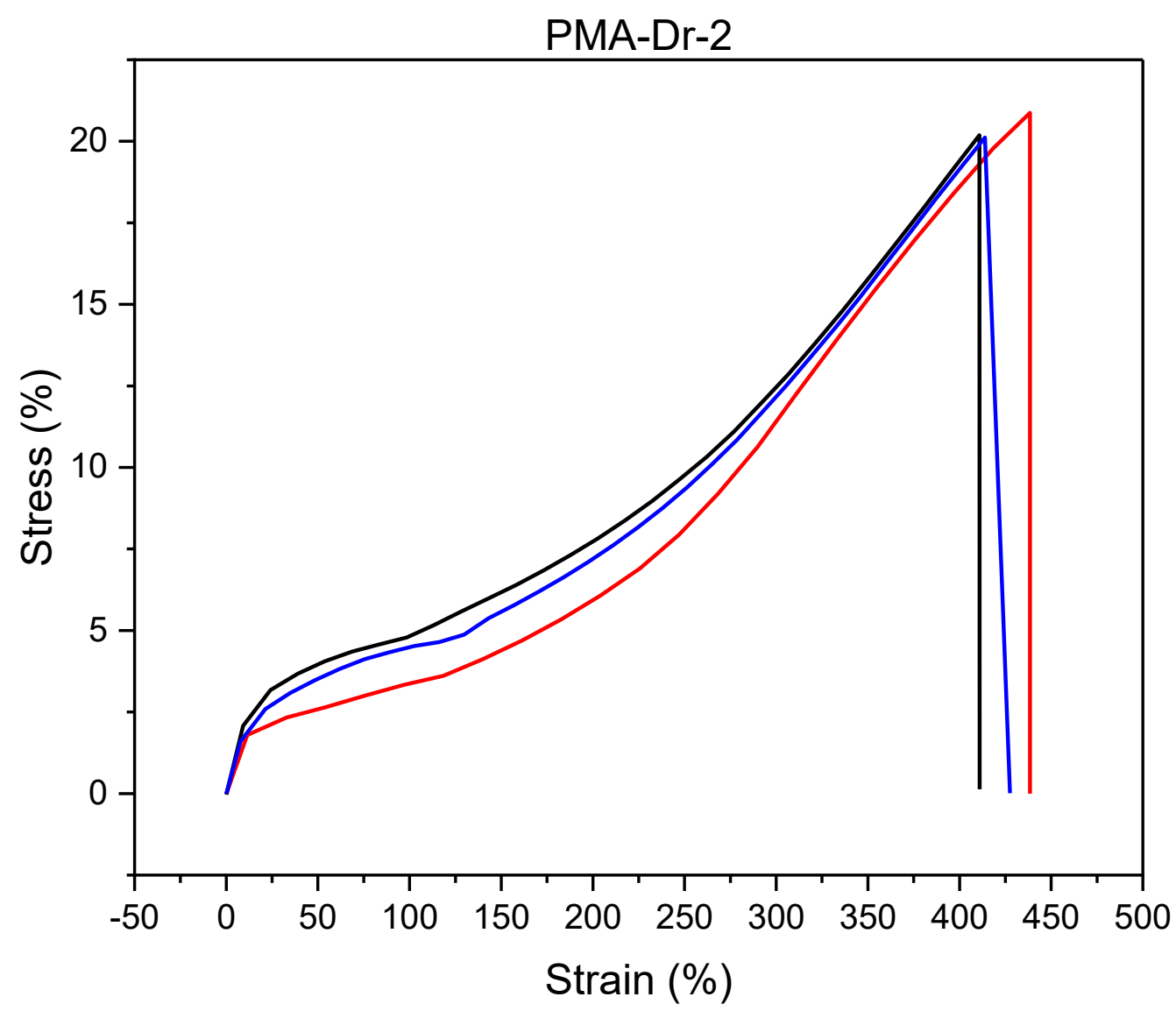

Figure S83: Stress-strain curves at 30%·s^{-1} (5000 μm·s^{-1}) strain rate of **PMA-Dr-2** obtained from three separate tensile experiments at 30%·s^{-1} (5000 μm·s^{-1}) strain rate.

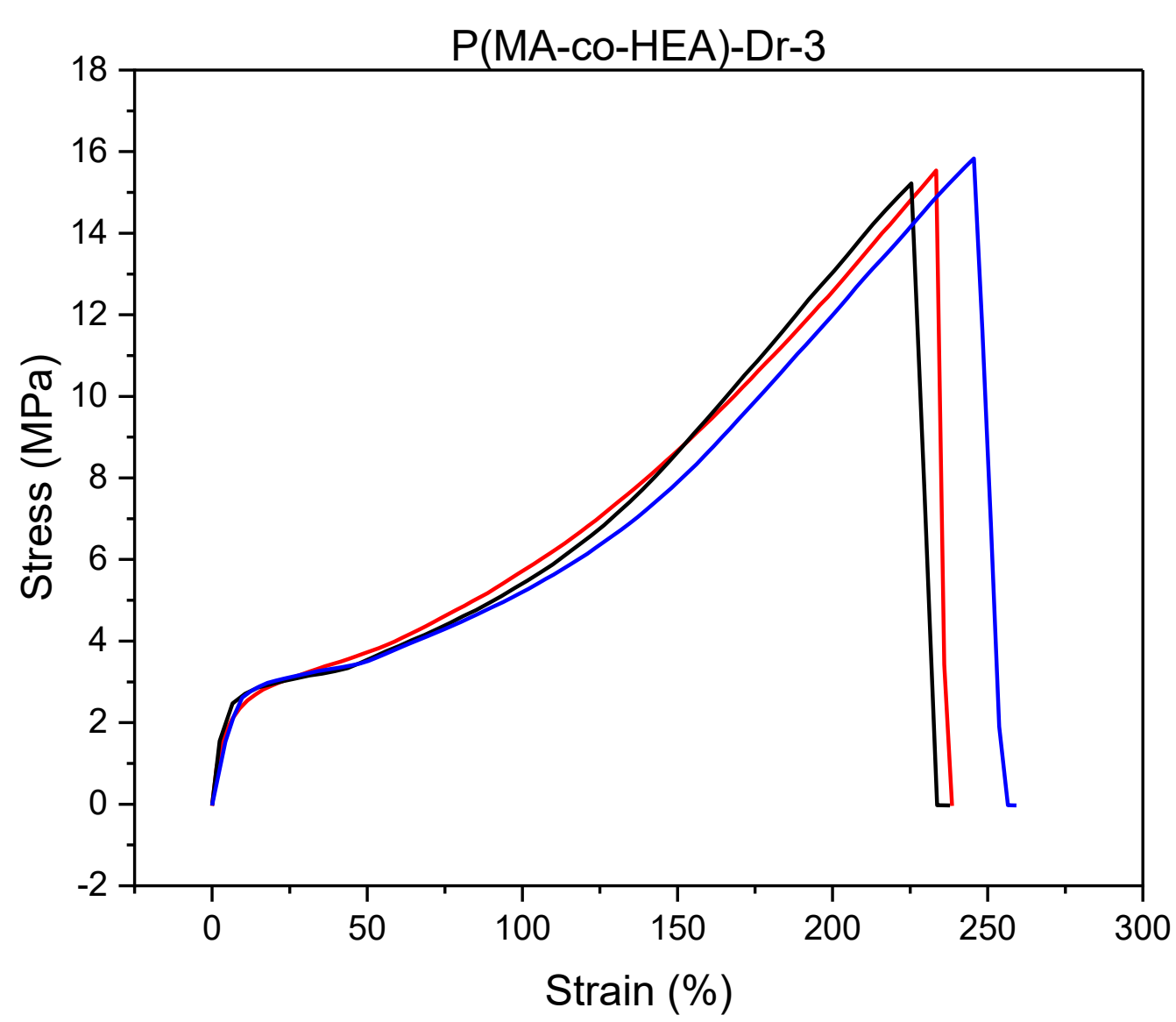

Figure S84: Stress-strain curves 2.2%·s^{-1} (375 μm·s^{-1}) strain rate of **P(MA-*co*-HEA)-Dr-3** obtained from three separate tensile experiments at 2.2%·s^{-1} (375 μm·s^{-1}) strain rate.

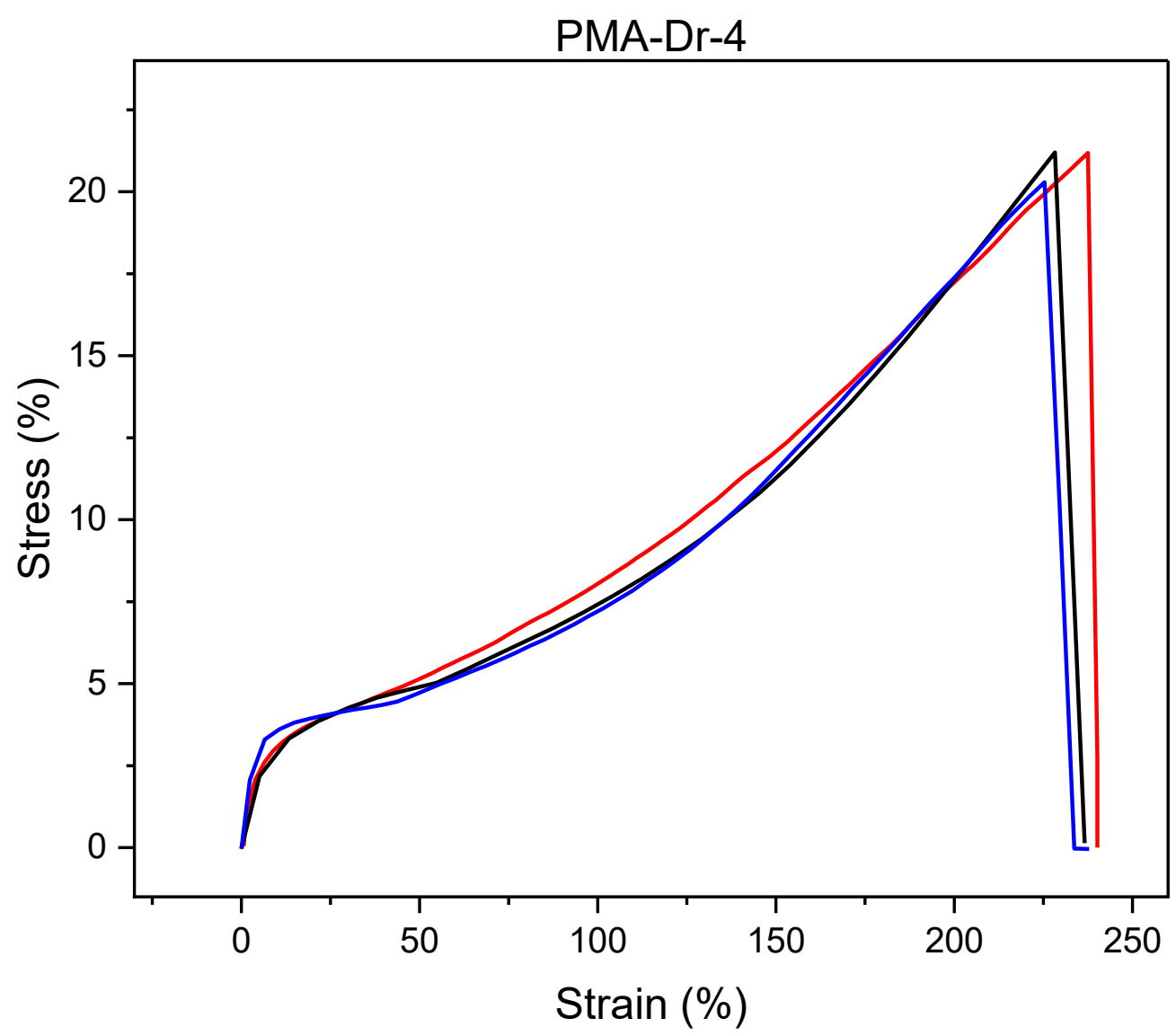

Figure S85: Stress-strain curves of **PMA-Dr-4** obtained from three separate tensile experiments at 2.2%·s^{-1} (375 µm·s^{-1}) strain rate.

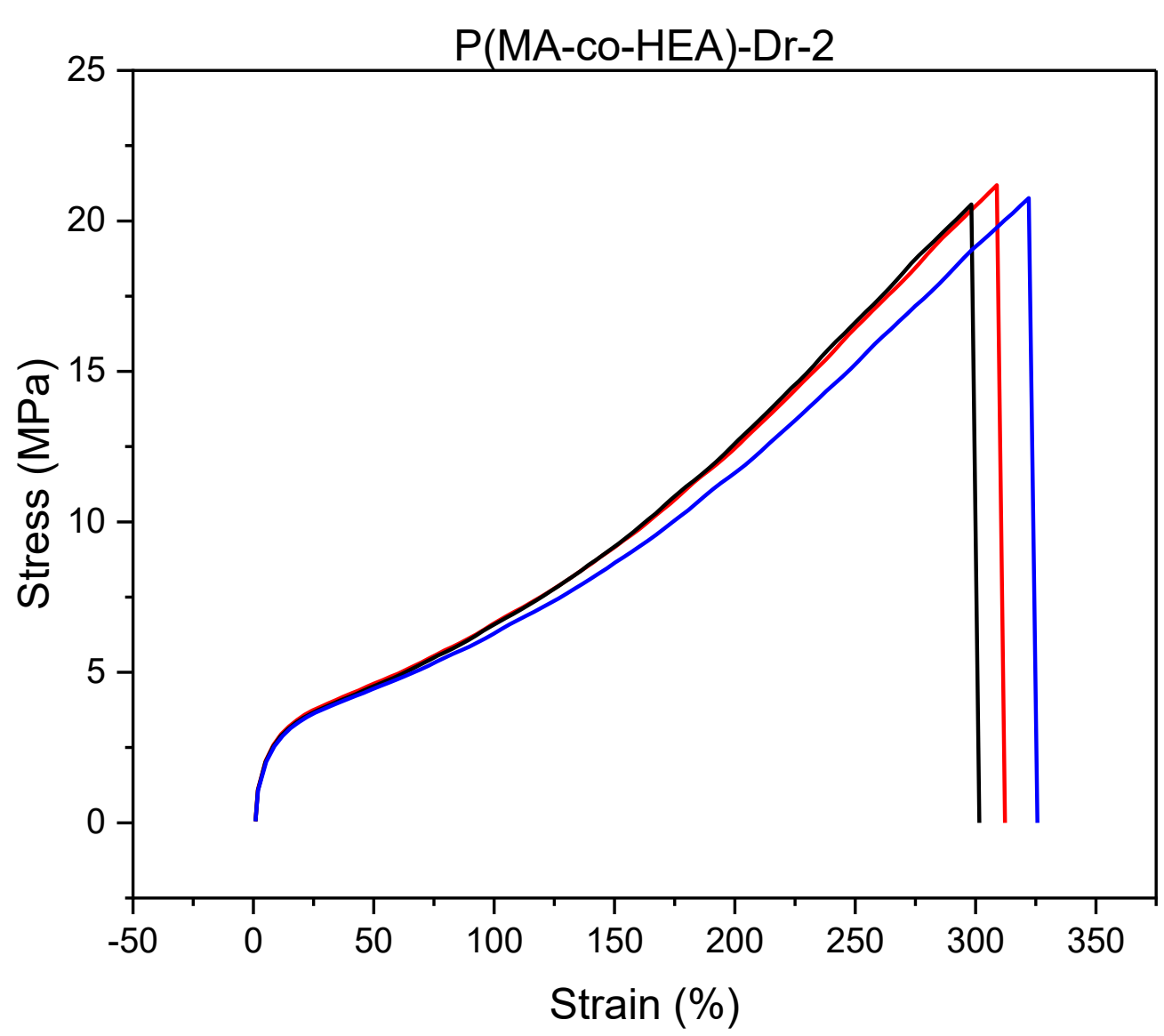

Figure S86: Stress-strain curves of **P(MA-*co*-HEA)-Dr-2** obtained from three separate tensile experiments at 2.2%·s^{-1} (375 µm·s^{-1}) strain rate.

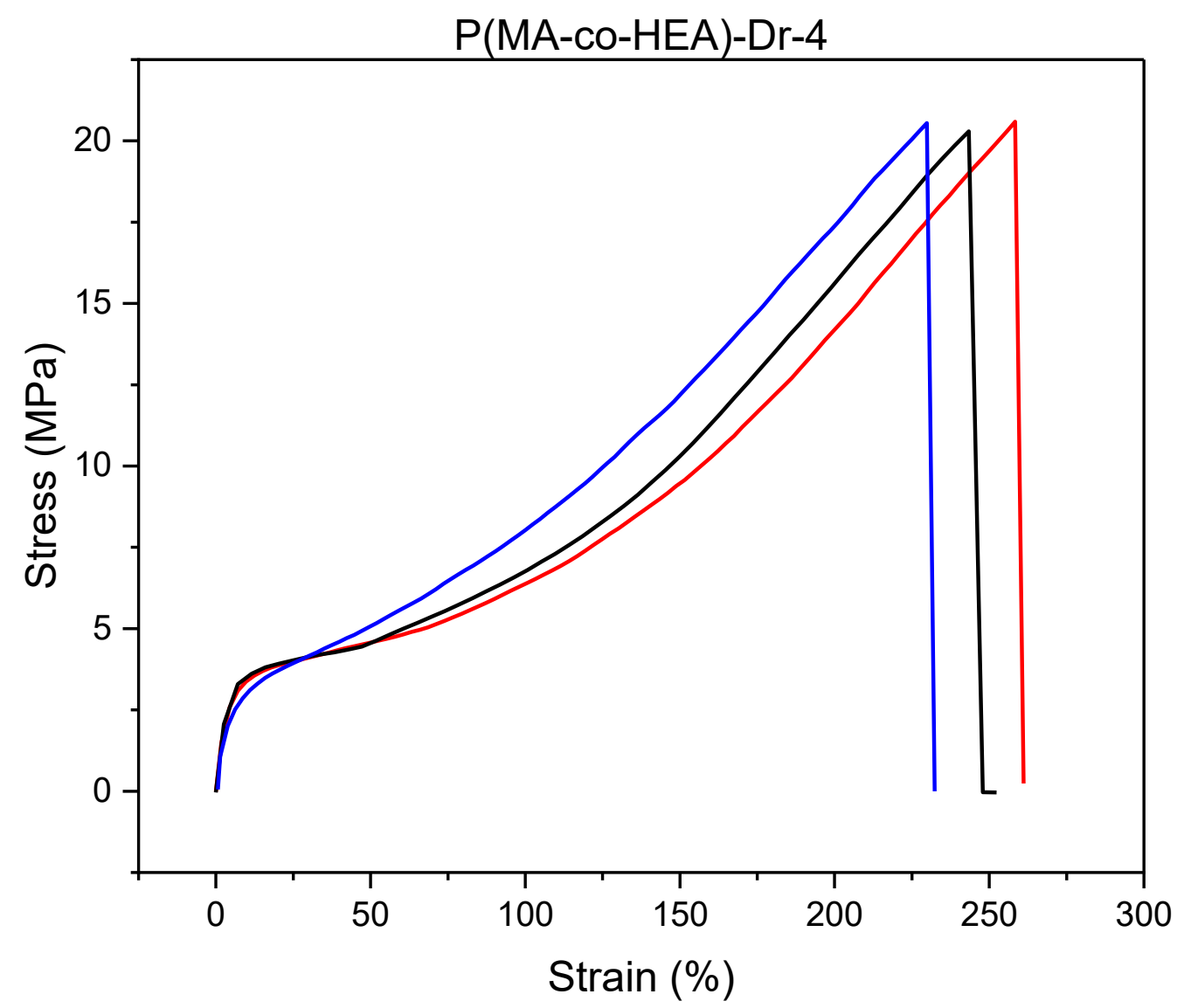

Figure S87: Stress-strain curves of **P(MA-*co*-HEA)-Dr-4** obtained from three separate tensile experiments at 2.2%·s^{-1} (375 µm·s^{-1}) strain rate.

Table S2: Strain and stress at break values (**ε_B** and **σ_{UTS}**, respectively) and Young's modulus (**E_Y**) of the mechanoresponsive polymers.

**Polymer**	Strain at break **ε_B** (%)	Stress at break **σ_{UTS}** (MPa)	Young's modulus **E_Y** (MPa)
**PMA-Dr-1**	312±8	18.8±1.6	19.8±3.6
**PMA-Dr-2**[a]	421±15	20.4±0.4	20.0±3.2
**P(MA-*co*-HEA)-Dr-3**	242 ± 15	15.5 ± 0.3	40.2±12.3
**PMA-Dr-4**	235±6	20.9±0.5	46.2±4.1
**P(MA-*co*-HEA)-Dr-2**	310 ± 12	20.8 ± 0.3	48.3 ± 2.9
**P(MA-*co*-HEA)-Dr-4**	245 ± 14	20.3 ± 0.4	47.5 ± 33.6

[a] Experiment carried out at 30%·s^{-1} (5000 µm·s^{-1})

Non-mechanoresponsive polymer networks. All stress–strain curves displayed profiles typical of amorphous, crosslinked elastomers; however, the stresses at break were lower than

those of the mechanoresponsive counterparts. In particular, **P(MA-*co*-HEA)-Dr-1** and **PMA-Dr-3** did not reach the values observed for their mechanoresponsive analogues.

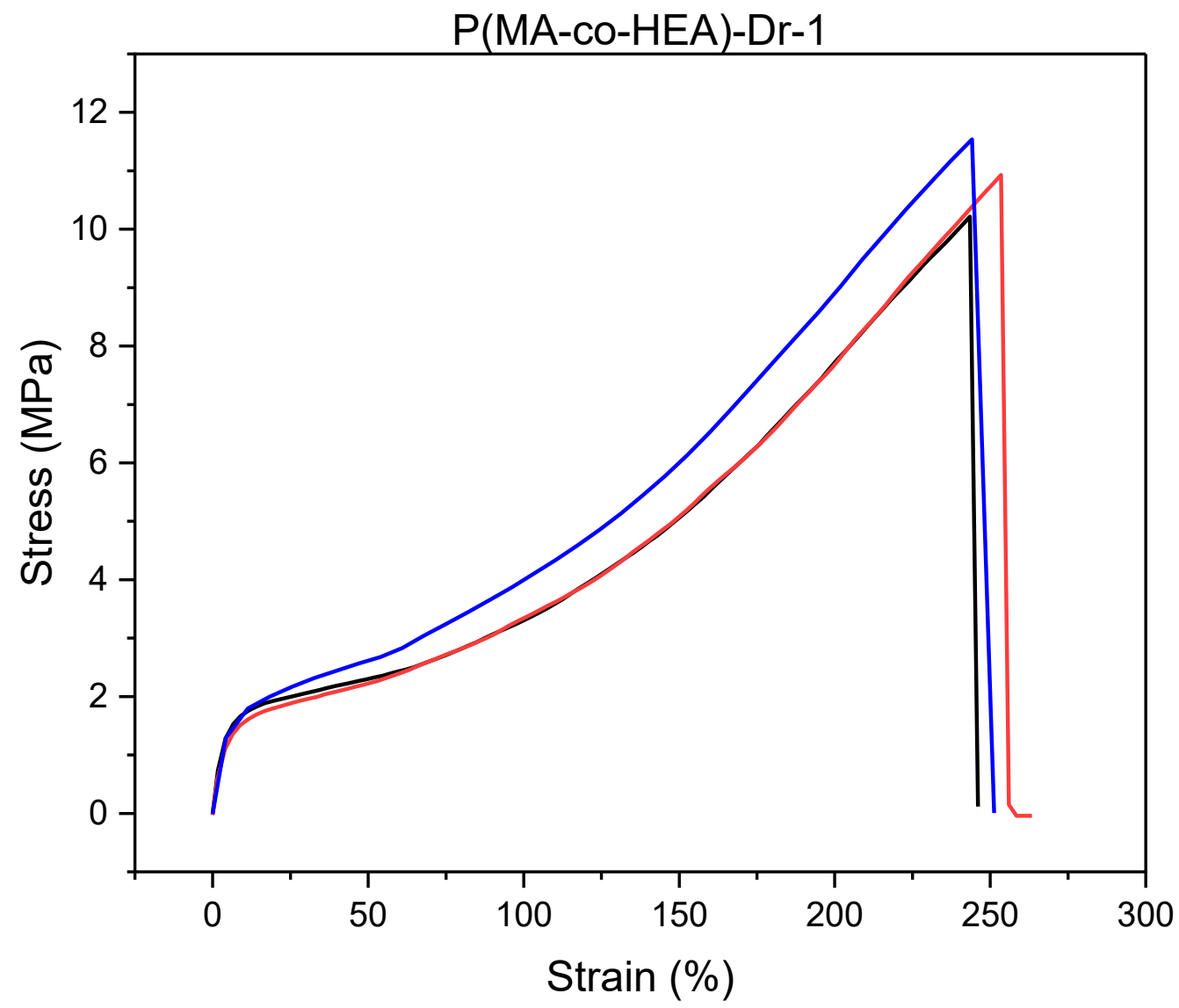

Figure S88: Stress-strain curves of **P(MA-*co*-HEA)-Dr-1** obtained from three separate tensile experiments at 2.2%·s^{-1} (375 µm·s^{-1}) strain rate**.**

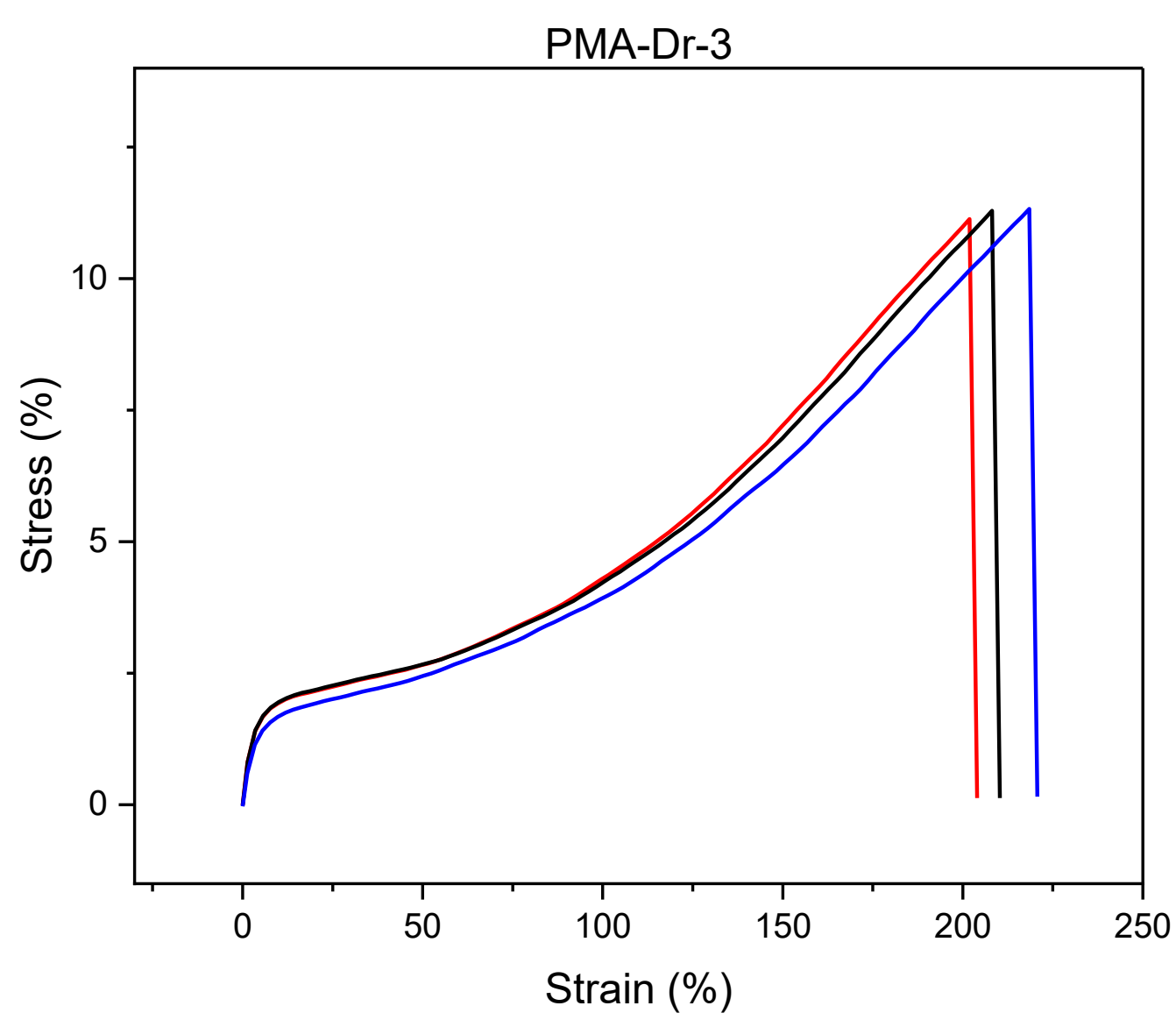

Figure S89: Stress-strain curves of **PMA-Dr-3** obtained from three separate tensile experiments at 2.2%·s^{-1} (375 µm·s^{-1}) strain rate.

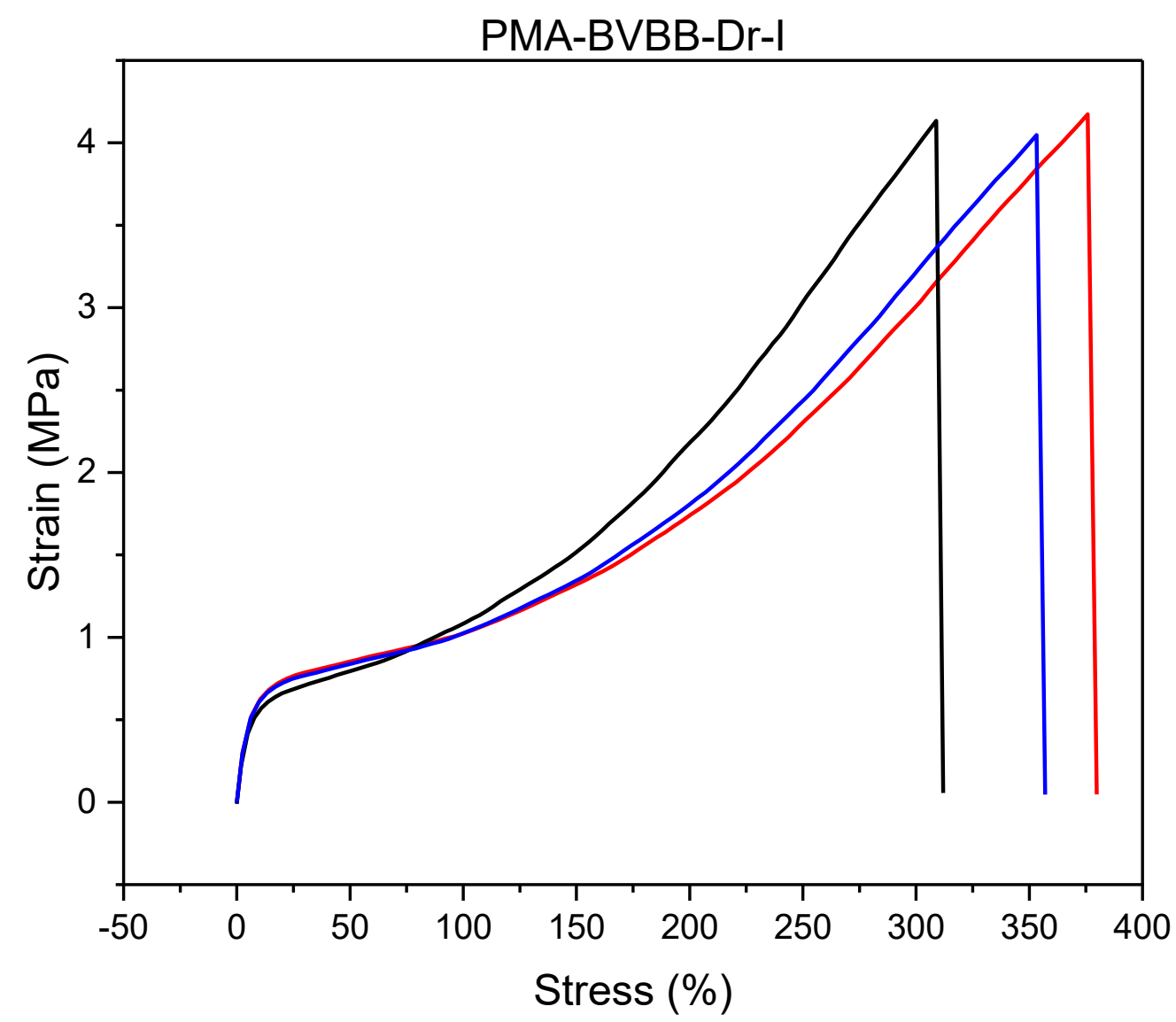

Figure S90: Stress-strain curves of **PMA-BVBB-Dr-I** obtained from three separate tensile experiments at 2.2%·s^{-1} (375 μm·s^{-1}) strain rate.

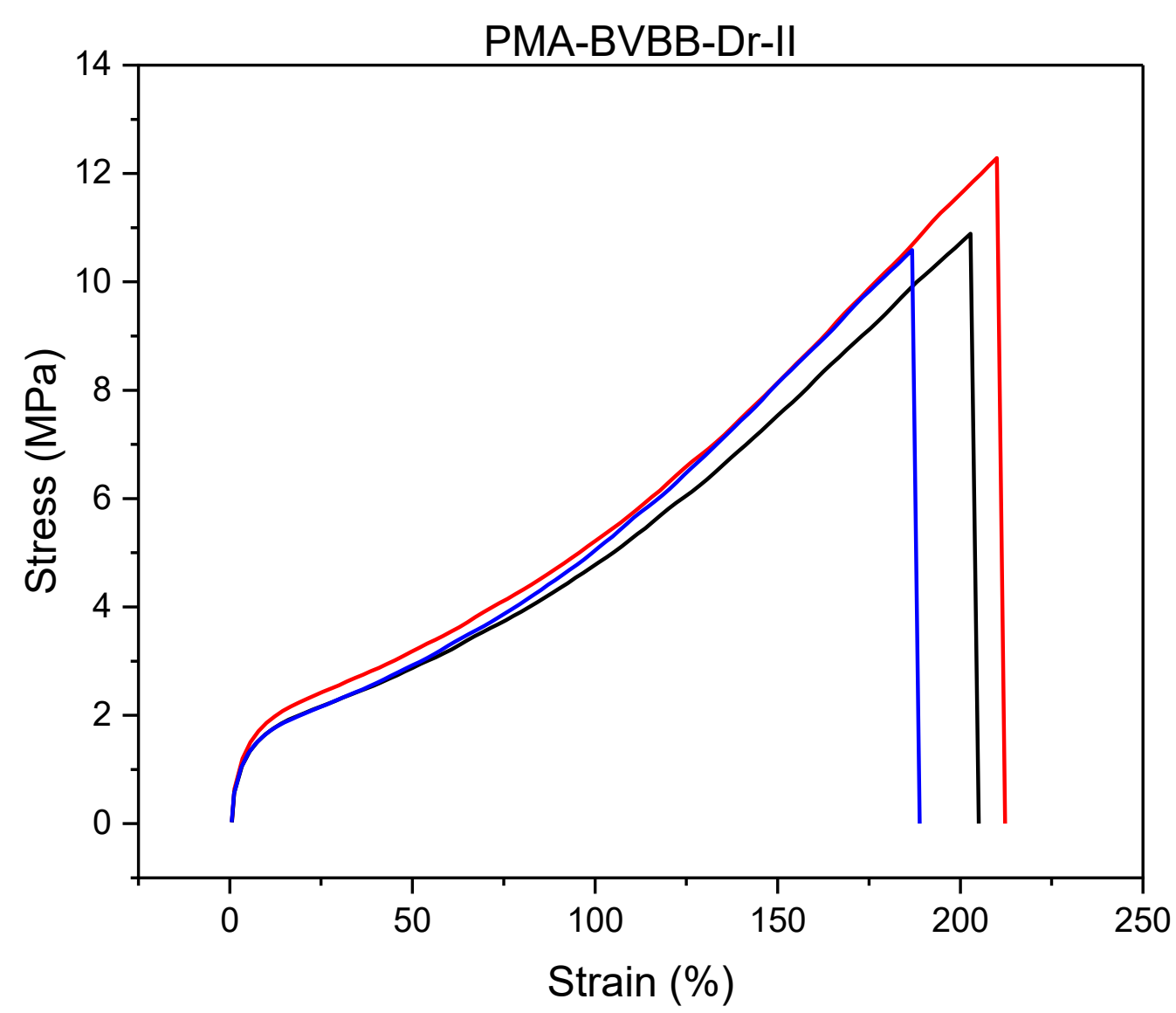

Figure S91: Stress-strain curves of **PMA-BVBB-Dr-II** obtained from three separate tensile experiments at 2.2%·s^{-1} (375 μm·s^{-1}) strain rate.

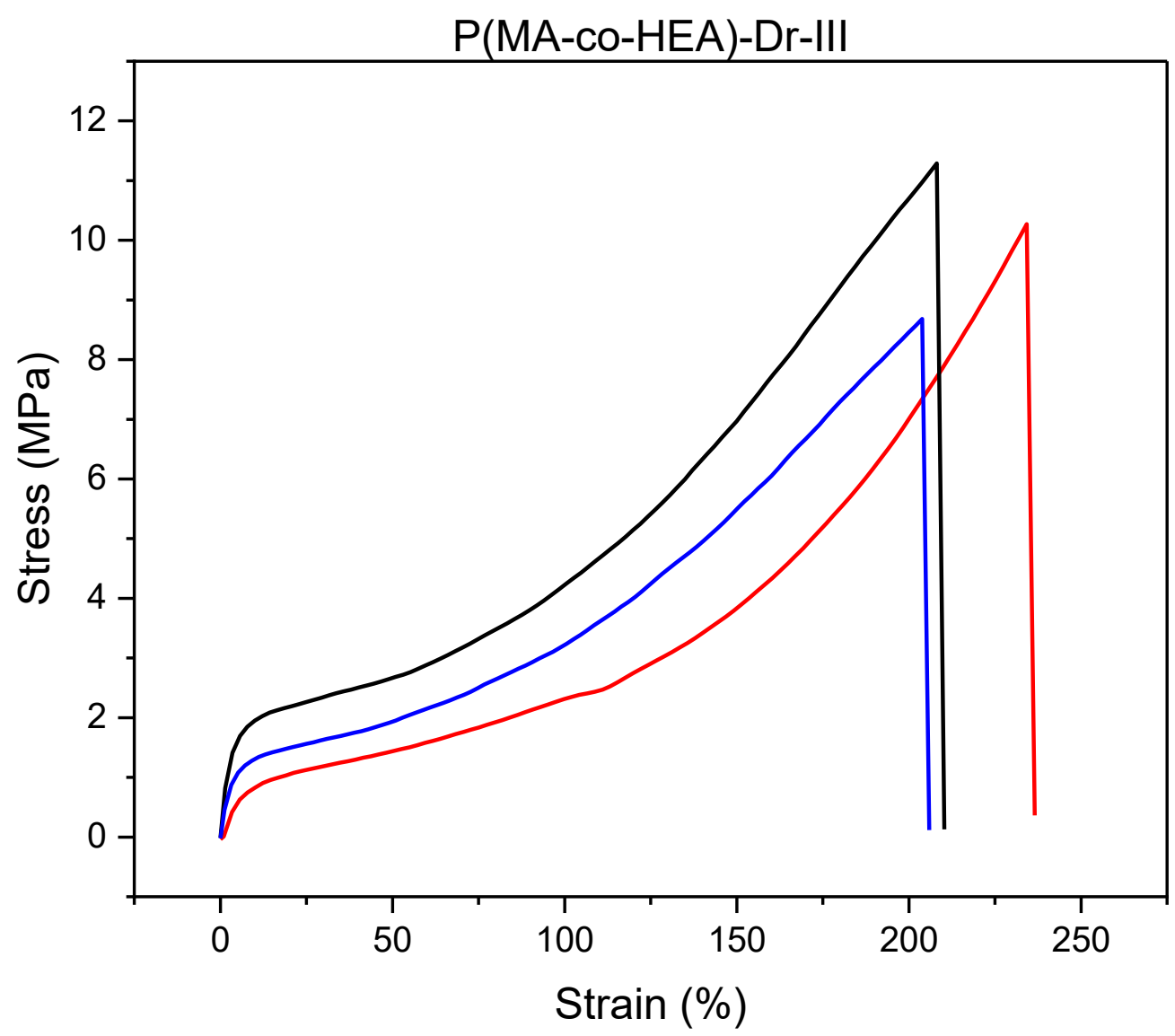

Figure S92: Stress-strain curves of **P(MA-*co*-HEA)-Dr-III** obtained from three separate tensile experiments at 2.2%·s⁻¹ (375 μm·s⁻¹) strain rate.

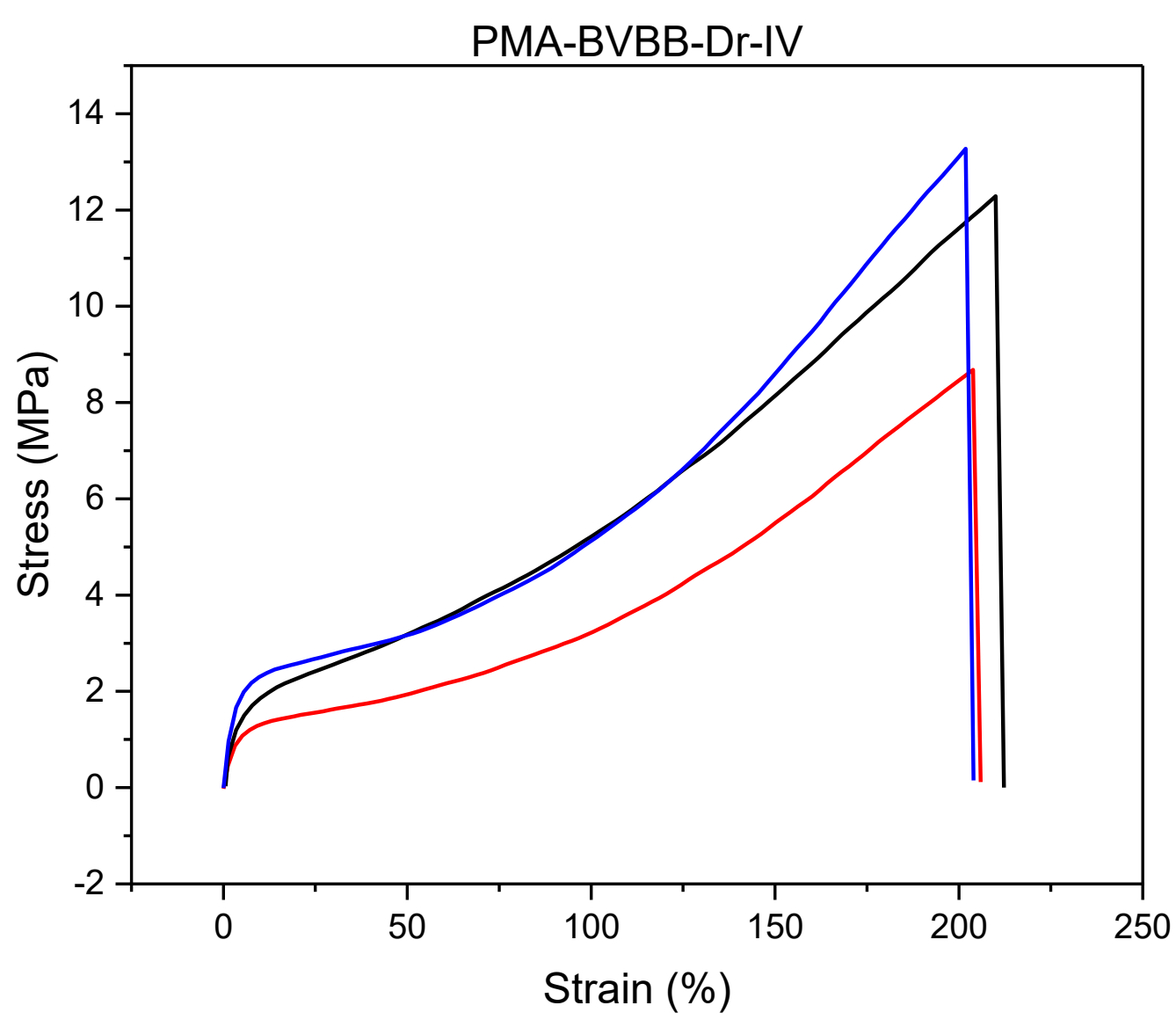

Figure S93: Stress-strain curves of **PMA-BVBB-Dr-IV** obtained from three separate tensile experiments at 2.2%·s⁻¹ (375 μm·s⁻¹) strain rate.

Table S3: Strain and stress at break values (ε_B and σ_{UTS}, respectively) and Young's modulus (E_Y) of the non-mechanoresponsive polymers

**Polymer**	Strain at break ε_B (%)	Stress at break σ_{UTS} (MPa)	Young's modulus E_Y (MPa)
**PMA-BVBB-Dr-I**	326 ± 21	4.0 ± 0.1	35.3 ± 6.5
**PMA-BVBB-Dr-II**	199.2 ± 9.7	11.3± 1.0	25.7±1.0
**P(MA-*co*-HEA)-BVBB-Dr-III**	215.4 ± 16.7	10.1 ± 1.3	19.2± 7.0
**PMA-BVBB-Dr-IV**	205.9 ± 4.2	11.4 ± 2.5	23.3 ± 5.7
**P(MA-*co*-HEA)-Dr-1**	247 ± 4	10.7 ± 0.8	32 ± 3
**PMA-Dr-3**	212±8	11.2±0.1	28.7±2.3

Thermogravimetric analysis (TGA) and Differential Scanning Calorimetry (DSC)

Thermal characterization of the PMA-based polymer networks was performed on homogeneous films with a controlled thickness of 250 µm. TGA was performed under nitrogen to assess the thermal stability of the samples. All PMA-based networks displayed a stable weight profile up to approximately 350 °C, followed by a sharp weight loss associated with thermal degradation. The TGA curves reveal that thermal degradation onset occurs at 350–370 °C for all samples, with **PMA-Dr-1** showing a slightly earlier onset than the others. The temperature at which the materials lost 5% of their weight ($T_{d5\%}$) was above 350 ºC for all the samples. Residual weights at 600 °C were similar across the series, indicating comparable char yields. The full TGA profiles are shown in Figure S94.

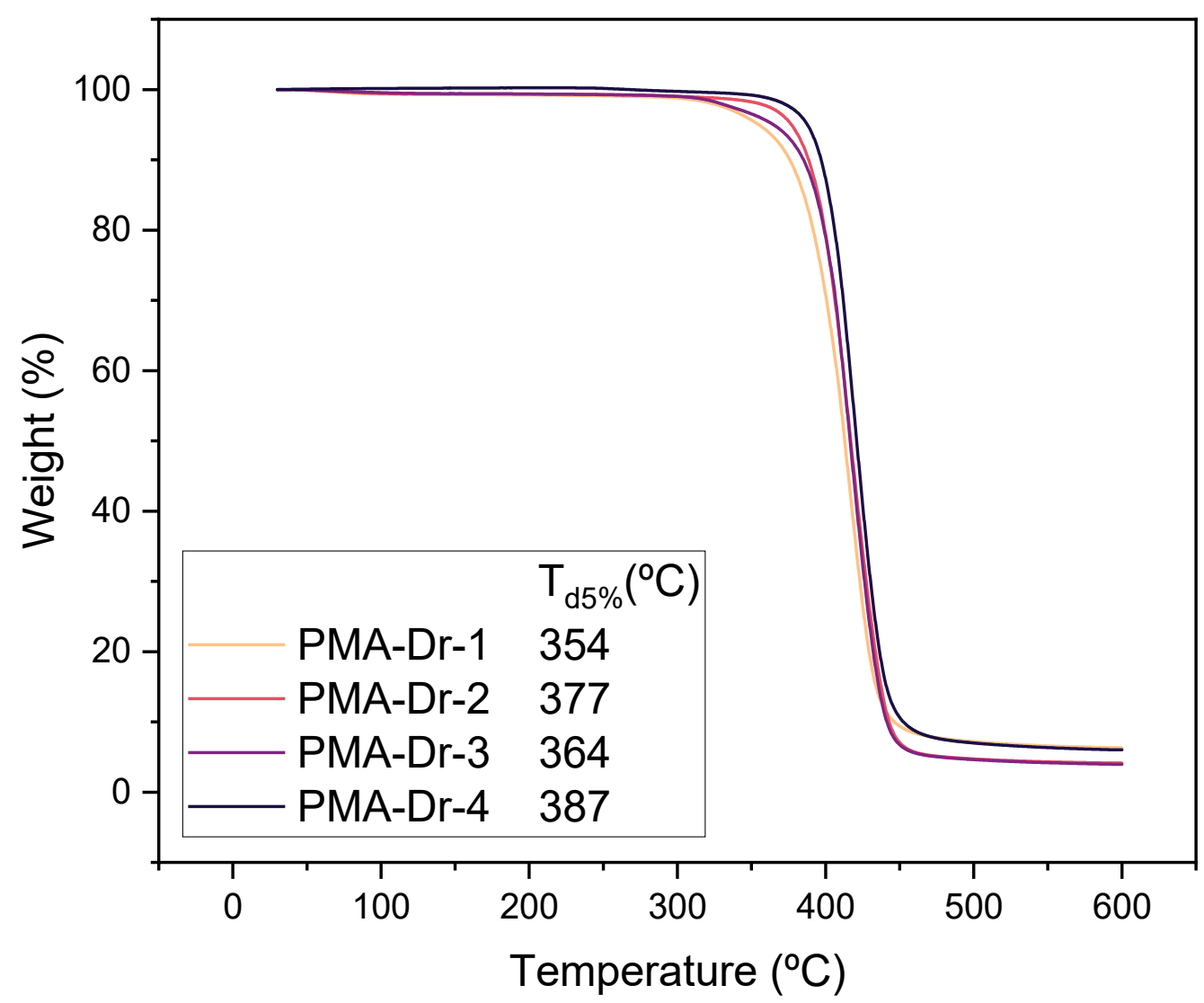

Figure S94: TGA profiles of the PMA polymer networks.

TGA was also performed on PMA-*co*-HEA-based networks to evaluate their thermal stability. The films, also prepared with a controlled thickness of 250 µm, showed once again good thermal stability up to approximately 350–370 °C. A sharp weight loss associated with thermal decomposition followed (360–380 °C), and residual weights at 600 °C ranged from 8% to 4%. These results confirm that the incorporation of HEA does not drastically alter the degradation profile, though subtle effects related to the nature of the diarylmethane mechanophore are evident. Full TGA curves are shown in Figure S95.

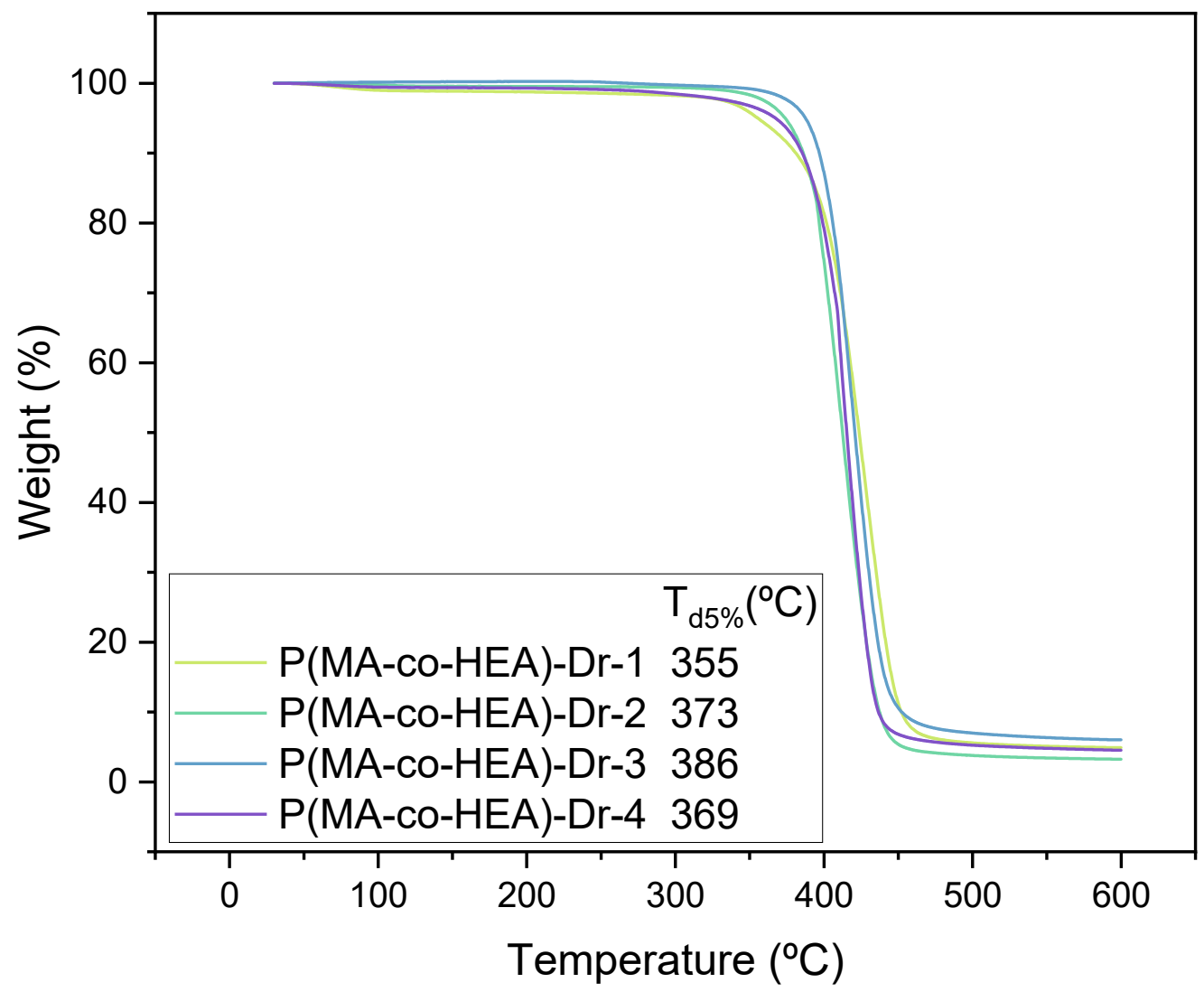

Figure S95: TGA profiles of the P(MA-*co*-HEA) polymer networks.

Finally, TGA experiments on the control polymer films **PMA-BVBB-Dr-I/II/IV** and **P(MA-*co*-HEA)-Dr-III** prepared in the same ways as their mechanoresponsive counterpart, showed the same thermal stability all over the series, with thermal decomposition over 350–380°C and residual weights at 600 °C ranging from 8% to 4%. Full TGA curves are shown in Figure S96.

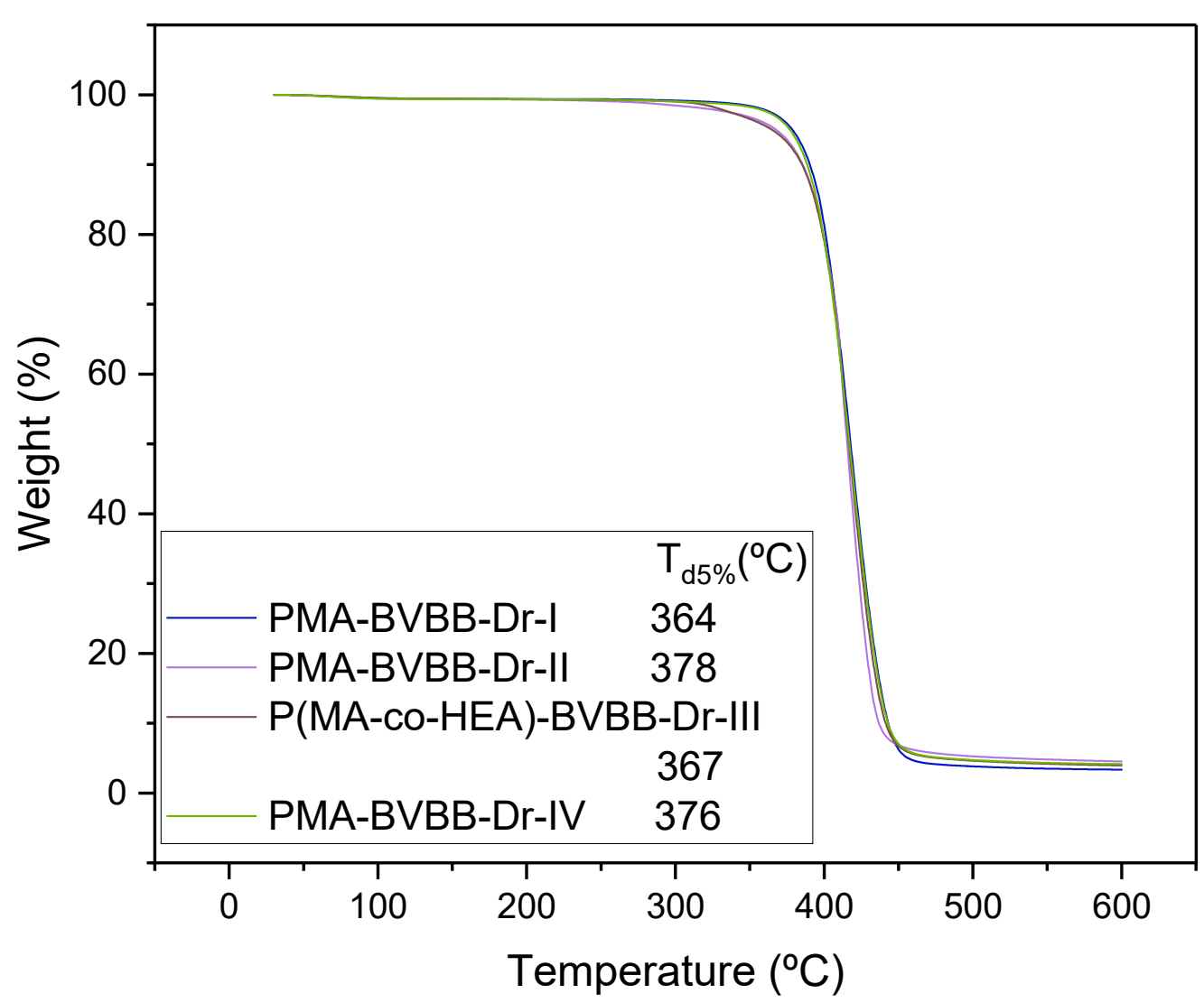

Figure S96: TGA profiles of the control polymer networks.

DSC analysis was carried out with two heating cycles and two cooling cycles, with 5 minutes of isotherm between each ramp. The heating and cooling cycles were performed in the temperature range from −80 to 200 °C at a constant rate of 10 °C min^{-1}. All the T_g values for the PMA and P(MA-*co*-HEA) polymer networks were found to be near room temperature.

Table S4: Glass transition temperature (T_g) of the PMA polymer series

Polymer	T_g (ºC)
**PMA-Dr-1**	21.3
**PMA-Dr-2**	21.0
**PMA-Dr-3**	21.4
**PMA-Dr-4**	21.8

Table S5: Glass transition temperature (T_g) of the P(MA-*co*-HEA) polymer series

Polymer	T_g (ºC)
**P(MA-*co*-HEA)-Dr-1**	22.8
**P(MA-*co*-HEA)-Dr-2**	21.3
**P(MA-*co*-HEA)-Dr-3**	22.6
**P(MA-*co*-HEA)-Dr-4**	22.8

Table S6: Glass transition temperature (T_g) of the PMA and P(MA-*co*-HEA) control polymer series

Polymer	T_g (ºC)
**PMA-BVBB-Dr-I**	14.0
**PMA-BVBB-Dr-II**	12.2
**P(MA-*co*-HEA) - BVBB-Dr-III**	18.8
**PMA-BVBB-Dr-IV**	14.8

PMA series

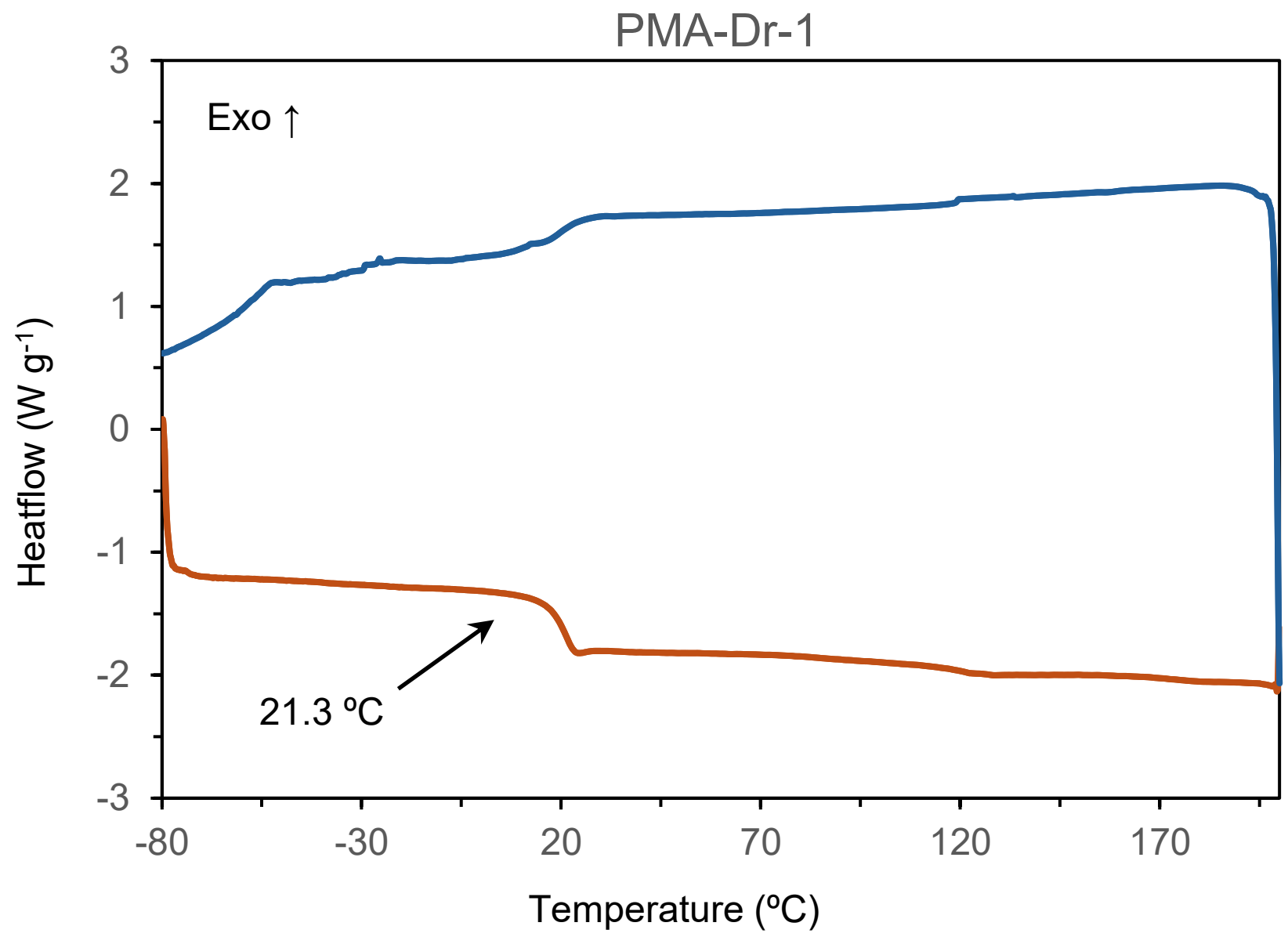

Figure S97: DSC profile (second cycle) of **PMA-Dr-1**. Heating (red trace) and cooling (blue trace) cycles were carried out at a rate of 10 °C min⁻¹.

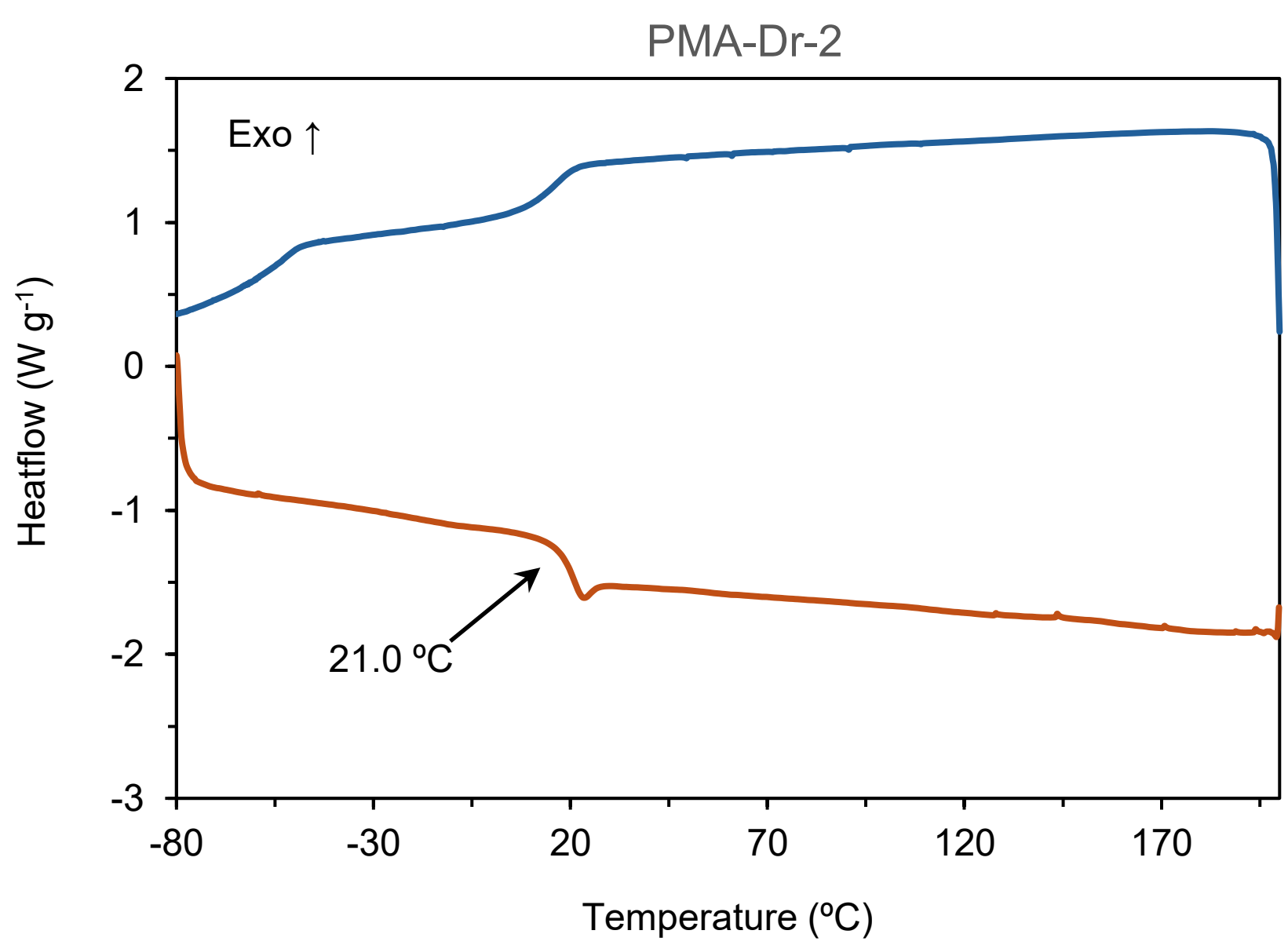

Figure S98: DSC profile (second cycle) of **PMA-Dr-2**. Heating (red trace) and cooling (blue trace) cycles were carried out at a rate of 10 °C min⁻¹.

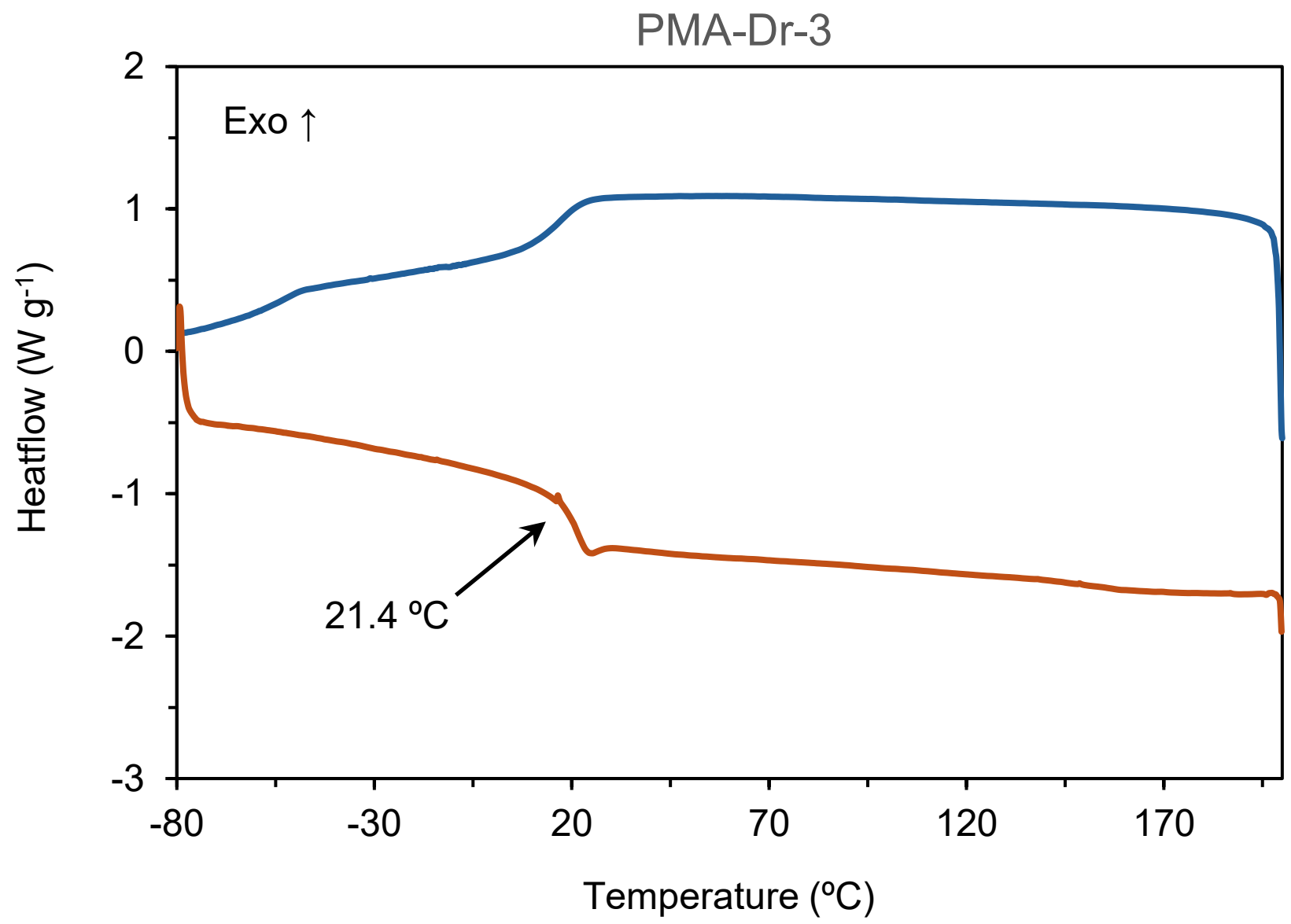

Figure S99: DSC profile (second cycle) of **PMA-Dr-3**. Heating (red trace) and cooling (blue trace) cycles were carried out at a rate of 10 °C min^{-1}.

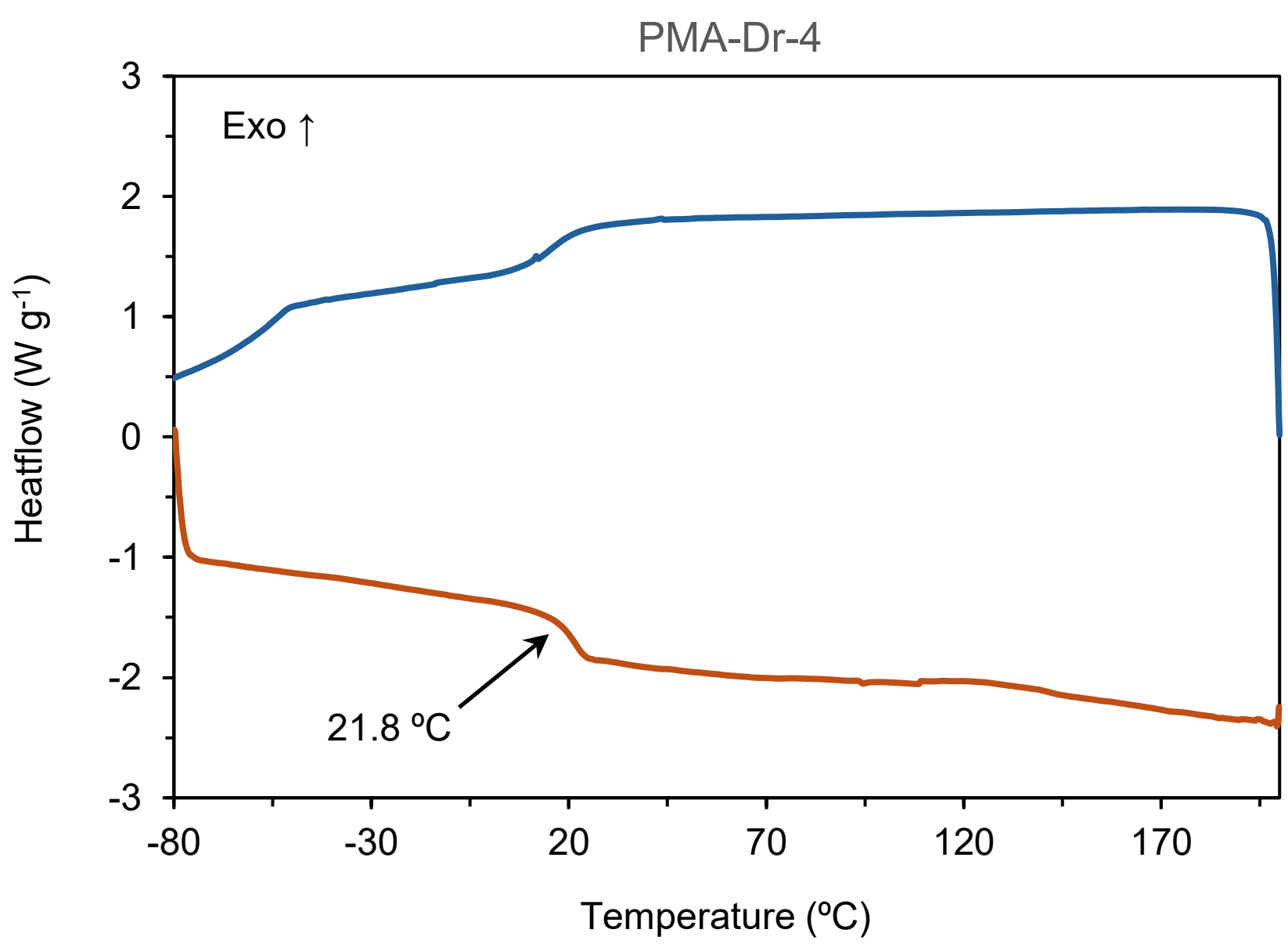

Figure S100: DSC profile (second cycle) of **PMA-Dr-4**. Heating (red trace) and cooling (blue trace) cycles were carried out at a rate of 10 °C min^{-1}.

P(MA-*co*-HEA) series

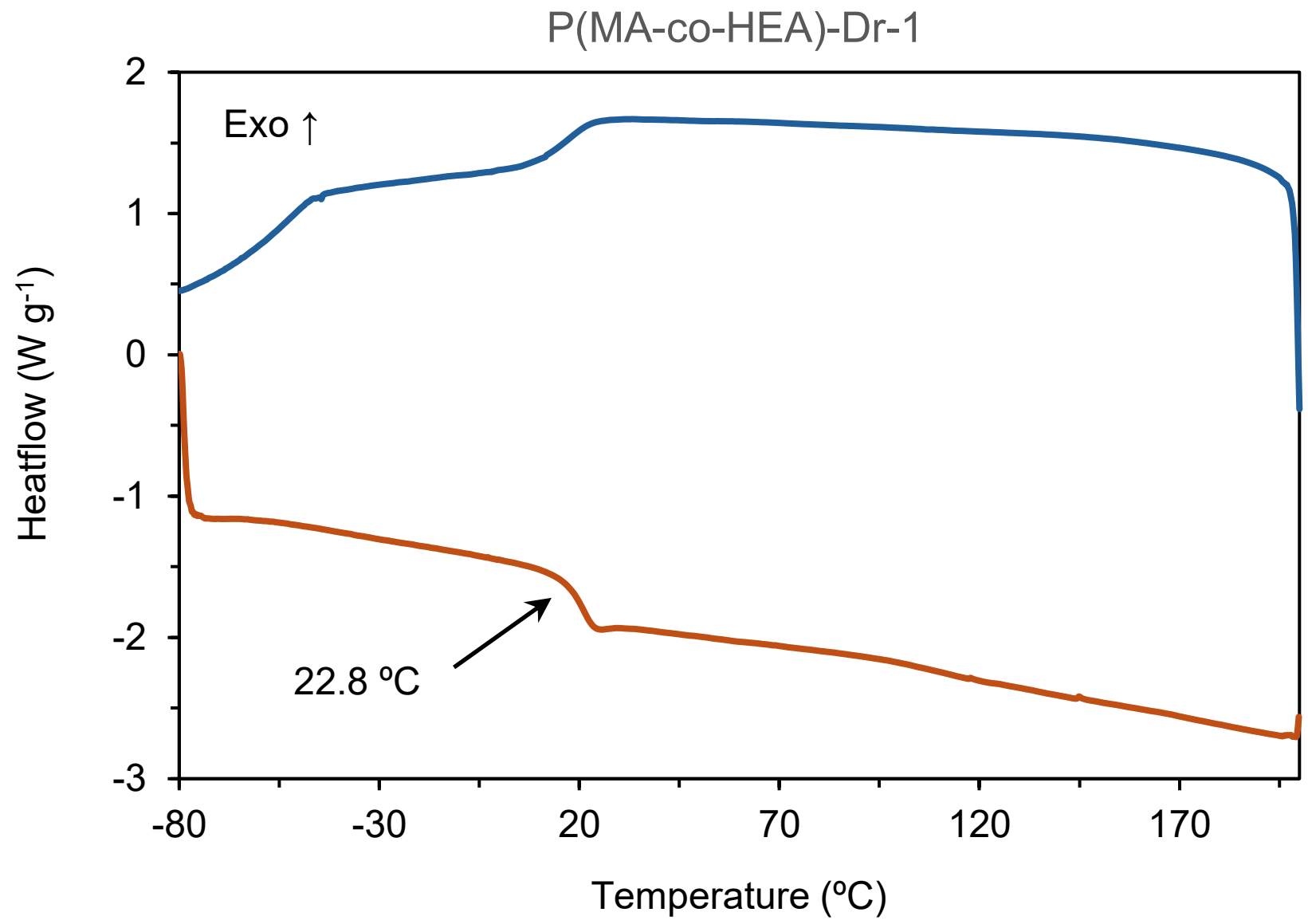

Figure S101: DSC profile (second cycle) of **P(MA-*co*-HEA)-Dr-1**. Heating (red trace) and cooling (blue trace) cycles were carried out at a rate of 10 °C min^{-1}.

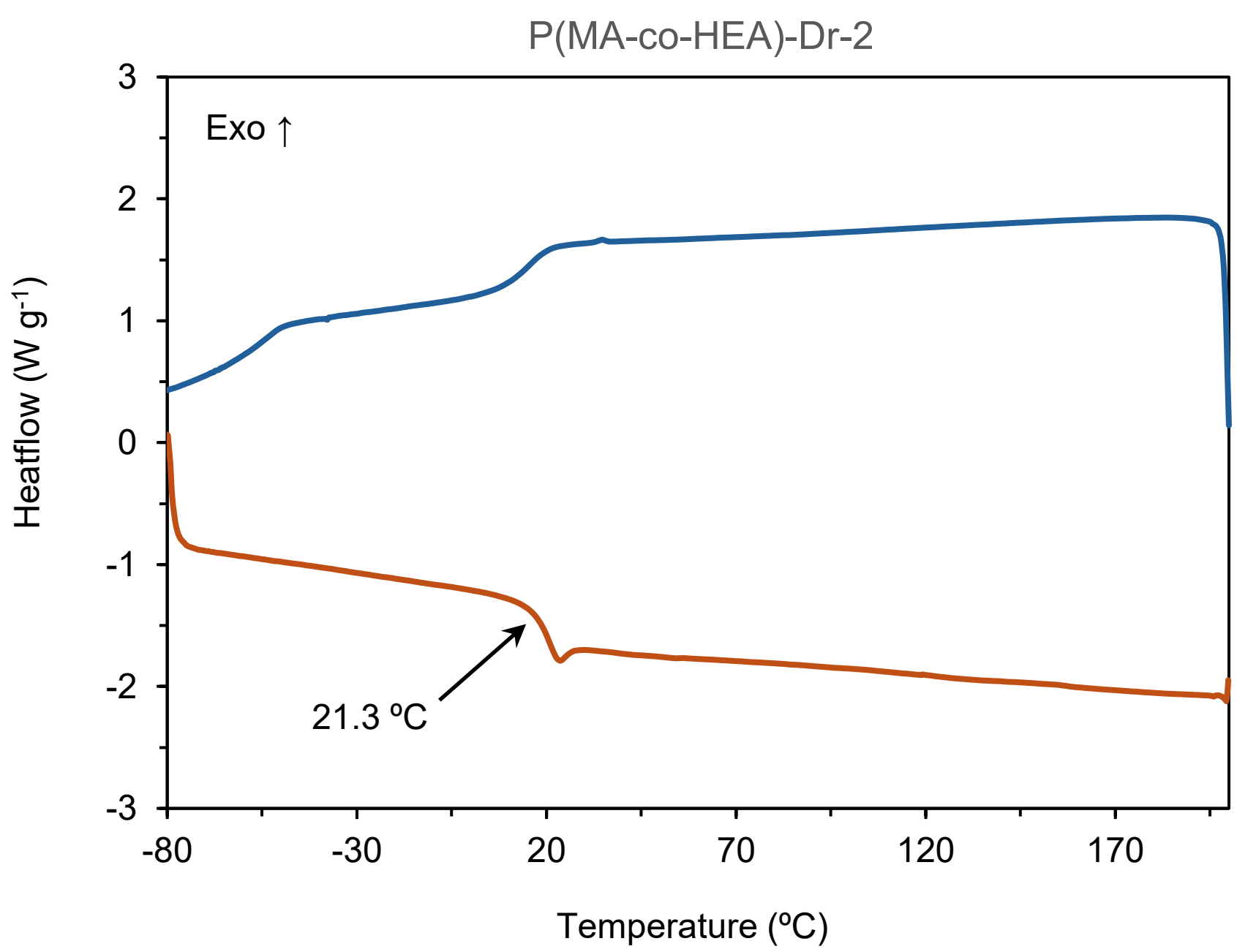

Figure S102: DSC profile (second cycle) of **P(MA-*co*-HEA)-Dr-2**. Heating (red trace) and cooling (blue trace) cycles were carried out at a rate of 10 °C min^{-1}.

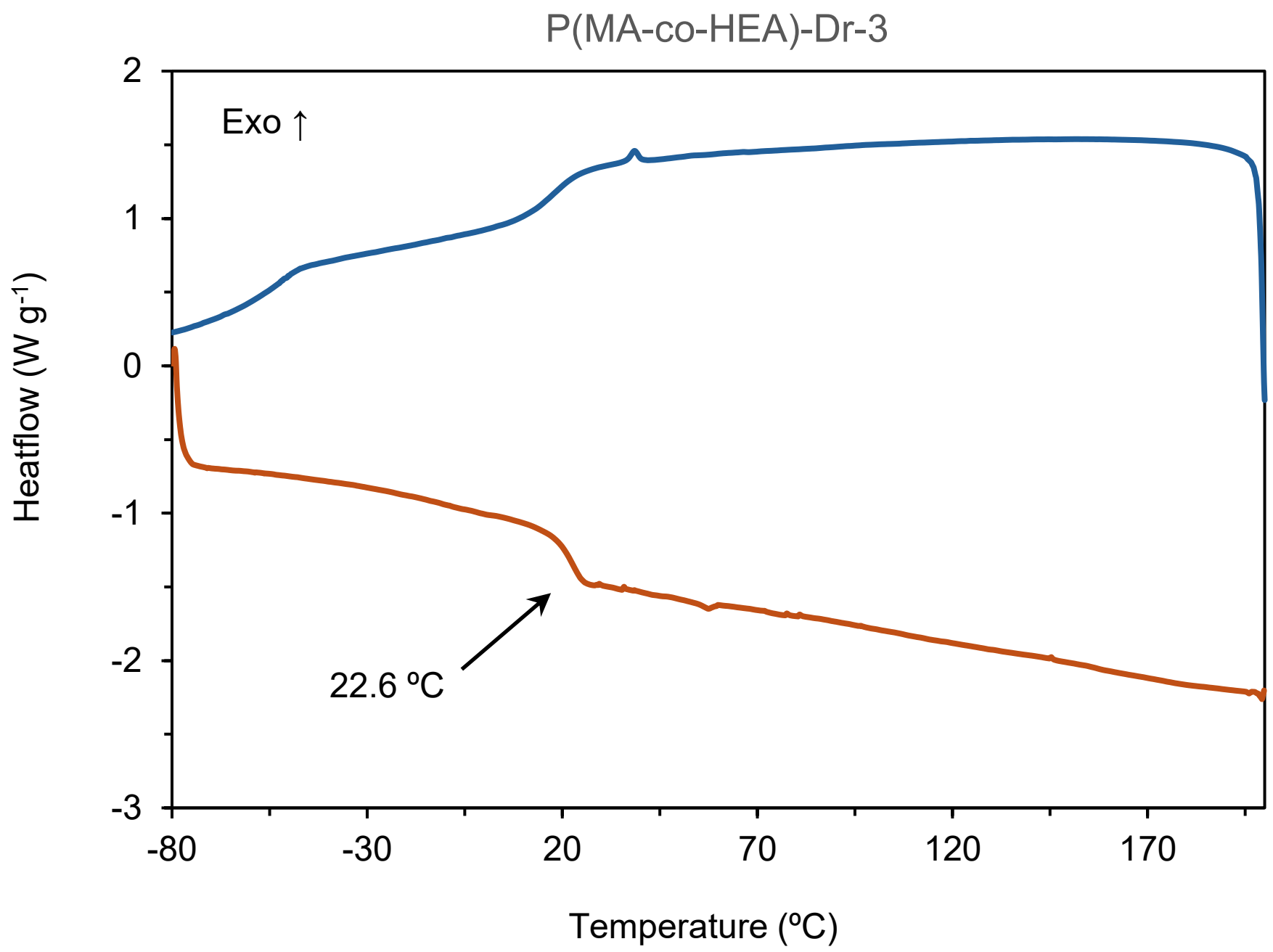

Figure S103: DSC profile (second cycle) of **P(MA-*co*-HEA)-Dr-3**. Heating (red trace) and cooling (blue trace) cycles were carried out at a rate of 10 °C min^{-1}.

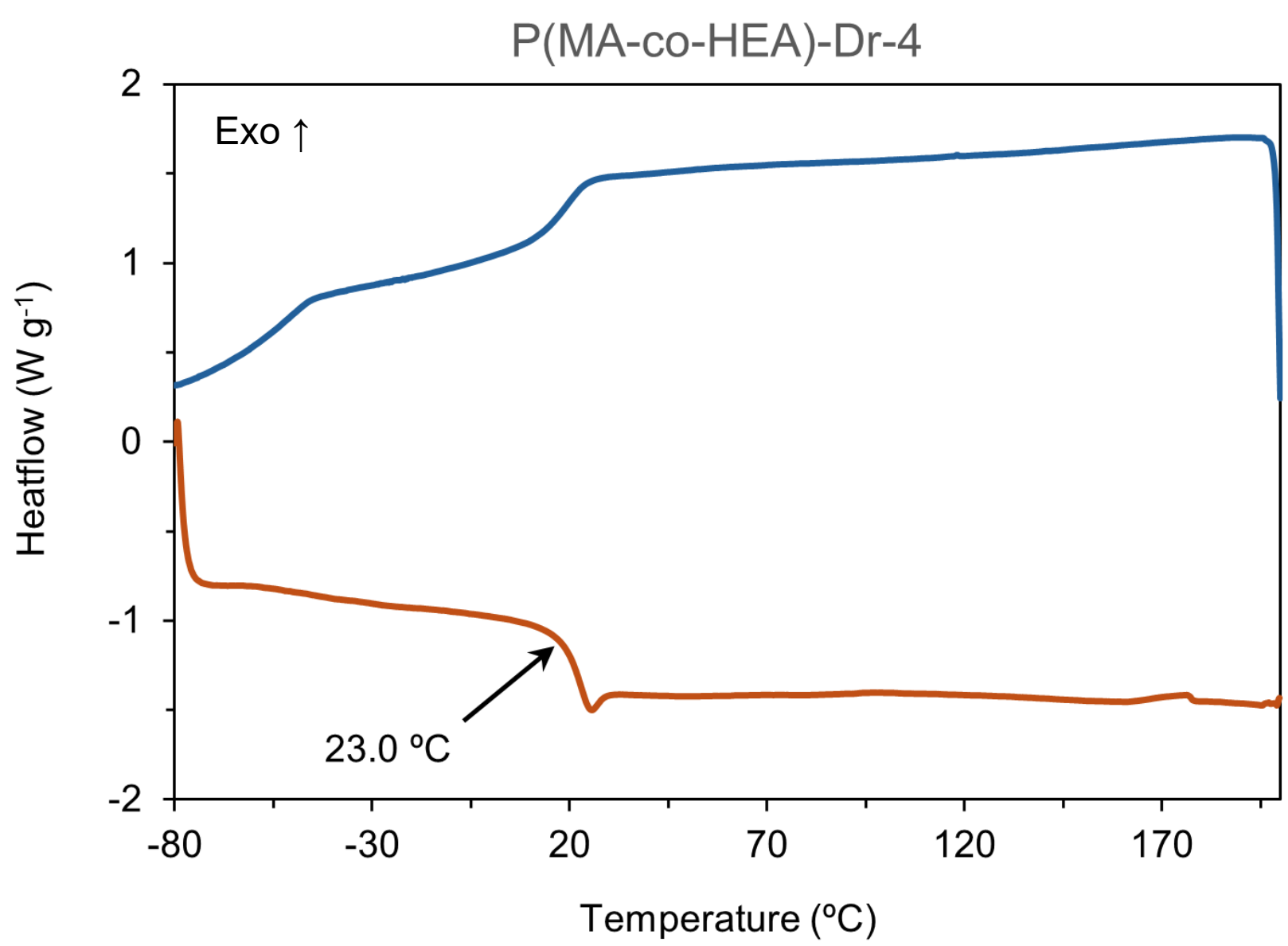

Figure S104: DSC profile (second cycle) of **P(MA-*co*-HEA)-Dr-4**. Heating (red trace) and cooling (blue trace) cycles were carried out at a rate of 10 °C min^{-1}.

Control polymer series

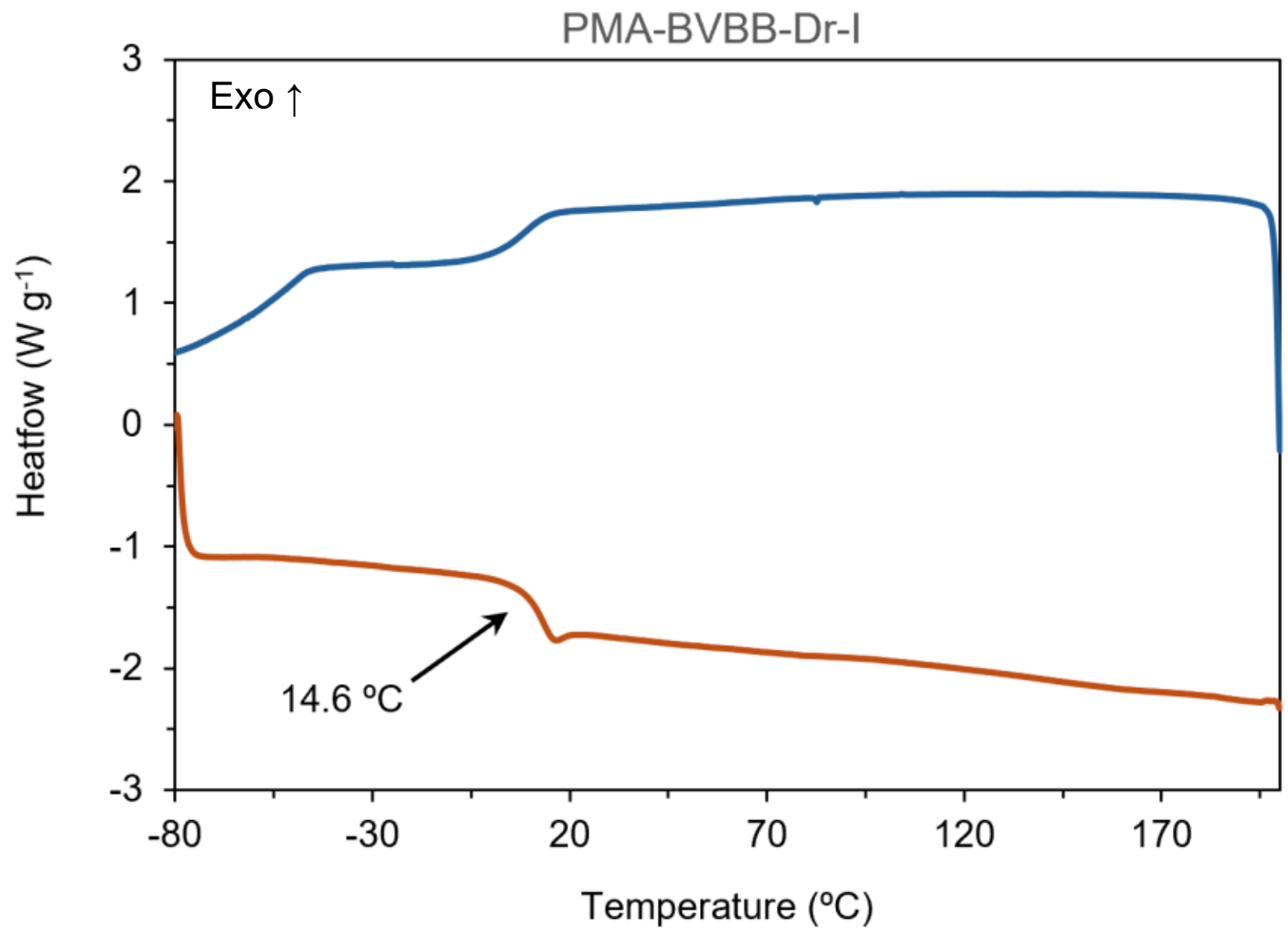

Figure S105: DSC profile (second cycle) of **PMA-BVBB-Dr-I**. Heating (red trace) and cooling (blue trace) cycles were carried out at a rate of 10 °C min^{-1}.

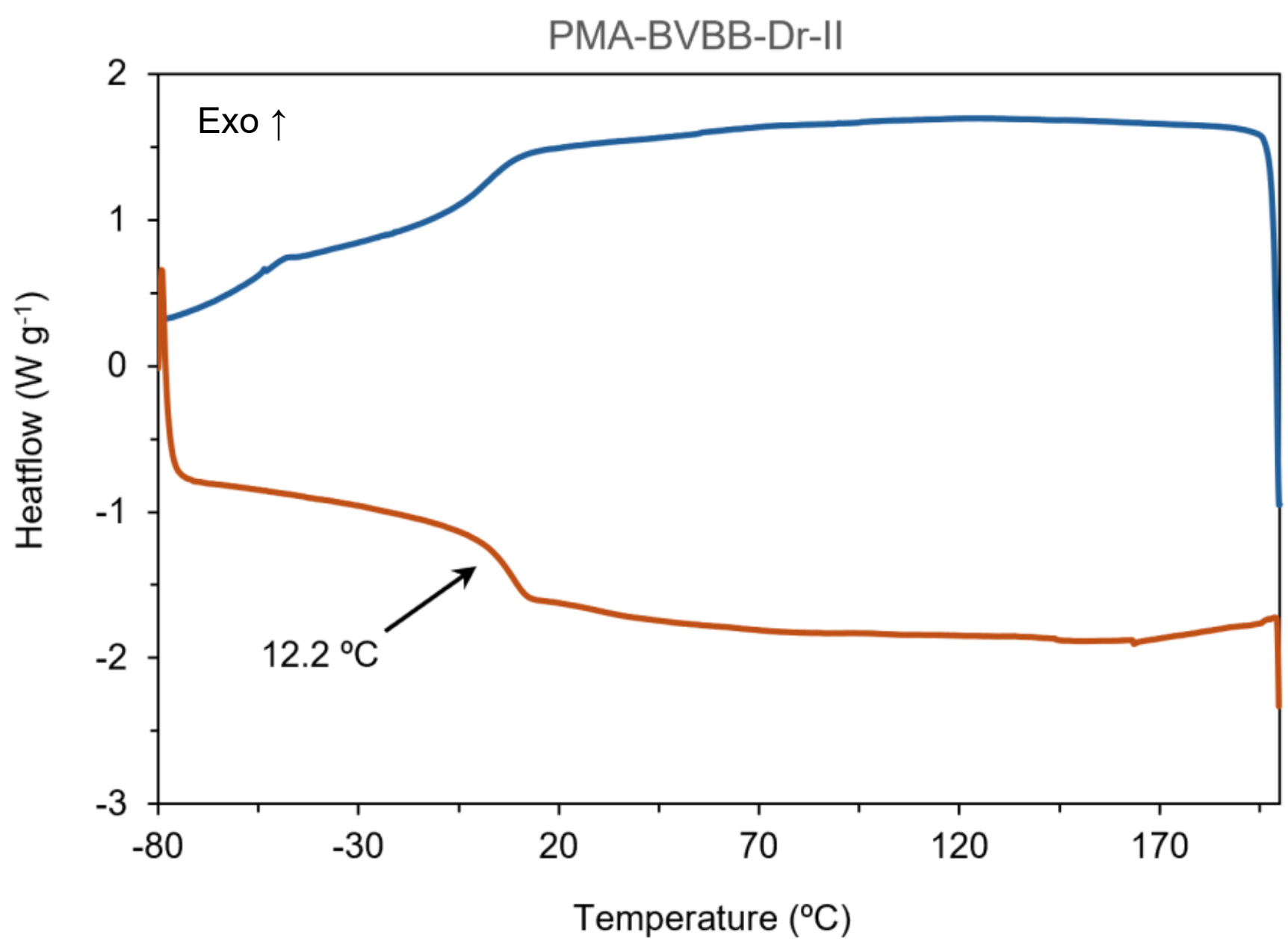

Figure S106: DSC profile (second cycle) of **PMA-BVBB-Dr-II**. Heating (red trace) and cooling (blue trace) cycles were carried out at a rate of 10 °C min^{-1}.

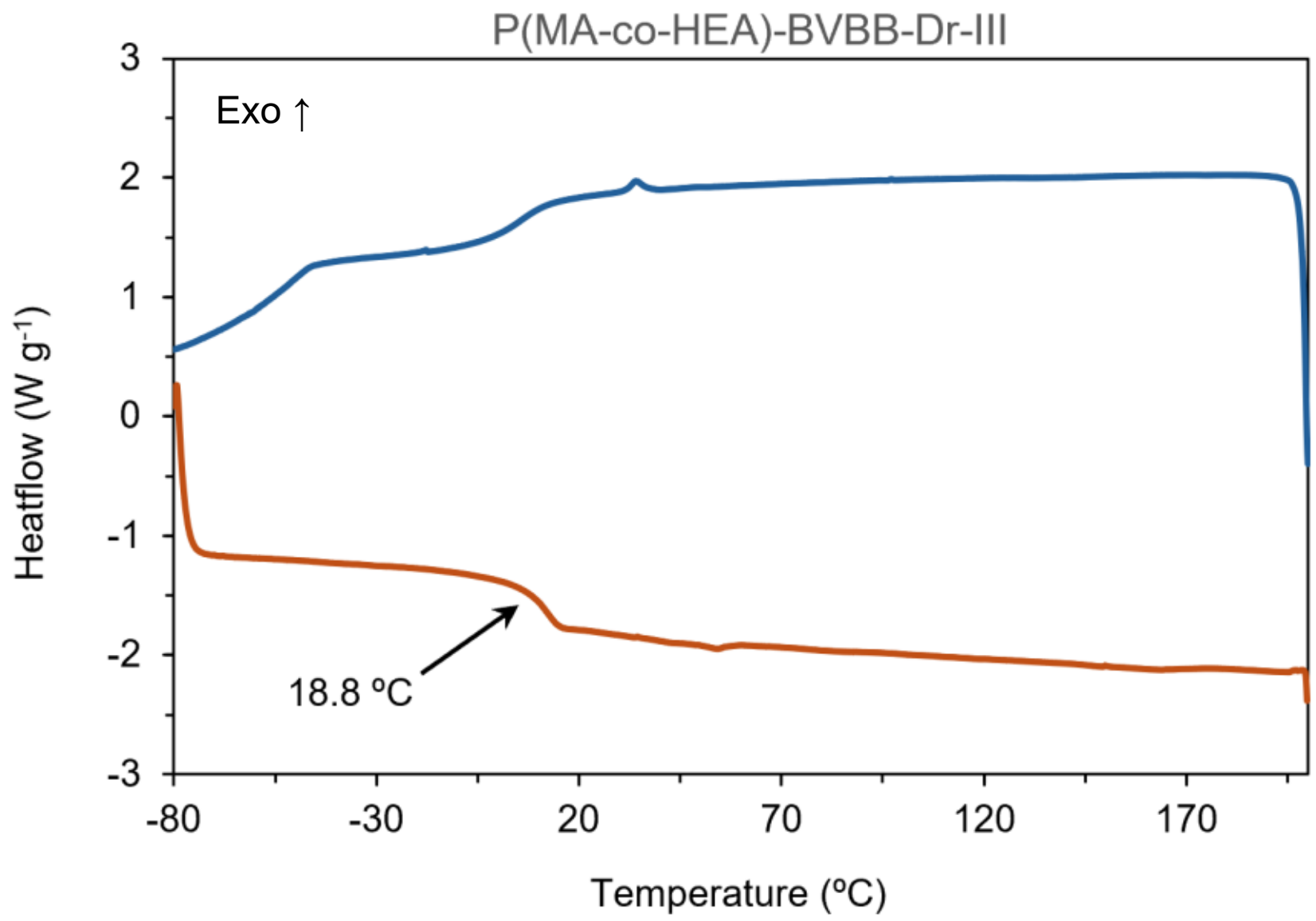

Figure S107: DSC profile (second cycle) of **P(MA-*co*-HEA)-BVBB-Dr-III**. Heating (red trace) and cooling (blue trace) cycles were carried out at a rate of 10 °C min^{-1}.

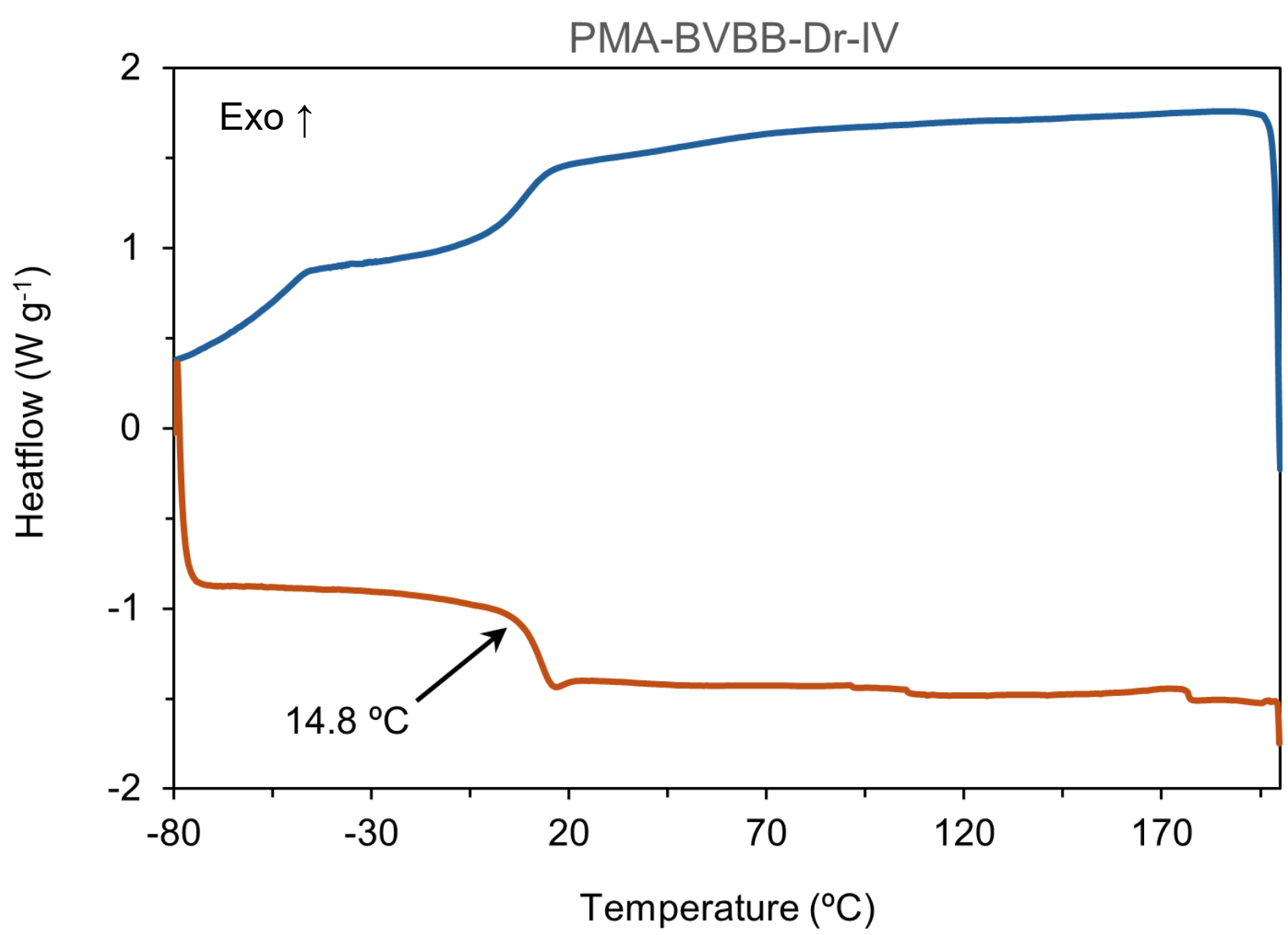

Figure S108: DSC profile (second cycle) of **PMA-BVBB-Dr-IV**. Heating (red trace) and cooling (blue trace) cycles were carried out at a rate of 10 °C min^{-1}.

Electron Paramagnetic Resonance spectroscopy (EPR)

EPR spectra were collected on solid polymer films at ambient temperature under an argon atmosphere. Spectra were recorded for the samples before and after uniaxial stretching. Additional measurements of **PMA-Dr-1**, **PMA-Dr-2**, **P(MA-*co*-HEA)-Dr-3**, and **PMA-Dr-4** were performed while irradiating directly inside the microwave cavity with a UV mercury lamp (λ = 200-2000 nm, power intensity of 100W, irradiation time of 1 hour) to generate paramagnetic species. The weak EPR signal observed before stretching is attributed to the mechanical cutting required to prepare the polymer strip for insertion into the EPR tube. This sample preparation step is expected to generate a small population of carbon-centered radicals at the cut edges, giving rise to the observed background signal.

Unstretched **PMA-Dr-1** films showed no detectable EPR signal attributable to radical species (microwave frequency: $9.855261 \cdot x\ 10^9$ Hz). After stretching, however, a clear resonance with $g = 2.003$ was observed (microwave frequency: $9.855147 \cdot x\ 10^9$ Hz), consistent with carbon-centered organic radicals. In all other polymers, no significant changes in the EPR spectra were detected upon stretching, indicating the absence of selective homolytic bond cleavage.

Spectra shown in the main text were smoothed (window size 45). Below, the non-smoothed spectra of the films before and after stretching are reported for completeness.

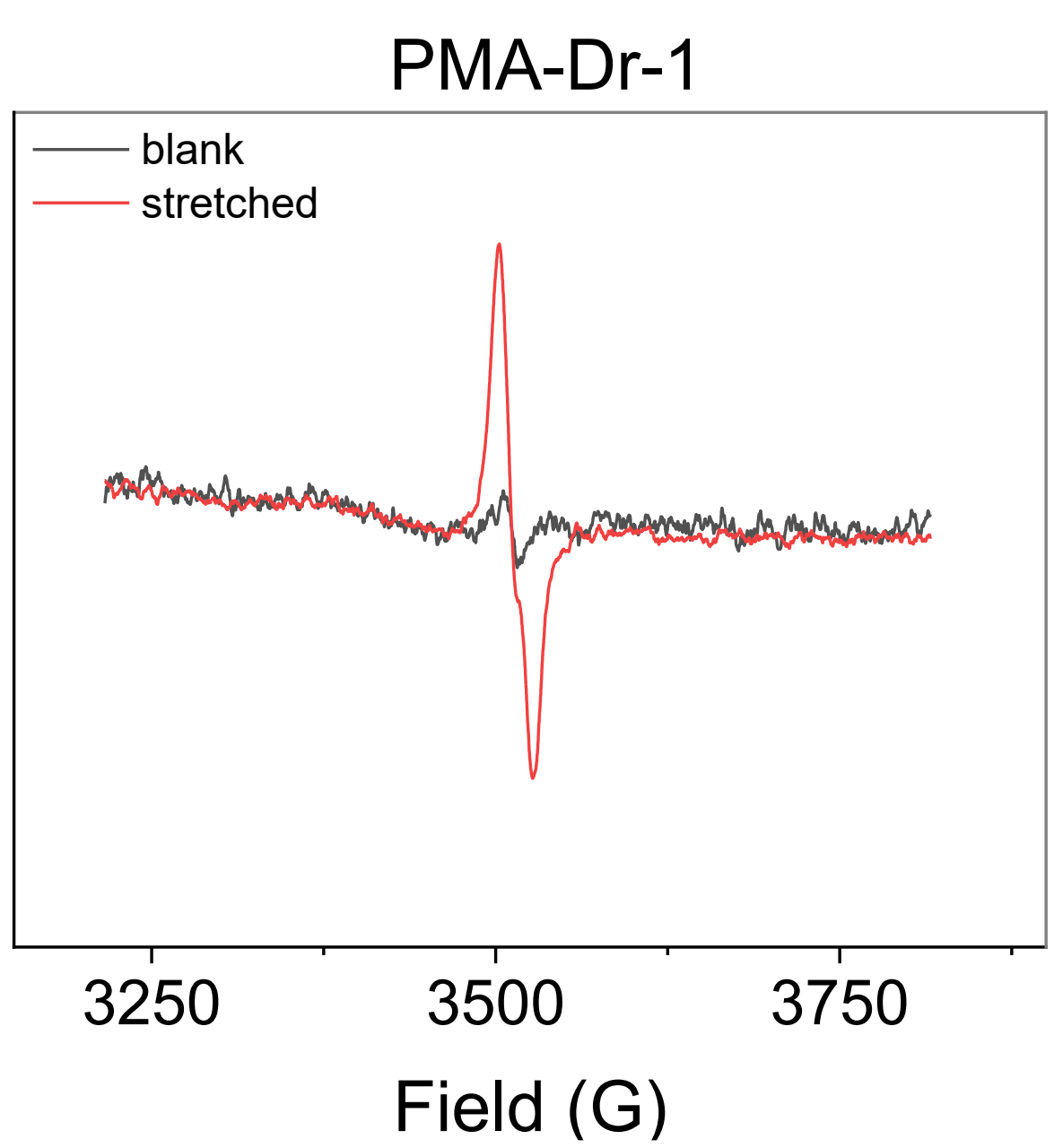

Figure S109: non-smoothed EPR signal of **PMA-Dr-1** films before (black trace, microwave frequency: $9.855261 \cdot x\ 10^9$ Hz) and after (red trace, microwave frequency: $9.855147 \cdot x\ 10^9$ Hz) stretching.

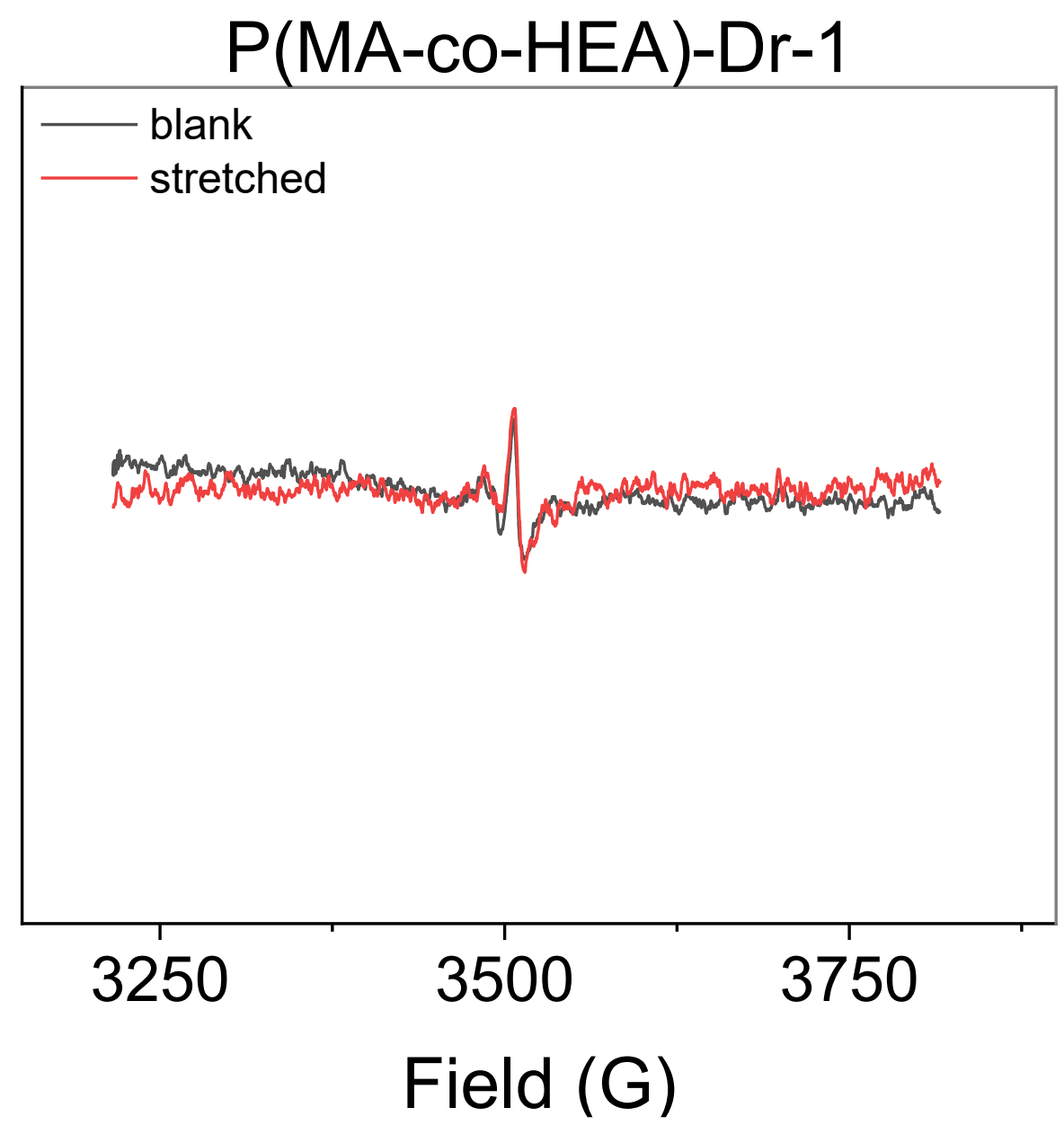

Figure S110: non-smoothed EPR signal of **P(MA-*co*-HEA)-Dr-1** films before (black trace, microwave frequency: 9.85 4393·x 10^9 Hz) and after (red trace, microwave frequency: 9.855036·x 10^9 Hz) stretching.

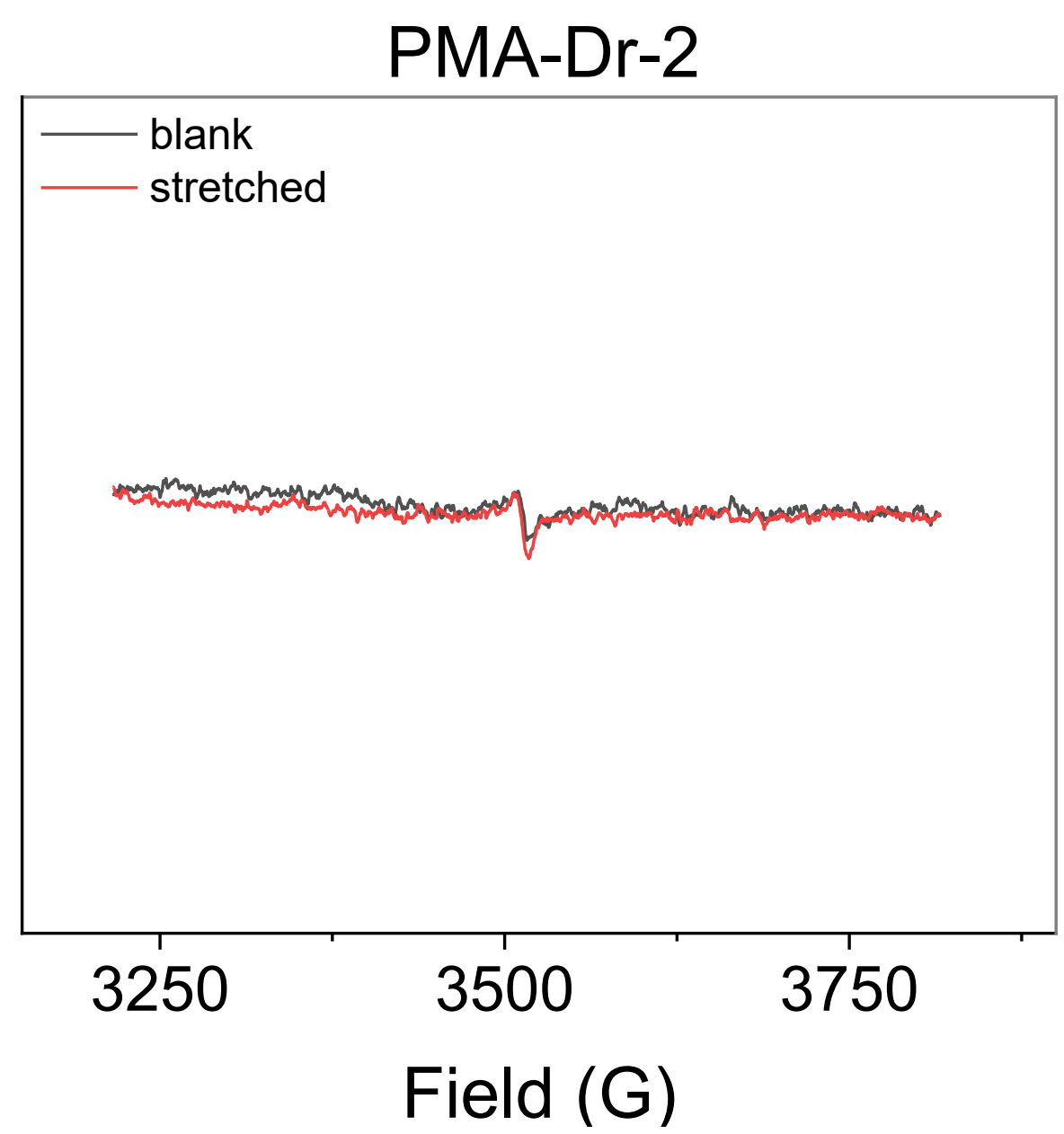

Figure S111: non-smoothed EPR signal of **PMA-Dr-2** films before (black trace, microwave frequency: 9.8554122·x 10^9 Hz) and after (red trace, microwave frequency: 9.8553346·x 10^9 Hz) stretching.

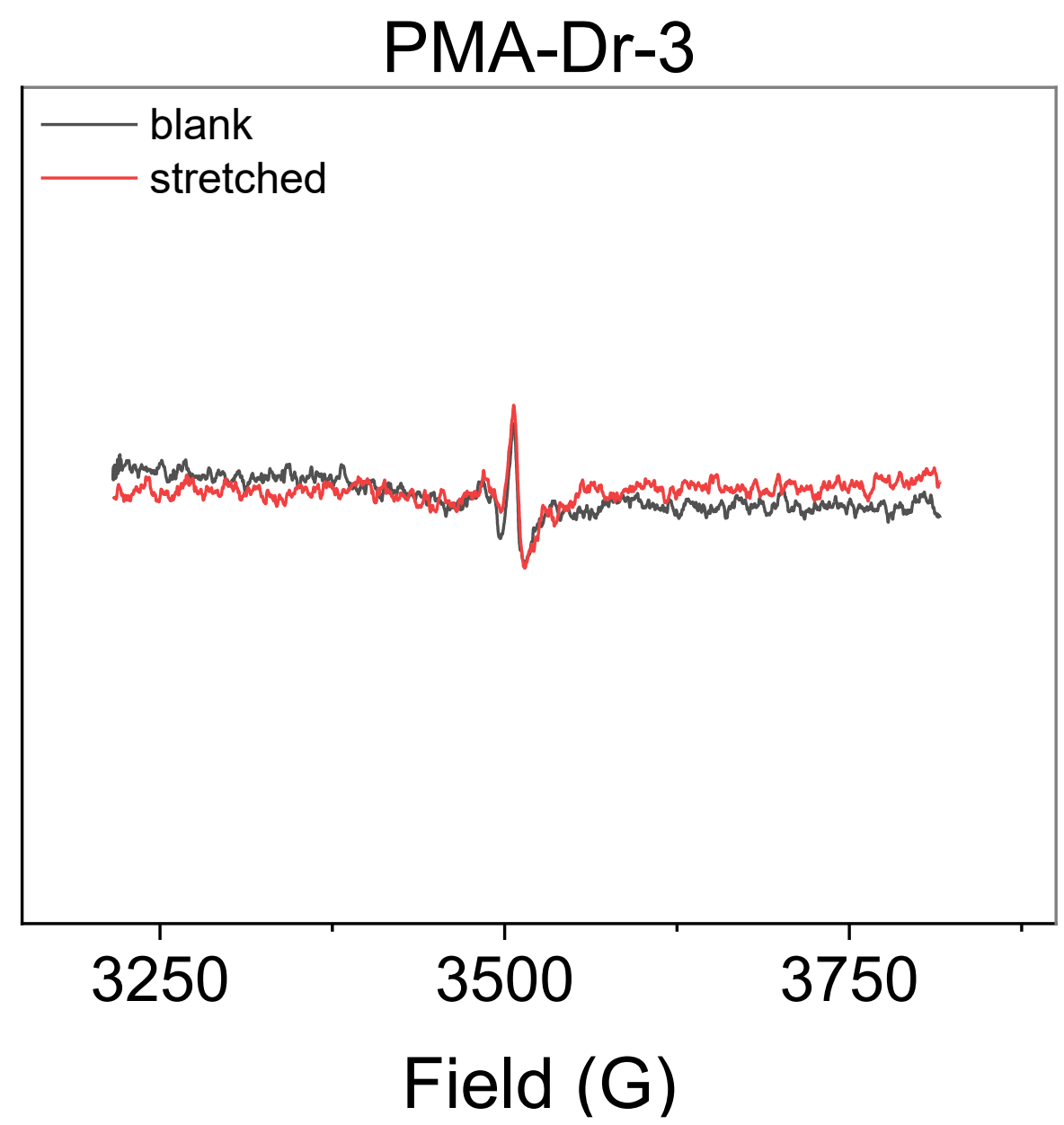

Figure S112: non-smoothed EPR signal of **PMA-Dr-3** films before (black trace, microwave frequency: 9.8554525·x 10^9 Hz) and after (red trace, microwave frequency: 9.854247·x 10^9 Hz) stretching.

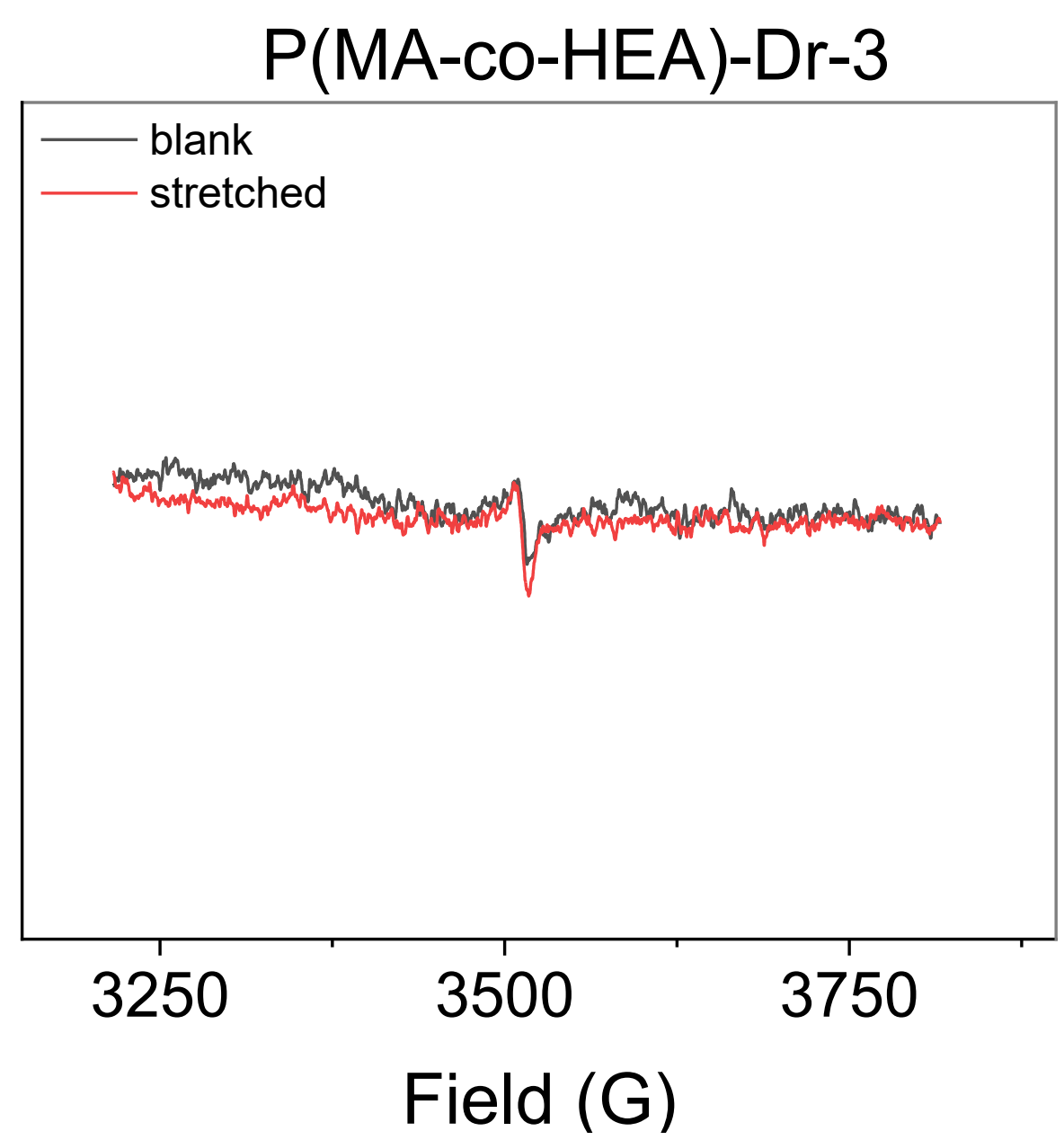

Figure S113: non-smoothed EPR signal **P(MA-*co*-HEA)-Dr-3** films before (black trace, microwave frequency: 9.8554408·x 10^9 Hz) and after (red trace, microwave frequency: 9.854507·x 10^9 Hz) stretching.

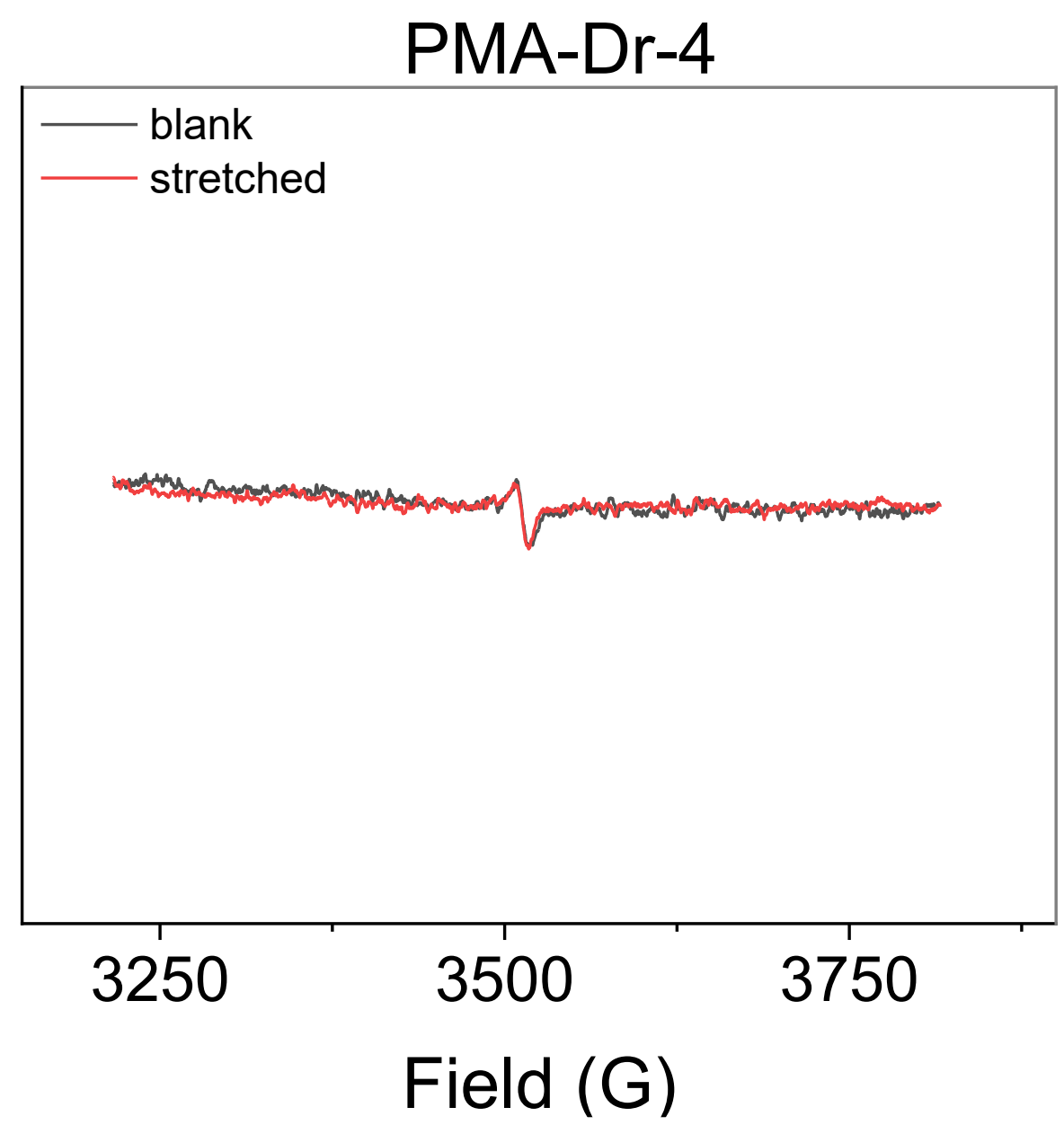

Figure S114: non-smoothed EPR signal of **PMA-Dr-4** films before (black trace, microwave frequency: 9.850813·x 10^9 Hz) and after (red trace, microwave frequency: 9.849946·x 10^9 Hz) stretching.

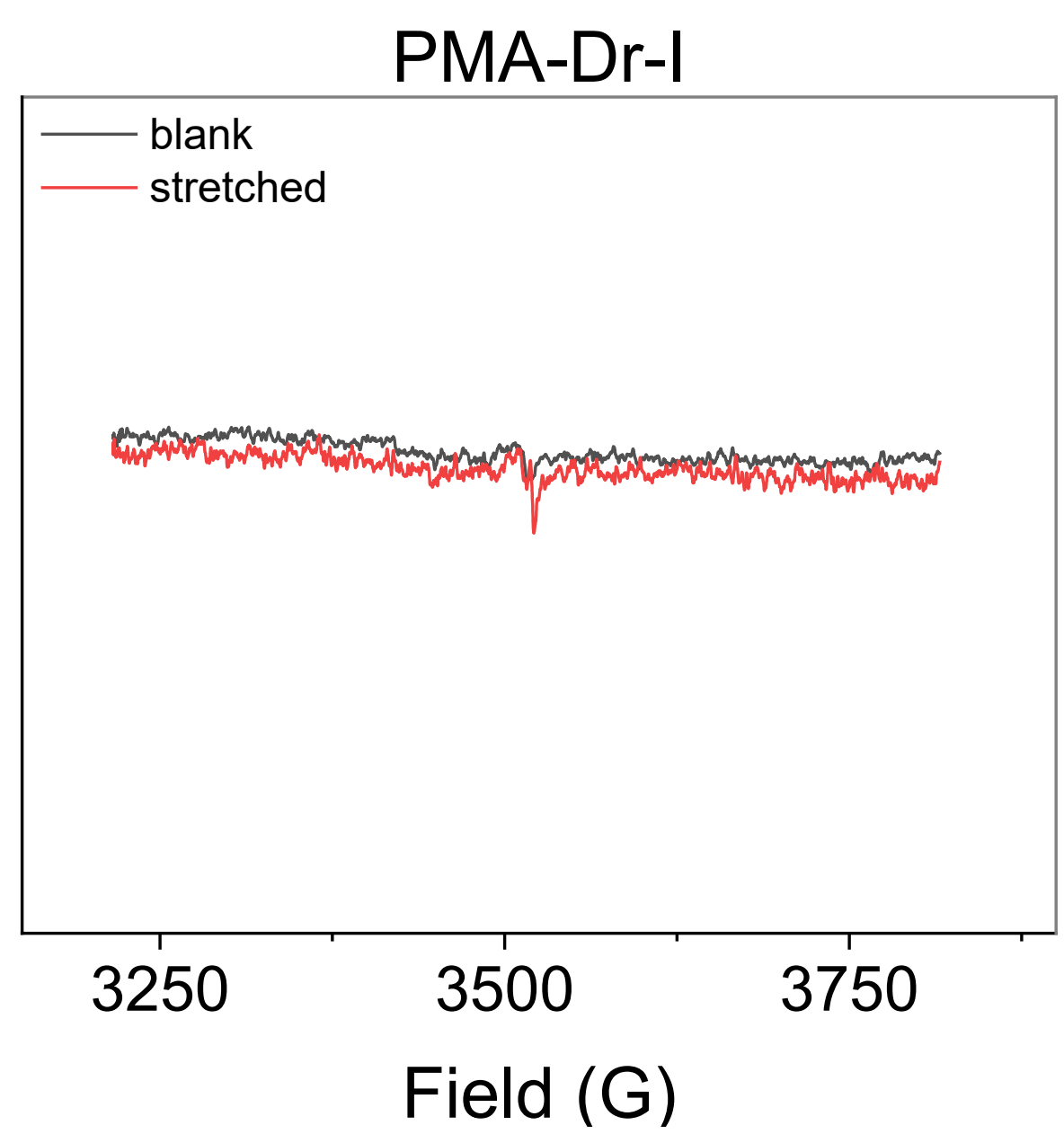

Figure S115: non-smoothed EPR signal of **PMA-Dr-I** films before (black trace, microwave frequency: 9.849933·x 10^9 Hz) and after (red trace, microwave frequency: 9.853485·x 10^9 Hz) stretching.

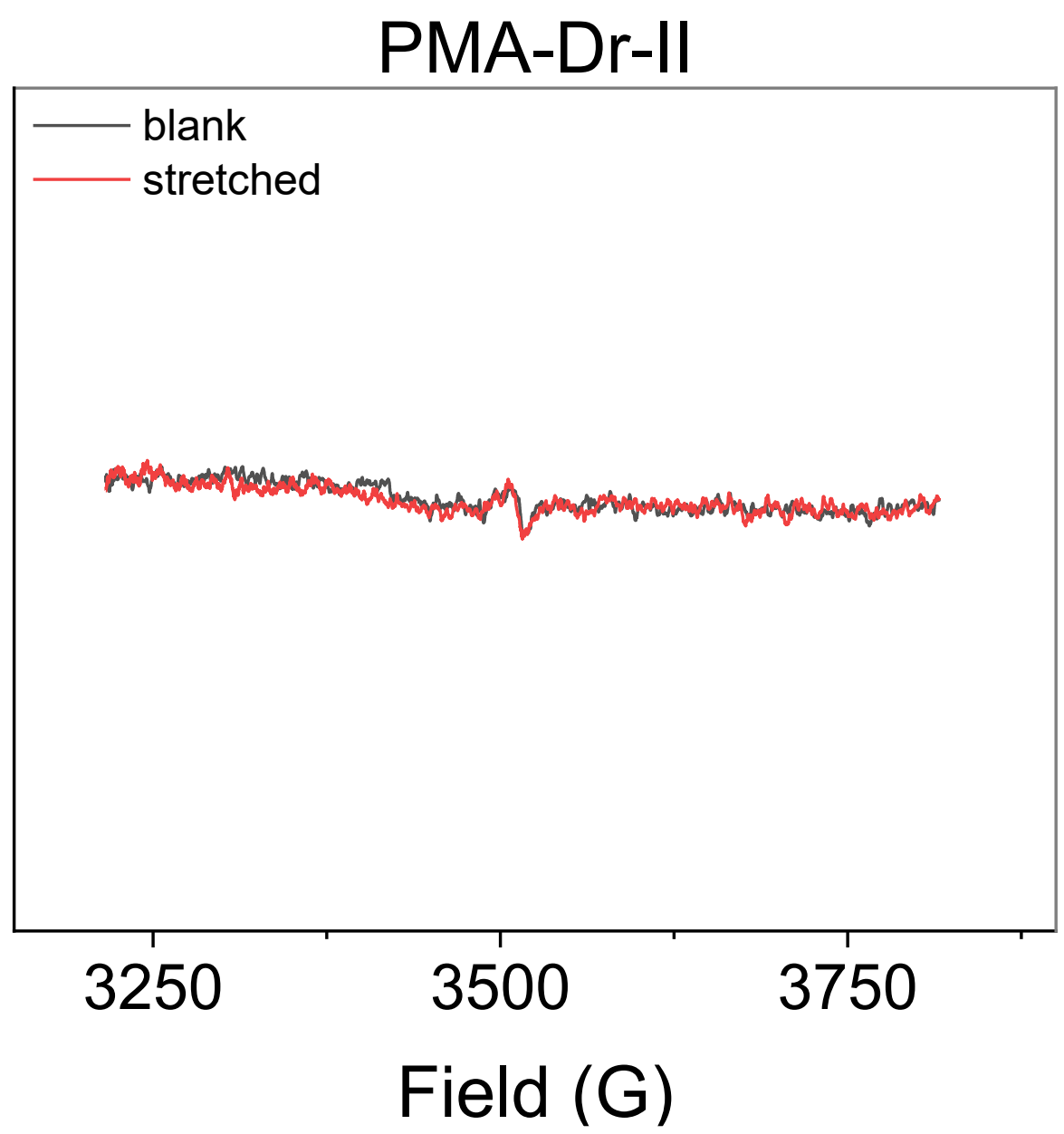

Figure S116: non-smoothed EPR signal of **PMA-Dr-II** films before (black trace, microwave frequency: 9.84995·x 10^9 Hz) and after (red trace, microwave frequency: 9.850416·x 10^9 Hz) stretching.

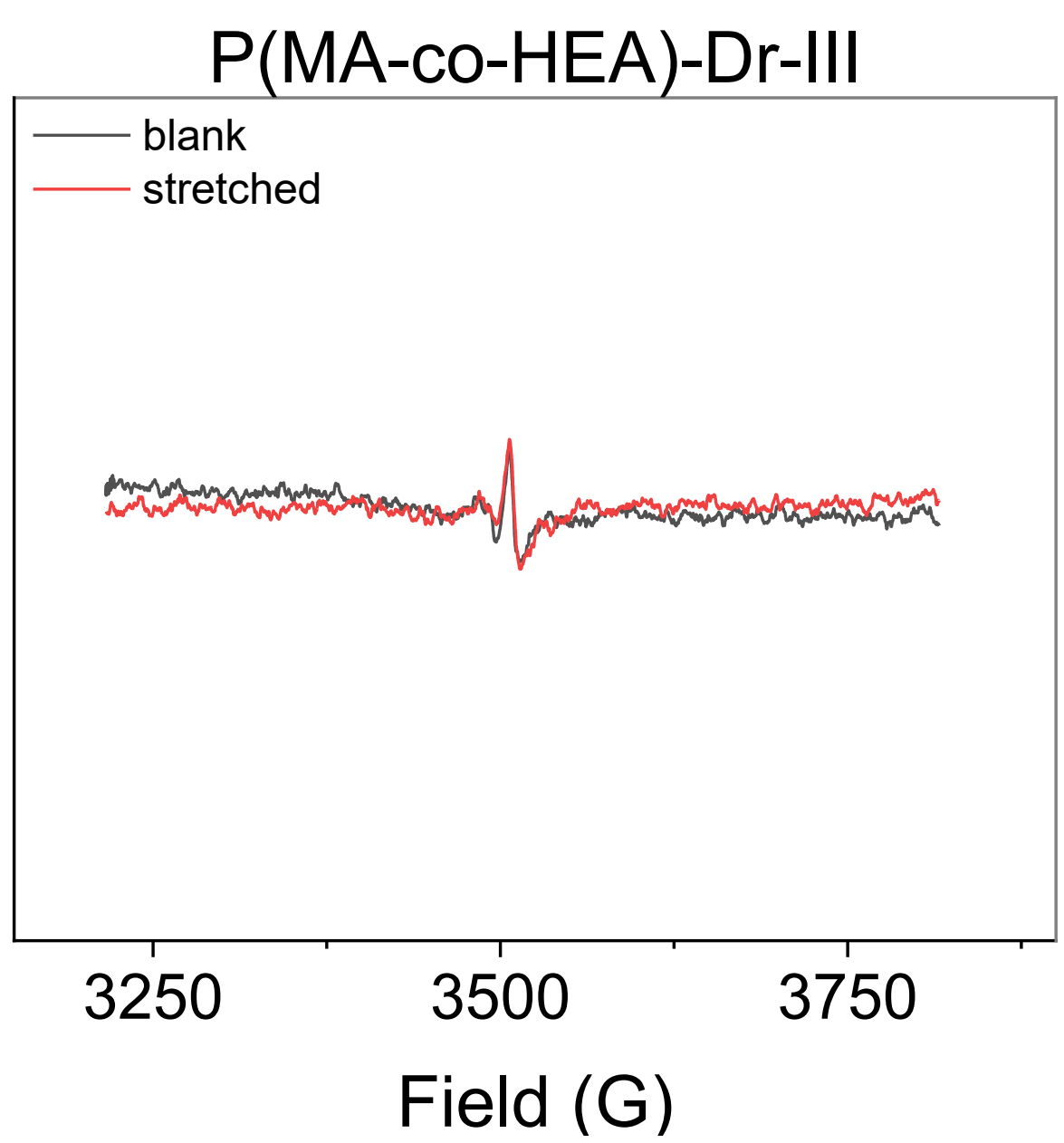

Figure S117: non-smoothed EPR signal of **P(MA-*co*-HEA)-Dr-III** films before (black trace, microwave frequency: 9.850143·x 10^9 Hz) and after (red trace, microwave frequency: 9.854364·x 10^9 Hz) stretching.

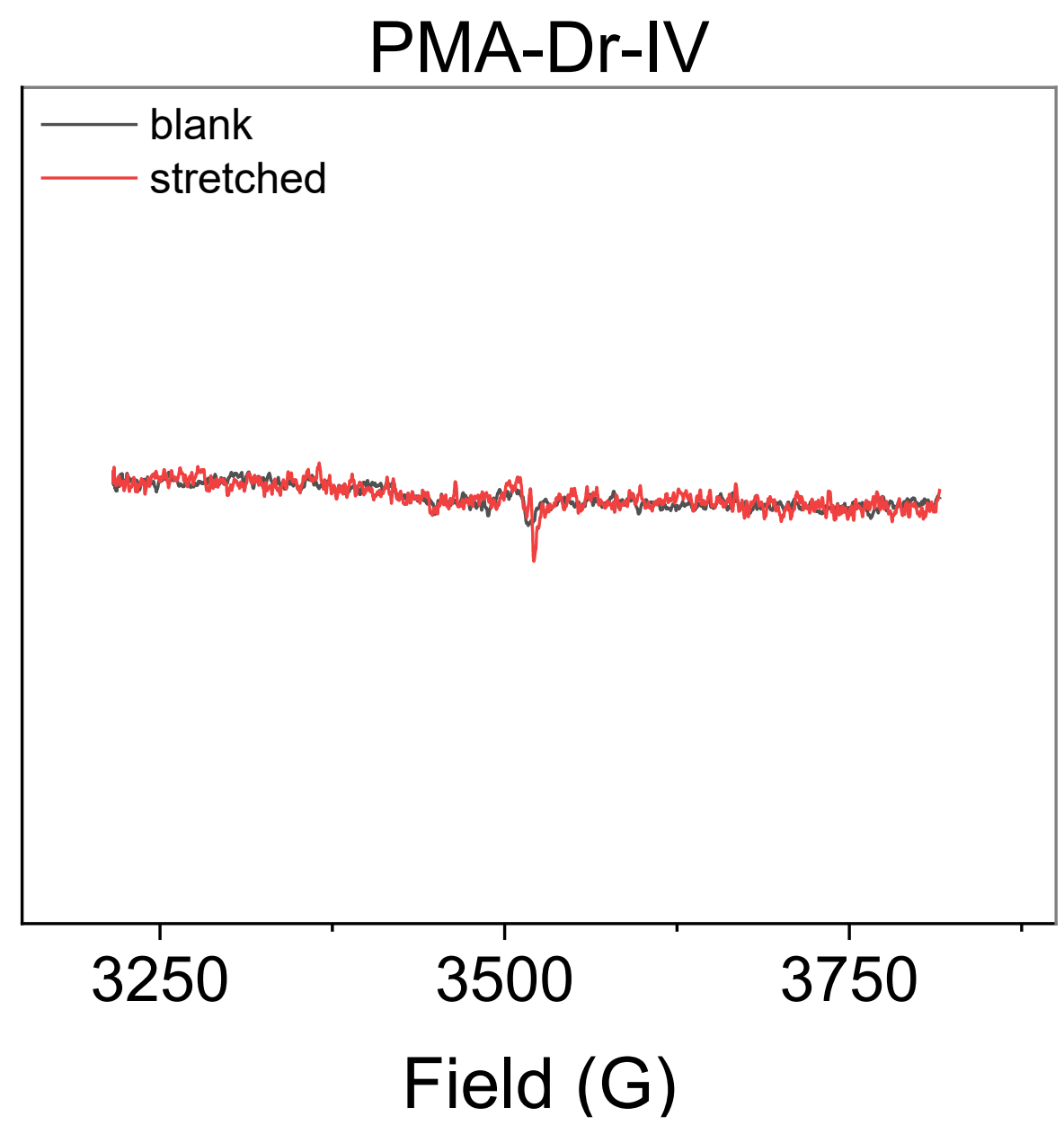

Figure S118: non-smoothed EPR signal of **PMA-Dr-IV** films before (black trace, microwave frequency: 9.854339·x 10^9 Hz) and after (red trace, microwave frequency: 9.839451·x 10^9 Hz) stretching.

After irradiation (λ = 200-2000 nm, power intensity of 100W, irradiation time of 1 hour) of **PMA-Dr-1**, the characteristic orange fluorescence associated with the freshly generated radical species was no longer observed. Instead, only a weak blue fluorescence remained under 365 nm UV illumination, while the film exhibited a slight yellowish coloration in daylight.

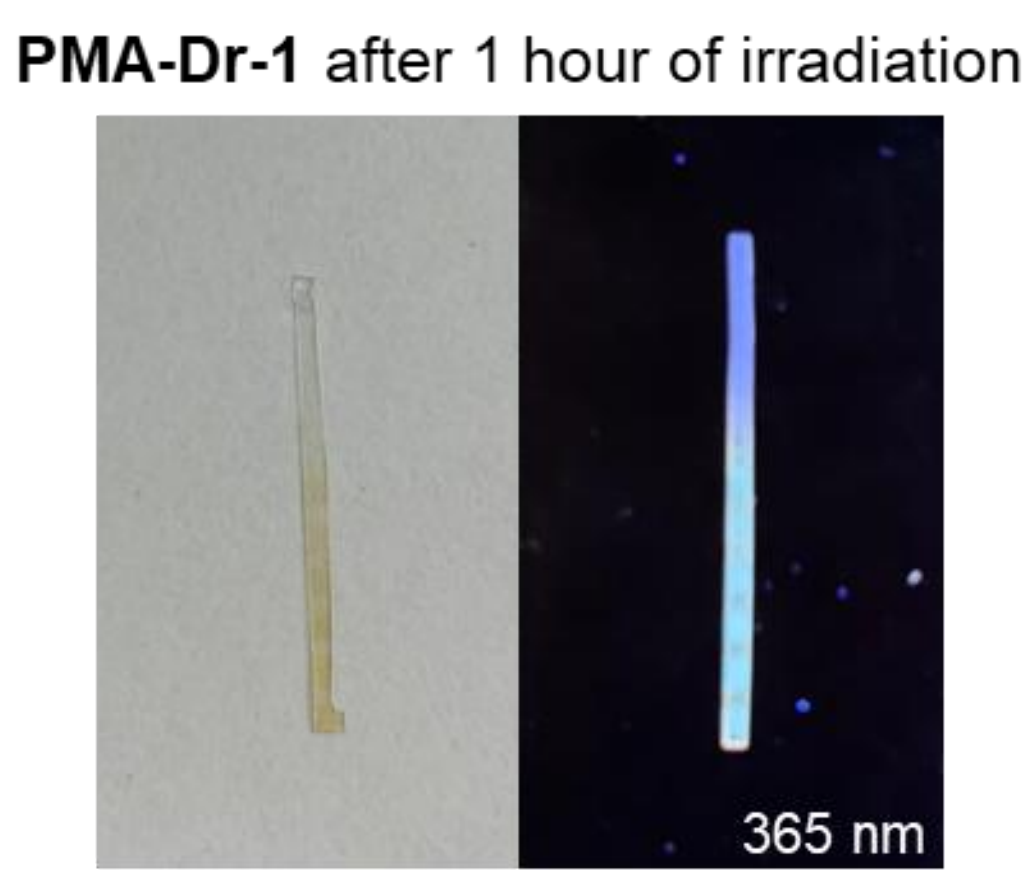

Figure S119: PMA-Dr-1 films after 1 h of continuous Hg lamp irradiation (λ = 200-2000 nm, power intensity of 100W) under ambient atmosphere. Left: photograph under visible light. Right: photograph under 365 nm UV.

Computational results

In the computational modeling of the studied molecules **Dr-A–D**, the different environments were approximated by using the polarizable continuum model (PCM) for several solvents: ethanol (as the strongest hydrogen bond donor), methyl propanoate, methyl butanoate (decreasing in dielectric constant but remaining more polar than PMA), and PMMA (defined by dielectric constant = 3.9 and EpsInf = 2.222) from the square of the refractive index.[8] The linkages between the mechanophore and the polymer were approximated by adding a methyl group on the ends where the polymer is attached.

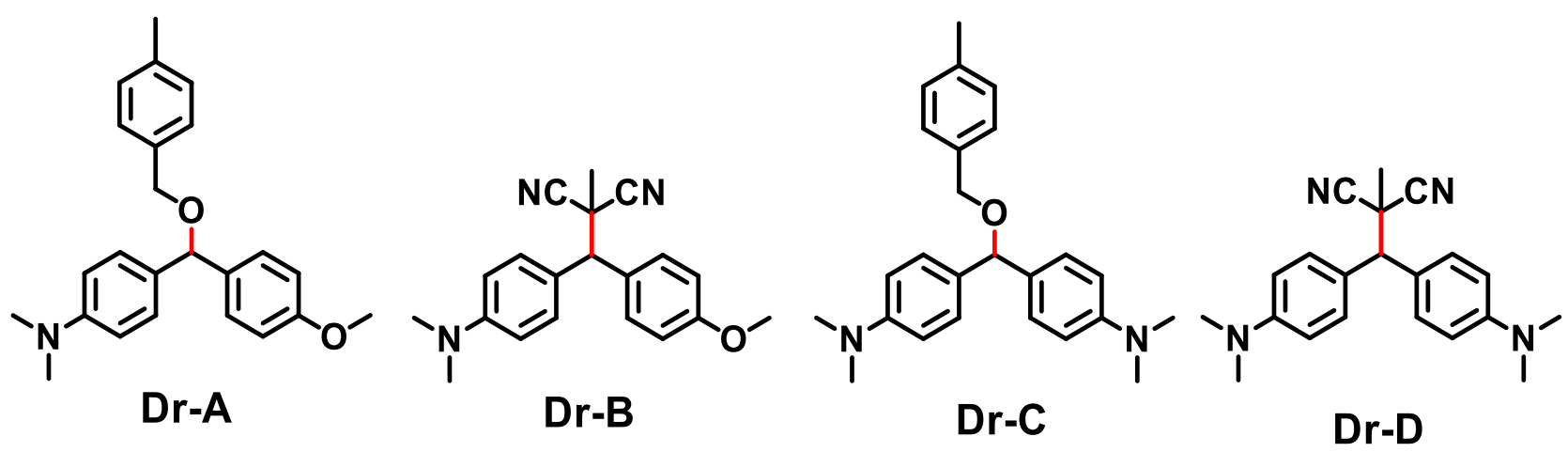

Figure S120: Computed diarylmethane structures.

Table S7: Free energy (kcal/mol) of the C–O or C–C bond cleavage in **Dr-A** and **Dr-B**.

	Benzyl ether **Dr-A**				Malonitrile **Dr-B**		
**Solvent**	**ΔG$_{hom}$**	**ΔG$_{het}$**	**ΔG$_{het·MeOH}$**	**ΔΔG$_{(hom-het)}$**	**ΔG$_{hom}$**	**ΔG$_{het}$**	**ΔΔG$_{(hom-het)}$**
**EtOH**	42.7	32.8	26.7	+9.8	28.2	14.8	+13.4
**$EtCH_2CO_2Me$**	43.4	47.3	(39.7)[a]	-3.8	28.7	26.6	+2.1
**PMMA**	43.9	54.9	(46.6)[a]	-11.0	28.9	32.9	-4.0

[a] At low polarity, no hydrogen bonding moieties are expected to be available in the polymer.

Table S8: Free energy (kcal/mol) of the C–O or C–C bond cleavage in **Dr-C** and **Dr-D**.

	Benzyl ether **Dr-C**				Malonitrile **Dr-D**		
**Solvent**	**ΔG$_{hom}$**	**ΔG$_{het}$**	**ΔG$_{het·MeOH}$**	**ΔΔG$_{(hom-het)}$**	**ΔG$_{hom}$**	**ΔG$_{het}$**	**ΔΔG$_{(hom-het)}$**
**EtOH**	42.9	27.8	21.7	+15.1	28.0	9.3	+18.7
**$EtCH_2CO_2Me$**	43.5	41.2	(33.6)[a]	+2.4	28.5	20.2	+8.3
**PMMA**	43.6	48.4	(40.0)[a]	-4.7	28.6	26.3	+2.3

[a] At low polarity, no hydrogen bonding moieties are expected to be available in the polymer.

Additional computational calculations were performed with the inclusion of applied force using the external force is explicitly included (EFEI) method on model mechanophores **Dr-A**–**Dr-D** (Table S9). Implicit solvation with CPCM for PMMA was modeled with the same parameters as in the thermodynamic calculations.

Application of an external force substantially lowers the dissociation barriers associated with bond cleavage in all systems investigated (Table S9). The calculations support the accessibility of the experimentally observed bond-cleavage events under force and do not reveal alternative competing pathways on the force-modified potential energy surface. These results are therefore consistent with the experimentally observed reactivity trends and support the structure–reactivity relationships discussed in the main text.

Table S9: EFEI activation and reaction energies (kcal/mol) for the mechanochemical cleavage of mechanophores **Dr-A–Dr-D** in PMMA. **Dr-A** and **Dr-C** were modeled at 5.5 nN and referenced to the homolytic cleavage products, while **Dr-B** and **Dr-D** were modeled at 4.5 nN and referenced to the heterolytic cleavage products.

	$\Delta G^{\ddagger}$ / kcal mol^{-1}	ΔG_{r} / kcal mol^{-1}
**Dr-A**	18.4	-45.6
**Dr-B**	11.4	-9.1
**Dr-C**	16.9	-46.4
**Dr-D**	10.0	-15.3

Table S10: Energies reported for the computed structures with the EFEI formalism (ORCA 6.1; LLT: B3LYP-D3(BJ)/def2-SVP TightSCF, CPCM(PMMA); HLT: B3LYP-D3(BJ)/def2-TZVPPD, VeryTightSCF, DefGrid3, CPCM(PMMA)). Where applicable, the force (5.5 nN or 4.5 nN) is appended in the name. All fragment codes match those used in the Gaussian calculations.

	E / Ha	G / Ha	E_{HLT} / Ha
**EFEI_A_5-5_nN**	-1134.43431	-1134.04180	-1135.68418
**EFEI_B_4-5_nN**	-1012.87834	-1012.56575	-1014.00161
**EFEI_C_5-5_nN**	-1153.84700	-1153.41665	-1155.11125
**EFEI_D_4-5_nN**	-1032.29217	-1031.94174	-1033.42969
**EFEI_TSA_5-5_nN**	-1134.39987	-1134.00909	-1135.65314
**EFEI_TSB_4-5_nN**	-1012.85796	-1012.54649	-1013.98237

**EFEI_TSC_5-5_nN**	-1153.81538	-1153.38634	-1155.08301
**EFEI_TSD_4-5_nN**	-1032.27420	-1031.92477	-1033.41281
**EFEIref_dmnome**	-749.53936	-749.28122	-750.36427
**EFEIref_dmn2**	-749.39354	-749.13112	-750.21779
**EFEIref_obn**	-768.95341	-768.65754	-769.79253
**EFEIref_mln**	-768.81735	-768.51680	-769.65605

The naming of the calculated structures as found in the listed coordinates and the dataset in ioChem-BD (at DOI: 10.19061/iochem-bd-1-342) is the following:

compound_(rad/an/cat)_(explicit-solvent)_(implicit-solvent)

The structures listed in Table S9 can be found optimised with ethanol (no suffix), methyl butanoate ("_MeButa"), or poly(methyl methacrylate) ("_PMMA") as implicit solvents. All ether structures have also been optimised with one explicit methanol hydrogen bonding to the oxygen atom. Single-point energy calculations on the optimised structures are prefixed with "SP_".

Table S11: Calculated structures and fragmentation products.

Compound	Code	Fragments (homolytic)	Fragments (heterolytic)
**Dr-A**	ar2dmnomeobn<br>ar2dmnomeobn_meoh	ar2dmnome_rad<br>obn_rad	ar2dmnome_cat<br>obn_an<br>obn_an_meoh
**Dr-B**	ar2dmnomemln	(ar2dmnome_rad)<br>mln_rad	(ar2dmnome_cat)<br>mln_an
**Dr-C**	ar2dmn2obn<br>ar2dmn2obn_meoh	ar2dmn2_rad<br>(obn_rad)	ar2dmn2_cat<br>(obn_an)<br>(obn_an_meoh)
**Dr-D**	ar2dmn2mln	(ar2dmn2_rad)<br>(mln_rad)	(ar2dmn2_cat)<br>(mln_an)

Atomic coordinates of the calculated structures

SP_mln_an
Energy (POTENTIAL) = -263.9143511 Eh

	Atom	X	Y	Z
1	C	-0.0383	0.2689	-0.0327
2	C	-1.1637	-0.5406	0.1188
3	N	-2.1310	-1.2065	0.2379
4	C	1.2149	-0.3364	-0.1250
5	C	-0.1635	1.7855	0.0015
6	H	-0.0602	2.2155	1.0108
7	H	0.6018	2.2564	-0.6281
8	H	-1.1397	2.1030	-0.3848
9	N	2.2852	-0.8259	-0.2139

SP_ar2dmnomemln_MeButa
Energy (POTENTIAL) = -1014.5187587 Eh

	Atom	X	Y	Z
1	C	-0.9937	-0.3803	0.4413
2	C	-0.4727	1.0398	0.2827
3	C	-0.1210	1.7569	1.4338
4	C	0.3976	3.0457	1.3765
5	C	0.5940	3.6977	0.1357
6	C	0.2160	2.9831	-1.0261
7	C	-0.2934	1.6910	-0.9470
8	H	-0.5686	1.2018	-1.8755
9	H	0.3163	3.4336	-2.0058
10	H	0.6434	3.5444	2.3061
11	H	-0.2541	1.2963	2.4105
12	C	-2.3662	-0.6434	-0.3339
13	C	-2.1644	-0.7800	-1.7905
14	N	-2.0175	-0.8670	-2.9380
15	C	0.0335	-1.4734	0.1670
16	C	0.9024	-1.4368	-0.9361
17	C	1.8095	-2.4616	-1.1689
18	C	1.8797	-3.5592	-0.2968
19	C	1.0291	-3.6093	0.8138
20	C	0.1210	-2.5701	1.0303
21	H	-0.5350	-2.6250	1.8953
22	H	1.0624	-4.4377	1.5113
23	H	2.4804	-2.4317	-2.0218
24	H	0.8820	-0.5938	-1.6176
25	N	1.1320	4.9706	0.0604
26	C	1.1109	5.6789	-1.2111
27	C	1.3057	5.7442	1.2808
28	H	0.0895	5.8276	-1.5947
29	H	1.6846	5.1370	-1.9721
30	H	0.3552	5.9209	1.8082
31	H	1.9895	5.2382	1.9724
32	C	2.9109	-5.6527	0.2308
33	H	3.1986	-5.3727	1.2523
34	H	1.9700	-6.2167	0.2612
35	H	1.7452	6.7113	1.0321
36	H	1.5755	6.6578	-1.0837
37	H	-1.2946	-0.4825	1.4898
38	H	3.6926	-6.2753	-0.2073
39	C	-2.9232	-1.9266	0.1443
40	N	-3.3753	-2.9100	0.5616
41	C	-3.4017	0.4795	-0.0526
42	H	-3.5289	0.5827	1.0283
43	H	-3.0340	1.4235	-0.4569
44	H	-4.3659	0.2398	-0.5068
45	O	2.8049	-4.5091	-0.6127

FLUOfl_rad_MeButa
Energy (POTENTIAL) = -771.3695579 Eh

	Atom	X	Y	Z
1	C	-0.0008	-0.0017	-0.0899
2	C	-0.0648	-1.1246	-0.9388
3	C	-0.0366	-0.6842	-2.3490
4	C	-0.1812	-2.5095	-0.6655
5	C	-0.0978	-1.6481	-3.4046
6	C	-0.2429	-3.3928	-1.7116
7	H	-0.2303	-2.8638	0.3580
8	C	-0.1932	-2.9691	-3.0912
9	H	-0.0706	-1.3194	-4.4389
10	H	-0.3370	-4.4544	-1.5041
11	C	-0.0019	-0.0049	1.3803
12	C	-0.8120	0.8766	2.1091
13	C	0.8034	-0.8915	2.1071
14	C	-0.8094	0.8743	3.4993
15	H	-1.4699	1.5571	1.5773
16	C	0.7949	-0.8972	3.4978
17	H	1.4577	-1.5746	1.5743
18	C	-0.0061	-0.0114	4.2215
19	H	-1.4545	1.5670	4.0338
20	H	1.4312	-1.5990	4.0307
21	C	0.0647	1.1258	-0.9326
22	C	0.1794	2.5091	-0.6515
23	C	0.0399	0.6929	-2.3452
24	C	0.2431	3.3982	-1.6926
25	H	0.2256	2.8579	0.3741
26	C	0.1033	1.6626	-3.3954
27	C	0.1973	2.9819	-3.0746
28	H	0.0789	1.3395	-4.4316
29	H	0.3359	4.4588	-1.4791
30	C	0.0154	0.0088	5.7278
31	H	0.7475	0.7346	6.0988
32	H	-0.9590	0.2900	6.1361
33	H	0.2869	-0.9683	6.1357
34	H	0.2464	3.7383	-3.8501
35	H	-0.2408	-3.7213	-3.8709

SP_ar2dmn2obn_meoh_MeButa
Energy (POTENTIAL) = -1271.5802529 Eh

	Atom	X	Y	Z
1	C	-0.2976	-0.3292	-1.1005
2	C	-1.7086	-0.3803	-0.5459
3	C	-2.7249	0.3279	-1.2018
4	C	-2.0430	-1.0181	0.6551
5	C	-4.0189	0.3893	-0.6987
6	H	-2.4917	0.8686	-2.1161
7	C	-3.3324	-0.9714	1.1751
8	H	-1.2827	-1.5754	1.1950
9	C	-4.3614	-0.2580	0.5139
10	H	-4.7642	0.9452	-1.2544
11	H	-3.5342	-1.4943	2.1021
12	C	0.5940	-1.5249	-0.7901

13	C	1.9579	-1.3676	-0.5195
14	C	0.1026	-2.8349	-0.8559
15	C	2.7931	-2.4596	-0.2986
16	H	2.3782	-0.3678	-0.4765
17	C	0.9235	-3.9391	-0.6484
18	H	-0.9503	-3.0016	-1.0660
19	C	2.3016	-3.7837	-0.3668
20	H	3.8366	-2.2710	-0.0764
21	H	0.4807	-4.9264	-0.7030
22	N	3.1343	-4.8807	-0.1791
23	C	4.4661	-4.6660	0.3674
24	C	2.5362	-6.1963	-0.0058
25	H	4.4466	-4.1890	1.3606
26	H	5.0662	-4.0363	-0.2993
27	H	1.8614	-6.2467	0.8639
28	H	1.9652	-6.4865	-0.8952
29	N	-5.6429	-0.1872	1.0342
30	C	-6.7118	0.3779	0.2240
31	C	-6.0030	-1.0352	2.1605
32	H	-6.8656	-0.1734	-0.7168
33	H	-6.5042	1.4250	-0.0264
34	H	-5.8889	-2.1072	1.9356
35	H	-5.3883	-0.8056	3.0391
36	O	0.3660	0.9170	-0.7438
37	C	0.3776	1.2456	0.6604
38	H	-0.6278	1.5608	0.9662
39	H	0.6457	0.3595	1.2470
40	C	1.3837	2.3469	0.8582
41	C	2.6189	2.0958	1.4624
42	C	1.1100	3.6451	0.4039
43	C	3.5581	3.1180	1.6176
44	H	2.8505	1.0922	1.8125
45	C	2.0521	4.6601	0.5534
46	H	0.1644	3.8492	-0.0921
47	C	3.2904	4.4166	1.1683
48	H	4.5127	2.9019	2.0920
49	H	1.8248	5.6599	0.1896
50	C	4.2905	5.5330	1.3574
51	H	4.3218	6.1939	0.4839
52	H	4.0277	6.1555	2.2230
53	H	5.2999	5.1443	1.5273
54	C	0.1603	3.3640	-3.0754
55	H	-0.2450	4.1579	-3.7113
56	H	1.0217	3.7702	-2.5233
57	H	0.5234	2.5545	-3.7279
58	O	-0.8781	2.9342	-2.2064
59	H	-0.4918	2.2253	-1.6491
60	H	-0.3705	-0.2301	-2.1911
61	H	4.9758	-5.6269	0.4571
62	H	3.3283	-6.9349	0.1299
63	H	-7.6430	0.3537	0.7921
64	H	-7.0441	-0.8504	2.4301

SP_ar2dmnomeobn_PMMA
Energy (POTENTIAL) = -1136.3433808 Eh

	Atom	X	Y	Z
1	C	-0.3580	-0.5105	-1.3500
2	C	-1.4318	-1.4603	-0.8385
3	C	-2.5299	-1.7571	-1.6612
4	C	-1.4278	-1.9843	0.4576
5	C	-3.5769	-2.5515	-1.2129
6	H	-2.5632	-1.3541	-2.6708
7	C	-2.4717	-2.7846	0.9296
8	H	-0.5896	-1.7752	1.1156
9	C	-3.5552	-3.0725	0.0902
10	H	-4.4225	-2.7859	-1.8523
11	H	-2.4275	-3.1726	1.9405
12	C	1.0624	-0.7962	-0.8674
13	C	1.9696	0.2493	-0.6605
14	C	1.5451	-2.1006	-0.7069
15	C	3.2895	0.0145	-0.2865
16	H	1.6306	1.2721	-0.7904
17	C	2.8641	-2.3564	-0.3423
18	H	0.8772	-2.9436	-0.8619
19	C	3.7811	-1.3017	-0.1252
20	H	3.9377	0.8675	-0.1248
21	H	3.1739	-3.3878	-0.2242
22	N	5.1065	-1.5479	0.2176
23	C	5.9277	-0.4409	0.6840
24	C	5.4869	-2.8904	0.6307
25	H	5.5210	0.0411	1.5879
26	H	6.0285	0.3257	-0.0926
27	H	4.9302	-3.2379	1.5161
28	H	5.3209	-3.6107	-0.1785
29	C	-4.6604	-4.3977	1.7479
30	H	-3.8100	-5.0696	1.9225
31	H	-4.6650	-3.6135	2.5160
32	O	-0.7227	0.8678	-1.1348
33	C	-1.0035	1.2281	0.2238
34	H	-1.9985	0.8577	0.5072
35	H	-0.2734	0.7705	0.9040
36	C	-0.9392	2.7294	0.3215
37	C	0.1801	3.3707	0.8622
38	C	-1.9864	3.5178	-0.1709
39	C	0.2481	4.7639	0.9165
40	H	1.0073	2.7741	1.2405
41	C	-1.9161	4.9087	-0.1194
42	H	-2.8599	3.0345	-0.6024
43	C	-0.7989	5.5562	0.4290
44	H	1.1274	5.2426	1.3420
45	H	-2.7394	5.5036	-0.5090
46	C	-0.7422	7.0637	0.5165
47	H	-1.2594	7.5338	-0.3270
48	H	-1.2248	7.4245	1.4346
49	H	0.2912	7.4262	0.5276
50	H	-5.5892	-4.9674	1.8077
51	H	6.9297	-0.8094	0.9119
52	H	6.5525	-2.9040	0.8669
53	H	-0.3596	-0.5763	-2.4458
54	O	-4.6263	-3.8434	0.4371

ar2dmn2obn_MeButa
Energy (POTENTIAL) = -1155.49567195 Eh

	Atom	X	Y	Z
1	C	-0.3296	-0.1462	-1.3159
2	C	-1.7565	-0.0875	-0.7983
3	C	-2.7771	0.3912	-1.6281
4	C	-2.1087	-0.4156	0.5178
5	C	-4.0866	0.5372	-1.1821
6	H	-2.5450	0.6557	-2.6579
7	C	-3.4103	-0.2752	0.9878
8	H	-1.3508	-0.8058	1.1914

9	C	-4.4403	0.2187	0.1512
10	H	-4.8339	0.8981	-1.8786
11	H	-3.6216	-0.5547	2.0129
12	C	0.4981	-1.3386	-0.8393
13	C	1.8875	-1.2313	-0.7028
14	C	-0.0677	-2.5986	-0.6115
15	C	2.6773	-2.3159	-0.3328
16	H	2.3598	-0.2707	-0.8826
17	C	0.7060	-3.6983	-0.2489
18	H	-1.1415	-2.7303	-0.7105
19	C	2.1083	-3.5902	-0.1020
20	H	3.7447	-2.1625	-0.2265
21	H	0.2060	-4.6438	-0.0765
22	N	2.8919	-4.6875	0.2405
23	C	4.2759	-4.4655	0.6320
24	C	2.2371	-5.8902	0.7337
25	H	4.3706	-3.8067	1.5104
26	H	4.8478	-4.0193	-0.1895
27	H	1.6391	-5.7085	1.6415
28	H	1.5748	-6.3162	-0.0286
29	N	-5.7350	0.3877	0.6186
30	C	-6.7994	0.6791	-0.3299
31	C	-6.1018	-0.1723	1.9109
32	H	-6.9192	-0.1100	-1.0890
33	H	-6.6122	1.6255	-0.8507
34	H	-5.9630	-1.2641	1.9546
35	H	-5.5093	0.2774	2.7164
36	O	0.3751	1.0985	-1.1049
37	C	0.4493	1.5410	0.2555
38	H	-0.5209	1.9518	0.5672
39	H	0.6824	0.6999	0.9218
40	C	1.5289	2.5877	0.3382
41	C	2.8225	2.2565	0.7561
42	C	1.2716	3.9083	-0.0491
43	C	3.8306	3.2211	0.7909
44	H	3.0423	1.2335	1.0532
45	C	2.2797	4.8706	-0.0164
46	H	0.2729	4.1808	-0.3829
47	C	3.5763	4.5445	0.4076
48	H	4.8295	2.9424	1.1197
49	H	2.0593	5.8910	-0.3229
50	C	4.6572	5.5980	0.4765
51	H	4.5486	6.3368	-0.3253
52	H	4.6156	6.1447	1.4281
53	H	5.6555	5.1547	0.3987
54	H	-7.7422	0.7823	0.2099
55	H	-7.1512	0.0488	2.1125
56	H	4.7392	-5.4249	0.8697
57	H	2.9947	-6.6409	0.9656
58	H	-0.3806	-0.1896	-2.4117

ar2dmnome_cat_PMMA
Energy (POTENTIAL) = -750.361542553 Eh

	Atom	X	Y	Z
1	C	-0.0908	-0.3985	1.3056
2	C	0.0166	-1.6694	0.6704
3	C	0.5085	-1.8842	-0.6494
4	C	-0.3568	-2.8144	1.4232
5	C	0.5653	-3.1490	-1.1869
6	H	0.9029	-1.0548	-1.2232
7	C	-0.3242	-4.0883	0.8866
8	H	-0.7058	-2.6818	2.4435
9	C	0.1329	-4.2668	-0.4351
10	H	0.9600	-3.3223	-2.1822
11	H	-0.6393	-4.9323	1.4873
12	C	-0.1133	0.8981	0.7826
13	C	0.0164	1.9986	1.6935
14	C	-0.2987	1.2320	-0.5994
15	C	0.0525	3.2980	1.2704
16	H	0.1200	1.7875	2.7542
17	C	-0.2870	2.5273	-1.0356
18	H	-0.5162	0.4493	-1.3155
19	C	-0.0772	3.6167	-0.1225
20	H	-0.4649	2.7255	-2.0842
21	C	-0.1717	-6.6438	-0.3699
22	H	-1.2295	-6.6012	-0.0897
23	H	0.4474	-6.8021	0.5194
24	N	-0.0304	4.8895	-0.5562
25	C	-0.1751	5.2033	-1.9828
26	C	0.1574	5.9956	0.3901
27	H	-1.1571	4.8914	-2.3544
28	H	0.6053	4.7100	-2.5708
29	H	-0.6675	6.0393	1.1090
30	H	1.1016	5.8857	0.9334
31	H	0.1747	4.0859	2.0015
32	H	-0.0105	-7.4537	-1.0805
33	H	0.1848	6.9337	-0.1608
34	H	-0.0816	6.2789	-2.1204
35	H	-0.1739	-0.4507	2.3909
36	O	0.2219	-5.4475	-1.0592

ar2dmnomeobn_MeButa
Energy (POTENTIAL) = -1136.04699029 Eh

	Atom	X	Y	Z
1	C	-0.3539	-0.5079	-1.3480
2	C	-1.4289	-1.4574	-0.8387
3	C	-2.5233	-1.7577	-1.6650
4	C	-1.4292	-1.9785	0.4587
5	C	-3.5711	-2.5524	-1.2188
6	H	-2.5533	-1.3578	-2.6760
7	C	-2.4738	-2.7791	0.9286
8	H	-0.5939	-1.7670	1.1196
9	C	-3.5539	-3.0703	0.0857
10	H	-4.4136	-2.7894	-1.8614
11	H	-2.4330	-3.1645	1.9406
12	C	1.0659	-0.7962	-0.8653
13	C	1.9758	0.2473	-0.6602
14	C	1.5458	-2.1015	-0.7033
15	C	3.2955	0.0101	-0.2866
16	H	1.6393	1.2710	-0.7904
17	C	2.8645	-2.3600	-0.3392
18	H	0.8760	-2.9433	-0.8567
19	C	3.7844	-1.3071	-0.1239
20	H	3.9455	0.8619	-0.1258
21	H	3.1717	-3.3921	-0.2197
22	N	5.1090	-1.5559	0.2180
23	C	5.9331	-0.4503	0.6839
24	C	5.4870	-2.8992	0.6321
25	H	5.5274	0.0326	1.5876
26	H	6.0354	0.3159	-0.0930
27	H	4.9307	-3.2441	1.5186
28	H	5.3179	-3.6200	-0.1760

29	C	-4.6633	-4.3938	1.7429
30	H	-3.8127	-5.0641	1.9213
31	H	-4.6717	-3.6080	2.5091
32	O	-0.7173	0.8710	-1.1307
33	C	-0.9964	1.2306	0.2288
34	H	-1.9893	0.8567	0.5147
35	H	-0.2628	0.7758	0.9070
36	C	-0.9380	2.7322	0.3255
37	C	0.1871	3.3790	0.8479
38	C	-1.9964	3.5156	-0.1508
39	C	0.2498	4.7726	0.8998
40	H	1.0225	2.7866	1.2144
41	C	-1.9314	4.9070	-0.1017
42	H	-2.8749	3.0282	-0.5674
43	C	-0.8084	5.5599	0.4281
44	H	1.1335	5.2555	1.3110
45	H	-2.7636	5.4979	-0.4784
46	C	-0.7568	7.0677	0.5132
47	H	-1.2899	7.5346	-0.3222
48	H	-1.2264	7.4271	1.4387
49	H	0.2749	7.4346	0.5077
50	H	-5.5916	-4.9645	1.8002
51	H	6.9341	-0.8210	0.9117
52	H	6.5528	-2.9150	0.8667
53	H	-0.3554	-0.5718	-2.4439
54	O	-4.6252	-3.8416	0.4304

SP_mln_rad_MeButa
Energy (POTENTIAL) = -263.7424951 Eh

	Atom	X	Y	Z
1	C	-0.0241	0.2966	0.0001
2	C	-1.2008	-0.4692	0.0001
3	N	-2.1952	-1.0875	-0.0000
4	C	1.2058	-0.3875	0.0000
5	C	-0.0610	1.8012	0.0002
6	H	0.4560	2.1912	0.8853
7	H	0.4582	2.1914	-0.8834
8	H	-1.0903	2.1632	-0.0010
9	N	2.2421	-0.9305	0.0001

ar2dmnomeobn_PMMA
Energy (POTENTIAL) = -1136.04582093 Eh

	Atom	X	Y	Z
1	C	-0.3580	-0.5105	-1.3500
2	C	-1.4318	-1.4603	-0.8385
3	C	-2.5299	-1.7571	-1.6612
4	C	-1.4278	-1.9843	0.4576
5	C	-3.5769	-2.5515	-1.2129
6	H	-2.5632	-1.3541	-2.6708
7	C	-2.4717	-2.7846	0.9296
8	H	-0.5896	-1.7752	1.1156
9	C	-3.5552	-3.0725	0.0902
10	H	-4.4225	-2.7859	-1.8523
11	H	-2.4275	-3.1726	1.9405
12	C	1.0624	-0.7962	-0.8674
13	C	1.9696	0.2493	-0.6605
14	C	1.5451	-2.1006	-0.7069
15	C	3.2895	0.0145	-0.2865
16	H	1.6306	1.2721	-0.7904
17	C	2.8641	-2.3564	-0.3423
18	H	0.8772	-2.9436	-0.8619
19	C	3.7811	-1.3017	-0.1252
20	H	3.9377	0.8675	-0.1248
21	H	3.1739	-3.3878	-0.2242
22	N	5.1065	-1.5479	0.2176
23	C	5.9277	-0.4409	0.6840
24	C	5.4869	-2.8904	0.6307
25	H	5.5210	0.0411	1.5879
26	H	6.0285	0.3257	-0.0926
27	H	4.9302	-3.2379	1.5161
28	H	5.3209	-3.6107	-0.1785
29	C	-4.6604	-4.3977	1.7479
30	H	-3.8100	-5.0696	1.9225
31	H	-4.6650	-3.6135	2.5160
32	O	-0.7227	0.8678	-1.1348
33	C	-1.0035	1.2281	0.2238
34	H	-1.9985	0.8577	0.5072
35	H	-0.2734	0.7705	0.9040
36	C	-0.9392	2.7294	0.3215
37	C	0.1801	3.3707	0.8622
38	C	-1.9864	3.5178	-0.1709
39	C	0.2481	4.7639	0.9165
40	H	1.0073	2.7741	1.2405
41	C	-1.9161	4.9087	-0.1194
42	H	-2.8599	3.0345	-0.6024
43	C	-0.7989	5.5562	0.4290
44	H	1.1274	5.2426	1.3420
45	H	-2.7394	5.5036	-0.5090
46	C	-0.7422	7.0637	0.5165
47	H	-1.2595	7.5338	-0.3270
48	H	-1.2248	7.4245	1.4346
49	H	0.2912	7.4262	0.5276
50	H	-5.5892	-4.9674	1.8077
51	H	6.9297	-0.8094	0.9119
52	H	6.5525	-2.9040	0.8669
53	H	-0.3596	-0.5763	-2.4458
54	O	-4.6263	-3.8434	0.4371

SP_ar2dmnome_rad_PMMA
Energy (POTENTIAL) = -750.7019955 Eh

	Atom	X	Y	Z
1	C	-0.1239	-0.4162	1.3594
2	C	-0.0301	-1.6912	0.7078
3	C	0.5040	-1.9027	-0.5949
4	C	-0.4452	-2.8532	1.4082
5	C	0.5737	-3.1668	-1.1550
6	H	0.9109	-1.0657	-1.1503
7	C	-0.3818	-4.1263	0.8526
8	H	-0.8412	-2.7422	2.4151
9	C	0.1218	-4.2934	-0.4466
10	H	0.9943	-3.3149	-2.1454
11	H	-0.7235	-4.9757	1.4332
12	C	-0.0734	0.9043	0.8150
13	C	0.1128	2.0088	1.6916
14	C	-0.2534	1.2386	-0.5548
15	C	0.1709	3.3167	1.2469
16	H	0.2238	1.8155	2.7566
17	C	-0.2017	2.5453	-1.0112
18	H	-0.4926	0.4585	-1.2687
19	C	0.0392	3.6310	-0.1312
20	H	-0.3709	2.7254	-2.0662
21	C	-0.2021	-6.6639	-0.4202

22	H	-1.2698	-6.6145	-0.1694
23	H	0.3763	-6.8350	0.4971
24	N	0.1359	4.9330	-0.5914
25	C	-0.2071	5.2309	-1.9739
26	C	0.1542	6.0297	0.3653
27	H	-1.2575	4.9979	-2.2086
28	H	0.4296	4.6660	-2.6645
29	H	-0.7575	6.0669	0.9817
30	H	1.0166	5.9498	1.0374
31	H	0.3192	4.1039	1.9766
32	H	-0.0317	-7.4894	-1.1136
33	H	0.2430	6.9741	-0.1739
34	H	-0.0400	6.2924	-2.1643
35	H	-0.2553	-0.4634	2.4401
36	O	0.2294	-5.4891	-1.0976

SP_mln_rad_PMMA
Energy (POTENTIAL) = -263.7415101 Eh

	Atom	X	Y	Z
1	C	-0.0242	0.2954	0.0001
2	C	-1.2014	-0.4693	0.0001
3	N	-2.1970	-1.0858	0.0000
4	C	1.2062	-0.3876	0.0000
5	C	-0.0609	1.8004	0.0002
6	H	0.4560	2.1907	0.8852
7	H	0.4583	2.1909	-0.8833
8	H	-1.0902	2.1625	-0.0010
9	N	2.2437	-0.9284	0.0001

ar2dmnomeobn_meoh_MeButa
Energy (POTENTIAL) = -1251.78746449 Eh

	Atom	X	Y	Z
1	C	-0.5295	-0.3891	-1.0843
2	C	-1.9373	-0.5234	-0.5271
3	C	-2.9928	0.1078	-1.2074
4	C	-2.2223	-1.1577	0.6848
5	C	-4.2857	0.0879	-0.7033
6	H	-2.7868	0.6493	-2.1271
7	C	-3.5184	-1.1905	1.2068
8	H	-1.4238	-1.6449	1.2359
9	C	-4.5601	-0.5678	0.5078
10	H	-5.1026	0.5736	-1.2281
11	H	-3.6988	-1.6974	2.1475
12	C	0.4467	-1.5079	-0.7448
13	C	1.7987	-1.2400	-0.5015
14	C	0.0545	-2.8524	-0.7618
15	C	2.7165	-2.2577	-0.2580
16	H	2.1425	-0.2104	-0.4992
17	C	0.9588	-3.8843	-0.5302
18	H	-0.9844	-3.1064	-0.9540
19	C	2.3247	-3.6166	-0.2740
20	H	3.7457	-1.9844	-0.0589
21	H	0.5919	-4.9034	-0.5468
22	N	3.2390	-4.6401	-0.0609
23	C	4.5631	-4.3078	0.4435
24	C	2.7463	-5.9918	0.1606
25	H	4.5303	-3.7948	1.4180
26	H	5.1006	-3.6632	-0.2613
27	H	2.0824	-6.0644	1.0369
28	H	2.1952	-6.3566	-0.7139
29	C	-6.2012	-1.1830	2.1382
30	H	-5.9801	-2.2574	2.1015
31	H	-5.6723	-0.7334	2.9883
32	O	0.0356	0.9100	-0.7596
33	C	0.0396	1.2679	0.6384
34	H	-0.9834	1.5086	0.9527
35	H	0.3863	0.4181	1.2374
36	C	0.9562	2.4501	0.7993
37	C	2.2186	2.3104	1.3834
38	C	0.5693	3.7132	0.3291
39	C	3.0739	3.4079	1.5030
40	H	2.5376	1.3354	1.7451
41	C	1.4284	4.8039	0.4434
42	H	-0.3983	3.8310	-0.1522
43	C	2.6931	4.6724	1.0380
44	H	4.0517	3.2786	1.9617
45	H	1.1139	5.7752	0.0677
46	C	3.6028	5.8687	1.1915
47	H	3.5382	6.5346	0.3239
48	H	3.3287	6.4614	2.0743
49	H	4.6481	5.5659	1.3122
50	C	-0.3724	3.3206	-3.1401
51	H	-0.8567	4.0674	-3.7776
52	H	0.4506	3.8111	-2.5982
53	H	0.0605	2.5442	-3.7901
54	O	-1.3585	2.8009	-2.2588
55	H	-0.9030	2.1401	-1.6953
56	H	-0.6105	-0.3247	-2.1767
57	H	5.1439	-5.2248	0.5571
58	H	3.5944	-6.6609	0.3156
59	H	-7.2755	-1.0388	2.2634
60	O	-5.8615	-0.5394	0.9134

ar2dmn2mln
Energy (POTENTIAL) = -1033.70366104 Eh

	Atom	X	Y	Z
1	C	-1.1934	0.0411	0.4622
2	C	-0.4193	1.3440	0.3327
3	C	-0.0458	2.0210	1.5015
4	C	0.6947	3.1978	1.4743
5	C	1.1094	3.7704	0.2479
6	C	0.7104	3.1010	-0.9340
7	C	-0.0243	1.9201	-0.8839
8	H	-0.3029	1.4615	-1.8269
9	H	0.9707	3.5001	-1.9066
10	H	0.9428	3.6720	2.4160
11	H	-0.3423	1.6192	2.4681
12	C	-2.5252	0.0064	-0.4223
13	C	-2.2347	-0.2004	-1.8555
14	N	-2.0156	-0.3447	-2.9857
15	C	-0.3593	-1.2211	0.2860
16	C	0.5849	-1.3784	-0.7375
17	C	1.3129	-2.5535	-0.8886
18	C	1.1277	-3.6505	-0.0113
19	C	0.1922	-3.4798	1.0373
20	C	-0.5272	-2.2968	1.1684
21	H	-1.2388	-2.2106	1.9864
22	H	0.0237	-4.2687	1.7601
23	H	2.0352	-2.6103	-1.6937
24	H	0.7758	-0.5645	-1.4291
25	N	1.8710	4.9258	0.2064
26	C	2.0746	5.5983	-1.0692

27	C	2.0584	5.6969	1.4275
28	H	1.1291	5.9183	-1.5341
29	H	2.5986	4.9461	-1.7774
30	H	1.1069	6.0509	1.8540
31	H	2.5733	5.1026	2.1912
32	N	1.8273	-4.8339	-0.1730
33	C	2.9300	-4.8866	-1.1224
34	C	1.7725	-5.8420	0.8763
35	H	3.7300	-4.1694	-0.8813
36	H	2.5814	-4.6775	-2.1405
37	H	2.1529	-5.4694	1.8401
38	H	0.7457	-6.1941	1.0294
39	H	2.6819	6.5654	1.2103
40	H	2.6966	6.4809	-0.9127
41	H	-1.5922	0.0205	1.4825
42	H	2.3745	-6.7018	0.5786
43	H	3.3567	-5.8906	-1.1198
44	C	-3.3350	-1.1501	0.0159
45	N	-3.9853	-2.0331	0.3943
46	C	-3.3678	1.2981	-0.2387
47	H	-3.5626	1.4445	0.8269
48	H	-2.8072	2.1541	-0.6167
49	H	-4.3184	1.2216	-0.7715

SP_ar2dmnome_cat
Energy (POTENTIAL) = -750.5615481 Eh

	Atom	X	Y	Z
1	C	-0.0702	-0.3967	1.3012
2	C	0.0286	-1.6703	0.6650
3	C	0.5093	-1.8882	-0.6575
4	C	-0.3433	-2.8123	1.4217
5	C	0.5575	-3.1550	-1.1940
6	H	0.9005	-1.0614	-1.2372
7	C	-0.3186	-4.0879	0.8863
8	H	-0.6841	-2.6779	2.4443
9	C	0.1274	-4.2695	-0.4378
10	H	0.9427	-3.3287	-2.1930
11	H	-0.6316	-4.9295	1.4911
12	C	-0.0994	0.8980	0.7786
13	C	0.0253	1.9985	1.6918
14	C	-0.2858	1.2333	-0.6040
15	C	0.0537	3.2983	1.2716
16	H	0.1311	1.7849	2.7517
17	C	-0.2778	2.5287	-1.0379
18	H	-0.5000	0.4514	-1.3217
19	C	-0.0751	3.6181	-0.1218
20	H	-0.4572	2.7286	-2.0858
21	C	-0.1879	-6.6449	-0.3624
22	H	-1.2424	-6.5949	-0.0716
23	H	0.4380	-6.8030	0.5221
24	N	-0.0343	4.8904	-0.5534
25	C	-0.1527	5.2045	-1.9825
26	C	0.1171	5.9985	0.3973
27	H	-1.1379	4.9181	-2.3658
28	H	0.6213	4.6878	-2.5578
29	H	-0.7081	6.0093	1.1164
30	H	1.0651	5.9190	0.9393
31	H	0.1743	4.0855	2.0036
32	H	-0.0387	-7.4597	-1.0702
33	H	0.1099	6.9387	-0.1505
34	H	-0.0261	6.2766	-2.1203
35	H	-0.1403	-0.4483	2.3873
36	O	0.2067	-5.4543	-1.0613

SP_ar2dmnome_cat_PMMA
Energy (POTENTIAL) = -750.548357 Eh

	Atom	X	Y	Z
1	C	-0.0908	-0.3985	1.3056
2	C	0.0166	-1.6694	0.6704
3	C	0.5085	-1.8842	-0.6494
4	C	-0.3568	-2.8144	1.4232
5	C	0.5653	-3.1490	-1.1869
6	H	0.9029	-1.0548	-1.2232
7	C	-0.3242	-4.0883	0.8866
8	H	-0.7058	-2.6818	2.4435
9	C	0.1329	-4.2668	-0.4351
10	H	0.9600	-3.3223	-2.1822
11	H	-0.6393	-4.9323	1.4873
12	C	-0.1133	0.8981	0.7826
13	C	0.0164	1.9986	1.6935
14	C	-0.2987	1.2320	-0.5994
15	C	0.0525	3.2980	1.2704
16	H	0.1200	1.7875	2.7542
17	C	-0.2870	2.5273	-1.0356
18	H	-0.5162	0.4493	-1.3155
19	C	-0.0772	3.6167	-0.1225
20	H	-0.4649	2.7255	-2.0842
21	C	-0.1717	-6.6438	-0.3699
22	H	-1.2295	-6.6012	-0.0897
23	H	0.4474	-6.8021	0.5194
24	N	-0.0304	4.8895	-0.5562
25	C	-0.1751	5.2033	-1.9828
26	C	0.1574	5.9956	0.3901
27	H	-1.1571	4.8914	-2.3544
28	H	0.6053	4.7100	-2.5708
29	H	-0.6675	6.0393	1.1090
30	H	1.1016	5.8857	0.9334
31	H	0.1747	4.0859	2.0015
32	H	-0.0105	-7.4537	-1.0805
33	H	0.1848	6.9337	-0.1608
34	H	-0.0816	6.2789	-2.1204
35	H	-0.1739	-0.4507	2.3909
36	O	0.2219	-5.4475	-1.0592

SP_ar2dmn2_rad_MeButa
Energy (POTENTIAL) = -770.1544961 Eh

	Atom	X	Y	Z
1	C	-0.0170	-0.0010	1.4740
2	C	0.0372	-1.3022	0.8803
3	C	0.4402	-1.5849	-0.4528
4	C	-0.2870	-2.4395	1.6703
5	C	0.4782	-2.8749	-0.9585
6	H	0.7725	-0.7771	-1.0949
7	C	-0.2570	-3.7310	1.1752
8	H	-0.5894	-2.2855	2.7042
9	C	0.1260	-3.9956	-0.1653
10	H	0.8023	-3.0125	-1.9834
11	H	-0.5355	-4.5439	1.8355
12	C	-0.0155	1.2958	0.8685
13	C	0.2330	2.4392	1.6767
14	C	-0.3031	1.5694	-0.4960
15	C	0.2486	3.7270	1.1709

16	H	0.4271	2.2946	2.7376
17	C	-0.2940	2.8555	-1.0129
18	H	-0.5933	0.7587	-1.1547
19	C	0.0092	3.9798	-0.2048
20	H	-0.5456	2.9870	-2.0588
21	C	-0.3958	-6.3760	0.1109
22	C	0.3644	-5.4872	-2.0960
23	H	-1.4700	-6.2351	0.3086
24	H	0.1157	-6.4814	1.0748
25	H	-0.4300	-5.0161	-2.6959
26	H	1.3267	-5.0789	-2.4268
27	N	0.1762	-5.2870	-0.6669
28	N	0.0673	5.2623	-0.7301
29	C	-0.4079	5.4970	-2.0856
30	C	0.1230	6.3994	0.1770
31	H	-1.4760	5.2576	-2.2088
32	H	0.1604	4.8984	-2.8065
33	H	-0.7536	6.4546	0.8416
34	H	1.0219	6.3573	0.8026
35	H	0.4486	4.5461	1.8516
36	H	-0.2665	-7.3125	-0.4342
37	H	0.3695	-6.5569	-2.3110
38	H	0.1711	7.3204	-0.4064
39	H	-0.2591	6.5476	-2.3405
40	H	-0.0663	0.0028	2.5629

ar2dmnome_cat
Energy (POTENTIAL) = -750.374299227 Eh

	Atom	X	Y	Z
1	C	-0.0702	-0.3967	1.3012
2	C	0.0286	-1.6703	0.6650
3	C	0.5093	-1.8882	-0.6575
4	C	-0.3433	-2.8123	1.4217
5	C	0.5575	-3.1550	-1.1940
6	H	0.9005	-1.0614	-1.2372
7	C	-0.3186	-4.0879	0.8863
8	H	-0.6841	-2.6779	2.4443
9	C	0.1274	-4.2695	-0.4378
10	H	0.9427	-3.3287	-2.1930
11	H	-0.6316	-4.9295	1.4911
12	C	-0.0993	0.8980	0.7786
13	C	0.0253	1.9985	1.6918
14	C	-0.2858	1.2333	-0.6040
15	C	0.0537	3.2983	1.2716
16	H	0.1311	1.7849	2.7517
17	C	-0.2778	2.5287	-1.0379
18	H	-0.5000	0.4514	-1.3217
19	C	-0.0751	3.6181	-0.1218
20	H	-0.4572	2.7286	-2.0858
21	C	-0.1879	-6.6449	-0.3624
22	H	-1.2424	-6.5949	-0.0716
23	H	0.4380	-6.8030	0.5221
24	N	-0.0343	4.8904	-0.5534
25	C	-0.1527	5.2045	-1.9825
26	C	0.1171	5.9985	0.3973
27	H	-1.1379	4.9181	-2.3658
28	H	0.6213	4.6878	-2.5578
29	H	-0.7081	6.0093	1.1164
30	H	1.0651	5.9190	0.9393
31	H	0.1743	4.0855	2.0036
32	H	-0.0387	-7.4597	-1.0702
33	H	0.1099	6.9387	-0.1505
34	H	-0.0261	6.2766	-2.1203
35	H	-0.1403	-0.4483	2.3873
36	O	0.2067	-5.4543	-1.0613

SP_ar2dmn2obn
Energy (POTENTIAL) = -1155.7989505 Eh

	Atom	X	Y	Z
1	C	-0.3264	-0.1439	-1.3111
2	C	-1.7533	-0.0854	-0.7947
3	C	-2.7765	0.3808	-1.6291
4	C	-2.1045	-0.4042	0.5244
5	C	-4.0870	0.5243	-1.1847
6	H	-2.5463	0.6360	-2.6618
7	C	-3.4067	-0.2659	0.9932
8	H	-1.3451	-0.7853	1.2016
9	C	-4.4397	0.2160	0.1520
10	H	-4.8360	0.8745	-1.8846
11	H	-3.6168	-0.5379	2.0205
12	C	0.4999	-1.3378	-0.8356
13	C	1.8899	-1.2347	-0.7001
14	C	-0.0688	-2.5969	-0.6082
15	C	2.6778	-2.3217	-0.3326
16	H	2.3655	-0.2753	-0.8773
17	C	0.7024	-3.6994	-0.2484
18	H	-1.1430	-2.7260	-0.7054
19	C	2.1059	-3.5957	-0.1033
20	H	3.7454	-2.1706	-0.2260
21	H	0.1999	-4.6436	-0.0760
22	N	2.8866	-4.6947	0.2350
23	C	4.2728	-4.4780	0.6246
24	C	2.2299	-5.8996	0.7225
25	H	4.3701	-3.8211	1.5038
26	H	4.8450	-4.0321	-0.1968
27	H	1.6343	-5.7213	1.6321
28	H	1.5653	-6.3198	-0.0409
29	N	-5.7339	0.3830	0.6177
30	C	-6.8007	0.6622	-0.3330
31	C	-6.0981	-0.1639	1.9172
32	H	-6.9176	-0.1345	-1.0842
33	H	-6.6174	1.6042	-0.8628
34	H	-5.9548	-1.2544	1.9722
35	H	-5.5069	0.2968	2.7171
36	O	0.3800	1.1010	-1.0984
37	C	0.4580	1.5411	0.2636
38	H	-0.5125	1.9471	0.5801
39	H	0.6972	0.6992	0.9264
40	C	1.5333	2.5924	0.3435
41	C	2.8342	2.2629	0.7409
42	C	1.2651	3.9159	-0.0272
43	C	3.8386	3.2316	0.7717
44	H	3.0626	1.2384	1.0263
45	C	2.2696	4.8827	0.0017
46	H	0.2607	4.1884	-0.3433
47	C	3.5734	4.5580	0.4049
48	H	4.8427	2.9542	1.0848
49	H	2.0404	5.9050	-0.2910
50	C	4.6506	5.6155	0.4698
51	H	4.5275	6.3620	-0.3225
52	H	4.6190	6.1519	1.4275
53	H	5.6496	5.1772	0.3746

54	H	-7.7432	0.7664	0.2067
55	H	-7.1483	0.0549	2.1162
56	H	4.7326	-5.4393	0.8604
57	H	2.9865	-6.6528	0.9489
58	H	-0.3769	-0.1853	-2.4070

mln_an_MeButa
Energy (POTENTIAL) = -263.810007571 Eh

	Atom	X	Y	Z
1	C	-0.0390	0.2654	-0.0259
2	C	-1.1729	-0.5405	0.0748
3	N	-2.1470	-1.2028	0.1521
4	C	1.2189	-0.3367	-0.0797
5	C	-0.1582	1.7819	0.0003
6	H	0.0947	2.2278	0.9762
7	H	0.4973	2.2531	-0.7451
8	H	-1.1848	2.0860	-0.2350
9	N	2.2965	-0.8144	-0.1335

SP_ar2dmnomeobn_meoh_PMMA
Energy (POTENTIAL) = -1252.1275676 Eh

	Atom	X	Y	Z
1	C	-0.5275	-0.3904	-1.0824
2	C	-1.9346	-0.5224	-0.5225
3	C	-2.9880	0.1221	-1.1937
4	C	-2.2201	-1.1666	0.6839
5	C	-4.2795	0.1045	-0.6865
6	H	-2.7808	0.6727	-2.1078
7	C	-3.5151	-1.1973	1.2086
8	H	-1.4227	-1.6632	1.2283
9	C	-4.5548	-0.5620	0.5184
10	H	-5.0949	0.6008	-1.2037
11	H	-3.6962	-1.7125	2.1447
12	C	0.4478	-1.5106	-0.7449
13	C	1.7987	-1.2438	-0.4955
14	C	0.0554	-2.8549	-0.7696
15	C	2.7153	-2.2629	-0.2530
16	H	2.1427	-0.2143	-0.4888
17	C	0.9587	-3.8878	-0.5390
18	H	-0.9828	-3.1076	-0.9671
19	C	2.3233	-3.6213	-0.2763
20	H	3.7440	-1.9907	-0.0496
21	H	0.5918	-4.9068	-0.5619
22	N	3.2369	-4.6462	-0.0638
23	C	4.5585	-4.3162	0.4475
24	C	2.7431	-5.9984	0.1494
25	H	4.5220	-3.8070	1.4240
26	H	5.0992	-3.6689	-0.2526
27	H	2.0750	-6.0751	1.0224
28	H	2.1960	-6.3592	-0.7292
29	C	-6.1954	-1.1828	2.1462
30	H	-5.9810	-2.2584	2.0995
31	H	-5.6618	-0.7444	2.9994
32	O	0.0403	0.9074	-0.7586
33	C	0.0399	1.2685	0.6382
34	H	-0.9845	1.5078	0.9495
35	H	0.3868	0.4208	1.2401
36	C	0.9533	2.4532	0.7986
37	C	2.2121	2.3191	1.3913
38	C	0.5664	3.7132	0.3201
39	C	3.0641	3.4190	1.5116
40	H	2.5309	1.3463	1.7593
41	C	1.4222	4.8062	0.4351
42	H	-0.3982	3.8265	-0.1683
43	C	2.6832	4.6804	1.0385
44	H	4.0392	3.2942	1.9771
45	H	1.1079	5.7751	0.0530
46	C	3.5890	5.8796	1.1927
47	H	3.5286	6.5415	0.3216
48	H	3.3079	6.4757	2.0711
49	H	4.6343	5.5801	1.3213
50	C	-0.3597	3.3146	-3.1540
51	H	-0.8474	4.0562	-3.7950
52	H	0.4594	3.8118	-2.6122
53	H	0.0796	2.5388	-3.8008
54	O	-1.3442	2.7921	-2.2734
55	H	-0.8866	2.1367	-1.7055
56	H	-0.6104	-0.3260	-2.1747
57	H	5.1389	-5.2337	0.5597
58	H	3.5903	-6.6686	0.3053
59	H	-7.2686	-1.0335	2.2757
60	O	-5.8553	-0.5303	0.9272

SP_mln_an_MeButa
Energy (POTENTIAL) = -263.9017281 Eh

	Atom	X	Y	Z
1	C	-0.0390	0.2654	-0.0259
2	C	-1.1729	-0.5405	0.0748
3	N	-2.1470	-1.2028	0.1521
4	C	1.2189	-0.3367	-0.0797
5	C	-0.1582	1.7819	0.0003
6	H	0.0947	2.2278	0.9762
7	H	0.4973	2.2531	-0.7451
8	H	-1.1848	2.0860	-0.2350
9	N	2.2965	-0.8144	-0.1335

ar2dmn2mln_MeButa
Energy (POTENTIAL) = -1033.70030856 Eh

	Atom	X	Y	Z
1	C	-1.1928	0.0376	0.4667
2	C	-0.4217	1.3423	0.3367
3	C	-0.0408	2.0161	1.5044
4	C	0.6972	3.1943	1.4755
5	C	1.1015	3.7711	0.2482
6	C	0.6958	3.1047	-0.9325
7	C	-0.0361	1.9224	-0.8809
8	H	-0.3197	1.4646	-1.8229
9	H	0.9495	3.5067	-1.9058
10	H	0.9522	3.6658	2.4168
11	H	-0.3281	1.6097	2.4719
12	C	-2.5244	-0.0004	-0.4178
13	C	-2.2333	-0.2108	-1.8503
14	N	-2.0139	-0.3514	-2.9808
15	C	-0.3555	-1.2223	0.2895
16	C	0.6039	-1.3668	-0.7211
17	C	1.3342	-2.5397	-0.8748
18	C	1.1353	-3.6468	-0.0145
19	C	0.1840	-3.4896	1.0211
20	C	-0.5373	-2.3084	1.1558
21	H	-1.2628	-2.2332	1.9625
22	H	0.0031	-4.2879	1.7303
23	H	2.0681	-2.5872	-1.6701

24	H	0.8047	-0.5444	-1.3996
25	N	1.8601	4.9291	0.2047
26	C	2.0575	5.6029	-1.0705
27	C	2.0580	5.6947	1.4265
28	H	1.1097	5.9210	-1.5324
29	H	2.5809	4.9525	-1.7810
30	H	1.1103	6.0452	1.8646
31	H	2.5820	5.0982	2.1825
32	N	1.8384	-4.8286	-0.1796
33	C	2.9534	-4.8701	-1.1136
34	C	1.7645	-5.8507	0.8536
35	H	3.7505	-4.1560	-0.8534
36	H	2.6189	-4.6490	-2.1340
37	H	2.1267	-5.4922	1.8300
38	H	0.7350	-6.2052	0.9825
39	H	2.6777	6.5657	1.2074
40	H	2.6779	6.4870	-0.9157
41	H	-1.5913	0.0160	1.4874
42	H	2.3721	-6.7066	0.5553
43	H	3.3801	-5.8744	-1.1175
44	C	-3.3380	-1.1511	0.0282
45	N	-3.9944	-2.0245	0.4183
46	C	-3.3657	1.2931	-0.2402
47	H	-3.5597	1.4458	0.8248
48	H	-2.8059	2.1475	-0.6230
49	H	-4.3173	1.2137	-0.7711

refdiarcn_rad
Energy (POTENTIAL) = -823.315318386 Eh

	Atom	X	Y	Z
1	C	-0.0235	-0.0204	1.2104
2	C	0.0380	-1.3139	0.5459
3	C	0.6099	-1.4701	-0.7432
4	C	-0.4298	-2.4829	1.1887
5	C	0.6798	-2.7092	-1.3532
6	H	1.0347	-0.6106	-1.2498
7	C	-0.3658	-3.7321	0.5830
8	H	-0.8668	-2.4069	2.1800
9	C	0.1857	-3.8550	-0.7028
10	H	1.1307	-2.8234	-2.3339
11	H	-0.7486	-4.5965	1.1122
12	C	-0.0544	1.2738	0.5449
13	C	0.3839	2.4421	1.2092
14	C	-0.5672	1.4313	-0.7687
15	C	0.3479	3.6918	0.6025
16	H	0.7753	2.3651	2.2194
17	C	-0.6089	2.6710	-1.3800
18	H	-0.9693	0.5727	-1.2948
19	C	-0.1446	3.8162	-0.7070
20	H	-1.0150	2.7860	-2.3801
21	C	-0.1714	-6.2220	-0.7789
22	H	-1.2461	-6.1677	-0.5683
23	H	0.3736	-6.4341	0.1487
24	H	0.7066	4.5555	1.1493
25	H	0.0183	-7.0156	-1.5025
26	O	0.2976	-5.0187	-1.3910
27	O	-0.2249	4.9804	-1.3984
28	C	0.2155	6.1834	-0.7648
29	H	0.0582	6.9774	-1.4956
30	H	1.2797	6.1294	-0.5062
31	H	-0.3707	6.3944	0.1375
32	C	-0.0561	-0.0209	2.6238
33	N	-0.0826	-0.0214	3.7948

mln_an_PMMA
Energy (POTENTIAL) = -263.803164863 Eh

	Atom	X	Y	Z
1	C	-0.0390	0.2638	-0.0253
2	C	-1.1748	-0.5406	0.0715
3	N	-2.1512	-1.1997	0.1455
4	C	1.2203	-0.3365	-0.0762
5	C	-0.1576	1.7799	0.0002
6	H	0.1067	2.2279	0.9726
7	H	0.4888	2.2515	-0.7534
8	H	-1.1873	2.0834	-0.2229
9	N	2.2997	-0.8100	-0.1275

SP_ar2dmn2obn_meoh
Energy (POTENTIAL) = -1271.5835278 Eh

	Atom	X	Y	Z
1	C	-0.2986	-0.3234	-1.1012
2	C	-1.7099	-0.3759	-0.5484
3	C	-2.7327	0.3099	-1.2177
4	C	-2.0402	-0.9975	0.6627
5	C	-4.0283	0.3678	-0.7176
6	H	-2.5040	0.8339	-2.1429
7	C	-3.3303	-0.9537	1.1810
8	H	-1.2759	-1.5399	1.2120
9	C	-4.3660	-0.2611	0.5068
10	H	-4.7791	0.9045	-1.2847
11	H	-3.5283	-1.4634	2.1161
12	C	0.5924	-1.5192	-0.7894
13	C	1.9576	-1.3642	-0.5231
14	C	0.0984	-2.8288	-0.8495
15	C	2.7917	-2.4570	-0.3016
16	H	2.3805	-0.3654	-0.4816
17	C	0.9178	-3.9342	-0.6417
18	H	-0.9556	-2.9945	-1.0553
19	C	2.2976	-3.7810	-0.3649
20	H	3.8358	-2.2695	-0.0815
21	H	0.4726	-4.9206	-0.6915
22	N	3.1284	-4.8784	-0.1776
23	C	4.4626	-4.6653	0.3658
24	C	2.5285	-6.1935	-0.0001
25	H	4.4455	-4.1866	1.3580
26	H	5.0625	-4.0377	-0.3031
27	H	1.8573	-6.2407	0.8722
28	H	1.9534	-6.4839	-0.8867
29	N	-5.6473	-0.1930	1.0248
30	C	-6.7221	0.3477	0.2046
31	C	-5.9996	-1.0198	2.1703
32	H	-6.8688	-0.2213	-0.7265
33	H	-6.5254	1.3925	-0.0635
34	H	-5.8771	-2.0950	1.9675
35	H	-5.3854	-0.7669	3.0427
36	O	0.3648	0.9238	-0.7440
37	C	0.3841	1.2486	0.6617
38	H	-0.6188	1.5665	0.9728
39	H	0.6517	0.3602	1.2445
40	C	1.3950	2.3457	0.8579
41	C	2.6351	2.0868	1.4494
42	C	1.1218	3.6477	0.4138

43	C	3.5793	3.1051	1.6016
44	H	2.8666	1.0807	1.7919
45	C	2.0687	4.6590	0.5603
46	H	0.1722	3.8578	-0.0719
47	C	3.3120	4.4075	1.1624
48	H	4.5373	2.8828	2.0659
49	H	1.8415	5.6617	0.2046
50	C	4.3178	5.5193	1.3490
51	H	4.3395	6.1882	0.4814
52	H	4.0671	6.1342	2.2235
53	H	5.3277	5.1259	1.5031
54	C	0.1332	3.3827	-3.0552
55	H	-0.2759	4.1818	-3.6819
56	H	1.0000	3.7835	-2.5081
57	H	0.4876	2.5766	-3.7162
58	O	-0.8986	2.9485	-2.1788
59	H	-0.5089	2.2324	-1.6327
60	H	-0.3704	-0.2239	-2.1918
61	H	4.9700	-5.6271	0.4562
62	H	3.3200	-6.9327	0.1338
63	H	-7.6531	0.3225	0.7725
64	H	-7.0416	-0.8374	2.4368

SP_ar2dmn2obn_PMMA
Energy (POTENTIAL) = -1155.7944329 Eh

	Atom	X	Y	Z
1	C	-0.3306	-0.1463	-1.3179
2	C	-1.7574	-0.0890	-0.7994
3	C	-2.7773	0.3960	-1.6263
4	C	-2.1097	-0.4231	0.5150
5	C	-4.0864	0.5419	-1.1791
6	H	-2.5447	0.6660	-2.6545
7	C	-3.4109	-0.2831	0.9860
8	H	-1.3520	-0.8178	1.1862
9	C	-4.4400	0.2168	0.1523
10	H	-4.8332	0.9081	-1.8733
11	H	-3.6224	-0.5674	2.0097
12	C	0.4985	-1.3380	-0.8419
13	C	1.8874	-1.2279	-0.7035
14	C	-0.0652	-2.5992	-0.6166
15	C	2.6786	-2.3114	-0.3334
16	H	2.3577	-0.2662	-0.8824
17	C	0.7101	-3.6976	-0.2538
18	H	-1.1386	-2.7328	-0.7175
19	C	2.1117	-3.5867	-0.1046
20	H	3.7456	-2.1562	-0.2258
21	H	0.2118	-4.6444	-0.0833
22	N	2.8970	-4.6832	0.2387
23	C	4.2795	-4.4584	0.6329
24	C	2.2436	-5.8861	0.7320
25	H	4.3714	-3.8004	1.5124
26	H	4.8516	-4.0098	-0.1871
27	H	1.6438	-5.7049	1.6388
28	H	1.5832	-6.3139	-0.0310
29	N	-5.7349	0.3853	0.6212
30	C	-6.7985	0.6836	-0.3256
31	C	-6.1023	-0.1829	1.9092
32	H	-6.9198	-0.1008	-1.0895
33	H	-6.6098	1.6327	-0.8408
34	H	-5.9644	-1.2752	1.9459
35	H	-5.5097	0.2610	2.7179

36	O	0.3723	1.0986	-1.1068
37	C	0.4444	1.5414	0.2533
38	H	-0.5261	1.9531	0.5631
39	H	0.6755	0.7004	0.9206
40	C	1.5248	2.5870	0.3377
41	C	2.8155	2.2564	0.7646
42	C	1.2712	3.9062	-0.0567
43	C	3.8243	3.2200	0.8013
44	H	3.0324	1.2341	1.0667
45	C	2.2800	4.8675	-0.0221
46	H	0.2750	4.1777	-0.3983
47	C	3.5737	4.5418	0.4110
48	H	4.8210	2.9417	1.1371
49	H	2.0627	5.8867	-0.3346
50	C	4.6551	5.5947	0.4816
51	H	4.5542	6.3279	-0.3262
52	H	4.6065	6.1481	1.4290
53	H	5.6536	5.1499	0.4149
54	H	-7.7413	0.7853	0.2147
55	H	-7.1517	0.0376	2.1122
56	H	4.7450	-5.4169	0.8700
57	H	3.0019	-6.6357	0.9655
58	H	-0.3820	-0.1897	-2.4137

mln_rad_MeButa
Energy (POTENTIAL) = -263.668125113 Eh

	Atom	X	Y	Z
1	C	-0.0241	0.2966	0.0001
2	C	-1.2008	-0.4692	0.0001
3	N	-2.1952	-1.0875	-0.0000
4	C	1.2058	-0.3875	0.0000
5	C	-0.0610	1.8012	0.0002
6	H	0.4560	2.1912	0.8853
7	H	0.4582	2.1914	-0.8834
8	H	-1.0903	2.1632	-0.0010
9	N	2.2421	-0.9305	0.0001

mln_rad_PMMA
Energy (POTENTIAL) = -263.667261988 Eh

	Atom	X	Y	Z
1	C	-0.0242	0.2954	0.0001
2	C	-1.2014	-0.4693	0.0001
3	N	-2.1970	-1.0858	0.0000
4	C	1.2062	-0.3876	0.0000
5	C	-0.0609	1.8004	0.0002
6	H	0.4560	2.1907	0.8852
7	H	0.4583	2.1909	-0.8833
8	H	-1.0902	2.1625	-0.0010
9	N	2.2437	-0.9284	0.0001

SP_ar2dmn2obn_MeButa
Energy (POTENTIAL) = -1155.7958266 Eh

	Atom	X	Y	Z
1	C	-0.3296	-0.1462	-1.3159
2	C	-1.7565	-0.0875	-0.7983
3	C	-2.7771	0.3912	-1.6281
4	C	-2.1087	-0.4156	0.5178
5	C	-4.0866	0.5372	-1.1821
6	H	-2.5450	0.6557	-2.6579
7	C	-3.4103	-0.2752	0.9878
8	H	-1.3508	-0.8058	1.1914

9	C	-4.4403	0.2187	0.1512
10	H	-4.8339	0.8981	-1.8786
11	H	-3.6216	-0.5547	2.0129
12	C	0.4981	-1.3386	-0.8393
13	C	1.8875	-1.2313	-0.7028
14	C	-0.0677	-2.5986	-0.6115
15	C	2.6773	-2.3159	-0.3328
16	H	2.3598	-0.2707	-0.8826
17	C	0.7060	-3.6983	-0.2489
18	H	-1.1415	-2.7303	-0.7105
19	C	2.1083	-3.5902	-0.1020
20	H	3.7447	-2.1625	-0.2265
21	H	0.2060	-4.6438	-0.0765
22	N	2.8919	-4.6875	0.2405
23	C	4.2759	-4.4655	0.6320
24	C	2.2371	-5.8902	0.7337
25	H	4.3706	-3.8067	1.5104
26	H	4.8478	-4.0193	-0.1895
27	H	1.6391	-5.7085	1.6415
28	H	1.5748	-6.3162	-0.0286
29	N	-5.7350	0.3877	0.6186
30	C	-6.7994	0.6791	-0.3299
31	C	-6.1018	-0.1723	1.9109
32	H	-6.9192	-0.1100	-1.0890
33	H	-6.6122	1.6255	-0.8507
34	H	-5.9630	-1.2641	1.9546
35	H	-5.5093	0.2774	2.7164
36	O	0.3751	1.0985	-1.1049
37	C	0.4493	1.5410	0.2555
38	H	-0.5209	1.9518	0.5672
39	H	0.6824	0.6999	0.9218
40	C	1.5289	2.5877	0.3382
41	C	2.8225	2.2565	0.7561
42	C	1.2716	3.9083	-0.0491
43	C	3.8306	3.2211	0.7909
44	H	3.0423	1.2335	1.0532
45	C	2.2797	4.8706	-0.0164
46	H	0.2729	4.1808	-0.3829
47	C	3.5763	4.5445	0.4076
48	H	4.8295	2.9424	1.1197
49	H	2.0593	5.8910	-0.3229
50	C	4.6572	5.5980	0.4765
51	H	4.5486	6.3368	-0.3253
52	H	4.6156	6.1447	1.4281
53	H	5.6555	5.1547	0.3987
54	H	-7.7422	0.7823	0.2099
55	H	-7.1512	0.0488	2.1125
56	H	4.7392	-5.4249	0.8697
57	H	2.9947	-6.6409	0.9656
58	H	-0.3806	-0.1896	-2.4117

UVar2dmnome_rad_MeButa
Energy (POTENTIAL) = -750.2720755 Eh

	Atom	X	Y	Z
1	C	-0.1234	-0.4163	1.3558
2	C	-0.0304	-1.6917	0.7047
3	C	0.4985	-1.9045	-0.6001
4	C	-0.4411	-2.8537	1.4083
5	C	0.5674	-3.1694	-1.1588
6	H	0.9019	-1.0682	-1.1589
7	C	-0.3785	-4.1275	0.8542
8	H	-0.8330	-2.7420	2.4167
9	C	0.1198	-4.2958	-0.4470
10	H	0.9838	-3.3178	-2.1509
11	H	-0.7168	-4.9764	1.4374
12	C	-0.0741	0.9044	0.8122
13	C	0.1090	2.0085	1.6904
14	C	-0.2519	1.2403	-0.5579
15	C	0.1664	3.3170	1.2475
16	H	0.2180	1.8142	2.7554
17	C	-0.2009	2.5476	-1.0126
18	H	-0.4884	0.4612	-1.2736
19	C	0.0370	3.6329	-0.1308
20	H	-0.3681	2.7288	-2.0678
21	C	-0.2009	-6.6673	-0.4154
22	H	-1.2674	-6.6188	-0.1603
23	H	0.3818	-6.8358	0.4995
24	N	0.1329	4.9349	-0.5890
25	C	-0.2023	5.2342	-1.9734
26	C	0.1531	6.0311	0.3686
27	H	-1.2509	5.0000	-2.2140
28	H	0.4390	4.6710	-2.6612
29	H	-0.7580	6.0679	0.9856
30	H	1.0160	5.9503	1.0398
31	H	0.3121	4.1035	1.9783
32	H	-0.0322	-7.4934	-1.1084
33	H	0.2415	6.9757	-0.1701
34	H	-0.0358	6.2961	-2.1612
35	H	-0.2534	-0.4637	2.4367
36	O	0.2261	-5.4921	-1.0967

SP_ar2dmnomemln
Energy (POTENTIAL) = -1014.5224769 Eh

	Atom	X	Y	Z
1	C	-0.9991	-0.3808	0.4364
2	C	-0.4751	1.0384	0.2794
3	C	-0.1249	1.7539	1.4323
4	C	0.3971	3.0416	1.3773
5	C	0.5986	3.6944	0.1373
6	C	0.2215	2.9814	-1.0264
7	C	-0.2912	1.6903	-0.9494
8	H	-0.5661	1.2036	-1.8794
9	H	0.3244	3.4328	-2.0054
10	H	0.6404	3.5392	2.3081
11	H	-0.2632	1.2935	2.4083
12	C	-2.3733	-0.6400	-0.3371
13	C	-2.1747	-0.7743	-1.7946
14	N	-2.0292	-0.8654	-2.9420
15	C	0.0263	-1.4752	0.1602
16	C	0.8842	-1.4470	-0.9521
17	C	1.7922	-2.4716	-1.1839
18	C	1.8749	-3.5606	-0.3015
19	C	1.0353	-3.6021	0.8181
20	C	0.1259	-2.5634	1.0332
21	H	-0.5197	-2.6104	1.9064
22	H	1.0791	-4.4227	1.5241
23	H	2.4548	-2.4469	-2.0436
24	H	0.8553	-0.6106	-1.6414
25	N	1.1406	4.9652	0.0645
26	C	1.1195	5.6770	-1.2059
27	C	1.3091	5.7391	1.2865
28	H	0.0978	5.8297	-1.5868

29	H	1.6898	5.1348	-1.9692
30	H	0.3557	5.9192	1.8071
31	H	1.9862	5.2306	1.9826
32	C	2.9249	-5.6420	0.2427
33	H	3.2222	-5.3439	1.2559
34	H	1.9888	-6.2121	0.2922
35	H	1.7537	6.7043	1.0400
36	H	1.5875	6.6539	-1.0767
37	H	-1.2990	-0.4837	1.4849
38	H	3.7061	-6.2647	-0.1957
39	C	-2.9277	-1.9269	0.1346
40	N	-3.3748	-2.9173	0.5407
41	C	-3.4075	0.4823	-0.0499
42	H	-3.5305	0.5830	1.0316
43	H	-3.0400	1.4265	-0.4536
44	H	-4.3733	0.2448	-0.5017
45	O	2.7997	-4.5106	-0.6171

SP_ar2dmnomeobn_meoh_MeButa
Energy (POTENTIAL) = -1252.1290391 Eh

	Atom	X	Y	Z
1	C	-0.5295	-0.3891	-1.0843
2	C	-1.9373	-0.5234	-0.5271
3	C	-2.9928	0.1078	-1.2074
4	C	-2.2223	-1.1577	0.6848
5	C	-4.2857	0.0879	-0.7033
6	H	-2.7868	0.6493	-2.1271
7	C	-3.5184	-1.1905	1.2068
8	H	-1.4238	-1.6449	1.2359
9	C	-4.5601	-0.5678	0.5078
10	H	-5.1026	0.5736	-1.2281
11	H	-3.6988	-1.6974	2.1475
12	C	0.4467	-1.5079	-0.7448
13	C	1.7987	-1.2400	-0.5015
14	C	0.0545	-2.8524	-0.7618
15	C	2.7165	-2.2577	-0.2580
16	H	2.1425	-0.2104	-0.4992
17	C	0.9588	-3.8843	-0.5302
18	H	-0.9844	-3.1064	-0.9540
19	C	2.3247	-3.6166	-0.2740
20	H	3.7457	-1.9844	-0.0589
21	H	0.5919	-4.9034	-0.5468
22	N	3.2390	-4.6401	-0.0609
23	C	4.5631	-4.3078	0.4435
24	C	2.7463	-5.9918	0.1606
25	H	4.5303	-3.7948	1.4180
26	H	5.1006	-3.6632	-0.2613
27	H	2.0824	-6.0644	1.0369
28	H	2.1952	-6.3566	-0.7139
29	C	-6.2012	-1.1830	2.1382
30	H	-5.9801	-2.2574	2.1015
31	H	-5.6723	-0.7334	2.9883
32	O	0.0356	0.9100	-0.7596
33	C	0.0396	1.2679	0.6384
34	H	-0.9834	1.5086	0.9527
35	H	0.3863	0.4181	1.2374
36	C	0.9562	2.4501	0.7993
37	C	2.2186	2.3104	1.3834
38	C	0.5693	3.7132	0.3291
39	C	3.0739	3.4079	1.5030
40	H	2.5376	1.3354	1.7451
41	C	1.4284	4.8039	0.4434
42	H	-0.3983	3.8310	-0.1522
43	C	2.6931	4.6724	1.0380
44	H	4.0517	3.2786	1.9617
45	H	1.1139	5.7752	0.0677
46	C	3.6028	5.8687	1.1915
47	H	3.5382	6.5346	0.3239
48	H	3.3287	6.4614	2.0743
49	H	4.6481	5.5659	1.3122
50	C	-0.3724	3.3206	-3.1401
51	H	-0.8567	4.0674	-3.7776
52	H	0.4506	3.8111	-2.5982
53	H	0.0605	2.5442	-3.7901
54	O	-1.3585	2.8009	-2.2588
55	H	-0.9030	2.1401	-1.6953
56	H	-0.6105	-0.3247	-2.1767
57	H	5.1439	-5.2248	0.5571
58	H	3.5944	-6.6609	0.3156
59	H	-7.2755	-1.0388	2.2634
60	O	-5.8615	-0.5394	0.9134

UVar2dmn2_rad_MeButa_cam
Energy (POTENTIAL) = -769.6944631 Eh

	Atom	X	Y	Z
1	C	0.0045	0.0011	1.5223
2	C	0.0396	-1.3006	0.9281
3	C	0.4166	-1.5861	-0.4120
4	C	-0.2775	-2.4356	1.7242
5	C	0.4371	-2.8764	-0.9179
6	H	0.7423	-0.7806	-1.0603
7	C	-0.2648	-3.7274	1.2291
8	H	-0.5601	-2.2795	2.7634
9	C	0.0922	-3.9947	-0.1181
10	H	0.7417	-3.0163	-1.9485
11	H	-0.5364	-4.5383	1.8946
12	C	0.0035	1.2978	0.9164
13	C	0.2739	2.4397	1.7196
14	C	-0.3072	1.5729	-0.4427
15	C	0.2885	3.7272	1.2132
16	H	0.4863	2.2940	2.7769
17	C	-0.2992	2.8588	-0.9602
18	H	-0.6144	0.7640	-1.0958
19	C	0.0259	3.9813	-0.1580
20	H	-0.5689	2.9917	-2.0013
21	C	-0.4398	-6.3716	0.1683
22	C	0.2859	-5.4882	-2.0523
23	H	-1.5093	-6.2238	0.3855
24	H	0.0884	-6.4801	1.1228
25	H	-0.5162	-5.0121	-2.6378
26	H	1.2446	-5.0862	-2.4006
27	N	0.1249	-5.2865	-0.6201
28	N	0.0826	5.2633	-0.6847
29	C	-0.4155	5.5007	-2.0314
30	C	0.1621	6.4002	0.2208
31	H	-1.4873	5.2682	-2.1351
32	H	0.1356	4.8983	-2.7623
33	H	-0.7019	6.4612	0.9013
34	H	1.0719	6.3524	0.8300
35	H	0.5062	4.5452	1.8899
36	H	-0.3265	-7.3091	-0.3787
37	H	0.2802	-6.5579	-2.2670

38	H	0.2056	7.3208	-0.3636
39	H	-0.2647	6.5504	-2.2893
40	H	-0.0249	0.0054	2.6118

ar2dmn2_rad_PMMA
Energy (POTENTIAL) = -769.954579096 Eh

	Atom	X	Y	Z
1	C	-0.0424	0.0014	1.4790
2	C	0.0193	-1.3004	0.8875
3	C	0.4498	-1.5843	-0.4365
4	C	-0.3259	-2.4360	1.6704
5	C	0.4941	-2.8745	-0.9410
6	H	0.7984	-0.7772	-1.0708
7	C	-0.2901	-3.7276	1.1759
8	H	-0.6499	-2.2807	2.6976
9	C	0.1210	-3.9934	-0.1556
10	H	0.8397	-3.0133	-1.9588
11	H	-0.5855	-4.5395	1.8301
12	C	-0.0282	1.2967	0.8706
13	C	0.2131	2.4416	1.6783
14	C	-0.2974	1.5664	-0.4982
15	C	0.2389	3.7276	1.1686
16	H	0.3933	2.2997	2.7421
17	C	-0.2781	2.8508	-1.0190
18	H	-0.5816	0.7539	-1.1573
19	C	0.0177	3.9765	-0.2105
20	H	-0.5160	2.9796	-2.0684
21	C	-0.4235	-6.3703	0.1056
22	C	0.3963	-5.4863	-2.0803
23	H	-1.5016	-6.2209	0.2750
24	H	0.0613	-6.4805	1.0827
25	H	-0.3848	-5.0154	-2.6978
26	H	1.3656	-5.0783	-2.3901
27	N	0.1779	-5.2860	-0.6559
28	N	0.0860	5.2578	-0.7395
29	C	-0.3761	5.4894	-2.0996
30	C	0.1322	6.3969	0.1650
31	H	-1.4437	5.2520	-2.2324
32	H	0.1975	4.8875	-2.8135
33	H	-0.7531	6.4568	0.8178
34	H	1.0225	6.3541	0.8027
35	H	0.4327	4.5483	1.8491
36	H	-0.2875	-7.3078	-0.4362
37	H	0.4063	-6.5562	-2.2949
38	H	0.1911	7.3164	-0.4201
39	H	-0.2226	6.5390	-2.3565
40	H	-0.1099	0.0071	2.5669

FLUOar2dmn2_rad_MeButa
Energy (POTENTIAL) = -770.1524352 Eh

	Atom	X	Y	Z
1	C	0.0069	0.0001	1.4893
2	C	0.0287	-1.2828	0.9016
3	C	0.3566	-1.5858	-0.4676
4	C	-0.2288	-2.4362	1.7241
5	C	0.3763	-2.8722	-0.9511
6	H	0.6395	-0.7872	-1.1396
7	C	-0.2097	-3.7158	1.2445
8	H	-0.4621	-2.2736	2.7728
9	C	0.0832	-3.9918	-0.1282
10	H	0.6354	-3.0192	-1.9926
11	H	-0.4285	-4.5267	1.9280
12	C	0.0059	1.2805	0.8957
13	C	0.2294	2.4378	1.7221
14	C	-0.2773	1.5777	-0.4848
15	C	0.2356	3.7146	1.2344
16	H	0.4084	2.2815	2.7824
17	C	-0.2727	2.8614	-0.9761
18	H	-0.5561	0.7782	-1.1575
19	C	0.0093	3.9831	-0.1524
20	H	-0.5108	3.0048	-2.0230
21	C	-0.4062	-6.3701	0.2001
22	C	0.2424	-5.5069	-2.0425
23	H	-1.4698	-6.2390	0.4423
24	H	0.1522	-6.4546	1.1363
25	H	-0.5907	-5.0697	-2.6105
26	H	1.1759	-5.0778	-2.4172
27	N	0.1038	-5.2829	-0.6158
28	N	0.0755	5.2658	-0.6596
29	C	-0.3457	5.5174	-2.0250
30	C	0.1304	6.3972	0.2485
31	H	-1.4133	5.3027	-2.1748
32	H	0.2294	4.9088	-2.7279
33	H	-0.7691	6.4678	0.8759
34	H	1.0032	6.3322	0.9032
35	H	0.4172	4.5293	1.9242
36	H	-0.2884	-7.3082	-0.3404
37	H	0.2687	-6.5781	-2.2367
38	H	0.2199	7.3152	-0.3307
39	H	-0.1663	6.5638	-2.2684
40	H	-0.0124	0.0024	2.5780

ar2dmnomeobn
Energy (POTENTIAL) = -1136.04957872 Eh

	Atom	X	Y	Z
1	C	-0.3489	-0.5033	-1.3452
2	C	-1.4251	-1.4533	-0.8403
3	C	-2.5108	-1.7636	-1.6745
4	C	-1.4343	-1.9672	0.4603
5	C	-3.5592	-2.5603	-1.2323
6	H	-2.5336	-1.3716	-2.6888
7	C	-2.4793	-2.7692	0.9263
8	H	-0.6058	-1.7486	1.1275
9	C	-3.5513	-3.0701	0.0757
10	H	-4.3944	-2.8047	-1.8816
11	H	-2.4454	-3.1484	1.9409
12	C	1.0702	-0.7952	-0.8626
13	C	1.9851	0.2451	-0.6619
14	C	1.5452	-2.1020	-0.6961
15	C	3.3043	0.0044	-0.2887
16	H	1.6533	1.2701	-0.7936
17	C	2.8634	-2.3647	-0.3325
18	H	0.8723	-2.9419	-0.8453
19	C	3.7885	-1.3147	-0.1218
20	H	3.9572	0.8544	-0.1301
21	H	3.1661	-3.3975	-0.2088
22	N	5.1117	-1.5675	0.2192
23	C	5.9404	-0.4633	0.6830
24	C	5.4850	-2.9115	0.6380
25	H	5.5361	0.0220	1.5857
26	H	6.0453	0.3012	-0.0951
27	H	4.9293	-3.2494	1.5273

28	H	5.3104	-3.6348	-0.1664
29	C	-4.6686	-4.3898	1.7327
30	H	-3.8164	-5.0550	1.9204
31	H	-4.6864	-3.5999	2.4940
32	O	-0.7111	0.8766	-1.1236
33	C	-0.9850	1.2348	0.2385
34	H	-1.9738	0.8553	0.5300
35	H	-0.2446	0.7841	0.9116
36	C	-0.9352	2.7368	0.3336
37	C	0.2025	3.3920	0.8177
38	C	-2.0145	3.5126	-0.1077
39	C	0.2578	4.7861	0.8648
40	H	1.0533	2.8062	1.1586
41	C	-1.9571	4.9048	-0.0633
42	H	-2.9044	3.0193	-0.4920
43	C	-0.8209	5.5661	0.4272
44	H	1.1514	5.2754	1.2460
45	H	-2.8056	5.4894	-0.4121
46	C	-0.7761	7.0743	0.5076
47	H	-1.3385	7.5359	-0.3113
48	H	-1.2189	7.4320	1.4467
49	H	0.2527	7.4474	0.4696
50	H	-5.5948	-4.9641	1.7849
51	H	6.9397	-0.8376	0.9117
52	H	6.5511	-2.9306	0.8700
53	H	-0.3503	-0.5632	-2.4413
54	O	-4.6220	-3.8433	0.4163

mln_rad
Energy (POTENTIAL) = -263.669902544 Eh

	Atom	X	Y	Z
1	C	-0.0240	0.2990	0.0001
2	C	-1.1996	-0.4690	0.0001
3	N	-2.1915	-1.0912	0.0001
4	C	1.2051	-0.3873	-0.0000
5	C	-0.0614	1.8030	0.0002
6	H	0.4557	2.1922	0.8855
7	H	0.4582	2.1924	-0.8834
8	H	-1.0906	2.1645	-0.0011
9	N	2.2388	-0.9349	0.0000

ar2dmn2_rad_MeButa
Energy (POTENTIAL) = -769.955572220 Eh

	Atom	X	Y	Z
1	C	-0.0170	-0.0010	1.4740
2	C	0.0372	-1.3022	0.8803
3	C	0.4402	-1.5849	-0.4528
4	C	-0.2870	-2.4395	1.6703
5	C	0.4782	-2.8749	-0.9585
6	H	0.7725	-0.7771	-1.0949
7	C	-0.2570	-3.7310	1.1752
8	H	-0.5894	-2.2855	2.7042
9	C	0.1260	-3.9956	-0.1653
10	H	0.8023	-3.0125	-1.9834
11	H	-0.5355	-4.5439	1.8355
12	C	-0.0155	1.2958	0.8685
13	C	0.2330	2.4392	1.6767
14	C	-0.3031	1.5694	-0.4960
15	C	0.2486	3.7270	1.1709
16	H	0.4271	2.2946	2.7376
17	C	-0.2940	2.8555	-1.0129
18	H	-0.5933	0.7587	-1.1547
19	C	0.0092	3.9798	-0.2048
20	H	-0.5456	2.9870	-2.0588
21	C	-0.3958	-6.3760	0.1109
22	C	0.3644	-5.4872	-2.0960
23	H	-1.4700	-6.2351	0.3086
24	H	0.1157	-6.4814	1.0748
25	H	-0.4300	-5.0161	-2.6959
26	H	1.3267	-5.0789	-2.4268
27	N	0.1762	-5.2870	-0.6669
28	N	0.0673	5.2623	-0.7301
29	C	-0.4079	5.4970	-2.0856
30	C	0.1230	6.3994	0.1770
31	H	-1.4760	5.2576	-2.2088
32	H	0.1604	4.8984	-2.8065
33	H	-0.7536	6.4546	0.8416
34	H	1.0219	6.3573	0.8026
35	H	0.4486	4.5461	1.8516
36	H	-0.2665	-7.3125	-0.4342
37	H	0.3695	-6.5569	-2.3110
38	H	0.1711	7.3204	-0.4064
39	H	-0.2591	6.5476	-2.3405
40	H	-0.0663	0.0028	2.5629

SP_ar2dmn2mln_MeButa
Energy (POTENTIAL) = -1033.9699572 Eh

	Atom	X	Y	Z
1	C	-1.1928	0.0376	0.4667
2	C	-0.4217	1.3423	0.3367
3	C	-0.0408	2.0161	1.5044
4	C	0.6972	3.1943	1.4755
5	C	1.1015	3.7711	0.2482
6	C	0.6958	3.1047	-0.9325
7	C	-0.0361	1.9224	-0.8809
8	H	-0.3197	1.4646	-1.8229
9	H	0.9495	3.5067	-1.9058
10	H	0.9522	3.6658	2.4168
11	H	-0.3281	1.6097	2.4719
12	C	-2.5244	-0.0004	-0.4178
13	C	-2.2333	-0.2108	-1.8503
14	N	-2.0139	-0.3514	-2.9808
15	C	-0.3555	-1.2223	0.2895
16	C	0.6039	-1.3668	-0.7211
17	C	1.3342	-2.5397	-0.8748
18	C	1.1353	-3.6468	-0.0145
19	C	0.1840	-3.4896	1.0211
20	C	-0.5373	-2.3084	1.1558
21	H	-1.2628	-2.2332	1.9625
22	H	0.0031	-4.2879	1.7303
23	H	2.0681	-2.5872	-1.6701
24	H	0.8047	-0.5444	-1.3996
25	N	1.8601	4.9291	0.2047
26	C	2.0575	5.6029	-1.0705
27	C	2.0580	5.6947	1.4265
28	H	1.1097	5.9210	-1.5324
29	H	2.5809	4.9525	-1.7810
30	H	1.1103	6.0452	1.8646
31	H	2.5820	5.0982	2.1825
32	N	1.8384	-4.8286	-0.1796
33	C	2.9534	-4.8701	-1.1136
34	C	1.7645	-5.8507	0.8536

35	H	3.7505	-4.1560	-0.8534
36	H	2.6189	-4.6490	-2.1340
37	H	2.1267	-5.4922	1.8300
38	H	0.7350	-6.2052	0.9825
39	H	2.6777	6.5657	1.2074
40	H	2.6779	6.4870	-0.9157
41	H	-1.5913	0.0160	1.4874
42	H	2.3721	-6.7066	0.5553
43	H	3.3801	-5.8744	-1.1175
44	C	-3.3380	-1.1511	0.0282
45	N	-3.9944	-2.0245	0.4183
46	C	-3.3657	1.2931	-0.2402
47	H	-3.5597	1.4458	0.8248
48	H	-2.8059	2.1475	-0.6230
49	H	-4.3173	1.2137	-0.7711

UVar3m2_rad_MeButa
Energy (POTENTIAL) = -1345.219461 Eh

	Atom	X	Y	Z
1	C	0.0366	-0.0115	1.2387
2	C	0.0624	-1.2831	0.5243
3	C	0.7344	-1.4313	-0.7161
4	C	-0.5800	-2.4405	1.0211
5	C	0.7624	-2.6351	-1.3988
6	H	1.2736	-0.5838	-1.1276
7	C	-0.5681	-3.6490	0.3339
8	H	-1.1314	-2.3783	1.9542
9	C	0.1053	-3.7813	-0.8971
10	H	1.3347	-2.7030	-2.3188
11	H	-1.1164	-4.4853	0.7524
12	C	-0.0044	0.0098	2.7013
13	C	-0.7035	1.0169	3.4092
14	C	0.6474	-0.9760	3.4809
15	C	-0.7473	1.0317	4.7989
16	H	-1.2362	1.7807	2.8511
17	C	0.6009	-0.9519	4.8703
18	H	1.2084	-1.7589	2.9799
19	C	-0.0950	0.0515	5.5622
20	H	-1.3094	1.8143	5.3046
21	H	1.1223	-1.7236	5.4330
22	C	0.0458	1.2454	0.4936
23	C	0.6883	2.4042	0.9845
24	C	-0.5903	1.3750	-0.7667
25	C	0.6956	3.6033	0.2805
26	H	1.1993	2.3587	1.9414
27	C	-0.5824	2.5640	-1.4761
28	H	-1.1069	0.5178	-1.1871
29	C	0.0611	3.7168	-0.9729
30	H	-1.0688	2.5989	-2.4460
31	C	-0.3591	-6.1993	-0.9610
32	C	-0.1464	-4.9705	-3.0347
33	C	0.1283	-7.4416	-1.7061
34	H	-1.4635	-6.1929	-0.9459
35	H	-0.0039	-6.2501	0.0727
36	C	0.3366	-6.2520	-3.7062
37	H	-1.2369	-4.8744	-3.1780
38	H	0.3304	-4.1133	-3.5131
39	H	-0.3367	-8.3385	-1.2867
40	H	1.2222	-7.5260	-1.5986
41	H	0.0206	-6.2739	-4.7532
42	H	1.4373	-6.2981	-3.6654
43	N	0.1776	-4.9986	-1.6000
44	O	-0.2255	-7.4030	-3.0841
45	N	0.0851	4.9002	-1.7357
46	C	-1.1971	5.3844	-2.2704
47	C	0.9131	6.0097	-1.2645
48	C	-0.9507	6.4275	-3.3554
49	H	-1.8012	5.8350	-1.4636
50	H	-1.7683	4.5561	-2.6930
51	C	1.0852	7.0359	-2.3836
52	H	0.4686	6.5029	-0.3817
53	H	1.9004	5.6315	-0.9827
54	H	-1.8993	6.8535	-3.6949
55	H	-0.4451	5.9548	-4.2135
56	O	-0.1687	7.5103	-2.8615
57	H	1.6317	7.9083	-2.0135
58	H	1.6579	6.5830	-3.2095
59	H	1.1973	4.4572	0.7214
60	C	-0.1159	0.0909	7.0710
61	H	0.7453	0.6476	7.4656
62	H	-1.0192	0.5834	7.4470
63	H	-0.0726	-0.9164	7.4998

SP_ar2dmn2_cat_PMMA
Energy (POTENTIAL) = -770.0098743 Eh

	Atom	X	Y	Z
1	C	-0.0274	0.0010	1.3953
2	C	0.0112	-1.2854	0.8186
3	C	0.3339	-1.5738	-0.5425
4	C	-0.2459	-2.4103	1.6612
5	C	0.3399	-2.8554	-1.0298
6	H	0.6451	-0.7736	-1.2026
7	C	-0.2642	-3.6963	1.1856
8	H	-0.4626	-2.2368	2.7119
9	C	0.0132	-3.9697	-0.1908
10	H	0.6263	-3.0176	-2.0607
11	H	-0.4884	-4.5048	1.8690
12	C	-0.0650	1.2879	0.8197
13	C	0.1907	2.4121	1.6638
14	C	-0.3852	1.5777	-0.5416
15	C	0.2099	3.6986	1.1895
16	H	0.4057	2.2376	2.7147
17	C	-0.3905	2.8597	-1.0278
18	H	-0.6953	0.7781	-1.2031
19	C	-0.0652	3.9733	-0.1871
20	H	-0.6749	3.0229	-2.0590
21	C	-0.2887	-6.3645	0.2081
22	C	0.2785	-5.4926	-2.0893
23	H	-1.2867	-6.2769	0.6517
24	H	0.4526	-6.4311	1.0124
25	H	0.1576	-6.5568	-2.2858
26	H	-0.4137	-4.9431	-2.7361
27	N	-0.0065	-5.2314	-0.6763
28	N	-0.0448	5.2354	-0.6714
29	C	-0.3276	5.4981	-2.0846
30	C	0.2356	6.3676	0.2147
31	H	-1.3545	5.2144	-2.3425
32	H	0.3657	4.9493	-2.7308
33	H	0.1976	7.2897	-0.3631
34	H	-0.5069	6.4331	1.0179
35	H	0.4331	4.5063	1.8739
36	H	-0.2502	-7.2859	-0.3706

37	H	1.3059	-5.2088	-2.3453
38	H	1.2330	6.2800	0.6596
39	H	-0.2064	6.5624	-2.2798
40	H	-0.0283	0.0005	2.4854

SP_ar2dmnomeobn_meoh
Energy (POTENTIAL) = -1252.1323025 Eh

	Atom	X	Y	Z
1	C	-0.5318	-0.3845	-1.0877
2	C	-1.9407	-0.5232	-0.5349
3	C	-3.0011	0.0818	-1.2307
4	C	-2.2238	-1.1399	0.6870
5	C	-4.2960	0.0562	-0.7313
6	H	-2.7989	0.6054	-2.1616
7	C	-3.5211	-1.1780	1.2050
8	H	-1.4226	-1.6094	1.2494
9	C	-4.5675	-0.5799	0.4912
10	H	-5.1166	0.5206	-1.2697
11	H	-3.6993	-1.6701	2.1538
12	C	0.4455	-1.5015	-0.7452
13	C	1.7985	-1.2326	-0.5078
14	C	0.0532	-2.8462	-0.7535
15	C	2.7176	-2.2490	-0.2627
16	H	2.1428	-0.2032	-0.5090
17	C	0.9585	-3.8771	-0.5204
18	H	-0.9865	-3.1015	-0.9395
19	C	2.3259	-3.6086	-0.2709
20	H	3.7471	-1.9743	-0.0670
21	H	0.5909	-4.8961	-0.5294
22	N	3.2407	-4.6303	-0.0569
23	C	4.5658	-4.2956	0.4457
24	C	2.7491	-5.9819	0.1721
25	H	4.5326	-3.7792	1.4181
26	H	5.1027	-3.6532	-0.2616
27	H	2.0907	-6.0511	1.0526
28	H	2.1925	-6.3493	-0.6976
29	C	-6.2079	-1.1885	2.1265
30	H	-5.9730	-2.2601	2.1068
31	H	-5.6883	-0.7187	2.9710
32	O	0.0293	0.9166	-0.7609
33	C	0.0400	1.2698	0.6392
34	H	-0.9810	1.5137	0.9576
35	H	0.3852	0.4168	1.2339
36	C	0.9624	2.4474	0.8005
37	C	2.2299	2.2982	1.3719
38	C	0.5772	3.7155	0.3421
39	C	3.0914	3.3912	1.4901
40	H	2.5479	1.3199	1.7250
41	C	1.4423	4.8019	0.4548
42	H	-0.3948	3.8410	-0.1283
43	C	2.7122	4.6607	1.0364
44	H	4.0727	3.2544	1.9388
45	H	1.1288	5.7770	0.0883
46	C	3.6292	5.8515	1.1888
47	H	3.5558	6.5258	0.3284
48	H	3.3687	6.4370	2.0805
49	H	4.6743	5.5428	1.2938
50	C	-0.3988	3.3297	-3.1194
51	H	-0.8814	4.0813	-3.7524
52	H	0.4302	3.8136	-2.5810
53	H	0.0244	2.5515	-3.7733
54	O	-1.3836	2.8140	-2.2328
55	H	-0.9288	2.1451	-1.6778
56	H	-0.6098	-0.3195	-2.1802
57	H	5.1464	-5.2121	0.5625
58	H	3.5982	-6.6502	0.3243
59	H	-7.2844	-1.0556	2.2444
60	O	-5.8702	-0.5592	0.8918

SP_ar2dmn2_rad
Energy (POTENTIAL) = -770.1569508 Eh

	Atom	X	Y	Z
1	C	-0.0424	0.0006	1.4691
2	C	0.0166	-1.3016	0.8774
3	C	0.4325	-1.5880	-0.4515
4	C	-0.3173	-2.4375	1.6665
5	C	0.4741	-2.8792	-0.9542
6	H	0.7710	-0.7825	-1.0933
7	C	-0.2841	-3.7302	1.1746
8	H	-0.6296	-2.2813	2.6972
9	C	0.1123	-3.9985	-0.1621
10	H	0.8078	-3.0192	-1.9757
11	H	-0.5702	-4.5413	1.8338
12	C	-0.0300	1.2976	0.8638
13	C	0.2041	2.4410	1.6773
14	C	-0.2929	1.5729	-0.5059
15	C	0.2288	3.7293	1.1731
16	H	0.3785	2.2959	2.7416
17	C	-0.2744	2.8596	-1.0217
18	H	-0.5704	0.7635	-1.1715
19	C	0.0145	3.9838	-0.2072
20	H	-0.5067	2.9917	-2.0719
21	C	-0.4020	-6.3803	0.1195
22	C	0.3810	-5.4959	-2.0848
23	H	-1.4769	-6.2436	0.3143
24	H	0.1078	-6.4812	1.0848
25	H	-0.3998	-5.0231	-2.7004
26	H	1.3512	-5.0931	-2.3989
27	N	0.1655	-5.2892	-0.6599
28	N	0.0826	5.2653	-0.7299
29	C	-0.3634	5.5019	-2.0957
30	C	0.1296	6.4029	0.1782
31	H	-1.4287	5.2633	-2.2407
32	H	0.2192	4.9036	-2.8051
33	H	-0.7561	6.4601	0.8300
34	H	1.0194	6.3582	0.8164
35	H	0.4162	4.5478	1.8581
36	H	-0.2677	-7.3168	-0.4240
37	H	0.3841	-6.5662	-2.2957
38	H	0.1878	7.3234	-0.4047
39	H	-0.2090	6.5527	-2.3458
40	H	-0.1067	0.0050	2.5572

ar2dmn2_rad
Energy (POTENTIAL) = -769.957738802 Eh

	Atom	X	Y	Z
1	C	-0.0424	0.0006	1.4691
2	C	0.0166	-1.3016	0.8774
3	C	0.4325	-1.5880	-0.4515
4	C	-0.3173	-2.4375	1.6665
5	C	0.4741	-2.8792	-0.9542
6	H	0.7710	-0.7825	-1.0933

7	C	-0.2841	-3.7302	1.1746
8	H	-0.6296	-2.2813	2.6972
9	C	0.1123	-3.9985	-0.1621
10	H	0.8078	-3.0192	-1.9757
11	H	-0.5702	-4.5413	1.8338
12	C	-0.0300	1.2976	0.8638
13	C	0.2041	2.4410	1.6773
14	C	-0.2929	1.5729	-0.5059
15	C	0.2288	3.7293	1.1731
16	H	0.3785	2.2959	2.7416
17	C	-0.2744	2.8596	-1.0217
18	H	-0.5704	0.7635	-1.1715
19	C	0.0145	3.9838	-0.2072
20	H	-0.5067	2.9917	-2.0719
21	C	-0.4020	-6.3803	0.1195
22	C	0.3810	-5.4959	-2.0849
23	H	-1.4769	-6.2436	0.3143
24	H	0.1078	-6.4812	1.0848
25	H	-0.3998	-5.0231	-2.7004
26	H	1.3512	-5.0931	-2.3989
27	N	0.1655	-5.2892	-0.6599
28	N	0.0825	5.2653	-0.7299
29	C	-0.3634	5.5019	-2.0957
30	C	0.1296	6.4029	0.1782
31	H	-1.4287	5.2633	-2.2407
32	H	0.2192	4.9036	-2.8051
33	H	-0.7561	6.4601	0.8300
34	H	1.0194	6.3582	0.8164
35	H	0.4162	4.5478	1.8581
36	H	-0.2677	-7.3168	-0.4240
37	H	0.3841	-6.5662	-2.2957
38	H	0.1878	7.3234	-0.4047
39	H	-0.2090	6.5527	-2.3458
40	H	-0.1067	0.0050	2.5572

SP_ar2dmnome_rad
Energy (POTENTIAL) = -750.7053575 Eh

	Atom	X	Y	Z
1	C	-0.1206	-0.4168	1.3467
2	C	-0.0296	-1.6933	0.6969
3	C	0.4852	-1.9092	-0.6132
4	C	-0.4292	-2.8546	1.4089
5	C	0.5510	-3.1762	-1.1684
6	H	0.8796	-1.0746	-1.1809
7	C	-0.3693	-4.1303	0.8586
8	H	-0.8097	-2.7411	2.4215
9	C	0.1149	-4.3018	-0.4479
10	H	0.9562	-3.3257	-2.1651
11	H	-0.6984	-4.9780	1.4487
12	C	-0.0748	0.9047	0.8049
13	C	0.0998	2.0078	1.6874
14	C	-0.2468	1.2446	-0.5655
15	C	0.1545	3.3178	1.2489
16	H	0.2035	1.8110	2.7525
17	C	-0.1979	2.5534	-1.0163
18	H	-0.4764	0.4677	-1.2859
19	C	0.0316	3.6377	-0.1299
20	H	-0.3600	2.7370	-2.0718
21	C	-0.1996	-6.6753	-0.4038
22	H	-1.2631	-6.6279	-0.1370
23	H	0.3941	-6.8386	0.5046
24	N	0.1255	4.9396	-0.5831
25	C	-0.1891	5.2423	-1.9723
26	C	0.1478	6.0346	0.3768
27	H	-1.2332	5.0057	-2.2285
28	H	0.4638	4.6827	-2.6521
29	H	-0.7625	6.0695	0.9944
30	H	1.0114	5.9524	1.0471
31	H	0.2929	4.1027	1.9829
32	H	-0.0367	-7.5028	-1.0961
33	H	0.2347	6.9799	-0.1603
34	H	-0.0234	6.3051	-2.1539
35	H	-0.2465	-0.4645	2.4281
36	O	0.2171	-5.4999	-1.0943

ar2dmnomeobn_meoh_PMMA
Energy (POTENTIAL) = -1251.78617454 Eh

	Atom	X	Y	Z
1	C	-0.5275	-0.3904	-1.0824
2	C	-1.9346	-0.5224	-0.5225
3	C	-2.9880	0.1221	-1.1937
4	C	-2.2201	-1.1666	0.6839
5	C	-4.2795	0.1045	-0.6865
6	H	-2.7808	0.6727	-2.1078
7	C	-3.5151	-1.1973	1.2086
8	H	-1.4227	-1.6632	1.2283
9	C	-4.5548	-0.5620	0.5184
10	H	-5.0949	0.6008	-1.2037
11	H	-3.6962	-1.7125	2.1447
12	C	0.4478	-1.5106	-0.7449
13	C	1.7987	-1.2438	-0.4955
14	C	0.0554	-2.8549	-0.7696
15	C	2.7153	-2.2629	-0.2530
16	H	2.1427	-0.2143	-0.4888
17	C	0.9587	-3.8878	-0.5390
18	H	-0.9828	-3.1076	-0.9671
19	C	2.3233	-3.6213	-0.2763
20	H	3.7440	-1.9907	-0.0496
21	H	0.5918	-4.9068	-0.5619
22	N	3.2369	-4.6462	-0.0638
23	C	4.5585	-4.3162	0.4475
24	C	2.7431	-5.9984	0.1494
25	H	4.5220	-3.8070	1.4240
26	H	5.0992	-3.6689	-0.2526
27	H	2.0750	-6.0751	1.0224
28	H	2.1960	-6.3592	-0.7292
29	C	-6.1954	-1.1828	2.1462
30	H	-5.9810	-2.2584	2.0995
31	H	-5.6618	-0.7444	2.9994
32	O	0.0403	0.9074	-0.7586
33	C	0.0399	1.2685	0.6382
34	H	-0.9845	1.5078	0.9495
35	H	0.3868	0.4208	1.2401
36	C	0.9533	2.4532	0.7986
37	C	2.2121	2.3191	1.3913
38	C	0.5664	3.7132	0.3201
39	C	3.0641	3.4190	1.5116
40	H	2.5309	1.3463	1.7593
41	C	1.4222	4.8062	0.4351
42	H	-0.3982	3.8265	-0.1683
43	C	2.6832	4.6804	1.0385
44	H	4.0392	3.2942	1.9771

45	H	1.1079	5.7751	0.0530
46	C	3.5890	5.8796	1.1927
47	H	3.5286	6.5415	0.3216
48	H	3.3079	6.4757	2.0711
49	H	4.6343	5.5801	1.3213
50	C	-0.3597	3.3146	-3.1540
51	H	-0.8474	4.0562	-3.7950
52	H	0.4594	3.8118	-2.6122
53	H	0.0796	2.5388	-3.8008
54	O	-1.3442	2.7921	-2.2734
55	H	-0.8866	2.1367	-1.7055
56	H	-0.6104	-0.3260	-2.1747
57	H	5.1389	-5.2337	0.5597
58	H	3.5903	-6.6686	0.3053
59	H	-7.2686	-1.0335	2.2757
60	O	-5.8553	-0.5303	0.9272

SP_ar2dmn2mln
Energy (POTENTIAL) = -1033.9737334 Eh

	Atom	X	Y	Z
1	C	-1.1934	0.0411	0.4622
2	C	-0.4193	1.3440	0.3327
3	C	-0.0458	2.0210	1.5015
4	C	0.6947	3.1978	1.4743
5	C	1.1094	3.7704	0.2479
6	C	0.7104	3.1010	-0.9340
7	C	-0.0243	1.9201	-0.8839
8	H	-0.3029	1.4615	-1.8269
9	H	0.9707	3.5001	-1.9066
10	H	0.9428	3.6720	2.4160
11	H	-0.3423	1.6192	2.4681
12	C	-2.5252	0.0064	-0.4223
13	C	-2.2347	-0.2004	-1.8555
14	N	-2.0156	-0.3447	-2.9857
15	C	-0.3593	-1.2211	0.2860
16	C	0.5849	-1.3784	-0.7375
17	C	1.3129	-2.5535	-0.8886
18	C	1.1277	-3.6505	-0.0113
19	C	0.1922	-3.4798	1.0373
20	C	-0.5272	-2.2968	1.1684
21	H	-1.2388	-2.2106	1.9864
22	H	0.0237	-4.2687	1.7601
23	H	2.0352	-2.6103	-1.6937
24	H	0.7758	-0.5645	-1.4291
25	N	1.8710	4.9258	0.2064
26	C	2.0746	5.5983	-1.0692
27	C	2.0584	5.6969	1.4275
28	H	1.1291	5.9183	-1.5341
29	H	2.5986	4.9461	-1.7774
30	H	1.1069	6.0509	1.8540
31	H	2.5733	5.1026	2.1912
32	N	1.8273	-4.8339	-0.1730
33	C	2.9300	-4.8866	-1.1224
34	C	1.7725	-5.8420	0.8763
35	H	3.7300	-4.1694	-0.8813
36	H	2.5814	-4.6775	-2.1405
37	H	2.1529	-5.4694	1.8401
38	H	0.7456	-6.1941	1.0294
39	H	2.6819	6.5654	1.2103
40	H	2.6966	6.4809	-0.9127
41	H	-1.5922	0.0205	1.4825
42	H	2.3745	-6.7018	0.5786
43	H	3.3567	-5.8906	-1.1198
44	C	-3.3350	-1.1501	0.0159
45	N	-3.9853	-2.0331	0.3943
46	C	-3.3678	1.2981	-0.2387
47	H	-3.5626	1.4445	0.8269
48	H	-2.8072	2.1541	-0.6167
49	H	-4.3184	1.2216	-0.7715

FLUOrefdiarcn_rad_MeButa
Energy (POTENTIAL) = -823.5281981 Eh

	Atom	X	Y	Z
1	C	-0.0194	-0.0202	1.1517
2	C	0.0163	-1.3038	0.4990
3	C	0.5303	-1.4950	-0.8234
4	C	-0.4030	-2.4886	1.1887
5	C	0.5943	-2.7366	-1.3975
6	H	0.9160	-0.6471	-1.3743
7	C	-0.3339	-3.7378	0.6190
8	H	-0.7917	-2.3928	2.1962
9	C	0.1583	-3.8846	-0.6941
10	H	0.9929	-2.8682	-2.3978
11	H	-0.6707	-4.5967	1.1858
12	C	-0.0256	1.2636	0.4985
13	C	0.3588	2.4486	1.2079
14	C	-0.4768	1.4549	-0.8466
15	C	0.3137	3.6982	0.6367
16	H	0.7003	2.3527	2.2324
17	C	-0.5168	2.6970	-1.4218
18	H	-0.8341	0.6069	-1.4161
19	C	-0.1174	3.8453	-0.6976
20	H	-0.8681	2.8287	-2.4396
21	C	-0.1674	-6.2535	-0.7132
22	H	-1.2305	-6.2069	-0.4592
23	H	0.4247	-6.4383	0.1880
24	H	0.6224	4.5572	1.2193
25	H	0.0029	-7.0512	-1.4337
26	O	0.2525	-5.0525	-1.3574
27	O	-0.1840	5.0137	-1.3634
28	C	0.2001	6.2154	-0.6986
29	H	0.0608	7.0136	-1.4251
30	H	1.2503	6.1727	-0.3949
31	H	-0.4344	6.3963	0.1741
32	C	-0.0519	-0.0206	2.5603
33	N	-0.0792	-0.0212	3.7276

ar2dmn2obn_meoh
Energy (POTENTIAL) = -1271.23928971 Eh

	Atom	X	Y	Z
1	C	-0.2986	-0.3234	-1.1012
2	C	-1.7099	-0.3759	-0.5484
3	C	-2.7327	0.3099	-1.2177
4	C	-2.0402	-0.9975	0.6627
5	C	-4.0283	0.3678	-0.7176
6	H	-2.5040	0.8339	-2.1429
7	C	-3.3303	-0.9537	1.1810
8	H	-1.2759	-1.5399	1.2120
9	C	-4.3660	-0.2611	0.5068
10	H	-4.7791	0.9045	-1.2847
11	H	-3.5283	-1.4634	2.1161
12	C	0.5924	-1.5192	-0.7894

13	C	1.9576	-1.3642	-0.5231
14	C	0.0984	-2.8288	-0.8495
15	C	2.7917	-2.4570	-0.3016
16	H	2.3805	-0.3654	-0.4816
17	C	0.9178	-3.9342	-0.6417
18	H	-0.9556	-2.9945	-1.0553
19	C	2.2976	-3.7810	-0.3649
20	H	3.8358	-2.2695	-0.0815
21	H	0.4726	-4.9206	-0.6915
22	N	3.1284	-4.8784	-0.1776
23	C	4.4626	-4.6653	0.3658
24	C	2.5285	-6.1935	-0.0001
25	H	4.4455	-4.1866	1.3580
26	H	5.0625	-4.0377	-0.3031
27	H	1.8573	-6.2407	0.8722
28	H	1.9534	-6.4839	-0.8867
29	N	-5.6473	-0.1930	1.0248
30	C	-6.7221	0.3477	0.2046
31	C	-5.9996	-1.0198	2.1703
32	H	-6.8688	-0.2213	-0.7265
33	H	-6.5254	1.3925	-0.0635
34	H	-5.8771	-2.0950	1.9675
35	H	-5.3854	-0.7669	3.0427
36	O	0.3648	0.9238	-0.7440
37	C	0.3841	1.2486	0.6617
38	H	-0.6188	1.5665	0.9728
39	H	0.6517	0.3602	1.2445
40	C	1.3950	2.3457	0.8579
41	C	2.6351	2.0867	1.4494
42	C	1.1218	3.6477	0.4138
43	C	3.5793	3.1051	1.6016
44	H	2.8666	1.0807	1.7919
45	C	2.0687	4.6590	0.5603
46	H	0.1722	3.8578	-0.0719
47	C	3.3119	4.4075	1.1624
48	H	4.5373	2.8828	2.0659
49	H	1.8415	5.6617	0.2046
50	C	4.3178	5.5193	1.3490
51	H	4.3395	6.1882	0.4814
52	H	4.0671	6.1342	2.2235
53	H	5.3277	5.1259	1.5031
54	C	0.1332	3.3827	-3.0552
55	H	-0.2759	4.1818	-3.6819
56	H	1.0000	3.7835	-2.5081
57	H	0.4876	2.5766	-3.7162
58	O	-0.8986	2.9485	-2.1788
59	H	-0.5089	2.2324	-1.6327
60	H	-0.3704	-0.2239	-2.1918
61	H	4.9700	-5.6271	0.4562
62	H	3.3200	-6.9327	0.1338
63	H	-7.6531	0.3225	0.7725
64	H	-7.0416	-0.8374	2.4368

FLUOfl_cat_MeButa
Energy (POTENTIAL) = -771.1873699 Eh

	Atom	X	Y	Z
1	C	-0.0008	-0.0017	-0.0745
2	C	-0.0565	-1.1459	-0.9374
3	C	-0.0335	-0.6954	-2.3220
4	C	-0.1822	-2.5083	-0.6634
5	C	-0.1004	-1.6170	-3.3861
6	C	-0.2463	-3.3923	-1.7304
7	H	-0.2444	-2.8733	0.3539
8	C	-0.1989	-2.9543	-3.0817
9	H	-0.0839	-1.2765	-4.4150
10	H	-0.3438	-4.4543	-1.5333
11	C	-0.0011	-0.0061	1.3765
12	C	-0.7508	0.9372	2.1008
13	C	0.7454	-0.9540	2.0958
14	C	-0.7540	0.9236	3.4860
15	H	-1.3701	1.6540	1.5728
16	C	0.7467	-0.9484	3.4825
17	H	1.3609	-1.6720	1.5652
18	C	-0.0013	-0.0129	4.2034
19	H	-1.3578	1.6481	4.0240
20	H	1.3433	-1.6813	4.0168
21	C	0.0562	1.1484	-0.9294
22	C	0.1799	2.5091	-0.6459
23	C	0.0368	0.7073	-2.3171
24	C	0.2461	3.4003	-1.7067
25	H	0.2387	2.8673	0.3740
26	C	0.1061	1.6361	-3.3747
27	C	0.2028	2.9714	-3.0610
28	H	0.0924	1.3025	-4.4060
29	H	0.3421	4.4610	-1.5022
30	C	0.0190	0.0071	5.7063
31	H	0.7215	0.7655	6.0683
32	H	-0.9651	0.2535	6.1127
33	H	0.3317	-0.9563	6.1147
34	H	0.2598	3.7130	-3.8494
35	H	-0.2544	-3.6905	-3.8752

SP_ar2dmnomeobn_MeButa
Energy (POTENTIAL) = -1136.3447494 Eh

	Atom	X	Y	Z
1	C	-0.3539	-0.5079	-1.3480
2	C	-1.4289	-1.4574	-0.8387
3	C	-2.5233	-1.7577	-1.6650
4	C	-1.4292	-1.9785	0.4587
5	C	-3.5711	-2.5524	-1.2188
6	H	-2.5533	-1.3578	-2.6760
7	C	-2.4738	-2.7791	0.9286
8	H	-0.5939	-1.7670	1.1196
9	C	-3.5539	-3.0703	0.0857
10	H	-4.4136	-2.7894	-1.8614
11	H	-2.4330	-3.1645	1.9406
12	C	1.0659	-0.7962	-0.8653
13	C	1.9758	0.2473	-0.6602
14	C	1.5458	-2.1015	-0.7033
15	C	3.2955	0.0102	-0.2866
16	H	1.6393	1.2710	-0.7904
17	C	2.8645	-2.3600	-0.3392
18	H	0.8760	-2.9433	-0.8567
19	C	3.7844	-1.3071	-0.1239
20	H	3.9455	0.8619	-0.1258
21	H	3.1717	-3.3921	-0.2197
22	N	5.1090	-1.5559	0.2180
23	C	5.9331	-0.4503	0.6839
24	C	5.4870	-2.8992	0.6321
25	H	5.5274	0.0326	1.5876
26	H	6.0354	0.3159	-0.0930
27	H	4.9307	-3.2441	1.5186

28	H	5.3179	-3.6200	-0.1760
29	C	-4.6633	-4.3938	1.7429
30	H	-3.8127	-5.0640	1.9213
31	H	-4.6717	-3.6080	2.5091
32	O	-0.7173	0.8710	-1.1307
33	C	-0.9964	1.2306	0.2288
34	H	-1.9893	0.8567	0.5147
35	H	-0.2628	0.7758	0.9070
36	C	-0.9380	2.7322	0.3255
37	C	0.1871	3.3790	0.8479
38	C	-1.9964	3.5156	-0.1508
39	C	0.2498	4.7726	0.8998
40	H	1.0225	2.7866	1.2144
41	C	-1.9314	4.9070	-0.1017
42	H	-2.8749	3.0282	-0.5674
43	C	-0.8084	5.5599	0.4281
44	H	1.1335	5.2555	1.3110
45	H	-2.7636	5.4979	-0.4784
46	C	-0.7568	7.0677	0.5132
47	H	-1.2899	7.5346	-0.3222
48	H	-1.2264	7.4271	1.4387
49	H	0.2749	7.4346	0.5077
50	H	-5.5916	-4.9645	1.8002
51	H	6.9341	-0.8210	0.9117
52	H	6.5528	-2.9150	0.8667
53	H	-0.3554	-0.5718	-2.4439
54	O	-4.6252	-3.8416	0.4304

ar2dmn2obn_PMMA
Energy (POTENTIAL) = -1155.49445441 Eh

	Atom	X	Y	Z
1	C	-0.3306	-0.1463	-1.3179
2	C	-1.7574	-0.0890	-0.7994
3	C	-2.7773	0.3960	-1.6263
4	C	-2.1097	-0.4231	0.5150
5	C	-4.0864	0.5419	-1.1791
6	H	-2.5447	0.6660	-2.6545
7	C	-3.4109	-0.2831	0.9860
8	H	-1.3520	-0.8178	1.1862
9	C	-4.4400	0.2168	0.1523
10	H	-4.8332	0.9081	-1.8733
11	H	-3.6224	-0.5674	2.0097
12	C	0.4985	-1.3380	-0.8419
13	C	1.8874	-1.2279	-0.7035
14	C	-0.0652	-2.5992	-0.6166
15	C	2.6786	-2.3114	-0.3334
16	H	2.3577	-0.2662	-0.8824
17	C	0.7101	-3.6976	-0.2538
18	H	-1.1386	-2.7328	-0.7175
19	C	2.1117	-3.5867	-0.1046
20	H	3.7456	-2.1562	-0.2258
21	H	0.2118	-4.6444	-0.0833
22	N	2.8970	-4.6832	0.2387
23	C	4.2795	-4.4584	0.6329
24	C	2.2436	-5.8861	0.7320
25	H	4.3714	-3.8004	1.5124
26	H	4.8516	-4.0098	-0.1871
27	H	1.6438	-5.7049	1.6388
28	H	1.5832	-6.3139	-0.0310
29	N	-5.7349	0.3853	0.6211
30	C	-6.7985	0.6836	-0.3256
31	C	-6.1023	-0.1829	1.9092
32	H	-6.9198	-0.1008	-1.0895
33	H	-6.6098	1.6327	-0.8408
34	H	-5.9644	-1.2752	1.9459
35	H	-5.5097	0.2610	2.7179
36	O	0.3723	1.0986	-1.1068
37	C	0.4444	1.5414	0.2533
38	H	-0.5261	1.9531	0.5631
39	H	0.6755	0.7004	0.9206
40	C	1.5248	2.5870	0.3377
41	C	2.8155	2.2564	0.7646
42	C	1.2712	3.9062	-0.0567
43	C	3.8243	3.2200	0.8013
44	H	3.0324	1.2341	1.0667
45	C	2.2800	4.8675	-0.0221
46	H	0.2750	4.1777	-0.3983
47	C	3.5737	4.5418	0.4110
48	H	4.8210	2.9417	1.1371
49	H	2.0627	5.8867	-0.3346
50	C	4.6551	5.5947	0.4816
51	H	4.5542	6.3279	-0.3262
52	H	4.6065	6.1481	1.4290
53	H	5.6536	5.1499	0.4149
54	H	-7.7413	0.7853	0.2147
55	H	-7.1517	0.0376	2.1122
56	H	4.7450	-5.4169	0.8700
57	H	3.0019	-6.6357	0.9655
58	H	-0.3820	-0.1897	-2.4137

ar2dmnomemln
Energy (POTENTIAL) = -1014.25465508 Eh

	Atom	X	Y	Z
1	C	-0.9991	-0.3808	0.4364
2	C	-0.4751	1.0384	0.2794
3	C	-0.1249	1.7539	1.4323
4	C	0.3971	3.0416	1.3773
5	C	0.5986	3.6944	0.1373
6	C	0.2215	2.9814	-1.0264
7	C	-0.2912	1.6903	-0.9494
8	H	-0.5661	1.2036	-1.8794
9	H	0.3244	3.4328	-2.0054
10	H	0.6404	3.5392	2.3081
11	H	-0.2632	1.2935	2.4083
12	C	-2.3733	-0.6400	-0.3371
13	C	-2.1747	-0.7743	-1.7946
14	N	-2.0292	-0.8654	-2.9420
15	C	0.0263	-1.4752	0.1602
16	C	0.8842	-1.4470	-0.9521
17	C	1.7922	-2.4716	-1.1839
18	C	1.8749	-3.5606	-0.3015
19	C	1.0353	-3.6021	0.8181
20	C	0.1259	-2.5634	1.0332
21	H	-0.5197	-2.6104	1.9064
22	H	1.0791	-4.4227	1.5241
23	H	2.4548	-2.4469	-2.0436
24	H	0.8553	-0.6106	-1.6414
25	N	1.1406	4.9652	0.0645
26	C	1.1195	5.6770	-1.2059
27	C	1.3091	5.7391	1.2865
28	H	0.0978	5.8297	-1.5868
29	H	1.6898	5.1348	-1.9692

30	H	0.3557	5.9192	1.8071
31	H	1.9862	5.2306	1.9826
32	C	2.9249	-5.6420	0.2427
33	H	3.2222	-5.3439	1.2559
34	H	1.9888	-6.2121	0.2922
35	H	1.7537	6.7043	1.0400
36	H	1.5875	6.6539	-1.0767
37	H	-1.2990	-0.4837	1.4849
38	H	3.7061	-6.2647	-0.1957
39	C	-2.9277	-1.9269	0.1346
40	N	-3.3748	-2.9173	0.5407
41	C	-3.4075	0.4823	-0.0499
42	H	-3.5305	0.5830	1.0316
43	H	-3.0400	1.4265	-0.4536
44	H	-4.3733	0.2448	-0.5017
45	O	2.7997	-4.5106	-0.6171

SP_ar2dmn2_rad_PMMA
Energy (POTENTIAL) = -770.1533774 Eh

	Atom	X	Y	Z
1	C	-0.0424	0.0014	1.4790
2	C	0.0193	-1.3004	0.8875
3	C	0.4498	-1.5843	-0.4365
4	C	-0.3259	-2.4360	1.6704
5	C	0.4941	-2.8745	-0.9410
6	H	0.7984	-0.7772	-1.0708
7	C	-0.2901	-3.7276	1.1759
8	H	-0.6499	-2.2807	2.6976
9	C	0.1210	-3.9934	-0.1556
10	H	0.8397	-3.0133	-1.9588
11	H	-0.5855	-4.5395	1.8301
12	C	-0.0282	1.2967	0.8706
13	C	0.2131	2.4416	1.6783
14	C	-0.2974	1.5664	-0.4982
15	C	0.2389	3.7276	1.1686
16	H	0.3933	2.2997	2.7421
17	C	-0.2781	2.8508	-1.0190
18	H	-0.5816	0.7539	-1.1573
19	C	0.0177	3.9765	-0.2105
20	H	-0.5160	2.9796	-2.0684
21	C	-0.4235	-6.3703	0.1056
22	C	0.3963	-5.4863	-2.0803
23	H	-1.5016	-6.2209	0.2750
24	H	0.0613	-6.4805	1.0827
25	H	-0.3848	-5.0154	-2.6978
26	H	1.3656	-5.0783	-2.3901
27	N	0.1779	-5.2860	-0.6559
28	N	0.0860	5.2578	-0.7395
29	C	-0.3761	5.4894	-2.0996
30	C	0.1322	6.3969	0.1650
31	H	-1.4437	5.2520	-2.2324
32	H	0.1975	4.8875	-2.8135
33	H	-0.7531	6.4568	0.8178
34	H	1.0225	6.3541	0.8027
35	H	0.4327	4.5483	1.8491
36	H	-0.2875	-7.3078	-0.4362
37	H	0.4063	-6.5562	-2.2949
38	H	0.1911	7.3164	-0.4201
39	H	-0.2226	6.5390	-2.3565
40	H	-0.1099	0.0071	2.5669

SP_ar2dmnomeobn
Energy (POTENTIAL) = -1136.3478016 Eh

	Atom	X	Y	Z
1	C	-0.3489	-0.5033	-1.3452
2	C	-1.4251	-1.4533	-0.8403
3	C	-2.5108	-1.7636	-1.6745
4	C	-1.4343	-1.9672	0.4603
5	C	-3.5592	-2.5603	-1.2323
6	H	-2.5336	-1.3716	-2.6888
7	C	-2.4793	-2.7692	0.9263
8	H	-0.6058	-1.7486	1.1275
9	C	-3.5513	-3.0701	0.0757
10	H	-4.3944	-2.8047	-1.8816
11	H	-2.4454	-3.1484	1.9409
12	C	1.0702	-0.7952	-0.8626
13	C	1.9851	0.2451	-0.6619
14	C	1.5452	-2.1020	-0.6961
15	C	3.3043	0.0044	-0.2887
16	H	1.6533	1.2701	-0.7936
17	C	2.8634	-2.3647	-0.3325
18	H	0.8723	-2.9419	-0.8453
19	C	3.7885	-1.3147	-0.1218
20	H	3.9572	0.8544	-0.1301
21	H	3.1661	-3.3975	-0.2088
22	N	5.1117	-1.5675	0.2192
23	C	5.9404	-0.4633	0.6830
24	C	5.4850	-2.9115	0.6380
25	H	5.5361	0.0220	1.5857
26	H	6.0453	0.3012	-0.0951
27	H	4.9293	-3.2494	1.5273
28	H	5.3104	-3.6348	-0.1664
29	C	-4.6686	-4.3898	1.7327
30	H	-3.8164	-5.0550	1.9204
31	H	-4.6864	-3.5999	2.4940
32	O	-0.7111	0.8766	-1.1236
33	C	-0.9850	1.2348	0.2385
34	H	-1.9738	0.8553	0.5300
35	H	-0.2446	0.7841	0.9116
36	C	-0.9352	2.7368	0.3336
37	C	0.2025	3.3920	0.8177
38	C	-2.0145	3.5126	-0.1077
39	C	0.2578	4.7861	0.8648
40	H	1.0533	2.8062	1.1586
41	C	-1.9571	4.9048	-0.0633
42	H	-2.9044	3.0193	-0.4920
43	C	-0.8209	5.5661	0.4272
44	H	1.1514	5.2754	1.2460
45	H	-2.8056	5.4894	-0.4121
46	C	-0.7761	7.0743	0.5076
47	H	-1.3385	7.5359	-0.3113
48	H	-1.2189	7.4320	1.4467
49	H	0.2527	7.4474	0.4696
50	H	-5.5948	-4.9641	1.7849
51	H	6.9397	-0.8376	0.9117
52	H	6.5511	-2.9306	0.8700
53	H	-0.3503	-0.5632	-2.4413
54	O	-4.6220	-3.8433	0.4163

UVrefdiarcn_rad_MeButa_cam
Energy (POTENTIAL) = -823.087017 Eh

	Atom	X	Y	Z

1	C	-0.0238	-0.0204	1.2098
2	C	0.0376	-1.3138	0.5450
3	C	0.6084	-1.4704	-0.7444
4	C	-0.4289	-2.4824	1.1887
5	C	0.6780	-2.7097	-1.3542
6	H	1.0331	-0.6108	-1.2510
7	C	-0.3651	-3.7317	0.5831
8	H	-0.8635	-2.4055	2.1810
9	C	0.1849	-3.8551	-0.7029
10	H	1.1282	-2.8252	-2.3351
11	H	-0.7464	-4.5962	1.1133
12	C	-0.0543	1.2736	0.5440
13	C	0.3830	2.4415	1.2092
14	C	-0.5660	1.4318	-0.7698
15	C	0.3476	3.6913	0.6024
16	H	0.7720	2.3635	2.2201
17	C	-0.6071	2.6716	-1.3810
18	H	-0.9683	0.5731	-1.2957
19	C	-0.1435	3.8163	-0.7072
20	H	-1.0126	2.7880	-2.3811
21	C	-0.1709	-6.2211	-0.7782
22	H	-1.2458	-6.1678	-0.5664
23	H	0.3747	-6.4330	0.1494
24	H	0.7051	4.5550	1.1502
25	H	0.0183	-7.0156	-1.5011
26	O	0.2965	-5.0195	-1.3912
27	O	-0.2232	4.9811	-1.3988
28	C	0.2154	6.1824	-0.7643
29	H	0.0585	6.9775	-1.4943
30	H	1.2797	6.1296	-0.5043
31	H	-0.3715	6.3931	0.1380
32	C	-0.0566	-0.0209	2.6234
33	N	-0.0837	-0.0213	3.7942

SP_ar2dmn2mln_PMMA
Energy (POTENTIAL) = -1033.9682266 Eh

	Atom	X	Y	Z
1	C	-1.1928	0.0402	0.4682
2	C	-0.4204	1.3437	0.3345
3	C	-0.0362	2.0191	1.5001
4	C	0.7030	3.1964	1.4676
5	C	1.1052	3.7703	0.2385
6	C	0.6965	3.1024	-0.9400
7	C	-0.0366	1.9210	-0.8849
8	H	-0.3224	1.4613	-1.8252
9	H	0.9489	3.5022	-1.9145
10	H	0.9608	3.6691	2.4074
11	H	-0.3213	1.6143	2.4690
12	C	-2.5244	0.0011	-0.4156
13	C	-2.2340	-0.2137	-1.8476
14	N	-2.0148	-0.3548	-2.9781
15	C	-0.3566	-1.2209	0.2940
16	C	0.6061	-1.3662	-0.7131
17	C	1.3351	-2.5402	-0.8646
18	C	1.1312	-3.6470	-0.0056
19	C	0.1764	-3.4892	1.0263
20	C	-0.5435	-2.3070	1.1589
21	H	-1.2727	-2.2316	1.9623
22	H	-0.0090	-4.2878	1.7340
23	H	2.0715	-2.5886	-1.6574
24	H	0.8103	-0.5440	-1.3907
25	N	1.8647	4.9279	0.1912
26	C	2.0607	5.5986	-1.0854
27	C	2.0677	5.6944	1.4112
28	H	1.1126	5.9165	-1.5470
29	H	2.5828	4.9465	-1.7953
30	H	1.1221	6.0473	1.8522
31	H	2.5931	5.0978	2.1663
32	N	1.8334	-4.8301	-0.1686
33	C	2.9512	-4.8727	-1.0988
34	C	1.7536	-5.8526	0.8631
35	H	3.7487	-4.1597	-0.8359
36	H	2.6203	-4.6509	-2.1202
37	H	2.1122	-5.4958	1.8416
38	H	0.7230	-6.2057	0.9873
39	H	2.6885	6.5640	1.1894
40	H	2.6819	6.4828	-0.9336
41	H	-1.5907	0.0215	1.4893
42	H	2.3610	-6.7094	0.5667
43	H	3.3767	-5.8775	-1.1017
44	C	-3.3407	-1.1456	0.0360
45	N	-4.0001	-2.0137	0.4325
46	C	-3.3634	1.2969	-0.2425
47	H	-3.5570	1.4542	0.8221
48	H	-2.8026	2.1491	-0.6286
49	H	-4.3155	1.2168	-0.7725

SP_mln_an_PMMA
Energy (POTENTIAL) = -263.894921 Eh

	Atom	X	Y	Z
1	C	-0.0390	0.2638	-0.0252
2	C	-1.1748	-0.5406	0.0715
3	N	-2.1512	-1.1997	0.1455
4	C	1.2203	-0.3365	-0.0762
5	C	-0.1576	1.7799	0.0002
6	H	0.1067	2.2279	0.9726
7	H	0.4888	2.2515	-0.7534
8	H	-1.1873	2.0834	-0.2229
9	N	2.2997	-0.8100	-0.1275

SP_ar2dmnome_rad_MeButa
Energy (POTENTIAL) = -750.7030453 Eh

	Atom	X	Y	Z
1	C	-0.1234	-0.4163	1.3558
2	C	-0.0304	-1.6917	0.7047
3	C	0.4985	-1.9045	-0.6001
4	C	-0.4411	-2.8537	1.4083
5	C	0.5674	-3.1694	-1.1588
6	H	0.9019	-1.0682	-1.1589
7	C	-0.3785	-4.1275	0.8542
8	H	-0.8330	-2.7420	2.4167
9	C	0.1198	-4.2958	-0.4470
10	H	0.9838	-3.3178	-2.1509
11	H	-0.7168	-4.9764	1.4374
12	C	-0.0741	0.9044	0.8122
13	C	0.1090	2.0085	1.6904
14	C	-0.2519	1.2403	-0.5579
15	C	0.1664	3.3170	1.2475
16	H	0.2180	1.8142	2.7554
17	C	-0.2009	2.5476	-1.0126
18	H	-0.4884	0.4612	-1.2736
19	C	0.0370	3.6329	-0.1308

20	H	-0.3681	2.7288	-2.0678
21	C	-0.2009	-6.6673	-0.4154
22	H	-1.2674	-6.6188	-0.1603
23	H	0.3818	-6.8358	0.4995
24	N	0.1329	4.9349	-0.5890
25	C	-0.2023	5.2342	-1.9734
26	C	0.1531	6.0311	0.3686
27	H	-1.2509	5.0000	-2.2140
28	H	0.4390	4.6710	-2.6612
29	H	-0.7580	6.0679	0.9856
30	H	1.0160	5.9503	1.0398
31	H	0.3121	4.1035	1.9783
32	H	-0.0322	-7.4934	-1.1084
33	H	0.2415	6.9757	-0.1701
34	H	-0.0358	6.2961	-2.1612
35	H	-0.2534	-0.4637	2.4367
36	O	0.2261	-5.4921	-1.0967

FLUOar3m2_rad_MeButa
Energy (POTENTIAL) = -1345.9654743 Eh

	Atom	X	Y	Z
1	C	0.0360	-0.0037	1.1932
2	C	0.0660	-1.2587	0.5131
3	C	0.6912	-1.4212	-0.7711
4	C	-0.5319	-2.4488	1.0711
5	C	0.7000	-2.6164	-1.4329
6	H	1.2241	-0.5800	-1.2004
7	C	-0.5412	-3.6438	0.3976
8	H	-1.0385	-2.3811	2.0269
9	C	0.0615	-3.7771	-0.8842
10	H	1.2442	-2.6968	-2.3672
11	H	-1.0536	-4.4861	0.8468
12	C	0.0092	0.0100	2.6688
13	C	-0.8012	0.9124	3.3773
14	C	0.7841	-0.8867	3.4210
15	C	-0.8339	0.9145	4.7650
16	H	-1.4264	1.6058	2.8240
17	C	0.7533	-0.8761	4.8101
18	H	1.4276	-1.5905	2.9031
19	C	-0.0542	0.0239	5.5099
20	H	-1.4825	1.6170	5.2818
21	H	1.3707	-1.5799	5.3619
22	C	0.0311	1.2396	0.4872
23	C	0.6009	2.4377	1.0404
24	C	-0.5289	1.3697	-0.8277
25	C	0.6177	3.6259	0.3511
26	H	1.0538	2.3975	2.0244
27	C	-0.5305	2.5584	-1.5066
28	H	-0.9958	0.5048	-1.2850
29	C	0.0475	3.7376	-0.9455
30	H	-0.9650	2.5920	-2.4991
31	C	-0.3822	-6.1919	-0.9253
32	C	-0.2366	-4.9732	-3.0060
33	C	0.1191	-7.4209	-1.6688
34	H	-1.4837	-6.2088	-0.8876
35	H	-0.0021	-6.2292	0.0975
36	C	0.2641	-6.2398	-3.6765
37	H	-1.3298	-4.9026	-3.1278
38	H	0.2102	-4.1074	-3.4937
39	H	-0.3165	-8.3241	-1.2370
40	H	1.2145	-7.4801	-1.5881
41	H	-0.0684	-6.2752	-4.7158
42	H	1.3636	-6.2669	-3.6521
43	N	0.0992	-4.9835	-1.5805
44	O	-0.2574	-7.3954	-3.0369
45	N	0.0860	4.9190	-1.6868
46	C	-1.1361	5.3480	-2.3705
47	C	0.8320	6.0551	-1.1623
48	C	-0.8124	6.3788	-3.4370
49	H	-1.8357	5.7855	-1.6397
50	H	-1.6295	4.4962	-2.8373
51	C	1.0865	7.0665	-2.2695
52	H	0.2856	6.5420	-0.3379
53	H	1.7968	5.7143	-0.7810
54	H	-1.7317	6.7662	-3.8802
55	H	-0.2003	5.9199	-4.2275
56	O	-0.1223	7.4885	-2.8817
57	H	1.5632	7.9591	-1.8597
58	H	1.7497	6.6248	-3.0278
59	H	1.0666	4.4901	0.8251
60	C	-0.0661	0.0556	7.0152
61	H	0.6307	0.8115	7.3944
62	H	-1.0585	0.3053	7.4002
63	H	0.2324	-0.9075	7.4370

UVrefdiarcn_rad_cam
Energy (POTENTIAL) = -823.0897583 Eh

	Atom	X	Y	Z
1	C	-0.0235	-0.0204	1.2104
2	C	0.0380	-1.3139	0.5459
3	C	0.6099	-1.4701	-0.7432
4	C	-0.4298	-2.4829	1.1887
5	C	0.6798	-2.7092	-1.3532
6	H	1.0347	-0.6106	-1.2498
7	C	-0.3658	-3.7321	0.5830
8	H	-0.8668	-2.4069	2.1800
9	C	0.1857	-3.8550	-0.7028
10	H	1.1307	-2.8234	-2.3339
11	H	-0.7486	-4.5965	1.1122
12	C	-0.0544	1.2738	0.5449
13	C	0.3839	2.4421	1.2092
14	C	-0.5672	1.4313	-0.7687
15	C	0.3479	3.6918	0.6025
16	H	0.7753	2.3651	2.2194
17	C	-0.6089	2.6710	-1.3800
18	H	-0.9693	0.5727	-1.2948
19	C	-0.1446	3.8162	-0.7070
20	H	-1.0150	2.7860	-2.3801
21	C	-0.1714	-6.2220	-0.7789
22	H	-1.2461	-6.1677	-0.5683
23	H	0.3736	-6.4341	0.1487
24	H	0.7066	4.5555	1.1493
25	H	0.0183	-7.0156	-1.5025
26	O	0.2976	-5.0187	-1.3910
27	O	-0.2249	4.9804	-1.3984
28	C	0.2155	6.1834	-0.7648
29	H	0.0582	6.9774	-1.4956
30	H	1.2797	6.1294	-0.5062
31	H	-0.3707	6.3944	0.1375
32	C	-0.0561	-0.0209	2.6238
33	N	-0.0826	-0.0214	3.7948

SP_mln_rad

Energy (POTENTIAL) = -263.7445364 Eh

	Atom	X	Y	Z
1	C	-0.0240	0.2990	0.0001
2	C	-1.1996	-0.4690	0.0001
3	N	-2.1915	-1.0912	0.0001
4	C	1.2051	-0.3873	-0.0000
5	C	-0.0614	1.8030	0.0002
6	H	0.4557	2.1922	0.8855
7	H	0.4582	2.1924	-0.8834
8	H	-1.0906	2.1645	-0.0011
9	N	2.2388	-0.9349	0.0000

SP_ar2dmnomemln_PMMA

Energy (POTENTIAL) = -1014.5170403 Eh

	Atom	X	Y	Z
1	C	-0.9930	-0.3804	0.4422
2	C	-0.4729	1.0400	0.2834
3	C	-0.1201	1.7574	1.4340
4	C	0.3974	3.0465	1.3761
5	C	0.5914	3.6985	0.1353
6	C	0.2127	2.9838	-1.0260
7	C	-0.2955	1.6913	-0.9464
8	H	-0.5711	1.2015	-1.8745
9	H	0.3120	3.4343	-2.0058
10	H	0.6445	3.5452	2.3054
11	H	-0.2509	1.2962	2.4108
12	C	-2.3648	-0.6447	-0.3333
13	C	-2.1623	-0.7814	-1.7898
14	N	-2.0152	-0.8658	-2.9374
15	C	0.0350	-1.4729	0.1687
16	C	0.9074	-1.4336	-0.9315
17	C	1.8146	-2.4581	-1.1643
18	C	1.8813	-3.5582	-0.2952
19	C	1.0270	-3.6112	0.8122
20	C	0.1189	-2.5722	1.0289
21	H	-0.5409	-2.6300	1.8908
22	H	1.0569	-4.4422	1.5069
23	H	2.4881	-2.4267	-2.0150
24	H	0.8893	-0.5888	-1.6108
25	N	1.1276	4.9723	0.0594
26	C	1.1093	5.6788	-1.2127
27	C	1.3064	5.7444	1.2795
28	H	0.0888	5.8259	-1.5996
29	H	1.6859	5.1368	-1.9716
30	H	0.3585	5.9194	1.8121
31	H	1.9945	5.2386	1.9672
32	C	2.9084	-5.6540	0.2281
33	H	3.1924	-5.3787	1.2521
34	H	1.9668	-6.2173	0.2526
35	H	1.7433	6.7126	1.0299
36	H	1.5723	6.6587	-1.0853
37	H	-1.2941	-0.4824	1.4908
38	H	3.6912	-6.2759	-0.2091
39	C	-2.9225	-1.9265	0.1476
40	N	-3.3758	-2.9075	0.5695
41	C	-3.4006	0.4784	-0.0538
42	H	-3.5292	0.5825	1.0269
43	H	-3.0330	1.4223	-0.4583
44	H	-4.3645	0.2379	-0.5086
45	O	2.8072	-4.5076	-0.6109

ar2dmnome_rad_PMMA

Energy (POTENTIAL) = -750.505563601 Eh

	Atom	X	Y	Z
1	C	-0.1239	-0.4162	1.3594
2	C	-0.0301	-1.6912	0.7078
3	C	0.5040	-1.9027	-0.5949
4	C	-0.4452	-2.8532	1.4082
5	C	0.5737	-3.1668	-1.1550
6	H	0.9109	-1.0657	-1.1503
7	C	-0.3818	-4.1263	0.8526
8	H	-0.8412	-2.7422	2.4151
9	C	0.1218	-4.2934	-0.4466
10	H	0.9943	-3.3149	-2.1454
11	H	-0.7235	-4.9757	1.4332
12	C	-0.0734	0.9043	0.8150
13	C	0.1128	2.0089	1.6916
14	C	-0.2534	1.2386	-0.5548
15	C	0.1709	3.3167	1.2469
16	H	0.2238	1.8155	2.7566
17	C	-0.2017	2.5453	-1.0112
18	H	-0.4926	0.4585	-1.2687
19	C	0.0392	3.6310	-0.1312
20	H	-0.3709	2.7254	-2.0662
21	C	-0.2021	-6.6639	-0.4202
22	H	-1.2698	-6.6145	-0.1694
23	H	0.3763	-6.8350	0.4971
24	N	0.1359	4.9330	-0.5914
25	C	-0.2071	5.2309	-1.9739
26	C	0.1542	6.0297	0.3653
27	H	-1.2575	4.9979	-2.2086
28	H	0.4296	4.6660	-2.6645
29	H	-0.7575	6.0669	0.9817
30	H	1.0166	5.9498	1.0374
31	H	0.3192	4.1039	1.9766
32	H	-0.0317	-7.4894	-1.1136
33	H	0.2430	6.9741	-0.1739
34	H	-0.0400	6.2924	-2.1643
35	H	-0.2553	-0.4634	2.4401
36	O	0.2294	-5.4891	-1.0976

FLUOar2dmnome_rad_MeButa

Energy (POTENTIAL) = -750.7007277 Eh

	Atom	X	Y	Z
1	C	-0.1713	-0.4178	1.3279
2	C	-0.0769	-1.6705	0.6850
3	C	0.3992	-1.8903	-0.6548
4	C	-0.4091	-2.8612	1.4231
5	C	0.4841	-3.1500	-1.1948
6	H	0.7448	-1.0521	-1.2450
7	C	-0.3263	-4.1240	0.8798
8	H	-0.7506	-2.7512	2.4483
9	C	0.1102	-4.2887	-0.4495
10	H	0.8556	-3.2957	-2.2042
11	H	-0.6018	-4.9791	1.4851
12	C	-0.1271	0.8906	0.7975
13	C	0.0378	2.0069	1.6939
14	C	-0.3110	1.2469	-0.5892
15	C	0.0923	3.3007	1.2666
16	H	0.1339	1.7999	2.7560
17	C	-0.2555	2.5461	-1.0215

18	H	-0.5501	0.4774	-1.3105
19	C	-0.0226	3.6289	-0.1259
20	H	-0.4142	2.7409	-2.0751
21	C	-0.1293	-6.6658	-0.3838
22	H	-1.1798	-6.6476	-0.0732
23	H	0.5096	-6.8017	0.4957
24	N	0.0866	4.9253	-0.5675
25	C	-0.1271	5.2389	-1.9688
26	C	0.2125	6.0167	0.3826
27	H	-1.1546	5.0141	-2.2816
28	H	0.5598	4.6732	-2.6053
29	H	-0.6720	6.0972	1.0264
30	H	1.0934	5.8853	1.0178
31	H	0.2251	4.0846	2.0016
32	H	0.0287	-7.4915	-1.0766
33	H	0.3276	6.9527	-0.1608
34	H	0.0580	6.2989	-2.1321
35	H	-0.3000	-0.4668	2.4082
36	O	0.2170	-5.4859	-1.0924

ar2dmnome_rad_MeButa
Energy (POTENTIAL) = -750.506478289 Eh

	Atom	X	Y	Z
1	C	-0.1234	-0.4163	1.3558
2	C	-0.0304	-1.6917	0.7047
3	C	0.4985	-1.9045	-0.6001
4	C	-0.4411	-2.8537	1.4083
5	C	0.5674	-3.1694	-1.1588
6	H	0.9019	-1.0682	-1.1589
7	C	-0.3785	-4.1275	0.8542
8	H	-0.8330	-2.7420	2.4167
9	C	0.1198	-4.2958	-0.4470
10	H	0.9838	-3.3178	-2.1509
11	H	-0.7168	-4.9764	1.4374
12	C	-0.0741	0.9044	0.8122
13	C	0.1090	2.0085	1.6904
14	C	-0.2519	1.2403	-0.5579
15	C	0.1664	3.3170	1.2475
16	H	0.2180	1.8142	2.7554
17	C	-0.2009	2.5476	-1.0126
18	H	-0.4884	0.4612	-1.2736
19	C	0.0370	3.6329	-0.1308
20	H	-0.3681	2.7288	-2.0678
21	C	-0.2009	-6.6673	-0.4154
22	H	-1.2674	-6.6188	-0.1603
23	H	0.3819	-6.8358	0.4995
24	N	0.1329	4.9349	-0.5890
25	C	-0.2023	5.2342	-1.9734
26	C	0.1531	6.0311	0.3686
27	H	-1.2509	5.0000	-2.2140
28	H	0.4390	4.6710	-2.6612
29	H	-0.7580	6.0679	0.9856
30	H	1.0160	5.9503	1.0398
31	H	0.3121	4.1035	1.9783
32	H	-0.0322	-7.4934	-1.1084
33	H	0.2415	6.9757	-0.1701
34	H	-0.0358	6.2961	-2.1612
35	H	-0.2534	-0.4637	2.4367
36	O	0.2261	-5.4921	-1.0967

ar2dmnome_rad
Energy (POTENTIAL) = -750.508478768 Eh

	Atom	X	Y	Z
1	C	-0.1206	-0.4168	1.3467
2	C	-0.0296	-1.6933	0.6969
3	C	0.4852	-1.9092	-0.6132
4	C	-0.4292	-2.8546	1.4089
5	C	0.5510	-3.1762	-1.1684
6	H	0.8796	-1.0746	-1.1809
7	C	-0.3693	-4.1303	0.8586
8	H	-0.8097	-2.7411	2.4215
9	C	0.1149	-4.3018	-0.4479
10	H	0.9562	-3.3257	-2.1651
11	H	-0.6983	-4.9780	1.4487
12	C	-0.0748	0.9047	0.8049
13	C	0.0998	2.0078	1.6874
14	C	-0.2468	1.2446	-0.5655
15	C	0.1545	3.3178	1.2489
16	H	0.2035	1.8110	2.7525
17	C	-0.1979	2.5534	-1.0163
18	H	-0.4764	0.4677	-1.2859
19	C	0.0316	3.6377	-0.1299
20	H	-0.3600	2.7370	-2.0718
21	C	-0.1996	-6.6753	-0.4038
22	H	-1.2631	-6.6279	-0.1370
23	H	0.3941	-6.8386	0.5046
24	N	0.1255	4.9396	-0.5831
25	C	-0.1891	5.2423	-1.9723
26	C	0.1478	6.0346	0.3768
27	H	-1.2332	5.0057	-2.2285
28	H	0.4638	4.6827	-2.6521
29	H	-0.7625	6.0695	0.9944
30	H	1.0114	5.9524	1.0471
31	H	0.2929	4.1027	1.9829
32	H	-0.0367	-7.5028	-1.0961
33	H	0.2347	6.9799	-0.1603
34	H	-0.0234	6.3051	-2.1539
35	H	-0.2465	-0.4645	2.4281
36	O	0.2171	-5.4999	-1.0943

SP_ar2dmn2_cat_MeButa
Energy (POTENTIAL) = -770.0141906 Eh

	Atom	X	Y	Z
1	C	-0.0302	0.0010	1.3957
2	C	0.0103	-1.2853	0.8189
3	C	0.3341	-1.5734	-0.5420
4	C	-0.2464	-2.4105	1.6614
5	C	0.3411	-2.8550	-1.0294
6	H	0.6452	-0.7731	-1.2021
7	C	-0.2636	-3.6965	1.1858
8	H	-0.4638	-2.2370	2.7120
9	C	0.0142	-3.9696	-0.1907
10	H	0.6279	-3.0170	-2.0601
11	H	-0.4877	-4.5051	1.8690
12	C	-0.0679	1.2879	0.8200
13	C	0.1849	2.4122	1.6649
14	C	-0.3852	1.5774	-0.5421
15	C	0.2046	3.6987	1.1906
16	H	0.3971	2.2377	2.7163
17	C	-0.3897	2.8595	-1.0283
18	H	-0.6932	0.7779	-1.2045
19	C	-0.0665	3.9732	-0.1870

20	H	-0.6716	3.0225	-2.0602
21	C	-0.2864	-6.3644	0.2077
22	C	0.2796	-5.4917	-2.0896
23	H	-1.2842	-6.2775	0.6518
24	H	0.4553	-6.4306	1.0116
25	H	0.1571	-6.5556	-2.2866
26	H	-0.4116	-4.9407	-2.7361
27	N	-0.0053	-5.2310	-0.6766
28	N	-0.0443	5.2350	-0.6716
29	C	-0.3227	5.4972	-2.0857
30	C	0.2338	6.3674	0.2150
31	H	-1.3492	5.2148	-2.3463
32	H	0.3716	4.9469	-2.7295
33	H	0.1981	7.2891	-0.3634
34	H	-0.5113	6.4334	1.0157
35	H	0.4254	4.5066	1.8756
36	H	-0.2475	-7.2856	-0.3714
37	H	1.3074	-5.2090	-2.3452
38	H	1.2296	6.2793	0.6633
39	H	-0.1992	6.5613	-2.2810
40	H	-0.0328	0.0005	2.4858

ar2dmn2obn_meoh_MeButa

Energy (POTENTIAL) = -1271.23639342 Eh

	Atom	X	Y	Z
1	C	-0.2976	-0.3292	-1.1005
2	C	-1.7087	-0.3803	-0.5459
3	C	-2.7249	0.3279	-1.2018
4	C	-2.0430	-1.0181	0.6551
5	C	-4.0189	0.3893	-0.6987
6	H	-2.4917	0.8686	-2.1161
7	C	-3.3324	-0.9714	1.1751
8	H	-1.2827	-1.5754	1.1950
9	C	-4.3614	-0.2580	0.5139
10	H	-4.7642	0.9452	-1.2544
11	H	-3.5342	-1.4943	2.1021
12	C	0.5940	-1.5249	-0.7901
13	C	1.9579	-1.3676	-0.5195
14	C	0.1026	-2.8349	-0.8559
15	C	2.7931	-2.4596	-0.2986
16	H	2.3782	-0.3678	-0.4765
17	C	0.9235	-3.9391	-0.6484
18	H	-0.9503	-3.0016	-1.0660
19	C	2.3016	-3.7837	-0.3668
20	H	3.8366	-2.2710	-0.0764
21	H	0.4807	-4.9264	-0.7030
22	N	3.1343	-4.8807	-0.1791
23	C	4.4661	-4.6660	0.3674
24	C	2.5362	-6.1963	-0.0058
25	H	4.4466	-4.1890	1.3606
26	H	5.0662	-4.0363	-0.2993
27	H	1.8614	-6.2467	0.8639
28	H	1.9652	-6.4865	-0.8952
29	N	-5.6429	-0.1872	1.0342
30	C	-6.7118	0.3779	0.2240
31	C	-6.0030	-1.0352	2.1605
32	H	-6.8656	-0.1734	-0.7168
33	H	-6.5042	1.4250	-0.0264
34	H	-5.8889	-2.1072	1.9356
35	H	-5.3883	-0.8056	3.0391
36	O	0.3660	0.9170	-0.7438
37	C	0.3776	1.2456	0.6604
38	H	-0.6278	1.5608	0.9662
39	H	0.6457	0.3595	1.2470
40	C	1.3837	2.3469	0.8582
41	C	2.6189	2.0958	1.4624
42	C	1.1100	3.6451	0.4039
43	C	3.5581	3.1180	1.6176
44	H	2.8505	1.0922	1.8125
45	C	2.0521	4.6601	0.5534
46	H	0.1644	3.8492	-0.0921
47	C	3.2904	4.4166	1.1683
48	H	4.5127	2.9019	2.0920
49	H	1.8248	5.6599	0.1896
50	C	4.2905	5.5330	1.3574
51	H	4.3218	6.1939	0.4839
52	H	4.0277	6.1555	2.2230
53	H	5.2999	5.1443	1.5273
54	C	0.1603	3.3640	-3.0754
55	H	-0.2450	4.1579	-3.7113
56	H	1.0217	3.7702	-2.5233
57	H	0.5234	2.5545	-3.7279
58	O	-0.8781	2.9342	-2.2064
59	H	-0.4918	2.2253	-1.6491
60	H	-0.3705	-0.2301	-2.1911
61	H	4.9758	-5.6269	0.4571
62	H	3.3283	-6.9349	0.1299
63	H	-7.6430	0.3537	0.7921
64	H	-7.0441	-0.8504	2.4301

ar2dmn2mln_PMMA

Energy (POTENTIAL) = -1033.69875596 Eh

	Atom	X	Y	Z
1	C	-1.1928	0.0402	0.4682
2	C	-0.4204	1.3437	0.3345
3	C	-0.0362	2.0191	1.5001
4	C	0.7030	3.1964	1.4676
5	C	1.1052	3.7703	0.2385
6	C	0.6965	3.1024	-0.9400
7	C	-0.0366	1.9210	-0.8849
8	H	-0.3224	1.4613	-1.8252
9	H	0.9489	3.5022	-1.9145
10	H	0.9608	3.6691	2.4074
11	H	-0.3213	1.6143	2.4690
12	C	-2.5244	0.0011	-0.4156
13	C	-2.2340	-0.2137	-1.8476
14	N	-2.0148	-0.3548	-2.9781
15	C	-0.3566	-1.2209	0.2939
16	C	0.6061	-1.3662	-0.7131
17	C	1.3351	-2.5402	-0.8646
18	C	1.1312	-3.6470	-0.0056
19	C	0.1764	-3.4892	1.0263
20	C	-0.5435	-2.3070	1.1589
21	H	-1.2727	-2.2317	1.9623
22	H	-0.0090	-4.2878	1.7340
23	H	2.0715	-2.5886	-1.6574
24	H	0.8103	-0.5440	-1.3907
25	N	1.8647	4.9279	0.1912
26	C	2.0607	5.5986	-1.0854
27	C	2.0677	5.6944	1.4112
28	H	1.1126	5.9165	-1.5470
29	H	2.5828	4.9465	-1.7953

30	H	1.1221	6.0473	1.8522
31	H	2.5931	5.0978	2.1663
32	N	1.8334	-4.8301	-0.1686
33	C	2.9512	-4.8727	-1.0988
34	C	1.7536	-5.8526	0.8631
35	H	3.7487	-4.1597	-0.8359
36	H	2.6203	-4.6509	-2.1202
37	H	2.1122	-5.4958	1.8416
38	H	0.7230	-6.2057	0.9873
39	H	2.6885	6.5640	1.1894
40	H	2.6819	6.4828	-0.9336
41	H	-1.5907	0.0215	1.4893
42	H	2.3610	-6.7094	0.5667
43	H	3.3767	-5.8775	-1.1017
44	C	-3.3407	-1.1455	0.0360
45	N	-4.0001	-2.0137	0.4325
46	C	-3.3634	1.2969	-0.2425
47	H	-3.5570	1.4542	0.8221
48	H	-2.8026	2.1491	-0.6286
49	H	-4.3155	1.2168	-0.7725

ar2dmnomemln_PMMA
Energy (POTENTIAL) = -1014.24987001 Eh

	Atom	X	Y	Z
1	C	-0.9930	-0.3804	0.4422
2	C	-0.4729	1.0400	0.2834
3	C	-0.1201	1.7574	1.4340
4	C	0.3974	3.0465	1.3761
5	C	0.5914	3.6985	0.1353
6	C	0.2127	2.9838	-1.0260
7	C	-0.2955	1.6913	-0.9464
8	H	-0.5711	1.2015	-1.8745
9	H	0.3120	3.4343	-2.0058
10	H	0.6445	3.5452	2.3054
11	H	-0.2509	1.2962	2.4108
12	C	-2.3648	-0.6447	-0.3333
13	C	-2.1623	-0.7814	-1.7898
14	N	-2.0152	-0.8658	-2.9374
15	C	0.0350	-1.4729	0.1687
16	C	0.9074	-1.4336	-0.9315
17	C	1.8146	-2.4581	-1.1643
18	C	1.8813	-3.5582	-0.2952
19	C	1.0270	-3.6112	0.8122
20	C	0.1189	-2.5722	1.0289
21	H	-0.5409	-2.6300	1.8908
22	H	1.0569	-4.4422	1.5069
23	H	2.4881	-2.4267	-2.0150
24	H	0.8893	-0.5888	-1.6108
25	N	1.1276	4.9723	0.0594
26	C	1.1093	5.6788	-1.2127
27	C	1.3064	5.7444	1.2795
28	H	0.0888	5.8259	-1.5996
29	H	1.6859	5.1368	-1.9716
30	H	0.3585	5.9194	1.8121
31	H	1.9945	5.2386	1.9672
32	C	2.9084	-5.6540	0.2281
33	H	3.1924	-5.3787	1.2521
34	H	1.9668	-6.2173	0.2526
35	H	1.7433	6.7126	1.0299
36	H	1.5723	6.6587	-1.0853
37	H	-1.2941	-0.4824	1.4908
38	H	3.6912	-6.2759	-0.2091
39	C	-2.9225	-1.9265	0.1476
40	N	-3.3758	-2.9075	0.5695
41	C	-3.4006	0.4784	-0.0538
42	H	-3.5292	0.5825	1.0269
43	H	-3.0330	1.4223	-0.4583
44	H	-4.3645	0.2379	-0.5086
45	O	2.8072	-4.5076	-0.6109

SP_ar2dmn2obn_meoh_PMMA
Energy (POTENTIAL) = -1271.57878 Eh

	Atom	X	Y	Z
1	C	-0.2970	-0.3315	-1.0996
2	C	-1.7075	-0.3814	-0.5434
3	C	-2.7217	0.3348	-1.1937
4	C	-2.0425	-1.0248	0.6544
5	C	-4.0146	0.3980	-0.6885
6	H	-2.4875	0.8811	-2.1044
7	C	-3.3312	-0.9765	1.1759
8	H	-1.2834	-1.5875	1.1902
9	C	-4.3580	-0.2554	0.5203
10	H	-4.7582	0.9608	-1.2395
11	H	-3.5338	-1.5039	2.1002
12	C	0.5943	-1.5279	-0.7910
13	C	1.9580	-1.3710	-0.5205
14	C	0.1027	-2.8376	-0.8584
15	C	2.7930	-2.4633	-0.3008
16	H	2.3782	-0.3711	-0.4773
17	C	0.9235	-3.9421	-0.6521
18	H	-0.9503	-3.0038	-1.0689
19	C	2.3013	-3.7870	-0.3705
20	H	3.8367	-2.2751	-0.0791
21	H	0.4808	-4.9293	-0.7082
22	N	3.1342	-4.8847	-0.1838
23	C	4.4658	-4.6706	0.3624
24	C	2.5360	-6.1999	-0.0118
25	H	4.4471	-4.1942	1.3560
26	H	5.0658	-4.0405	-0.3042
27	H	1.8607	-6.2513	0.8576
28	H	1.9652	-6.4893	-0.9017
29	N	-5.6393	-0.1831	1.0425
30	C	-6.7064	0.3899	0.2362
31	C	-6.0016	-1.0385	2.1621
32	H	-6.8633	-0.1556	-0.7075
33	H	-6.4952	1.4376	-0.0087
34	H	-5.8912	-2.1093	1.9291
35	H	-5.3857	-0.8177	3.0422
36	O	0.3679	0.9136	-0.7431
37	C	0.3783	1.2432	0.6605
38	H	-0.6278	1.5568	0.9658
39	H	0.6478	0.3578	1.2479
40	C	1.3822	2.3465	0.8579
41	C	2.6171	2.0985	1.4638
42	C	1.1065	3.6435	0.4015
43	C	3.5542	3.1226	1.6187
44	H	2.8501	1.0957	1.8152
45	C	2.0466	4.6603	0.5508
46	H	0.1612	3.8451	-0.0961
47	C	3.2845	4.4199	1.1674
48	H	4.5087	2.9090	2.0945
49	H	1.8178	5.6591	0.1854

50	C	4.2824	5.5385	1.3560
51	H	4.3169	6.1954	0.4795
52	H	4.0150	6.1648	2.2174
53	H	5.2915	5.1520	1.5319
54	C	0.1616	3.3635	-3.0793
55	H	-0.2474	4.1557	-3.7149
56	H	1.0220	3.7730	-2.5280
57	H	0.5277	2.5556	-3.7323
58	O	-0.8742	2.9299	-2.2098
59	H	-0.4853	2.2230	-1.6521
60	H	-0.3710	-0.2320	-2.1901
61	H	4.9756	-5.6316	0.4511
62	H	3.3278	-6.9389	0.1233
63	H	-7.6374	0.3664	0.8050
64	H	-7.0419	-0.8523	2.4338

ar2dmn2_cat_MeButa
Energy (POTENTIAL) = -769.824773455 Eh

	Atom	X	Y	Z
1	C	-0.0302	0.0010	1.3957
2	C	0.0103	-1.2853	0.8189
3	C	0.3341	-1.5734	-0.5420
4	C	-0.2464	-2.4105	1.6614
5	C	0.3411	-2.8550	-1.0294
6	H	0.6452	-0.7731	-1.2021
7	C	-0.2636	-3.6965	1.1858
8	H	-0.4638	-2.2370	2.7120
9	C	0.0141	-3.9696	-0.1907
10	H	0.6279	-3.0170	-2.0601
11	H	-0.4877	-4.5051	1.8690
12	C	-0.0679	1.2879	0.8200
13	C	0.1849	2.4122	1.6649
14	C	-0.3852	1.5774	-0.5421
15	C	0.2046	3.6987	1.1906
16	H	0.3971	2.2377	2.7163
17	C	-0.3897	2.8595	-1.0283
18	H	-0.6932	0.7779	-1.2045
19	C	-0.0665	3.9732	-0.1870
20	H	-0.6716	3.0225	-2.0602
21	C	-0.2864	-6.3644	0.2077
22	C	0.2796	-5.4917	-2.0896
23	H	-1.2842	-6.2775	0.6518
24	H	0.4553	-6.4306	1.0116
25	H	0.1571	-6.5556	-2.2866
26	H	-0.4116	-4.9407	-2.7361
27	N	-0.0053	-5.2310	-0.6766
28	N	-0.0443	5.2350	-0.6716
29	C	-0.3227	5.4972	-2.0857
30	C	0.2338	6.3674	0.2150
31	H	-1.3492	5.2148	-2.3463
32	H	0.3716	4.9469	-2.7295
33	H	0.1981	7.2891	-0.3634
34	H	-0.5113	6.4334	1.0157
35	H	0.4254	4.5066	1.8756
36	H	-0.2475	-7.2856	-0.3714
37	H	1.3074	-5.2090	-2.3452
38	H	1.2296	6.2793	0.6633
39	H	-0.1992	6.5613	-2.2810
40	H	-0.0328	0.0005	2.4858

ar2dmnome_cat_MeButa
Energy (POTENTIAL) = -750.366029075 Eh

	Atom	X	Y	Z
1	C	-0.0867	-0.3979	1.3057
2	C	0.0191	-1.6694	0.6699
3	C	0.5102	-1.8844	-0.6499
4	C	-0.3551	-2.8138	1.4226
5	C	0.5656	-3.1495	-1.1876
6	H	0.9047	-1.0553	-1.2242
7	C	-0.3238	-4.0879	0.8857
8	H	-0.7036	-2.6812	2.4430
9	C	0.1326	-4.2666	-0.4359
10	H	0.9595	-3.3224	-2.1833
11	H	-0.6396	-4.9315	1.4866
12	C	-0.1110	0.8980	0.7826
13	C	0.0175	1.9989	1.6938
14	C	-0.2966	1.2318	-0.5997
15	C	0.0521	3.2982	1.2709
16	H	0.1214	1.7874	2.7544
17	C	-0.2859	2.5269	-1.0357
18	H	-0.5130	0.4490	-1.3160
19	C	-0.0774	3.6167	-0.1222
20	H	-0.4637	2.7252	-2.0843
21	C	-0.1750	-6.6432	-0.3692
22	H	-1.2325	-6.5987	-0.0880
23	H	0.4443	-6.8017	0.5198
24	N	-0.0318	4.8891	-0.5558
25	C	-0.1732	5.2026	-1.9829
26	C	0.1524	5.9957	0.3907
27	H	-1.1548	4.8917	-2.3562
28	H	0.6077	4.7078	-2.5689
29	H	-0.6723	6.0355	1.1099
30	H	1.0973	5.8892	0.9334
31	H	0.1736	4.0862	2.0020
32	H	-0.0159	-7.4544	-1.0790
33	H	0.1756	6.9339	-0.1601
34	H	-0.0779	6.2780	-2.1204
35	H	-0.1668	-0.4502	2.3912
36	O	0.2201	-5.4484	-1.0602

ar2dmn2_cat_PMMA
Energy (POTENTIAL) = -769.820566083 Eh

	Atom	X	Y	Z
1	C	-0.0274	0.0010	1.3953
2	C	0.0112	-1.2854	0.8186
3	C	0.3339	-1.5738	-0.5425
4	C	-0.2459	-2.4103	1.6612
5	C	0.3399	-2.8554	-1.0298
6	H	0.6451	-0.7736	-1.2026
7	C	-0.2642	-3.6963	1.1856
8	H	-0.4626	-2.2368	2.7119
9	C	0.0132	-3.9697	-0.1908
10	H	0.6263	-3.0176	-2.0607
11	H	-0.4884	-4.5048	1.8690
12	C	-0.0650	1.2879	0.8197
13	C	0.1907	2.4121	1.6638
14	C	-0.3852	1.5777	-0.5416
15	C	0.2099	3.6986	1.1895
16	H	0.4057	2.2376	2.7147
17	C	-0.3905	2.8597	-1.0278
18	H	-0.6953	0.7781	-1.2031
19	C	-0.0652	3.9733	-0.1871

20	H	-0.6749	3.0229	-2.0590
21	C	-0.2887	-6.3645	0.2081
22	C	0.2785	-5.4926	-2.0893
23	H	-1.2867	-6.2769	0.6517
24	H	0.4526	-6.4311	1.0124
25	H	0.1576	-6.5568	-2.2858
26	H	-0.4137	-4.9431	-2.7361
27	N	-0.0065	-5.2314	-0.6763
28	N	-0.0448	5.2354	-0.6714
29	C	-0.3276	5.4981	-2.0846
30	C	0.2356	6.3676	0.2147
31	H	-1.3545	5.2144	-2.3425
32	H	0.3657	4.9493	-2.7308
33	H	0.1975	7.2897	-0.3631
34	H	-0.5069	6.4331	1.0179
35	H	0.4331	4.5063	1.8739
36	H	-0.2502	-7.2859	-0.3706
37	H	1.3059	-5.2088	-2.3453
38	H	1.2330	6.2800	0.6596
39	H	-0.2064	6.5624	-2.2798
40	H	-0.0283	0.0005	2.4854

ar2dmn2_cat
Energy (POTENTIAL) = -769.832475002 Eh

	Atom	X	Y	Z
1	C	-0.0326	-0.0000	1.4006
2	C	0.0123	-1.2855	0.8222
3	C	0.3374	-1.5708	-0.5391
4	C	-0.2421	-2.4124	1.6632
5	C	0.3453	-2.8515	-1.0288
6	H	0.6482	-0.7693	-1.1978
7	C	-0.2580	-3.6978	1.1854
8	H	-0.4590	-2.2405	2.7140
9	C	0.0179	-3.9678	-0.1924
10	H	0.6325	-3.0114	-2.0598
11	H	-0.4804	-4.5080	1.8673
12	C	-0.0733	1.2868	0.8249
13	C	0.1758	2.4118	1.6702
14	C	-0.3890	1.5754	-0.5380
15	C	0.1956	3.6982	1.1954
16	H	0.3855	2.2374	2.7221
17	C	-0.3930	2.8572	-1.0248
18	H	-0.6957	0.7756	-1.2006
19	C	-0.0707	3.9714	-0.1836
20	H	-0.6730	3.0197	-2.0573
21	C	-0.2856	-6.3632	0.1994
22	C	0.2783	-5.4844	-2.0961
23	H	-1.2813	-6.2742	0.6477
24	H	0.4588	-6.4345	1.0003
25	H	0.1508	-6.5469	-2.2965
26	H	-0.4111	-4.9277	-2.7395
27	N	-0.0040	-5.2275	-0.6818
28	N	-0.0446	5.2323	-0.6699
29	C	-0.3156	5.4925	-2.0858
30	C	0.2326	6.3655	0.2158
31	H	-1.3422	5.2139	-2.3503
32	H	0.3788	4.9371	-2.7250
33	H	0.2029	7.2860	-0.3648
34	H	-0.5163	6.4352	1.0125
35	H	0.4138	4.5067	1.8807
36	H	-0.2520	-7.2822	-0.3834
37	H	1.3069	-5.2049	-2.3518
38	H	1.2257	6.2748	0.6697
39	H	-0.1864	6.5555	-2.2827
40	H	-0.0362	-0.0013	2.4906

ar2dmn2obn
Energy (POTENTIAL) = -1155.49837868 Eh

	Atom	X	Y	Z
1	C	-0.3264	-0.1439	-1.3111
2	C	-1.7533	-0.0854	-0.7947
3	C	-2.7765	0.3808	-1.6291
4	C	-2.1045	-0.4042	0.5244
5	C	-4.0870	0.5243	-1.1847
6	H	-2.5463	0.6360	-2.6618
7	C	-3.4067	-0.2659	0.9932
8	H	-1.3451	-0.7853	1.2016
9	C	-4.4397	0.2160	0.1520
10	H	-4.8360	0.8745	-1.8846
11	H	-3.6168	-0.5379	2.0205
12	C	0.4999	-1.3378	-0.8356
13	C	1.8899	-1.2347	-0.7001
14	C	-0.0688	-2.5969	-0.6082
15	C	2.6778	-2.3217	-0.3326
16	H	2.3655	-0.2753	-0.8773
17	C	0.7024	-3.6994	-0.2484
18	H	-1.1430	-2.7260	-0.7054
19	C	2.1059	-3.5957	-0.1033
20	H	3.7454	-2.1706	-0.2260
21	H	0.1999	-4.6436	-0.0760
22	N	2.8866	-4.6947	0.2350
23	C	4.2728	-4.4780	0.6246
24	C	2.2299	-5.8996	0.7225
25	H	4.3701	-3.8211	1.5038
26	H	4.8450	-4.0321	-0.1968
27	H	1.6343	-5.7213	1.6321
28	H	1.5653	-6.3198	-0.0409
29	N	-5.7339	0.3830	0.6177
30	C	-6.8007	0.6622	-0.3330
31	C	-6.0981	-0.1639	1.9172
32	H	-6.9176	-0.1345	-1.0842
33	H	-6.6174	1.6042	-0.8628
34	H	-5.9548	-1.2544	1.9722
35	H	-5.5069	0.2968	2.7171
36	O	0.3800	1.1010	-1.0984
37	C	0.4580	1.5411	0.2636
38	H	-0.5125	1.9471	0.5801
39	H	0.6972	0.6992	0.9264
40	C	1.5333	2.5924	0.3435
41	C	2.8342	2.2629	0.7409
42	C	1.2651	3.9159	-0.0272
43	C	3.8386	3.2316	0.7717
44	H	3.0626	1.2384	1.0263
45	C	2.2696	4.8827	0.0017
46	H	0.2607	4.1884	-0.3433
47	C	3.5734	4.5580	0.4049
48	H	4.8427	2.9542	1.0848
49	H	2.0404	5.9050	-0.2910
50	C	4.6506	5.6155	0.4698
51	H	4.5275	6.3620	-0.3225
52	H	4.6190	6.1519	1.4275
53	H	5.6496	5.1772	0.3746

54	H	-7.7432	0.7664	0.2067
55	H	-7.1483	0.0549	2.1162
56	H	4.7326	-5.4393	0.8604
57	H	2.9865	-6.6528	0.9489
58	H	-0.3769	-0.1853	-2.4070

mln_an
Energy (POTENTIAL) = -263.822641895 Eh

	Atom	X	Y	Z
1	C	-0.0383	0.2689	-0.0327
2	C	-1.1637	-0.5406	0.1188
3	N	-2.1310	-1.2065	0.2379
4	C	1.2149	-0.3364	-0.1250
5	C	-0.1635	1.7855	0.0015
6	H	-0.0602	2.2155	1.0108
7	H	0.6018	2.2564	-0.6281
8	H	-1.1397	2.1030	-0.3848
9	N	2.2852	-0.8259	-0.2139

ar2dmn2obn_meoh_PMMA
Energy (POTENTIAL) = -1271.23507922 Eh

	Atom	X	Y	Z
1	C	-0.2970	-0.3315	-1.0996
2	C	-1.7075	-0.3814	-0.5434
3	C	-2.7217	0.3348	-1.1937
4	C	-2.0425	-1.0248	0.6544
5	C	-4.0146	0.3980	-0.6885
6	H	-2.4875	0.8811	-2.1044
7	C	-3.3312	-0.9765	1.1759
8	H	-1.2835	-1.5875	1.1902
9	C	-4.3580	-0.2554	0.5203
10	H	-4.7582	0.9608	-1.2395
11	H	-3.5338	-1.5039	2.1002
12	C	0.5943	-1.5279	-0.7910
13	C	1.9580	-1.3710	-0.5205
14	C	0.1027	-2.8376	-0.8585
15	C	2.7930	-2.4633	-0.3008
16	H	2.3782	-0.3711	-0.4773
17	C	0.9235	-3.9421	-0.6521
18	H	-0.9503	-3.0038	-1.0689
19	C	2.3013	-3.7870	-0.3705
20	H	3.8367	-2.2751	-0.0791
21	H	0.4808	-4.9293	-0.7082
22	N	3.1342	-4.8847	-0.1838
23	C	4.4658	-4.6706	0.3624
24	C	2.5360	-6.1999	-0.0118
25	H	4.4471	-4.1942	1.3560
26	H	5.0658	-4.0405	-0.3042
27	H	1.8607	-6.2513	0.8576
28	H	1.9652	-6.4893	-0.9017
29	N	-5.6393	-0.1831	1.0425
30	C	-6.7064	0.3899	0.2362
31	C	-6.0016	-1.0385	2.1621
32	H	-6.8633	-0.1556	-0.7075
33	H	-6.4952	1.4376	-0.0087
34	H	-5.8912	-2.1093	1.9291
35	H	-5.3857	-0.8177	3.0422
36	O	0.3679	0.9136	-0.7431
37	C	0.3783	1.2432	0.6605
38	H	-0.6278	1.5568	0.9658
39	H	0.6478	0.3578	1.2479
40	C	1.3822	2.3465	0.8579
41	C	2.6171	2.0985	1.4638
42	C	1.1065	3.6435	0.4015
43	C	3.5542	3.1226	1.6187
44	H	2.8501	1.0957	1.8152
45	C	2.0466	4.6603	0.5508
46	H	0.1612	3.8451	-0.0961
47	C	3.2845	4.4199	1.1674
48	H	4.5087	2.9090	2.0945
49	H	1.8178	5.6591	0.1854
50	C	4.2824	5.5385	1.3560
51	H	4.3169	6.1954	0.4795
52	H	4.0150	6.1648	2.2174
53	H	5.2915	5.1520	1.5319
54	C	0.1616	3.3635	-3.0793
55	H	-0.2474	4.1557	-3.7149
56	H	1.0220	3.7730	-2.5280
57	H	0.5277	2.5556	-3.7323
58	O	-0.8742	2.9299	-2.2098
59	H	-0.4853	2.2230	-1.6521
60	H	-0.3710	-0.2320	-2.1901
61	H	4.9756	-5.6316	0.4511
62	H	3.3278	-6.9389	0.1233
63	H	-7.6374	0.3664	0.8050
64	H	-7.0419	-0.8523	2.4338

ar2dmnomemln_MeButa
Energy (POTENTIAL) = -1014.25138994 Eh

	Atom	X	Y	Z
1	C	-0.9937	-0.3803	0.4413
2	C	-0.4727	1.0398	0.2827
3	C	-0.1210	1.7569	1.4338
4	C	0.3976	3.0457	1.3765
5	C	0.5940	3.6977	0.1357
6	C	0.2160	2.9831	-1.0261
7	C	-0.2934	1.6910	-0.9470
8	H	-0.5686	1.2018	-1.8755
9	H	0.3163	3.4336	-2.0058
10	H	0.6434	3.5444	2.3061
11	H	-0.2541	1.2963	2.4105
12	C	-2.3662	-0.6434	-0.3339
13	C	-2.1644	-0.7800	-1.7905
14	N	-2.0175	-0.8670	-2.9380
15	C	0.0335	-1.4734	0.1670
16	C	0.9024	-1.4368	-0.9361
17	C	1.8095	-2.4616	-1.1689
18	C	1.8797	-3.5592	-0.2968
19	C	1.0291	-3.6093	0.8138
20	C	0.1211	-2.5701	1.0303
21	H	-0.5350	-2.6250	1.8953
22	H	1.0624	-4.4377	1.5113
23	H	2.4804	-2.4317	-2.0218
24	H	0.8820	-0.5938	-1.6176
25	N	1.1320	4.9706	0.0604
26	C	1.1109	5.6789	-1.2111
27	C	1.3057	5.7442	1.2808
28	H	0.0895	5.8276	-1.5947
29	H	1.6846	5.1370	-1.9721
30	H	0.3552	5.9209	1.8082
31	H	1.9895	5.2382	1.9724
32	C	2.9109	-5.6527	0.2308

33	H	3.1986	-5.3727	1.2523
34	H	1.9700	-6.2167	0.2612
35	H	1.7452	6.7113	1.0321
36	H	1.5755	6.6578	-1.0837
37	H	-1.2946	-0.4825	1.4898
38	H	3.6926	-6.2753	-0.2073
39	C	-2.9232	-1.9266	0.1443
40	N	-3.3753	-2.9100	0.5616
41	C	-3.4017	0.4795	-0.0526
42	H	-3.5289	0.5827	1.0283
43	H	-3.0340	1.4235	-0.4569
44	H	-4.3659	0.2398	-0.5068
45	O	2.8049	-4.5091	-0.6127

SP_ar2dmnome_cat_MeButa
Energy (POTENTIAL) = -750.5529843 Eh

	Atom	X	Y	Z
1	C	-0.0867	-0.3979	1.3057
2	C	0.0191	-1.6694	0.6699
3	C	0.5102	-1.8844	-0.6499
4	C	-0.3551	-2.8138	1.4226
5	C	0.5656	-3.1495	-1.1876
6	H	0.9047	-1.0553	-1.2242
7	C	-0.3238	-4.0879	0.8857
8	H	-0.7036	-2.6812	2.4430
9	C	0.1326	-4.2666	-0.4359
10	H	0.9595	-3.3224	-2.1833
11	H	-0.6396	-4.9315	1.4866
12	C	-0.1110	0.8980	0.7826
13	C	0.0175	1.9989	1.6938
14	C	-0.2966	1.2318	-0.5997
15	C	0.0521	3.2982	1.2709
16	H	0.1214	1.7874	2.7544
17	C	-0.2859	2.5269	-1.0357
18	H	-0.5130	0.4490	-1.3160
19	C	-0.0774	3.6167	-0.1222
20	H	-0.4637	2.7252	-2.0843
21	C	-0.1750	-6.6432	-0.3692
22	H	-1.2325	-6.5987	-0.0880
23	H	0.4443	-6.8017	0.5198
24	N	-0.0318	4.8891	-0.5558
25	C	-0.1732	5.2026	-1.9829
26	C	0.1524	5.9957	0.3907
27	H	-1.1548	4.8917	-2.3562
28	H	0.6077	4.7078	-2.5689
29	H	-0.6723	6.0355	1.1099
30	H	1.0973	5.8892	0.9334
31	H	0.1736	4.0862	2.0020
32	H	-0.0159	-7.4544	-1.0790
33	H	0.1756	6.9339	-0.1601
34	H	-0.0779	6.2780	-2.1204
35	H	-0.1668	-0.4502	2.3912
36	O	0.2200	-5.4484	-1.0602

ar2dmnomeobn_meoh
Energy (POTENTIAL) = -1251.79030039 Eh

	Atom	X	Y	Z
1	C	-0.5318	-0.3845	-1.0877
2	C	-1.9407	-0.5232	-0.5349
3	C	-3.0011	0.0818	-1.2307
4	C	-2.2238	-1.1399	0.6870
5	C	-4.2960	0.0562	-0.7313
6	H	-2.7989	0.6054	-2.1616
7	C	-3.5211	-1.1780	1.2050
8	H	-1.4226	-1.6094	1.2493
9	C	-4.5675	-0.5799	0.4912
10	H	-5.1166	0.5206	-1.2697
11	H	-3.6993	-1.6701	2.1538
12	C	0.4455	-1.5015	-0.7452
13	C	1.7985	-1.2326	-0.5078
14	C	0.0532	-2.8462	-0.7535
15	C	2.7176	-2.2490	-0.2627
16	H	2.1428	-0.2032	-0.5090
17	C	0.9585	-3.8771	-0.5204
18	H	-0.9865	-3.1015	-0.9395
19	C	2.3259	-3.6086	-0.2709
20	H	3.7471	-1.9743	-0.0670
21	H	0.5909	-4.8961	-0.5294
22	N	3.2407	-4.6303	-0.0569
23	C	4.5658	-4.2956	0.4457
24	C	2.7491	-5.9819	0.1721
25	H	4.5326	-3.7792	1.4181
26	H	5.1027	-3.6532	-0.2616
27	H	2.0907	-6.0511	1.0526
28	H	2.1925	-6.3493	-0.6976
29	C	-6.2079	-1.1885	2.1265
30	H	-5.9730	-2.2601	2.1068
31	H	-5.6883	-0.7187	2.9710
32	O	0.0293	0.9166	-0.7609
33	C	0.0400	1.2698	0.6392
34	H	-0.9810	1.5137	0.9576
35	H	0.3852	0.4168	1.2339
36	C	0.9624	2.4474	0.8005
37	C	2.2299	2.2982	1.3719
38	C	0.5772	3.7155	0.3421
39	C	3.0914	3.3912	1.4901
40	H	2.5479	1.3199	1.7250
41	C	1.4423	4.8019	0.4548
42	H	-0.3948	3.8410	-0.1283
43	C	2.7122	4.6607	1.0364
44	H	4.0727	3.2544	1.9388
45	H	1.1288	5.7770	0.0883
46	C	3.6292	5.8515	1.1888
47	H	3.5558	6.5258	0.3284
48	H	3.3687	6.4370	2.0805
49	H	4.6743	5.5428	1.2938
50	C	-0.3988	3.3297	-3.1194
51	H	-0.8814	4.0813	-3.7524
52	H	0.4302	3.8136	-2.5810
53	H	0.0244	2.5515	-3.7733
54	O	-1.3836	2.8140	-2.2328
55	H	-0.9288	2.1451	-1.6778
56	H	-0.6098	-0.3195	-2.1802
57	H	5.1464	-5.2121	0.5625
58	H	3.5982	-6.6502	0.3243
59	H	-7.2844	-1.0556	2.2444
60	O	-5.8702	-0.5592	0.8918

refdiarcn_rad_MeButa
Energy (POTENTIAL) = -823.312921484 Eh

	Atom	X	Y	Z
1	C	-0.0238	-0.0204	1.2098

2	C	0.0376	-1.3138	0.5450
3	C	0.6084	-1.4704	-0.7444
4	C	-0.4289	-2.4824	1.1887
5	C	0.6780	-2.7097	-1.3542
6	H	1.0331	-0.6108	-1.2510
7	C	-0.3651	-3.7317	0.5831
8	H	-0.8635	-2.4055	2.1810
9	C	0.1849	-3.8551	-0.7029
10	H	1.1282	-2.8252	-2.3351
11	H	-0.7464	-4.5962	1.1133
12	C	-0.0543	1.2736	0.5440
13	C	0.3830	2.4415	1.2092
14	C	-0.5660	1.4318	-0.7698
15	C	0.3476	3.6913	0.6024
16	H	0.7720	2.3635	2.2201
17	C	-0.6071	2.6716	-1.3810
18	H	-0.9683	0.5731	-1.2957
19	C	-0.1435	3.8163	-0.7072
20	H	-1.0126	2.7880	-2.3811
21	C	-0.1709	-6.2211	-0.7782
22	H	-1.2458	-6.1678	-0.5664
23	H	0.3747	-6.4330	0.1494
24	H	0.7051	4.5550	1.1502
25	H	0.0183	-7.0156	-1.5011
26	O	0.2965	-5.0195	-1.3912
27	O	-0.2232	4.9811	-1.3988
28	C	0.2154	6.1824	-0.7643
29	H	0.0585	6.9775	-1.4943
30	H	1.2797	6.1296	-0.5043
31	H	-0.3715	6.3931	0.1380
32	C	-0.0566	-0.0209	2.6234
33	N	-0.0837	-0.0213	3.7942

SP_ar2dmn2_cat

Energy (POTENTIAL) = -770.0221086 Eh

	Atom	X	Y	Z
1	C	-0.0326	-0.0000	1.4006
2	C	0.0123	-1.2855	0.8222
3	C	0.3374	-1.5708	-0.5391
4	C	-0.2421	-2.4124	1.6632
5	C	0.3453	-2.8515	-1.0288
6	H	0.6482	-0.7693	-1.1978
7	C	-0.2580	-3.6978	1.1854
8	H	-0.4590	-2.2405	2.7140
9	C	0.0179	-3.9678	-0.1924
10	H	0.6325	-3.0114	-2.0598
11	H	-0.4804	-4.5080	1.8673
12	C	-0.0733	1.2868	0.8249
13	C	0.1758	2.4118	1.6702
14	C	-0.3890	1.5754	-0.5380
15	C	0.1956	3.6982	1.1954
16	H	0.3855	2.2374	2.7221
17	C	-0.3930	2.8572	-1.0248
18	H	-0.6957	0.7756	-1.2006
19	C	-0.0707	3.9714	-0.1836
20	H	-0.6730	3.0197	-2.0573
21	C	-0.2856	-6.3632	0.1994
22	C	0.2783	-5.4844	-2.0961
23	H	-1.2813	-6.2742	0.6477
24	H	0.4588	-6.4345	1.0003
25	H	0.1508	-6.5469	-2.2965
26	H	-0.4111	-4.9277	-2.7395
27	N	-0.0040	-5.2275	-0.6818
28	N	-0.0446	5.2323	-0.6699
29	C	-0.3156	5.4925	-2.0858
30	C	0.2326	6.3655	0.2158
31	H	-1.3422	5.2138	-2.3503
32	H	0.3788	4.9371	-2.7250
33	H	0.2029	7.2860	-0.3648
34	H	-0.5163	6.4352	1.0125
35	H	0.4138	4.5067	1.8807
36	H	-0.2520	-7.2822	-0.3834
37	H	1.3069	-5.2049	-2.3518
38	H	1.2257	6.2748	0.6697
39	H	-0.1864	6.5555	-2.2827
40	H	-0.0362	-0.0013	2.4906

SP_obn_an_PMMA

Energy (POTENTIAL) = -385.6860308 Eh

	Atom	X	Y	Z
1	O	-1.0854	-3.1799	0.2059
2	C	0.0820	-2.5754	0.3626
3	H	0.6062	-2.7347	1.3705
4	H	0.9185	-2.8886	-0.3571
5	C	0.0745	-1.0269	0.2234
6	C	1.2240	-0.2395	0.3809
7	C	-1.1235	-0.3771	-0.0721
8	C	1.1749	1.1477	0.2503
9	H	2.1739	-0.7266	0.6091
10	C	-1.1794	1.0145	-0.2049
11	H	-1.9967	-1.0144	-0.1911
12	C	-0.0339	1.8028	-0.0441
13	H	2.0827	1.7384	0.3768
14	H	-2.1279	1.5001	-0.4368
15	C	-0.0844	3.3099	-0.1674
16	H	-1.0573	3.6486	-0.5412
17	H	0.0849	3.8039	0.7996
18	H	0.6855	3.6868	-0.8534

SP_obn_an

Energy (POTENTIAL) = -385.7107601 Eh

	Atom	X	Y	Z
1	O	-1.0798	-3.1912	0.2112
2	C	0.0882	-2.5735	0.3632
3	H	0.6148	-2.7353	1.3661
4	H	0.9183	-2.8859	-0.3597
5	C	0.0708	-1.0272	0.2254
6	C	1.2238	-0.2417	0.3717
7	C	-1.1286	-0.3744	-0.0587
8	C	1.1758	1.1454	0.2383
9	H	2.1754	-0.7285	0.5910
10	C	-1.1831	1.0174	-0.1944
11	H	-2.0095	-1.0020	-0.1708
12	C	-0.0341	1.8034	-0.0465
13	H	2.0860	1.7333	0.3542
14	H	-2.1323	1.5032	-0.4197
15	C	-0.0837	3.3103	-0.1684
16	H	-1.0260	3.6456	-0.6154
17	H	0.0057	3.7987	0.8117
18	H	0.7371	3.6920	-0.7882

SP_obn_an_meoh_MeButa

Energy (POTENTIAL) = -501.4920305 Eh

	Atom	X	Y	Z
1	O	0.9634	-2.3772	-1.4221
2	C	1.6329	-1.4277	-0.7251
3	H	2.0021	-1.7575	0.2886
4	H	2.5800	-1.1013	-1.2351
5	C	0.8648	-0.1207	-0.4422
6	C	1.5015	0.9966	0.1175
7	C	-0.4970	-0.0171	-0.7360
8	C	0.8024	2.1744	0.3776
9	H	2.5661	0.9423	0.3481
10	C	-1.2012	1.1619	-0.4703
11	H	-0.9931	-0.8892	-1.1500
12	C	-0.5681	2.2781	0.0889
13	H	1.3230	3.0297	0.8073
14	H	-2.2641	1.2170	-0.7035
15	C	-1.3319	3.5459	0.4008
16	H	-2.2717	3.5962	-0.1601
17	H	-1.5856	3.6124	1.4680
18	H	-0.7457	4.4403	0.1567
19	O	-1.0796	-3.2862	-0.2329
20	H	-0.2177	-2.9169	-0.7108
21	C	-0.9379	-2.9985	1.1304
22	H	-0.7889	-1.9217	1.3335
23	H	-1.8485	-3.3067	1.6689
24	H	-0.0905	-3.5316	1.6073

obn_an_meoh_MeButa
Energy (POTENTIAL) = -501.317082876 Eh

	Atom	X	Y	Z
1	O	0.9634	-2.3772	-1.4221
2	C	1.6329	-1.4277	-0.7251
3	H	2.0021	-1.7575	0.2886
4	H	2.5800	-1.1013	-1.2351
5	C	0.8648	-0.1207	-0.4422
6	C	1.5015	0.9966	0.1175
7	C	-0.4970	-0.0171	-0.7360
8	C	0.8024	2.1744	0.3776
9	H	2.5661	0.9423	0.3481
10	C	-1.2012	1.1619	-0.4703
11	H	-0.9931	-0.8892	-1.1500
12	C	-0.5681	2.2781	0.0889
13	H	1.3230	3.0297	0.8073
14	H	-2.2641	1.2170	-0.7035
15	C	-1.3319	3.5459	0.4008
16	H	-2.2717	3.5962	-0.1601
17	H	-1.5856	3.6124	1.4680
18	H	-0.7457	4.4403	0.1567
19	O	-1.0797	-3.2862	-0.2329
20	H	-0.2177	-2.9169	-0.7108
21	C	-0.9379	-2.9985	1.1304
22	H	-0.7889	-1.9217	1.3335
23	H	-1.8485	-3.3067	1.6689
24	H	-0.0905	-3.5316	1.6073

obn_an_MeButa
Energy (POTENTIAL) = -385.559818597 Eh

	Atom	X	Y	Z
1	O	-1.0838	-3.1837	0.2102
2	C	0.0840	-2.5748	0.3629
3	H	0.6111	-2.7349	1.3681
4	H	0.9170	-2.8878	-0.3596
5	C	0.0732	-1.0270	0.2240
6	C	1.2236	-0.2401	0.3791
7	C	-1.1249	-0.3763	-0.0693
8	C	1.1748	1.1471	0.2478
9	H	2.1738	-0.7270	0.6057
10	C	-1.1804	1.0154	-0.2029
11	H	-2.0005	-1.0105	-0.1869
12	C	-0.0341	1.8030	-0.0448
13	H	2.0831	1.7370	0.3722
14	H	-2.1289	1.5009	-0.4336
15	C	-0.0841	3.3100	-0.1677
16	H	-1.0500	3.6481	-0.5594
17	H	0.0660	3.8024	0.8032
18	H	0.6988	3.6878	-0.8379

obn_an
Energy (POTENTIAL) = -385.574981019 Eh

	Atom	X	Y	Z
1	O	-1.0798	-3.1912	0.2112
2	C	0.0882	-2.5735	0.3633
3	H	0.6148	-2.7353	1.3661
4	H	0.9183	-2.8859	-0.3597
5	C	0.0708	-1.0272	0.2254
6	C	1.2238	-0.2417	0.3717
7	C	-1.1286	-0.3744	-0.0587
8	C	1.1758	1.1454	0.2383
9	H	2.1754	-0.7285	0.5910
10	C	-1.1831	1.0174	-0.1944
11	H	-2.0095	-1.0021	-0.1708
12	C	-0.0341	1.8034	-0.0465
13	H	2.0860	1.7333	0.3542
14	H	-2.1323	1.5032	-0.4197
15	C	-0.0837	3.3103	-0.1684
16	H	-1.0260	3.6456	-0.6154
17	H	0.0057	3.7987	0.8117
18	H	0.7371	3.6920	-0.7882

obn_rad_MeButa
Energy (POTENTIAL) = -385.438273659 Eh

	Atom	X	Y	Z
1	O	0.0687	-2.9249	-0.8990
2	C	0.1192	-2.5520	0.4298
3	H	-0.7457	-2.9123	1.0100
4	H	1.0528	-2.8608	0.9272
5	C	0.0696	-1.0300	0.2991
6	C	1.2510	-0.2973	0.1273
7	C	-1.1623	-0.3656	0.2426
8	C	1.2007	1.0851	-0.0336
9	H	2.2083	-0.8117	0.1323
10	C	-1.2053	1.0170	0.0814
11	H	-2.0843	-0.9329	0.3374
12	C	-0.0263	1.7660	-0.0555
13	H	2.1252	1.6457	-0.1488
14	H	-2.1666	1.5245	0.0564
15	C	-0.0759	3.2675	-0.1995
16	H	-0.9944	3.5922	-0.6993
17	H	-0.0507	3.7546	0.7845
18	H	0.7788	3.6442	-0.7709

obn_an_meoh_PMMA

Energy (POTENTIAL) = -501.309731375 Eh

	Atom	X	Y	Z
1	O	0.9631	-2.3802	-1.4194
2	C	1.6339	-1.4293	-0.7294
3	H	2.0100	-1.7538	0.2844
4	H	2.5779	-1.1006	-1.2451
5	C	0.8654	-0.1221	-0.4449
6	C	1.5008	0.9937	0.1188
7	C	-0.4957	-0.0178	-0.7417
8	C	0.8015	2.1711	0.3801
9	H	2.5650	0.9382	0.3516
10	C	-1.2001	1.1604	-0.4743
11	H	-0.9901	-0.8893	-1.1591
12	C	-0.5683	2.2753	0.0890
13	H	1.3212	3.0256	0.8132
14	H	-2.2627	1.2162	-0.7093
15	C	-1.3334	3.5416	0.4036
16	H	-2.2672	3.5982	-0.1671
17	H	-1.5994	3.5999	1.4685
18	H	-0.7437	4.4374	0.1732
19	O	-1.0846	-3.2821	-0.2279
20	H	-0.2222	-2.9196	-0.7085
21	C	-0.9369	-2.9900	1.1327
22	H	-0.7794	-1.9134	1.3318
23	H	-1.8483	-3.2894	1.6753
24	H	-0.0919	-3.5273	1.6101

SP_obn_an_MeButa

Energy (POTENTIAL) = -385.6944688 Eh

	Atom	X	Y	Z
1	O	-1.0838	-3.1837	0.2102
2	C	0.0840	-2.5748	0.3629
3	H	0.6111	-2.7349	1.3681
4	H	0.9170	-2.8878	-0.3596
5	C	0.0732	-1.0270	0.2240
6	C	1.2236	-0.2401	0.3791
7	C	-1.1249	-0.3763	-0.0693
8	C	1.1748	1.1471	0.2478
9	H	2.1738	-0.7270	0.6057
10	C	-1.1804	1.0154	-0.2029
11	H	-2.0005	-1.0105	-0.1869
12	C	-0.0341	1.8030	-0.0448
13	H	2.0831	1.7370	0.3722
14	H	-2.1289	1.5009	-0.4336
15	C	-0.0841	3.3100	-0.1677
16	H	-1.0500	3.6481	-0.5594
17	H	0.0660	3.8024	0.8032
18	H	0.6988	3.6878	-0.8379

obn_an_meoh

Energy (POTENTIAL) = -501.331188661 Eh

	Atom	X	Y	Z
1	O	0.9590	-2.3642	-1.4368
2	C	1.6265	-1.4254	-0.7151
3	H	1.9715	-1.7739	0.2989
4	H	2.5851	-1.1056	-1.2041
5	C	0.8623	-0.1170	-0.4347
6	C	1.5026	1.0028	0.1163
7	C	-0.5008	-0.0138	-0.7223
8	C	0.8044	2.1824	0.3725
9	H	2.5681	0.9508	0.3419
10	C	-1.2040	1.1670	-0.4613
11	H	-0.9991	-0.8872	-1.1307
12	C	-0.5674	2.2862	0.0885
13	H	1.3277	3.0397	0.7940
14	H	-2.2674	1.2215	-0.6916
15	C	-1.3277	3.5578	0.3923
16	H	-2.2744	3.6002	-0.1571
17	H	-1.5670	3.6375	1.4617
18	H	-0.7441	4.4482	0.1295
19	O	-1.0676	-3.2993	-0.2418
20	H	-0.2118	-2.9122	-0.7175
21	C	-0.9350	-3.0199	1.1266
22	H	-0.8187	-1.9411	1.3380
23	H	-1.8355	-3.3606	1.6608
24	H	-0.0721	-3.5315	1.5973

SP_obn_rad_PMMA

Energy (POTENTIAL) = -385.5457777 Eh

	Atom	X	Y	Z
1	O	0.0682	-2.9286	-0.8982
2	C	0.1192	-2.5520	0.4292
3	H	-0.7454	-2.9113	1.0108
4	H	1.0530	-2.8600	0.9271
5	C	0.0696	-1.0300	0.2985
6	C	1.2508	-0.2972	0.1274
7	C	-1.1620	-0.3655	0.2424
8	C	1.2006	1.0853	-0.0334
9	H	2.2080	-0.8117	0.1317
10	C	-1.2051	1.0171	0.0814
11	H	-2.0840	-0.9330	0.3363
12	C	-0.0262	1.7661	-0.0552
13	H	2.1251	1.6459	-0.1486
14	H	-2.1665	1.5246	0.0561
15	C	-0.0759	3.2678	-0.1990
16	H	-0.9943	3.5925	-0.6990
17	H	-0.0506	3.7550	0.7849
18	H	0.7785	3.6446	-0.7707

SP_obn_an_meoh_PMMA

Energy (POTENTIAL) = -501.4842842 Eh

	Atom	X	Y	Z
1	O	0.9631	-2.3802	-1.4194
2	C	1.6339	-1.4293	-0.7294
3	H	2.0100	-1.7538	0.2844
4	H	2.5779	-1.1006	-1.2451
5	C	0.8654	-0.1221	-0.4449
6	C	1.5008	0.9937	0.1188
7	C	-0.4957	-0.0178	-0.7417
8	C	0.8015	2.1711	0.3801
9	H	2.5650	0.9382	0.3516
10	C	-1.2001	1.1604	-0.4743
11	H	-0.9901	-0.8893	-1.1591
12	C	-0.5683	2.2753	0.0890
13	H	1.3212	3.0256	0.8132
14	H	-2.2627	1.2162	-0.7093
15	C	-1.3334	3.5416	0.4036
16	H	-2.2672	3.5982	-0.1671
17	H	-1.5994	3.5999	1.4685
18	H	-0.7437	4.4374	0.1732
19	O	-1.0846	-3.2821	-0.2279
20	H	-0.2222	-2.9196	-0.7085

21	C	-0.9369	-2.9900	1.1327
22	H	-0.7794	-1.9134	1.3318
23	H	-1.8483	-3.2894	1.6753
24	H	-0.0919	-3.5273	1.6101

obn_an_PMMA
Energy (POTENTIAL) = -385.551865436 Eh

	Atom	X	Y	Z
1	O	-1.0854	-3.1799	0.2059
2	C	0.0820	-2.5754	0.3626
3	H	0.6062	-2.7347	1.3705
4	H	0.9185	-2.8886	-0.3571
5	C	0.0745	-1.0269	0.2234
6	C	1.2240	-0.2395	0.3809
7	C	-1.1235	-0.3771	-0.0721
8	C	1.1749	1.1477	0.2503
9	H	2.1739	-0.7266	0.6091
10	C	-1.1794	1.0145	-0.2049
11	H	-1.9967	-1.0144	-0.1911
12	C	-0.0339	1.8028	-0.0441
13	H	2.0827	1.7384	0.3768
14	H	-2.1279	1.5001	-0.4368
15	C	-0.0844	3.3099	-0.1674
16	H	-1.0573	3.6486	-0.5412
17	H	0.0849	3.8039	0.7996
18	H	0.6855	3.6868	-0.8534

SP_obn_an_meoh
Energy (POTENTIAL) = -501.5071087 Eh

	Atom	X	Y	Z
1	O	0.9590	-2.3642	-1.4368
2	C	1.6265	-1.4254	-0.7151
3	H	1.9715	-1.7739	0.2989
4	H	2.5851	-1.1056	-1.2042
5	C	0.8623	-0.1170	-0.4347
6	C	1.5026	1.0028	0.1163
7	C	-0.5008	-0.0138	-0.7223
8	C	0.8044	2.1824	0.3725
9	H	2.5681	0.9508	0.3419
10	C	-1.2040	1.1670	-0.4613
11	H	-0.9991	-0.8872	-1.1307
12	C	-0.5674	2.2862	0.0885
13	H	1.3277	3.0398	0.7940
14	H	-2.2674	1.2215	-0.6916
15	C	-1.3277	3.5578	0.3923
16	H	-2.2744	3.6002	-0.1571
17	H	-1.5670	3.6375	1.4617
18	H	-0.7441	4.4482	0.1295
19	O	-1.0676	-3.2993	-0.2418
20	H	-0.2118	-2.9122	-0.7175
21	C	-0.9350	-3.0199	1.1266
22	H	-0.8187	-1.9411	1.3380
23	H	-1.8355	-3.3606	1.6608
24	H	-0.0721	-3.5315	1.5973

obn_rad
Energy (POTENTIAL) = -385.439530863 Eh

	Atom	X	Y	Z
1	O	0.0692	-2.9195	-0.8996
2	C	0.1192	-2.5522	0.4314
3	H	-0.7462	-2.9131	1.0097
4	H	1.0527	-2.8617	0.9278
5	C	0.0696	-1.0301	0.2993
6	C	1.2515	-0.2976	0.1265
7	C	-1.1628	-0.3658	0.2421
8	C	1.2011	1.0849	-0.0346
9	H	2.2091	-0.8114	0.1331
10	C	-1.2058	1.0168	0.0808
11	H	-2.0849	-0.9327	0.3388
12	C	-0.0263	1.7658	-0.0564
13	H	2.1255	1.6456	-0.1492
14	H	-2.1670	1.5244	0.0565
15	C	-0.0759	3.2672	-0.2000
16	H	-0.9947	3.5920	-0.6992
17	H	-0.0509	3.7529	0.7846
18	H	0.7793	3.6440	-0.7703

SP_obn_rad
Energy (POTENTIAL) = -385.5481546 Eh

	Atom	X	Y	Z
1	O	0.0692	-2.9195	-0.8996
2	C	0.1192	-2.5522	0.4314
3	H	-0.7462	-2.9131	1.0097
4	H	1.0527	-2.8617	0.9278
5	C	0.0696	-1.0301	0.2993
6	C	1.2515	-0.2976	0.1265
7	C	-1.1628	-0.3658	0.2421
8	C	1.2011	1.0849	-0.0346
9	H	2.2091	-0.8114	0.1331
10	C	-1.2058	1.0168	0.0808
11	H	-2.0849	-0.9327	0.3388
12	C	-0.0263	1.7658	-0.0564
13	H	2.1255	1.6456	-0.1492
14	H	-2.1670	1.5244	0.0565
15	C	-0.0759	3.2672	-0.2000
16	H	-0.9947	3.5920	-0.6992
17	H	-0.0509	3.7529	0.7846
18	H	0.7793	3.6440	-0.7703

SP_obn_rad_MeButa
Energy (POTENTIAL) = -385.5465302 Eh

	Atom	X	Y	Z
1	O	0.0687	-2.9249	-0.8990
2	C	0.1192	-2.5520	0.4298
3	H	-0.7457	-2.9123	1.0100
4	H	1.0528	-2.8608	0.9272
5	C	0.0696	-1.0300	0.2991
6	C	1.2510	-0.2973	0.1273
7	C	-1.1623	-0.3656	0.2426
8	C	1.2007	1.0851	-0.0336
9	H	2.2083	-0.8116	0.1323
10	C	-1.2053	1.0170	0.0814
11	H	-2.0843	-0.9329	0.3374
12	C	-0.0263	1.7660	-0.0555
13	H	2.1252	1.6457	-0.1488
14	H	-2.1666	1.5245	0.0564
15	C	-0.0759	3.2675	-0.1995
16	H	-0.9944	3.5922	-0.6993
17	H	-0.0507	3.7546	0.7845
18	H	0.7788	3.6442	-0.7709

obn_rad_PMMA

Energy (POTENTIAL) = -385.437685028 Eh

	Atom	X	Y	Z
1	O	0.0682	-2.9286	-0.8982
2	C	0.1192	-2.5520	0.4292
3	H	-0.7454	-2.9113	1.0108
4	H	1.0530	-2.8600	0.9271
5	C	0.0696	-1.0300	0.2985
6	C	1.2508	-0.2972	0.1274
7	C	-1.1620	-0.3655	0.2424
8	C	1.2006	1.0853	-0.0334
9	H	2.2080	-0.8117	0.1317
10	C	-1.2051	1.0171	0.0814
11	H	-2.0840	-0.9330	0.3363
12	C	-0.0262	1.7661	-0.0552
13	H	2.1251	1.6459	-0.1486
14	H	-2.1665	1.5246	0.0561
15	C	-0.0759	3.2678	-0.1990
16	H	-0.9943	3.5925	-0.6990
17	H	-0.0506	3.7550	0.7849
18	H	0.7785	3.6446	-0.7707

Bibliography

1. Frisch, M. J.; Trucks, G. W.; Schlegel, H. B.; Scuseria, G. E.; Robb, M. A.; Cheeseman, J. R.; Scalmani, G.; Barone, V.; Mennucci, B.; Petersson, G. A.; Nakatsuji, H.; Caricato, M.; Li, X.; Hratchian, H. P.; Izmaylov, A. F.; Bloino, J.; Zheng, G. *Gaussian 09*, Revision A.1; Gaussian, Inc.: Wallingford, CT, 2009.
2. Becke, A. D. Density-Functional Thermochemistry. III. The Role of Exact Exchange. *J. Chem. Phys.* **1993**, *98*, 5648–5652. https://doi.org/10.1063/1.464913.
3. Becke, A. D. Density-Functional Thermochemistry. IV. A New Dynamical Correlation Functional and Implications for Exact-Exchange Mixing. *J. Chem. Phys.* **1993**, *98*, 1372–1377. https://doi.org/10.1063/1.464304.
4. Lee, C.; Yang, W.; Parr, R. G. Development of the Colle–Salvetti Correlation-Energy Formula into a Functional of the Electron Density. *Phys. Rev. B* **1988**, *37*, 785–789. https://doi.org/10.1103/PhysRevB.37.785.
5. Grimme, S. Density Functional Theory with London Dispersion Corrections. *WIREs Comput. Mol. Sci.* **2011**, *1*, 211–228. https://doi.org/10.1002/wcms.30.
6. Hehre, W. J.; Ditchfield, R.; Pople, J. A. Self-Consistent Molecular Orbital Methods. XII. Further Extensions of Gaussian-Type Basis Sets for Use in Molecular Orbital Studies of Organic Molecules. *J. Chem. Phys.* **1972**, *56*, 2257–2261. https://doi.org/10.1063/1.1677527.
7. Cancès, E.; Mennucci, B.; Tomasi, J. A New Integral Equation Formalism for the Polarizable Continuum Model: Theoretical Background and Applications to Isotropic and Anisotropic Dielectrics. *J. Chem. Phys.* **1997**, *107*, 3032–3041. https://doi.org/10.1063/1.474659.
8. Nikolov, I. D.; Ivanov, C. D. Optical Plastic Refractive Measurements in the Visible and the Near-Infrared Regions. *Appl. Opt.* **2000**, *39*, 2067–2070. https://doi.org/10.1364/AO.39.002067.
9. Neese, F. Software Update: The ORCA Program System, Version 6.0. *WIREs Comput. Mol. Sci.* **2025**, *15*, e70019. https://doi.org/10.1002/wcms.70019.
10. Weigend, F.; Ahlrichs, R. Balanced Basis Sets of Split Valence, Triple Zeta Valence and Quadruple Zeta Valence Quality for H to Rn: Design and Assessment of Accuracy. *Phys. Chem. Chem. Phys.* **2005**, *7*, 3297–3305. https://doi.org/10.1039/B508541A.
11. Neese, F. An Improvement of the Resolution of the Identity Approximation for the Formation of the Coulomb Matrix. *J. Comput. Chem.* **2003**, *24*, 1740–1747. https://doi.org/10.1002/jcc.10318.
12. Álvarez-Moreno, M.; De Graaf, C.; López, N.; Maseras, F.; Poblet, J. M.; Bo, C. Managing the Computational Chemistry Big Data Problem: The IoChem-BD Platform. *J. Chem. Inf. Model.* **2015**, *55* (1), 95–103. https://doi.org/10.1021/ci500593j.
13. Ji, P.; Powles, N. T.; Atherton, J. H.; Page, M. I. Ionization of Carbon Acids in Liquid Ammonia. *Org. Lett.* **2011**, *13*, 6118–6121. https://doi.org/10.1021/ol2026153.
14. Li, J.; Lear, M. J.; Hayashi, Y. Sterically Demanding Oxidative Amidation of α-Substituted Malononitriles with Amines Using O_2. *Angew. Chem. Int. Ed.* **2016**, *55*, 9060–9064. https://doi.org/10.1002/anie.201603399.
15. Xu, D.; Huang, R.; He, Y.; Wang, M.; Wang, Y. Mechanogated PINO Radical Organocatalysis: Catalytic Amplification of Mechanical Force. *ACS Macro Lett*. **2026**, *15 (5)*, 738–746. https://doi.org/10.1021/acsmacrolett.6c00170
